# Novel A-Ring Chalcone Derivatives of Oleanolic and Ursolic Amides with Anti-Proliferative Effect Mediated through ROS-Triggered Apoptosis

**DOI:** 10.3390/ijms22189796

**Published:** 2021-09-10

**Authors:** Elmira Khusnutdinova, Anastasiya Petrova, Zulfia Zileeva, Ulyana Kuzmina, Liana Zainullina, Yulia Vakhitova, Denis Babkov, Oxana Kazakova

**Affiliations:** 1 Ufa Institute of Chemistry UFRC RAS, 71 pr. Oktyabrya, 450054 Ufa, Russia; ElmaH@inbox.ru (E.K.); ana.orgchem@gmail.com (A.P.); 2 Institute of Biochemistry and Genetics UFRC RAS, 71 pr. Oktyabrya, 450054 Ufa, Russia; zileeva81@list.ru (Z.Z.); ulia-bio@yandex.ru (U.K.); zainullinalf@gmail.com (L.Z.); juvv73@gmail.com (Y.V.); 3 Scientific Center for Innovative Drugs, Volgograd State Medical University, 39 Novorossiyskaya St., 400087 Volgograd, Russia; denis.a.babkov@gmail.com

**Keywords:** oleanolic acid, ursolic acid, Claisen-Schmidt reaction, anticancer activity, NCI-60, CellMiner, network-like similarity graphs, apoptosis

## Abstract

A series of A-ring modified oleanolic and ursolic acid derivatives including C28 amides (3-oxo-C2-nicotinoylidene/furfurylidene, 3β-hydroxy-C2-nicotinoylidene, 3β-nicotinoyloxy-, 2-cyano-3,4-seco-4(23)-ene, indolo-, lactame and azepane) were synthesized and screened for their cytotoxic activity against the NCI-60 cancer cell line panel. The results of the first assay of thirty-two tested compounds showed that eleven derivatives exhibited cytotoxicity against cancer cells, and six of them were selected for complete dose–response studies. A systematic study of local SARs has been carried out by comparative analysis of potency distributions and similarity relationships among the synthesized compounds using network-like similarity graphs. Among the oleanane type triterpenoids, C2-[4-pyridinylidene]-oleanonic C28-morpholinyl amide exhibited sub-micromolar potencies against 15 different tumor cell lines and revealed particular selectivity for non-small cell lung cancer (HOP-92) with a GI_50_ value of 0.0347 μM. On the other hand, superior results were observed for C2-[3-pyridinylidene]-ursonic *N*-methyl-piperazinyl amide **29**, which exhibited a broad-spectrum inhibition activity with GI_50_ < 1 μM against 33 tumor cell lines and <2 μM against all 60 cell lines. This compound has been further evaluated for cell cycle analysis to decipher the mechanism of action. The data indicate that compound **29** could exhibit both cytostatic and cytotoxic activity, depending on the cell line evaluated. The cytostatic activity appears to be determined by induction of the cell cycle arrest at the S (MCF-7, SH-SY5Y cells) or G_0_/G_1_ phases (A549 cells), whereas cytotoxicity of the compound against normal cells is nonspecific and arises from apoptosis without significant alterations in cell cycle distribution (HEK293 cells). Our results suggest that the antiproliferative effect of compound **29** is mediated through ROS-triggered apoptosis that involves mitochondrial membrane potential depolarization and caspase activation.

## 1. Introduction

**Figure 1:**
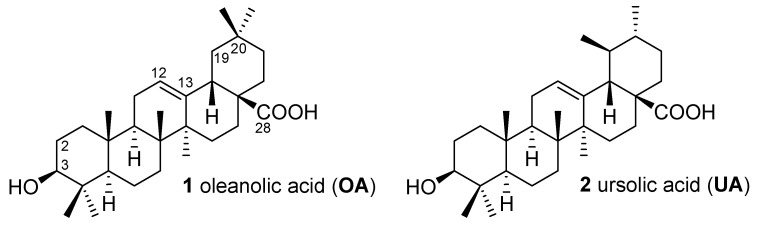
Structures of oleanolic (**1**) and ursolic (**2**) acids.

Plants have always played an important role in human health care [1]. The discovery of novel bioactive compounds from natural plants is one of the most effective trends in natural product research [2]. Among these, naturally occurring triterpenoids have found direct application as drug entities and play an important role as templates for the design, synthesis, and semi-synthesis of novel substances [3,4]. Pentacyclic triterpenoids, such as oleanolic (**1**) and ursolic (**2**) acids, contain a biologically active scaffold with a high safety profile in cancer therapy and are suitable to carry out different chemical transformations as several key positions (C2, C3, C12, C13, and C28, Figure 1). This inspires scientists to develop new methods for the chemical modification of the triterpene core, or to use well-known approaches to expand the effective pharmacological agents among the different types of triterpenoids.

Generally, the C2 position of pentacyclic triterpenoids is a preferential site to carry out modification and to prepare analogs with better anticancer activities than the parent acids [5,6]. The principles of oleanolic acid modification, specifically the formation of a 2-cyano-1-en-3-one on the A-ring, the modification of the C-ring by converting the 12(13)-ene to 12-oxo-9(11)-en and/or methyl esterification or formation of the imidazolide group of the C28 carboxylic group, has been known since 2000 and led to the development of the effective anticancer agents CDDO, CDDO-Me (methyl bordoxolone), and CDDO-Im, which are under clinical trials [7,8,9,10]. Similar chemical modifications have been conducted to other triterpene cores to improve their potency and overcome the drawbacks. For example, glycyrrhetinic acid has been used for the synthesis of CDDO analogs (soloxolones), which significantly improved cytotoxicity [11,12,13], and recently the effective inhibition by methyl soloxolone TGF-β-driven EMT of tumor cells was shown [14]. The same ursane-type analogs were obtained by an oxidative ozonolysis-mediated C-ring enone formation with a potency of approximately five-fold less than the corresponding oleanolic acid derivatives [15].

The Claisen–Schmidt aldol condensation giving 3-oxo-C2-benzylidene (or chalcone) triterpenoids is an efficient type of chemical modification and the first mentions of the physical and chemical properties of such derivatives date back to 1957 by D. H. R. Barton et al. [16]. Nowdays, these compounds demonstrate high potential as antibacterial and anti-inflammatory [17,18], antioxidant [19], antidiabetic [20,21,22,23], and cytotoxic agents [24,25,26,27,28,29]. Most of the C2-benzylidenes among the triterpenoids described are derivatives with the carboxylic group at C28 [24,28,29]. The strategy for modification of both the A-ring with chalcone introduction at C2 and derivatization of 28-COOH seems to be attractive because the obtained hybrid molecules while bearing two different pharmacophores can demonstrate high biological potential. For example, derivatives of the ursane type with *p*-chlorine-benzylidene- or 4-pyridynilidene- at C2 and nitrooxy ethyl substituents at C28 were found to be more cytotoxic than the parent drug (IC_50_ ranged between 4.28–12.74 μm) and the lead derivatives could induce cell cycle arrest at the G1 phase and apoptosis in a dose-dependent manner via caspase-8 activation [27,30].

The introduction of heterocyclic fragments, especially piperazine, has been demonstrated to enhance anticancer properties [31]. Recently we have found that modification of triterpenoids to indole derivatives on the A-ring with amidation to C28-amide, as well as the introduction of piperazine or *N*-methyl-piperazine, have a positive effect on anticancer activity [32,33]. Chalcone derivatives of messagenine and platanic acid [25], polyamino-lupanes [34], A-azepano-, and 3-amino-3,4-seco-triterpenoids [35] were also found to be effective antiproliferative agents against different cancer cell lines with submicromolar concentration values of GI_50_ < 1 μM.

Thus, the chemical controlled modification of triterpenoids using known methods [36,37], followed by their screening toward NCI-60 cancer cell panel [28,38,39], i.e., 60 lines from 9 types of cancer types, is still an attractive approach to obtain the SAR data of large series of tested compounds and to identify the lead derivative(s) with high antiproliferative activity and with selectivity against some types of cancers. Hence, we describe herein the synthesis of a new series of A-ring modified oleanolic and ursolic acids and their amides, the screening of their cytotoxic activity, as well as the clarification of the mechanism of action and a systematic study of local SAR analysis.

## 2. Results and Discussion

### 2.1. Chemistry

**Scheme 1:**
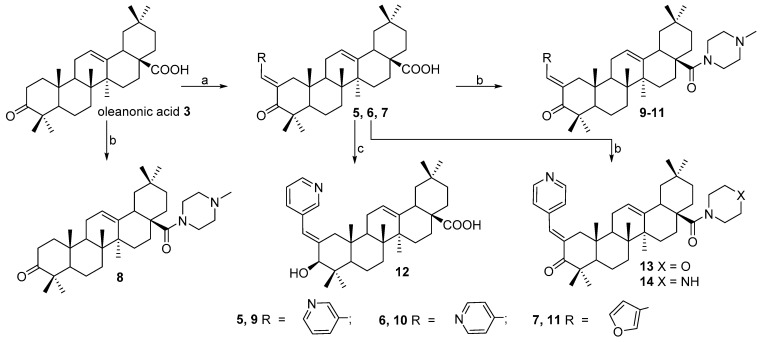
Synthesis of oleanolic acid derivatives **5**–**14**: (**a**) 3- or 4-pyridinecarboxaldehyde or furfural, 40% KOH, EtOH, rt, 8 h; (**b**) i (COCl)_2_, CH_2_Cl_2_, rt, 3 h; ii N-methylpiperazine, piperazine or morpholine, CH_2_Cl_2_, Et_3_N, ∆, 5 h; (**c**) NaBH_4_, i-PrOH, rt, 3 h.

**Scheme 2:**
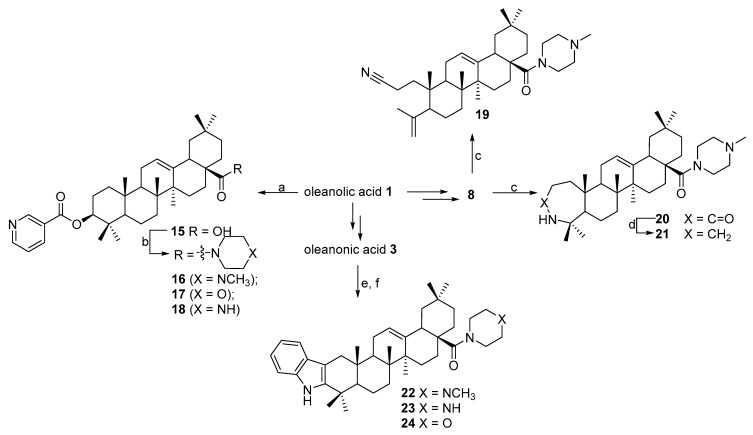
Synthesis of oleanolic acid derivatives **15**–**24**: (**a**) C_5_H_4_NCOCl, pyridine-Bu_3_N, rt, 4 h; (**b**) i (COCl)_2_, CH_2_Cl_2_, rt, 3 h; ii N-methylpiperazine, piperazine or morpholine, CH_2_Cl_2_, Et_3_N, ∆, 5 h; (**c**) i NH_2_OH∙HCl, NaOAc, EtOH, ∆, 4 h, 80–82%; ii SOCl_2_, dioxane, 1 h, rt; (**d**) LiAlH_4_, THF, 66 °C, 1 h; (**e**) PhNHNH_2_, AcOH, ∆, 5 h; (**f**) i (COCl)_2_, CH_2_Cl_2_, rt, 3 h; ii N-methylpiperazine, piperazine or morpholine, CH_2_Cl_2_, Et_3_N, ∆, 5 h.

**Scheme 3:**
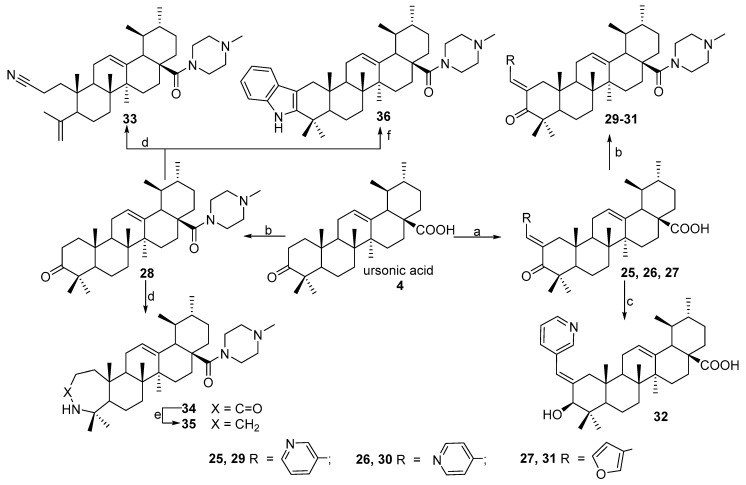
Synthesis of ursolic acid derivatives **25**–**36**: (**a**) 3- or 4-pyridinecarboxaldehyde or furfural, 40% KOH, EtOH, rt, 8 h; (**b**) i (COCl)_2_, CH_2_Cl_2_, rt, 3 h; ii N-methylpiperazine, CH_2_Cl_2_, Et_3_N, ∆, 5 h; (**c**) NaBH_4_, i-PrOH, rt, 3 h; (**d**) i NH_2_OH∙HCl, NaOAc, EtOH, ∆, 4 h, 80–82%; ii SOCl_2_, dioxane, 1 h, rt; (**e**) LiAlH_4_, THF, 66 °C, 1 h; (**f**) PhNHNH_2_, AcOH, ∆, 5 h.

We designed a new series of oleanonic (3) and ursonic (4) acids derivatives by the introduction of nitrogen-containing heterocycles to C2, C3, and C28 positions or A-ring skeleton transformation. (3- or 4)-Pyridinylidene and furfurylidene fragments were coupled at the C2-position; the nicotinoyloxy-fragment was introduced at C3-position; N-methylpiperazinyl-, piperazinyl- and morpholinyl-amides were synthesized at the C28-position. Modification of the A-ring included a Fischer indolization reaction to indole-fused derivatives, and Beckmann rearrangement to seven-membered lactame (with the following modification onto azepanes) and 2-nitrilo-3,4-seco-4(23)-en-derivatives were carried out. The synthesis of all of the mentioned derivatives **5**–**36** is presented on Scheme 1, Scheme 2 and Scheme 3.

The Claisen-Schmidt reaction of 3-oxo-acids **3** and **4** with 3- or 4-pyridinecarboxaldehydes or furfural afforded C2-nicotinoylidene/furfurylidene derivatives **5**–**7** and **25**–**27** in good yield (81–87%). Compounds **5** and **25** were reduced using sodium borohydride to afford 3β-hydroxy derivatives **12** and **32** (Scheme 1 and Scheme 3).

Acylation of cyclic amines (N-methyl-piperazine, piperazine or morpholine) by triterpenic acid chloride of 3-oxo-**3**–**7** and C2-nicotinoylidene/furfurylidene **25**–**27** derivatives led to amides of oleanane type **8**–**11**, **13**, and **14** and ursane type **28**–**31** in yields of 65–78% (Scheme 1, Scheme 2 and Scheme 3).

The esterification of oleanolic acid (**1**) by nicotinic acid chloride in a pyridine-tributylamine media under reflux led to 3β-nicotinoyloxy-derivative **15**, which was further transformed into appropriate amide analogs **16**–**18**.

The next series involving A-ring transformations is shown at Scheme 2 and Scheme 3. A-seco-4(23)-enes **19**, **33** and lactams **23**, **43**, **45**, **56** were obtained by a Beckmann rearrangement of the corresponding C3-oximes using SOCl_2_ in dioxane. Reduction of lactams **20** and **34** with lithium aluminum hydride in THF under reflux afforded azepanes **21** and **35** with 56–60% yield. Indoles **22**–**24** and **36** were obtained from oleanonic **3** and ursonic **4** acids using a Fischer reaction followed by amidation at the C28-position. The structures of the compounds were ascertained by the combined use of spectroscopy and elemental analyses.

Thus, a series of triterpenic acids with modified A-ring (3-oxo-, 3-oxo-C2-nicotinoylidene/furfurylidene, 3β-hydroxy-C2-nicotinoylidene-, 3β-nicotinoyloxy-, 2-cyano-3,4-seco-4(23)-ene, indolo-, lactame and azepane) and their C28 amides was synthesized.

### 2.2. Biological Evaluation

#### 2.2.1. NCI-60 Anticancer Drug Screening

Compounds **5**–**36** were selected by the National Cancer Institute (NCI) Developmental Therapeutic Program (www.dtp.nci.nih.gov, accessed on 16 October 2019) for the in vitro cell line screening to investigate their anticancer activity. Anticancer assays were performed according to the US NCI protocol, which was described elsewhere [40,41,42,43,44,45]. Compounds **5**–**36** were evaluated against 58 human tumor cell lines, which were derived from nine different cancer types: leukemia, melanoma, lung, colon, CNS, ovarian, renal, prostate, and breast cancers. At first, compounds were tested at a single high dose concentration (10 μM), and according to the criterion adopted by the NCI, compounds that reduced the growth of any of the cell lines to approximately 32% or less were considered to be active.

The results of this first assay showed that among all series of tested compounds oleanolic acid derivatives **7**, **9**, **10**, **12**, **13**, **17**, and **23**, as well as ursolic acid derivatives **27**, **29**, **31**, and **32** showed cytotoxicity against cancer cells. Compounds **5**, **6**, **8**, **11**, **14**–**16**, **18**–**22**, **24**–**26**, **28**, **30**, and **33**–**36** were not active (the percent of cell growth was over 32%). Results for each compound in the single-dose assay are reported in Tables S1–S3 (see Supporting Information).

Compounds **7**, **12**, **13**, **27**, **29**, and **32** were selected for complete dose–response studies with five different test concentrations (0.01, 0.1, 1, 10, and 100 μM). The dose–response curves (% growth vs. sample concentration) of these compounds against each cell line in the NCI screening, NMR data as well, can be found in the Supporting Information (Figures S1–S30). A comparative summary of the single-dose mean growth inhibition (%) for all active compounds, and for those that passed the initial one-dose screening test, the mean (GI_50_, μM) and the most sensitive cell line is provided in Table 1.

According to the first stage results which are presented in Table 1, the following cancer cell lines: melanoma (LOX IMVI), leukemia (HL-60(TB), SR), non-small lung cancer (NCI-H460), and colon cancer (HT29) were the most sensitive to oleanolic acid derivatives with a growth percent range from −95.08% to 25.52%. Melanoma (SK-MEL-5), renal cancer (UO-31), and colon cancer (COLO-250) cell lines were the most sensitive to ursolic acid derivatives with a growth percent range from −99.47% to −80.36%.

Compounds **7**, **13**, **27**, and **29** displayed high cytotoxic activity with a mean GI_50_ < 5 μM. The log mean values of the parameter for GI_50_, TGI, and LC_50_ related to the log values (the maximum sensitivity in excess of the mean) and log range values are given in Table 2. These parameters highlight the selectivity and potency of antitumor agents. Higher values of these deltas and ranges indicate high selectivity against some cancers over others. The lower median log GI_50_ value (−6.12) for compound **29** showed it to be the most potent compound for all cell lines. The effective growth inhibition of compound **13** (−5.81) also accounts for its high range log GI_50_ and log LC_50_ values with 2.3 and 1.23 respectively, among all 60 cell lines.

Compounds **13** and **29** displayed the most potent cytotoxic activity with significant inhibition for most of the 60 cell lines, and their mean GI_50_, TGI (concentration of compound that totally inhibits cell growth), and LC_50_ (concentration of compound that kills 50% of cells) values across each cell line are shown in Table 3.

Thus, compound **13** exhibited a broad spectrum of antiproliferative activity with a GI_50_ of <4 μM for 93% and <1 μM for 25% of the tested cell lines. Strong growth inhibition (GI_50_ < 1 μM) was observed against all leukemia cell lines with (from 0.365 μM to 0.891 μM), as well as against colon cancer (HCT-116 0.319 μM, HCT-115 0.939 μM, HT-29 0.912 μM), against renal cancer (UO-31 0.893 μM), against prostate cancer (PC-3 0.357 μM), and breast cancer (MCF7 0.509 μM, T-47D 0.781 μM, MDA-MB-468 0.712 μM) cell lines. The highest activity was observed for non-small cell lung cancer cell lines HOP-92 with a GI_50_ value of 0.0347 μM. Among the tumor subpanels, selectivity greater than 80-fold and 125-fold was observed between EKVX, NCI-H322M, and HOP-92 in the non-small cell lung cancer panel. With respect to the total growth inhibition effect of **13**, the HL-60(TB) and RPMI-8226 (TGI 3.96 μM and 3.36 μM, leukemia), NCI-H460 8226 (TGI 4.17 μM, NSCLC), HCT-116 (TGI 2.00 μM, colon), SF-539 (TGI 3.70 μM, CNS), RXF 393 (TGI 3.03 μM, renal), LOX IMVI, and SK-MEL-5 (TGI 2.72 μM and 3.01 μM, melanoma) cell lines were the most sensitive. At the LC_50_ level of cytotoxicity, most cell lines were not sufficiently impacted at the high test concentration of 100 μM, with the exception of HCT-116 colon cancer (LC_50_ 6.95 μM), SF-539 CNS cancer (LC_50_ 7.94 μM), LOX IMVI (LC_50_ 5.95 μM), SK-MEL-5 (LC_50_ 6.15 μM) melanoma, and RXF 393 (LC_50_ 7.53 μM) renal cancer cell lines. The HCT-116 colon cancer cell line is the most sensitive to compound **13** based on all three GI_50_, TGI, and LC_50_.

The GI_50_ value of compound **29** was <1 μM against 33 tumor cell lines and <2 μM against all cell lines. The highest activity was observed against all leukemia cell lines with GI_50_ value ranged from 0.171 μM to 0.360 μM, against colon cancer cell lines with GI_50_ value ranged from 0.332 μM to 0.924 μM and prostate PC-3 cancer cell line with GI_50_ 0.275 μM.

Based on a total inhibition value HL-60(TB) (TGI 0.981 μM) and RPMI-8226 (TGI 0.541 μM), leukemia cell lines were the most sensitive, as well as <2 μM against NSCL (HOP-92, NCI-H460, NCI-H522), colon (HCT-116, HT-29, SW-620), CNS (U251), melanoma (M14), ovarian (OVCAR-3), renal (UO-31), prostate (PC-3), and breast (MDA-MB-468) cancer cell lines. Taking into account the data of GI_50_, TGI, and LC_50_, compound **29** was the most efficient against HL-60 (TB) leukemia, HOP-92 NSCL, and PC-3 prostate cancer cell lines.

A comparison of obtained results for leader compounds with respect to the activity reported for the standard drug doxorubicine, used by NCI as control [46], reflects that compound **29** showed activity against prostate cancer PC-3 (GI_50_ = 0.275 μM) which is comparable with a standard drug (GI_50_ = 0.32 μM), as well as against colon cancer HCT-15 compounds **13** (GI_50_ = 0.939 μM) and **29** (GI_50_ = 0.654 μM) showed comparable activity, while this value was 0.95 μM for the standard drug doxorubicine. The highest activity was observed for compound **13** against non-small cell lung cancer HOP-92 (GI_50_ = 0.0347 μM), that is three-fold times more effective than for doxorubicine (GI_50_ = 0.10 μM), as well as against HCT-15 colon cancer cell line compound **13** was 10-fold (GI_50_ = 0.654 μM) and **29** (GI_50_ = 1.20 μM) was 5-fold more effective then doxorubicine (GI_50_ = 6.46 μM).

#### 2.2.2. Mechanisms In Vitro Studies

##### Cell Cycle Analysis

**Figure 2:**
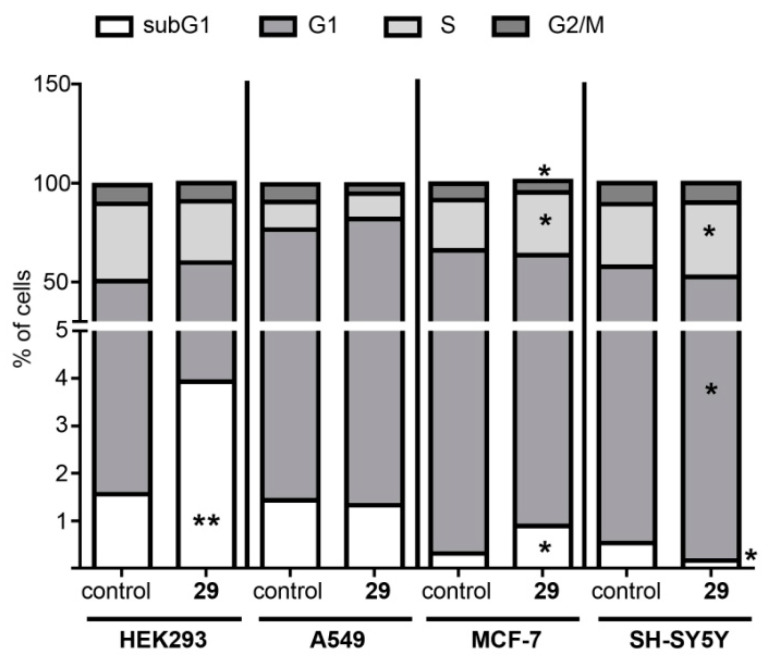
Cell cycle progression of HEK293, A549, MCF-7, and SHSY5Y cells upon compound **29** treatment. Data are expressed as mean ± S.E.M from three experiments, performed in triplicate. *—*p* < 0.05 vs. corresponding control (untreated) cells (Wilcoxon *t*-test); **—*p* < 0.01 vs. corresponding control (untreated) cells (Wilcoxon *t*-test).

**Figure 3:**
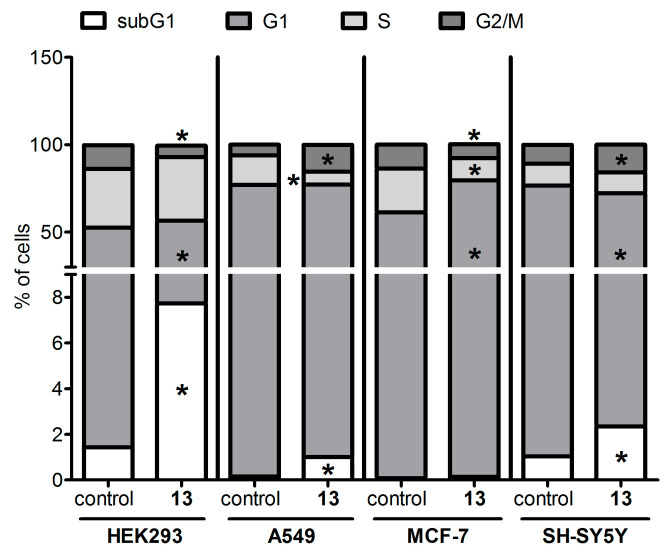
Cell cycle progression of HEK293, A549, MCF-7, and SHSY5Y cells upon compound **13** treatment. Data are expressed as mean ± S.E.M from three experiments, performed in triplicate. *—*p* < 0.05 vs. corresponding control (untreated) cells (Wilcoxon *t*-test).

Compounds **13** and **29**, found to be the most potent in the NCI cytotoxicity screening, have been further evaluated for cell cycle analysis to clarify the mechanisms of their action. PI (propidium iodide) staining followed by flow cytometry was performed to assess the cell cycle progression in response to compounds **13** and **29** exposure in cancerous (lung adenocarcinoma A549, breast adenocarcinoma MCF-7, neuroblastoma SH-SY5Y) and conditionally-normal (human embryonic kidney HEK293) cells. For cell cycle analysis, compounds **13** and **29** were used at their IC_50_ values, which were previously established for the aforementioned cell lines in the additional laboratory cytotoxicity screen (see Table S4, Supporting Information). Treatment of HEK293 cells with compound **29** (14.7 µM) for 48 h increased the number of apoptotic cells (detected on sub-G_1_ peak) with no significant changes to cell cycle pattern compared with control (0.1% DMSO-treated) cells, indicated the apoptosis induction (Figure 2). A549 cells upon the compound’s exposure (14.7 µM) displayed a moderate increase in the percentage of cells in the G_0_/G_1_ phase accompanied by the decline of cells number in the G_2_/M phase. Compound **29** (11.1 µM) caused the MCF-7 cells to undergo the cell cycle arrest in the S phase, a notable decrease of the proportion of cells in the G_2_/M phase, and an increase of apoptotic cells compared to the control (0.1% DMSO-treated) group (Figure 2). A similar action of compound **29** (14.6 µM) has been established towards SH-SY5Y cells: an elevation of cells in the S phase followed by a subsequent reduction in the number of cells in the G_1_ phase and a significant decrease of apoptotic cells compared to the control (0.1% DMSO-treated) group (Figure 2). Compound **13** (33 µM; 48 h of incubation) elicited a notable increase in thepercentage of sub-G_1_ cells indicating an apoptosis induction (Figure 3). A G_2_/M phase arrest with an accumulation of apoptotic cells was detected in A549 (61.1 µM) and SH-SY5Y (38.1 µM) cells. In MCF-7 cells, substance **13** (67.7 µM) evoked an arrest in the G_0_/G_1_ phase, a decline of the proportion of cells in the S and G_2_/M phases, and an increase in sub-G_1_ (Figure 3). Overall, these data indicate that compounds **29** and **13** could exhibit both cytostatic and cytotoxic activity, depending on the cell line evaluated. The cytostatic activity appears to determine by induction of the cell cycle arrest at the S (compound **29** in MCF-7, SH-SY5Y cells), G_0_/G_1_ (compound **29** in A549 cells; compound **13** in MCF-7 cells), or G_2_/M phases (compound **13** in A549 and SH-SY5Y cells), whereas cytotoxicity of both compounds arises on the triggering of apoptosis without significant alterations in cell cycle distribution (HEK293 cells).

##### The Cell Apoptosis Assay for Compound **29**

In addition, we have examined compound **29**-induced apoptosis in HEK293 and MCF-7 cells by Annexin V/SYTOX staining followed by flow cytometry. This approach allows the distinguishing of the early and late apoptotic cells. We have found that HEK293 and MCF-7 cells, treated for 24 h with compound **29**, exhibited a moderate increase both of early and late apoptotic cells in (Table 4), and when the treatment with **29** was maintained for 48 h, a pronounced augmentation of late apoptotic cells has been observed. Thus, these data indicate and confirm that compound **29** causes a significant time-dependent increase of apoptosis in HEK293 and MCF-7 cells.

##### Measurement of Intracellular Reactive Oxygen Species Level for Compound **29**

**Figure 4:**
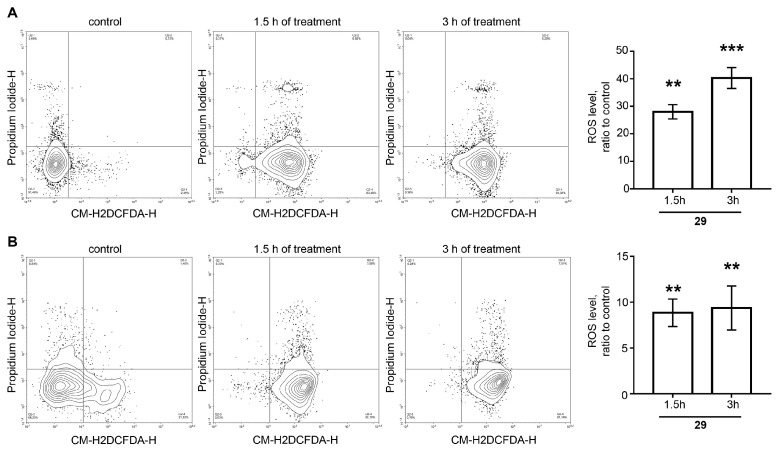
Effect of compound **29** on ROS generation in HEK293 (**A**) and MCF-7 (**B**) cells. Cells were incubated with compound **29** for 1.5 and 3 h. After incubation, compound cells were stained with CM-H2DCFDA, and intracellular ROS level was measured. Data are expressed as mean ± S.E.M from two independent experiments, performed in triplicate (**—*p* < 0.01, ***—*p* < 0.001 compared to control (untreated) cells; Wilcoxon *t*-test).

Excessive reactive oxygen species (ROS) production and associated mitochondrial disruption is known to result in oxidative stress and subsequent cell apoptosis [47]. Moreover, ROS have been demonstrated to be highly reactive species that cause DNA damage [48]. To evaluate whether compound **29**-induced apoptotic cell death was mediated by ROS generation, levels of intracellular ROS were estimated using 2′,7′-dichlorofluorescein diacetate (CM-H2DCFDA) as a fluorescent probe. Figure 4 shows a significant time-dependent increase in ROS accumulation in HEK293 cells, treated with compound **29** (~28 folds after 1.5 h and ~40 folds after 3 h), while in MCF-7 cells ROS generation was less pronounced (~8 folds after 1.5 h and ~9 folds after 3 h).

**Figure 5:**
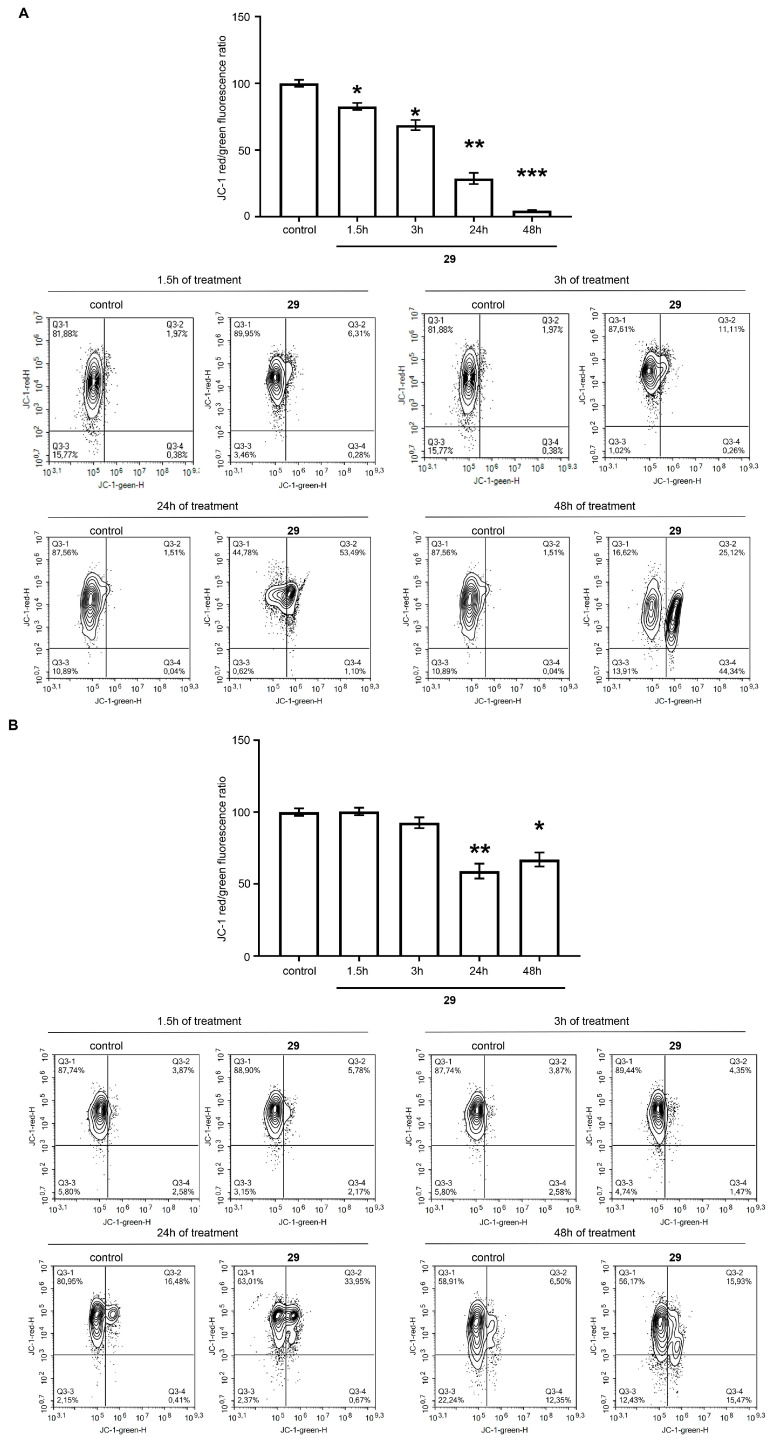
Mitochondrial membrane depolarization in HEK293 (**A**) and MCF-7 (**B**) cells upon compound **29** treatment. Cells were treated with compound **29** for 1.5, 3, 24 and 48 h. Data are expressed as mean ± S.E.M from two experiments, performed in triplicate (*—*p* < 0.05, **—*p* < 0.01, ***—*p* < 0.001 compared with control (untreated) cells; Wilcoxon *t*-test).

Considering the dissipation of mitochondrial membrane potential (MMP) as the earliest event of the apoptotic cascade and as one of the specific signs of apoptosis [49], we used the JC-1 cationic dye to detect the changes of mitochondrial membrane potential in HEK293 and MCF-7 cells upon compound **29** treatments. As demonstrated in Figure 5A, a progressive time-dependent decrease in the red/green fluorescence intensity ratio in HEK293 cells was observed after the substance treatment (14.7 µM), indicating the mitochondrial membrane depolarization. In MCF-7 cells, compound **29** (11.1 µM) evoked a moderate decline of red/green ratio in a time course-dependent manner (Figure 5B), suggesting a reduction of mitochondrial membrane potential, although to a lesser degree compared with that in HEK293 cells.

##### Caspase 8, 9 Activity Assay for Compound **29**

**Figure 6:**
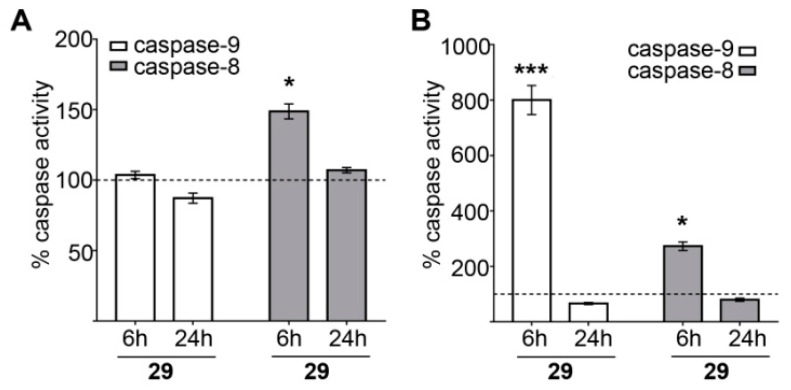
Caspases activity in HEK293 (**A**) and MCF-7 (**B**) cells upon compound **29** treatment. Cells were treated with **29** for 6 and 24 h. The activity is given as a percentage of the control group (untreated cells) considered 100%. Data are expressed as mean ± S.E.M from two experiments, performed in triplicate (*—*p* < 0.05, ***—*p* < 0.001 compared with corresponding control cells (shown as a dotted line); Wilcoxon *t*-test).

It is well-established that apoptosis can be triggered through two major pathways: the cell death receptor-mediated extrinsic pathway and the mitochondrial-mediated intrinsic pathway, resulting in activation of caspase-8 and caspase-9, respectively, followed by induction of the downstream executioner caspases-3/7 [50]. The intrinsic apoptosis pathway is initiated by mitochondrial alterations culminating in the release of mitochondrial cytochrome c with a concomitant reduction of the mitochondrial transmembrane potential. To further evaluate whether compound **29** preferentially affects the extrinsic and/or intrinsic apoptotic pathways, the activities of initiator caspases 8 and 9 were assessed. As shown in Figure 6A, compound **29** did not affect caspase-9 activity, whereas caspase-8 activity was increased after 6 h of compound’ treatment in HEK293 cells. Interestingly, in MCF-7 cells the substance caused a marked rise of caspase-9 and a less pronounced increase of caspase-8 activities at the 6 h time point (Figure 6B). Notably, the highest increment in values of caspase activity was observed for caspase-8 in HEK293 cells and caspase-9 in MCF-7 cells, suggesting a role of caspase-8 in mediating apoptosis in HEK293 cells, while caspase-9 may promote apoptotic cell death in MCF-7 cells. Taken together, these data indicate the involvement of caspase-dependent apoptosis and presume that compound **29** dependently on the cell line may evoke apoptosis preferentially by intrinsic or by extrinsic pathway.

In summary, despite the precise targets of compound **29** remaining elusive, overall data clearly demonstrated that the substance raised ROS generation, which in turn, resulted in cell-cycle dependent (MCF-7) or cell-cycle-independent (HEK293 cells) apoptosis. Annexin V/SYTOX staining, evaluation of mitochondrial membrane potential, and initiator caspases activity prove the apoptosis induction and suggested that compound **29** caused the apoptosis in MCF-7 cells mainly via the intrinsic pathway by depolarising MMP and subsequent activation of downstream caspase-9, although the contribution of the cell death receptor-mediated pathway is not excluded as well. Withal, in compound **29**-treated HEK293 cells, the accumulation of sub-G_1_ apoptotic cells occurred without disturbances of the cell cycle and was accompanied by a decrease of MMP and substantial activation of caspase-8, thus, proposing the involvement of the extrinsic apoptosis pathway. However, the extrinsic pathway can converge on the intrinsic pathway through the caspase-8-mediated direct cleavage of BID protein, which is responsible for mitochondrial cytochrome c release followed by the subsequent triggering of the mitochondrial-centered control mechanism [51]. According to literature data, the most relevant mechanisms of the anticancer activity of triterpenoids involved cell cycle arrest, apoptosis, and autophagy triggered by the effect of these secondary metabolites on the mitogen-activated different signaling pathways [3,4,5]. Mechanistically, ursolic acid mediates its antitumor potential through inhibition of NF-κB activation induced by carcinogenic agents with targets at cyclooxygenase 2, matrix metallo- proteinase 9, and cyclin D11 [52]. It also inhibits tumor growth through other promising mechanisms involving angiogenesis and metastasis [53]. In a side-by-side comparison, C-2-benzylidene-3-oxo-ursolic acid derivative, contained indole fragments, inhibited glioma cell growth, induced apoptosis, and arrested the cell cycle through metabolic pathway down-regulation [29].

### 2.3. Structure-Activity Analysis with Network-like Similarity Graphs

In order to systematically comprehend the biological data obtained and guide future drug design efforts, we performed a structure-activity relationship analysis with network-like similarity graphs [54] using the Rubberband Forcefield approach implemented in DataWarrior software [55]. In essence, it maps the studied molecules onto 2D-chemical space as graph nodes so that similar structures are located closely. Similarity relationships between them are shown as graph edges. In addition, the so-called Structure-Activity Landscape Index (SALI) is calculated for all pairs of similar molecules. The SALI value is proportional to activity change and inversely proportional to the dissimilarity between molecules. SALI defines the size of nodes and allows easy identification of activity cliffs when an abrupt change in activity is achieved with small structural modification. As an activity measure, mean growth percentages from NCI test panel cell lines were used. Chemical structures were represented with circular fingerprint SkelSpheres for fine-grained chemical similarity and with Flexophore to assess 3D-pharmacophore similarity.

**Figure 7:**
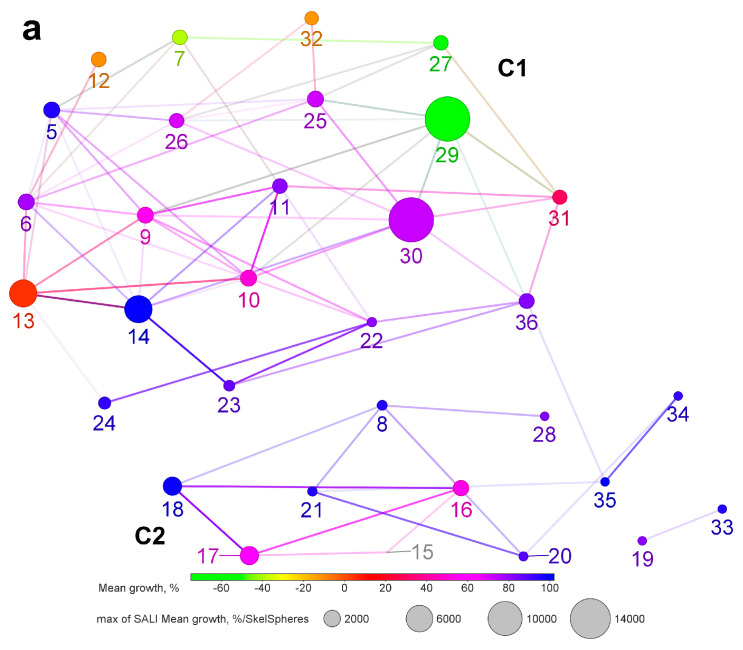

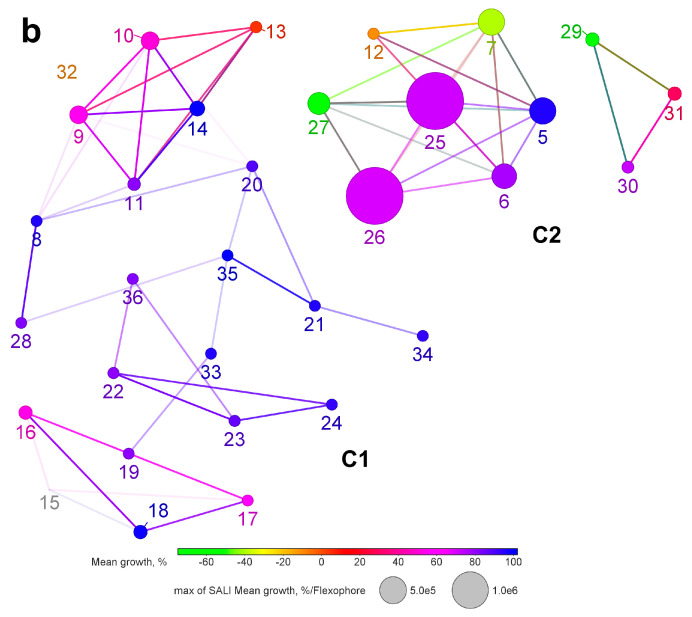
Network-like similarity graphs generated with SkelSpheres (**a**) and Flexophore (**b**) descriptors. Compounds are displayed as nodes and edges indicate molecular similarity relationships. Nodes are colored according to compound cytotoxicity using a continuous color spectrum from green (highest potency in the data set) over red to blue (lowest potency). Nodes are scaled in size according to their contribution to local SAR discontinuity.

The obtained network-like similarity graphs are shown in Figure 7. According to the SkelSpheres descriptor, which takes into account the mutual arrangement of the atoms and stereochemistry, the compounds were distributed rather uniformly. Two clusters can be recognized. C1 contains C3- benzylidene and indole-fused derivatives. C2 is comprised of C3-oxo and C3-nicotinoyloxy, lactame, and azepane derivatives, with all of them showing low cytotoxicity and flat SAR. The most active compounds belong to C1, but are separated with a large “chemical distance” and inactive analogs (except for **7** and **27**, which differ only in C29 methyl position and have similar potency). Compound **13** demonstrates that the C28-morpholinyl fragment is clearly beneficial over piperazinyl and N-methylpiperazinyl (**9**, **10**, **11**) or free carboxyl (**5**, **6**), and C2-4-nicotinoylidene is superior to 3-nicotinoylidene (**5**, **9**), or furfurylidene (**11**). At the same time, compounds **7** and **27**, comprising C2-furfurylidene and C28-carboxyl, are more active than **13**, and the lead compound **29** features C2-3-nicotinoylidene and C28-N-methylpiperazinyl.

Hence, the 2D SkelSpheres fingerprint appears to be unable to correctly perceive SAR in the series. Since it is reasonable to assume that compounds of similar structure share the mechanism of action, i.e., have the same molecular target, we performed a similarity analysis with the Flexophore descriptor. The latter takes into account molecular flexibility and pharmacophoric features responsible for protein-binding behavior. As Figure 7b shows, this approach produced better results. We can see a big area of continuous SAR with inactive compounds on the right side (C1), while hits populate the upper-right corner and are located more closely to each other (C2). Several activity cliffs can be readily recognized here as well. The largest cliff shows that for ursolic derivatives **25** and **26**, activity is vastly improved upon the introduction of C2-furfurylidene (compounds **7** and **27**). Lead ursolic acid derivative **29** stands out having only two neighbors with close pharmacophore properties, inactive **30** and **31**, where C2-3-nicotinoylidene is substituted with C2-4-nicotinoylidene or C2-furfurylidene, respectively.

Thus, the introduction of different substituents at the C2 position of oleanonic **3** or ursonic **4** acids showed that only the furfurylidene group led to the higher cytotoxic activity, resulting in the potent analogs **7** and **27**, which inhibited a broad spectrum and good antiproliferative activity with a mean growth inhibition percentage between −58.89% and −40.62% in the first stage. As shown in Table 1, compounds **7** and **27** had mean GI_50_ (concentration of compound that inhibits cell growth by 50%) values of 3.39 μM and 4.57 μM, respectively, which showed that ursane core is 1.34-fold more active than oleanane. The reduction of 3-oxo-group of the inactive C2-3-pyridinylidene acids **5** and **25** resulted in the more active analogs **12** and **32** with a mean GI_50_ of 8.13 μM and of 15.14 μM.

Modification of inactive N-methylpiperazinyl-amides **8** and **28** at C2-positions had a different influence which depends on the triterpene core type. Thus, among C2-3-pyridinylidene-N-methylpiperazinyl amides, ursane derivative **29** showed higher activity with a mean growth inhibition percentage of −74.90%, while moderate activity was observed for oleanolic acid analogue **9** with 55.08% of mean growth. Similarly, among the C2-furfurylidene-amides **11** and **31**, the activity was observed for ursane type analog **31** with selectivity against renal cancer UO-31 (−97.52%).

On the other hand, among the C2-4-pyridinylidene amides **10** and **30**, only oleanane type derivative **10** showed inhibitory activity against the leukemia HL-60(TB) cell line in the the one-dose 60-cell assay. The replacement of the N-methyl-piperazinyl fragment (compound **10**) by a morpholinyl moiety (compound **13**) led to superior results with a mean GI_50_ of 1.55 μM, and LOX IMVI (melanoma) was the most sensitive cancer cell line. The piperazinyl amide **14** did not show any antiproliferative activity. On the contrary, the indole-derivatives **22**–**24** and **36** showed weak activity against most of the investigated cell lines, as well as seco- **19**, **33**, lactame **20**, **34**, and azepane-derivatives **21**, **35**.

We can conclude that compounds presented here are characterized with non-additive SAR, i.e., substituents do not act independently, and the final effect on cytotoxicity could not be ruled out from individual structure modifications. Analysis of clusters represented by active molecules suggests that substituents at C2 and C28 have a strong influence on cytotoxicity, but their direction depends on the core triterpene structure. Therefore, pharmacophore modeling should be used to guide further optimization of lead compounds. The mapping of novel virtual structures onto a network-like similarity graph developed in this work may provide a venue to overcome this issue.

### 2.4. CellMiner and Gene Enrichment Analysis

To govern the mechanism of action studies for lead compounds, we have analyzed their cytotoxic activity spectrum using the CellMiner pattern comparison tool [56]. The premise of this approach is in the assumption that drugs with a similar cytotoxic activity profile share a molecular target or mechanism of action. Hence, pGI_50_ values obtained for NCI-60 cell lines for compounds **4**–**6**, **10**, **11**, and **21** were used as seeds to identify significant (*p* < 0.05) correlations with compounds that were previously tested at NCI. Results were filtered to exclude weak correlations (Pearson’s coefficient *r* < 0.5) and substances with unknown mechanisms of action (Table 5). We also identified correlations between the 60-cell line gene expression patterns and cancer cell lines sensitivity profiles using CellMiner and Gene Ontology (GO) term enrichment analysis to further elucidate plausible molecular effectors and targets of compounds’ action (Table S4). By analyzing the NCI-60 cell lines for a correlation between their transcriptome and their sensitivity to the cytotoxic effects, we found genes that were significantly correlated (*p* < 0.05) with their in vitro antiproliferative activity.

No analogs with known mechanisms of action have been found for compound **7**. Gene enrichment analysis revealed several interesting traits. Significant correlations were found for genes involved in interleukin-4 receptor binding, lipid-transporting, and sterol-transporting ATPase activity (ABCG1), as well as other genes, mediating immune cell activation (CD2, CD48, CR2, CCR9) and cholesterol metabolism.

The activity distribution of compounds **12**, **13**, and **29** against NCI-60 cell lines correlates the most with several benzamide type HDAC inhibitors. HDAC inhibitors mostly act via epigenetic regulation and are known to cause cell cycle arrest and apoptosis, reduce angiogenesis, and modulate immune response [57]. Similarly, compound **12** appears to act via CD38, CD52, CCKBR, P2RY1, CXCR4, and RXFP3 genes that are involved in the elevation of cytosolic Ca^2+^ concentration (which ultimately leads to apoptosis) and activation of lymphocytes and leukocytes.

Other correlating drugs for compound **13** are alkylating agents and PARP1 inhibitor olaparib, which damage and prevent the reparation of DNA, respectively. Curiously, gene enrichment analysis suggests that **13** regulates adenylate cyclase activity by the G-protein signaling pathway of calcitonin receptor, which probably explains the similarity of cytotoxic specificity between **13** and tyrosine kinase inhibitors vismodegib and LDK-378.

For compound **27**, triapine and camptothecin derivatives were found to share a similar activity profile. Triapine (3-aminopyridine-2-carboxaldehyde thiosemicarbazone) is a small molecule inhibitor of ribonucleotide reductase, reducing the availability of deoxyribonucleotides required for DNA synthesis and currently being investigated in clinical trials [58]. Camptothecin derivative NSC 681634 targets topoisomerase I to induce DNA strand breaks [59]. Gene enrichment analysis failed to reveal any additional insights.

The most promising compound **29** appears to act via a wide range of genes, as enrichment analysis shows. Notably, there is a correlation with genes involved in histone deacetylase and chromatin remodeling complexes (TOP2B, RBBP4, HDAC1, HDAC6, PKN1), which corresponds to activity pattern similarity of **29** and HDAC inhibitors. Interestingly, HDAC inhibitor trichostatin A induces G_0_/G_1_ phase arrest in hepatoma cells HepG2 and Huh-7, and **29** exhibits a similar action in A549 cells [60]. Another important aspect of compound **29**’s action is the involvement of genes regulating mitochondrial (NDUFA5, NDUFB11, ATP5A1, NDUFAB1) and ribosomal functions (RPS25, RPS9, RPSA, RPS10, RPS12), which are essential for the survival of cancer cells. These are in agreement with the experimental data on mitochondrial dysfunction, caused by compound **29**. This might also serve as an explanation of the cell cycle arrest at the S phase which is energy consuming and critically depends on ribosomal protein synthesis. Additionally, genes participating in pyruvate dehydrogenase activity are also affected (PDHA1, PDHB, DLAT), which suggests that compound **29** might also inhibit anaerobic glycolysis. This multifaceted nature might explain the high cytotoxic activity of **29**.

The activity profile of compound **32** significantly correlates with several tyrosine kinase inhibitors and alkylating agents LDK-378 and estramustine. Gene enrichment consistently shows that the cytotoxic activity of **32** is mediated via the interleukin-2 signaling (IL2RA, IL2RB), specifically MAPK/ERK pathway (IL26, NOD1, NOD2, TNF genes). Recombinant IL-2 is approved in the USA and several European countries for the treatment of malignant melanoma and renal cancer. Furthermore, multiple genes involved in cytokine secretion and lymphocyte-mediated immune reactions are enriched, reflecting the greater activity of **32** towards lymphoid cancer cell lines.

### 2.5. Computational ADMET Profiling of Compound **29**

Preliminary assessment of the pharmacokinetic and toxicological properties of lead compound **29** was performed with several predicting services utilizing different models. The choice of services was based on applicability criterion since not all of them are trained on compounds of triterpene nature. Consensus results are shown in Table 5. Computational results show that compound **29** is highly lipophilic and, consequently, predicted to have low water solubility. However, no solubility issues were noted in biological experiments. Due to high logP, the compound is likely to have high intestinal absorption and good cellular permeability due to P-glycoprotein inhibition.

The predicted volume of distribution (VDss) is low, which is typical for lipophilic compounds bound to tissue and cellular components (e.g., protein, lipid) and might favor antitumor activity. Compound **29** is predicted to be degraded by CYP3A4, which is known to oxidize steroids and other large molecules. There is also a possibility of CYP3A4 inhibition, which could be circumvented by decreasing the lipophilicity, adding steric hindrance to the heterocycle para to the nitrogen, or adding an electronic substitution (e.g., halogen) that reduces the pKa of the nitrogen [61]. Furthermore, bearing planar amide moiety, compound **29** could be a substrate for CYP1A2. Low predicted clearance values in conjunction with a short half-life (T_1/2_) might reflect the possibility that the compound is prone to rapid metabolic degradation. Thus, structural modifications might be required to improve its metabolic stability and achieve a more favorable pharmacokinetic profile.

Compound **29** is predicted to be non-mutagenic but might be a hERG inhibitor, which again might be addressed by the introduction of polar substituents [62]. According to predicted moderate oral acute toxicity, the compound can be attributed to Category 3 according to GHS classification.

Overall, computational ADMET profiling renders compound **29** as suitable for future in vivo testing and indicates that possible drawbacks, such as rapid metabolic degradation and cardiotoxicity, might be addressed by a logP decrease (Table 6).

## 3. Materials and Methods

### 3.1. Experimental Part

#### 3.1.1. General

The spectra were recorded at the Center for the Collective Use ‘Chemistry’ of the UIC UFRC RAS and RCCU “Agidel” of the UFRC RAS. ^1^H and ^13^C NMR spectra (δ, ppm, Hz) were recorded on a “Bruker *Avance*-III” 500 and 125.5 MHz, respectively. (Bruker, Billerica, MA, USA), in CDCl_3_, internal standard—tetramethylsilane. Mass spectra were obtained on a liquid chromatograph–mass spectrometer LCMS-2010 EV (Shimadzu, Kyoto, Japan). Melting points were detected on a microtable «Rapido PHMK05» (Nagema, Dresden, Germany). Optical rotations were measured on a polarimeter “Perkin-Elmer 241 MC” (PerkinElmer, Waltham MA, USA) in a tube length of 1 dm. Elemental analysis was performed on a Euro EA-3000 CHNS analyzer (Eurovector, Milan, Italy), the main standard is acetanilide. Thin-layer chromatography analyses were performed on Sorbfil plates (Sorbpolimer, Krasnodar, Russian Federation), using the solvent system chloroform–ethyl acetate, 40:1. Substances were detected by a 10% solution of sulfuric acid solution with subsequent heating at 100–120 °C for 2–3 min. All chemicals were of reagent grade (Sigma-Aldrich, St. Louis, MO, USA). The solvents were purified and 3-oxo-, 3-oximino-triterpenoids were synthesized according to the standard methods. Compounds **5**, **6**, **25**, and **26** [65], **8** and **28** [66], 2,3-indole derivatives of compounds 3, 4 [67], and 15 [68] were obtained according to the methods described previously.

#### 3.1.2. Synthesis of Compounds **7** and **27**

Furfural (0.11 mL, 1.3 mmol) and 40% KOH in ethanol (2.5 mL) were added to a solution of compound **3** or compound **4** (0.45 g, 1 mmol) in ethanol (5 mL) under stirring and cooling (from −5 to 10 °C). The mixture was stirred for 24 h at room temperature, pH was adjusted to neutral values with 5% HCl solution, and the mixture was poured into cold water (50 mL). The residue was filtered, washed with water, and dried, then purified by column chromatography on **Al_2_O_3_** using petroleum ether–CHCl_3_ (1:1 to 1:3) as eluent.

2-[3-(2E-furyl)-prop-2-en-1-one]-3-oxo-olean-12-en-28-oic acid **7**. Yield 0.48 g (90%). *R_f_* 0.25; mp 147–149 °C. [α]*_D_*^20^ + 2 (*c* 0.5, CHCl_3_). ^1^H NMR (δ, ppm): 0.81, 0.90, 1.05, 1.12, 1.18, 1.20, 1.22 (7 s, 21H, 7CH**_3_**), 1.23–2.09 (m, 20H, CH, CH**_2_**), 2.27 and 3.16 (both d, 2H, J 3.7 Hz, H-1), 5.40 (s, 1H, H-12), 6.50 (m, 1H), 6.58 (d, 1H, J 3.6 Hz), 7.29 (br. s, 1H), 7.54 (d, 1H, H-1′, J 1.7 Hz). ^13^C NMR (δ, ppm): 15.7, 16.6, 20.3, 22.5, 23.0, 23.6, 23.7, 25.7, 25.8, 27.7, 29.9, 30.7, 31.8, 32.3, 33.1, 33.8, 35.7, 39.1, 41.1, 41.9, 44.3, 44.8, 45.4, 45.9, 46.7, 52.6, 112.2, 115.5, 122.4 (C-12), 124.2, 130.8 (C-2), 143.7 (C-13), 144.4, 152.6, 184.4 (C-28), 207.3 (C-3). Analysis calculated for C**_36_**H**_48_**O_4_ (***M*** 532.36): C 78.91, H 9.08; found: C 78.90, H 9.07. APCI (*m*/*z*): 533.36 (M + H)^+^ 100%.

2-[3-(2E-furyl)-prop-2-en-1-one]-3-oxo-ursan-12-en-28-oic acid **27**. Yield 0.47 g (89%). *R_f_* 0.20; mp 156–157 °C. [α]*_D_*^20^ + 54.5 (*c* 0.5, CHCl_3_). ^1^H NMR (δ, ppm): 0.80, 0.91, 0.95, 1.00, 1.09, 1.11, 1.21 (7 s, 21H, 7CH**_3_**), 1.23–2.09 (m, 20H, CH, CH**_2_**), 2.22 and 3.18 (both d, 2H, J 3.8 Hz, H-1), 5.32 (s, 1H, H-12), 6.50 (m, 1H), 6.61 (d, 1H, J 3.6 Hz), 7.29 (br. s, 1H), 7.55 (d, 1H, H-1′, J 1.7 Hz). ^13^C NMR (δ, ppm): 15.5, 16.7, 17.5, 17.7, 18.5, 20.3, 22.3, 22.7, 23.6, 24.5, 28.2, 29.7, 29.9, 32.2, 34.2, 36.3, 38.7, 39.6, 42.4, 44.2, 45.2, 45.3, 45.3, 46.0, 53.1, 52.5, 112.2, 115.5, 122.4, 125.1 (C-12), 130.8 (C-2), 135.9 (C-13), 144.1, 152.4, 182.3 (C-28), 207.4 (C-3). Analysis calculated for C**_36_**H**_48_**O_4_ (***M*** 532.36): C 78.81, H 9.08; found: C 78.79, H 9.09. APCI (*m*/*z*): 533.36 (M + H)^+^ 100%.

#### 3.1.3. Synthesis of Compounds **9**–**11**, **13**, **14**, **16**–**18**, **22**–**24**, **29**–**31**, **36**

To a solution of compound **5**–**7**, 15, 2,3-indolo-olenolic acid [64] or **25**–**27** (1 mmol) in CH_2_Cl_2_ (20 mL) (COCl)_2_ (3 mmol; 0.26 mL) was added and stirred at room temperature for 2 h. The mixture was concentrated to dryness under reduced pressure and the resulting acid chloride was dissolved in CH_2_Cl_2_ (10 mL), 3 drops of Et_3_N and 1.5 mmol of the corresponding amine were added: (a) *N*-methylpiperazine (for synthesis of compounds **9**–**11**, **16**, **22**, **29**–**31**, **36**); (b) morpholine (for synthesis of compounds **13**, **17**, and **24**); (c) piperazine (for synthesis of compounds **14**, **18**, and **23**). After completion of the reactions (TLC control) the organic layers were treated with 5% HCl (3 × 50 mL) until neutral pH, dried over CaCl_2_, and evaporated under reduced pressure. The residue was purified by column chromatography on Al_2_O_3_ using petroleum ether–CHCl_3_ (10:1 to 0:10) as eluent.

N-2-[3-(2E-Pyridinyl)-prop-2-en-1-one]-3-oxoolean-12-en-28-oyl)-methylpiperazine **9**. Yield 0.54 g (87%). *R_f_* 0.23; mp 177–178 °C. [α]*_D_*^20^ + 13.5 (*c* 0.1, CHCl_3_). ^1^H NMR (δ, ppm): 0.80, 0.90, 1.01, 1.11, 1.19, 1.35, 1.40 (7 s, 21H, 7CH_3_), 1.39–2.19 (m, 19H, CH and CH_2_), 2.30 (s, 3H, NCH_3_), 2.31–2.41 (m, 4H, 2CH_2_), 2.89–2.98 (m, 2H, H-1), 3.41–3.71 (m, 4H, 2CH_2_), 5.21 (s, 1H, H-12), 7.35 (m, 1H, Ar-CH), 7.41 (s, 1H, vinilic H), 7.73 (d, 1H, J 8 Hz), 8.52 (d, 1H, J 4.0 Hz, Ar-CH), 8.73 (s, 1H, Ar-CH). ^13^C NMR (δ, ppm): 15.5, 16.7, 17.5, 17.7, 18.5, 20.3, 21.3, 22.3, 22.7, 23.6, 24.5, 28.2, 29.7, 29.9, 30.6, 32.2, 34.2, 36.3, 38.7, 39.3, 39.6, 42.4, 44.2, 45.2, 45.3, 45.3, 46.0, 53.1, 55.1, 55.1, 123.4, 124.8 (C-12), 128.3, 131.8, 133.6, 137.1 (C-2), 144.4 (C-13), 149.1, 151.0, 175.1 (C-28), 207.4 (C-3). Analysis calculated for C**_41_**H**_59_**N_3_O_2_ (***M*** 625.93): C 78.67, H 9.50, N 6.71; found: C 78.54, H 9.36, N 6.69. APCI (*m*/*z*): 626.73 (M + H)^+^ 100%.

N-2-[4-(2E-pyridinyl)-prop-2-en-1-one]-3-oxoolean-12-en-28-oyl)-methylpiperazine **10**. Yield 0.56 g (90%). *R_f_* 0.25; mp 156–157 °C. [α]*_D_*^20^ + 7 (*c* 0.5, CHCl_3_). ^1^H NMR (δ, ppm): 0.80, 0.90, 1.01, 1.11, 1.19, 1.34, 1.41 (7 s, 21H, 7CH_3_), 1.39–2.19 (m, 19H, CH and CH_2_), 2.31 (s, 3H, NCH_3_), 2.31–2.42 (m, 4H, 2CH_2_), 2.90–2.98 (m, 2H, H-1), 3.41–3.71 (m, 4H, 2CH_2_), 5.23 (s, 1H, H-12), 6.85 (s, 1H, H-1′), 7.02 (d, 2H, J 5.84 Hz, Ar-CH), 8.36 (d, 2H, J 5.68 Hz, Ar-CH). ^13^C NMR (δ, ppm): 15.5, 16.7, 17.5, 17.7, 18.5, 20.3, 21.3, 22.3, 22.7, 23.6, 24.5, 28.2, 29.7, 29.9, 30.6, 31.5, 32.2, 34.2, 36.3, 38.7, 39.3, 39.6, 42.4, 44.2, 45.2, 45.3, 45.3, 46.0, 53.1, 55.1, 55.1, 122.0, 122.0, 125.1 (C-12), 134.0 (C-2), 138.0, 143.8 (C-13), 149.4, 149.4, 175.8 (C-28), 207.4 (C-3). Analysis calculated for C**_41_**H**_59_**N_3_O_2_ (*M* 625.93): C 78.67, H 9.50, N 6.71; found: C 78.54, H 9.36, N 6.69. APCI (*m*/*z*): 626.73 (M + H)^+^ 100%.

N-2-[2-(2E-furyl)-prop-2-en-1-one]-3-oxo-olean-12-en-28-oyl)-methylpiperazine **11**. Yield 0.58 g (95%). *R_f_* 0.35; mp 160–161 °C. [α]*_D_*^20^ + 24° (*c* 0.5, CHCl_3_). ^1^H NMR (δ, ppm): 0.79, 0.87, 0.91, 1.09, 1.10, 1.29, 1.41 (7 s, 21H, 7CH_3_), 1.39–2.19 (m, 19H, CH and CH_2_), 2.31 (s, 3H, NCH_3_), 2.31–2.51 (m, 4H, 2CH_2_), 3.02–3.11 (m, 2H, H-1), 3.61–3.79 (m, 4H, 2CH_2_), 5.31 (s, 1H, H-12), 6.45–6.48 (m, 1H), 6.60 (d, 1H, J 3.4 Hz), 7.28–7.30 (m, 1H), 7.56 (s, 1H, H-1′). ^13^C NMR (δ, ppm): 15.66, 16.44, 20.42, 22.46, 22.50, 22.67, 23.63, 24.04, 25.73, 27.87, 28.60, 29.90, 30.39, 31.98, 33.05, 33.99, 34.00, 35.73, 38.95, 42.11, 43.78, 44.36, 44.80, 45.51, 45.68, 46.41, 47.48, 52.70, 54.99, 54.99, 112.20, 115.43, 121.35, 124.12 (C-12), 130.92 (C-2), 144.36 (C-13), 144.91, 152.57, 174.97 (C-28), 207.33 (C-3). Analysis calculated for C**_40_**H**_58_**N_2_O_3_ (***M*** 614.92): C 78.13, H 9.51, N 4.56; found: C 78.54, H 9.72, N 4.63. APCI (*m*/*z*): 615.71 (M + H)^+^ 100%.

N-2-[4-(2E-pyridinyl)-prop-2-en-1-one]-3-oxo-olean-12-en-28-oyl)-morpholine **13**. Yield 0.55 g (89%). *R_f_* 0.30; mp 187–188 °C. [α]*_D_*^20^ + 34 (*c* 0.1, CHCl_3_). ^1^H NMR (δ, ppm): 0.79, 0.81, 0.85, 0.91, 1.09, 1.18, 1.21 (7 s, 21H, 7CH_3_), 1.39–3.12 (m, 19H, CH and CH_2_), 1.52–1.71 (m, 4H, 2CH_2_), 2.90–2.98 (m, 2H, H-1), 3.51–3.71 (m, 4H, 2CH_2_), 5.23 (s, 1H, H-12), 7.35 (d, 2H, J 5.6 Hz), 7.71 (s, 1H, vinilic H), 8.62 (d, 2H, J 7.2 Hz). ^13^C NMR (δ, ppm): 15.3, 16.5, 20.3, 22.6, 22.7, 23.2, 23.6, 24.0, 25.8, 27.8, 29.5, 29.6, 29.7, 29.8, 30.4, 31.9, 32.0, 33.0, 34.0, 36.4, 39.0, 42.1, 42.1, 43.7, 44.1, 45.2, 45.4, 45.5, 46.1, 46.3, 47.5, 53.0, 66.9, 66.9, 123 (C-12), 144.9 (C-13), 148.3 (C-1′), 150.7, 175.1 (C-28), 207.4 (C-3). Analysis calculated for C**_40_**H**_56_**N_2_O_3_ (*M* 612.88): C 78.39, H 9.21, N 4.57; found: C 78.38, H 9.19, N 4.56. APCI (*m*/*z*): 613.87 (M + H)^+^ 100%.

N-2-[4-(2E-pyridinyl)-prop-2-en-1-one]-3-oxo-olean-12-en-28-oyl)-piperazine **14**. Yield 0.46 g (75%). *R_f_* 0.15; mp 148–149 °C. [α]*_D_*^20^ + 14 (*c* 0.5, CHCl_3_). ^1^H NMR (δ, ppm): 0.78, 0.81, 0.85, 0.90, 1.09, 1.18, 1.21 (7 s, 21H, 7CH_3_), 1.22–2.15 (m, 19H, CH and CH_2_), 3.10–3.21 (m, 4H, 2CH_2_), 2.90–2.98 (m, 2H, H-1), 3.55–3.69 (m, 4H, 2CH_2_), 4.21 (br. s, 1H, NH), 5.23 (s, 1H, H-12), 7.35 (d, 2H, J 5.6 Hz), 7.70 (s, 1H, vinilic H), 8.63 (d, 2H, J 7.2 Hz). ^13^C NMR (δ, ppm): 15.3, 16.5, 20.3, 22.7, 22.8, 23.1, 23.6, 24.1, 25.8, 27.8, 29.5, 29.6, 29.7, 29.9, 30.5, 31.9, 32.0, 33.0, 34.0, 36.4, 39.0, 42.1, 42.1, 43.8, 44.1, 45.2, 45.4, 45.5, 46.1, 46.3, 47.5, 53.0, 58.5, 58.8, 123 (C-12), 144.9 (C-13), 148.3 (C-1′), 150.7, 175.6 (C-28), 207.5 (C-3). Analysis calculated for C_40_H_57_N_3_O_2_ (*M* 611.90): C 78.51, H 9.39, N 6.87; found: C 78.50, H 9.38, N 6.86. APCI (*m*/*z*): 612.87 (M + H)^+^ 100%.

N-(3β-Nicotinoyloxy-olean-12-en-28-oyl)-methylpiperazine **16**. Yield 0.50 g (78%). *R_f_* 0.35; mp 201–202 °C. [α]*_D_*^20^ + 7 (*c* 0.1, CHCl_3_). ^1^H NMR (δ, ppm): 0.76, 0.80, 0.84, 0.90, 1.09, 1.13, 1.17 (7 s, 21H, 7CH_3_), 1.31–2.20 (m, 24H, CH and CH_2_), 2.15–2.38 (m, 4H, 2CH_2_), 2.31 (s, 3H, NCH_3_), 3.61–3.78 (m, 4H, 2CH_2_), 5.31 (s, 1H, H-12), 7.42 (1 H, dd, J 4.9 Hz, 4.7, H_arom_), 8.23 (1 H, ddd, J 5.1 Hz, 2.1 Hz, 1.8 Hz, H_arom_), 8.76 (1 H, t, J 4.6 Hz, H_arom_), 9.22 (1 H, dd, J 1.9 Hz, 7.0 Hz, H_arom_). ^13^C NMR (125.5 MHz, CDCl_3_): 15.7, 16.9, 17.5, 19.3, 21.3, 23.3, 23.4, 23.7, 23.8, 28.3, 30.6, 31.0, 32.6, 34.0, 34.3, 35.6, 37.0, 37.4, 37.7, 38.8, 39.6, 41.5, 42.3, 45.3, 45.9, 46.1, 46.4, 48.6, 53.4, 55.2, 55.2, 83.0 (C-3), 121.4, 123.9 (C-12), 127.4, 138.5, 144.7 (C-13), 149.5, 151.8, 164.3, 176.0 (C-28). Analysis calculated for C_41_H_61_N_3_O_3_ (*M* 643.94): C 76.47, H 9.55, N 6.53; found: C 76.43, H 9.54, N 6.52. APCI (*m*/*z*): 644.92 (M + H)^+^ 100%.

N-(3β-Nicotinoyloxy-olean-12-en-28-oyl)-morpholine **17**. Yield 0.54 g (85%). *R_f_* 0.30; mp 197–198 °C. [α]*_D_*^20^ + 18 (*c* 0.5, CHCl_3_). ^1^H NMR (δ, ppm): 0.75, 0.81, 0.89, 0.91, 0.99, 1.02, 1.12 (7 s, 21H, 7CH_3_), 1.21–2.15 (m, 23H, CH and CH_2_), 3.01–3.11 (m, 4H, 2CH_2_), 3.52–3.69 (m, 4H, 2CH_2_), 4.75 (t, 1H, J = 8.4, H-3), 5.23 (s, 1H, H-12), 7.41 (1 H, dd, J 4.9 Hz, 4.7 Hz, Harom), 8.24 (1 H, ddd, J 5.1 Hz, 2.1 Hz, 1.8 Hz, Harom), 8.75 (1 H, t, J 4.6 **Hz**, Harom), 9.21 (1 H, dd, J 1.9 Hz, 7.0 Hz, Harom). ^13^C NMR (δ, ppm): 15.4, 16.7, 17.0, 18.2, 22.7, 23.4, 23.6, 24.1, 26.0, 27.9, 28.2, 30.0, 30.4, 32.7, 33.1, 34.0, 37.0, 37.8, 38.0, 38.1, 39.1, 41.9, 43.5, 46.0, 46.3, 47.4, 47.7, 55.4, 67.0, 67.0, 83.0 (C-3), 121.4, 123.9 (C-12), 127.4, 138.5, 144.7 (C-13), 149.5, 151.8, 164.3, 175.2 (C-28). Analysis calculated for C**_40_**H**_58_**N_2_O_4_ (*M* 630.90): C 76.15, H 9.27, N 4.44; found: C 76.13, H 9.26, N 4.44. APCI (*m*/*z*): 631.89 (M + H)^+^ 100%.

N-(3β-Nicotinoyloxy-olean-12-en-28-oyl)-piperazine **18**. Yield 0.44 g (70%). *R_f_* 0.30; mp 131–132 °C. [α]*_D_*^20^ + 17 (*c* 0.5, CHCl_3_). ^1^H NMR (δ, ppm): 0.76, 0.82, 0.89, 0.92, 0.99, 1.03, 1.15 (7 s, 21H, 7CH_3_), 1.23–2.16 (m, 23H, CH and CH_2_), 3.10–3.21 (m, 4H, 2CH_2_), 3.55–3.69 (m, 4H, 2CH_2_), 4.21 (br. s, 1H, NH), 4.76 (t, 1H, J 8.4 Hz, H-3), 5.24 (s, 1H, H-12), 7.40 (1 H, dd, J 4.9 Hz, 4.7 Hz, H_arom_), 8.25 (1 H, ddd, J 5.1 Hz, 2.1 Hz, 1.8 Hz, H_arom_), 8.76 (1 H, t, J 4.6 Hz, H_arom_), 9.22 (1 H, dd, J 1.9 Hz, 7.0 Hz, H_arom_). ^13^C NMR (125.5 MHz, CDCl_3_): 15.4, 16.7, 17.1, 18.2, 22.8, 23.4, 23.7, 24.1, 26.1, 27.9, 28.3, 30.1, 30.4, 31.0, 32.8, 33.1, 34.1, 37.0, 37.8, 38.0, 38.2, 39.1, 41.9, 43.6, 46.0, 46.3, 47.4, 47.8, 58.6, 58.6, 83.0 (C-3), 121.4, 124.0 (C-12), 127.5, 138.5, 144.5 (C-13), 149.5, 151.8, 164.3, 176.5 (C-28). Analysis calculated for C**_40_**H**_59_**N_3_O_3_ (***M*** **629.92**): C 76.27, H 9.44, N 6.67; found: C 76.26, H 9.42, N 6.65. APCI (*m*/*z*): 630.91 (M + H)^+^ 100%.

N-([3,2b]-Indolo-olean-12-en-28-oyl)-N-methylpiperazine **22**. Yield 0.54 g (90%). *R_f_* 0.35; mp 174–175 °C. [α]*_D_*^20^ + 63 (*c* 0.1, CHCl_3_). ^1^H NMR (500 MHz, CDCl_3_): 0.83, 0.91, 0.99, 1.01, 1.10, 1.15, 1.32 (7 s, 21H, 7CH_3_), 1.31–2.20 (m, 19H, CH and CH_2_), 2.15–2.38 (m, 4H, 2CH_2_), 2.31 (s, 3H, NCH_3_), 2.71–2.95 (m, 2H, H-1), 3.61–3.78 (m, 4H, 2CH_2_), 5.31 (s, 1H, H-12), 7.07–7.44 (m, 4H_arom_, 4CH), 8.10 (br. s, 1H, NH). ^13^C NMR (125.5 MHz, CDCl_3_): 15.7, 16.9, 17.5, 19.3, 21.3, 23.3, 23.4, 23.7, 23.8, 28.3, 30.6, 31.0, 32.6, 34.0, 34.3, 35.6, 37.0, 37.4, 38.8, 39.6, 41.5, 42.3, 45.3, 45.9, 46.1, 46.4, 48.6, 53.4, 55.2, 55.2, 106.8, 110.4 (C-2), 118.0, 118.8, 120.8, 121.9 (C-12), 128.3, 136.2, 141.0 (C-3), 144.6 (C-13), 175.1 (C-28). Analysis calculated for C**_41_**H**_59_**N_3_O (*M* 609.47): C 80.74, H 9.75, N 6.89; found: C 80.72, H 9.73, N 6.85. APCI (*m*/*z*): 610.47 (M + H)^+^ 100%.

N-([3,2b]-Indolo-olean-12-en-28-oyl)-piperazine **23**. Yield 0.51 g (85%). *R_f_* 0.20; mp 205–206 °C. [α]*_D_*^20^ + 34 (*c* 0.5, CHCl_3_). ^1^H NMR (500 MHz, CDCl_3_): 0.84, 0.91, 0.99, 1.02, 1.11, 1.15, 1.31 (7 s, 21H, 7CH_3_), 1.31–2.20 (m, 19H, CH and CH_2_), 3.10–3.21 (m, 4H, 2CH_2_), 2.80–2.95 (m, 2H, H-1), 3.55–3.69 (m, 4H, 2CH_2_), 4.21 (br. s, 1H, NH), 5.31 (s, 1H, H-12), 7.07–7.44 (m, 4H_arom_, 4CH), 8.10 (br. s, 1H, NH). ^13^C NMR (125.5 MHz, CDCl_3_): 15.7, 16.9, 17.5, 19.3, 21.3, 23.3, 23.4, 23.7, 23.8, 28.3, 30.6, 31.0, 32.6, 34.0, 34.3, 35.6, 37.0, 37.4, 38.8, 39.6, 41.5, 42.3, 45.3, 45.9, 46.4, 48.6, 53.4, 55.2, 55.2, 106.8, 110.4 (C-2), 118.0, 118.8, 120.8, 121.9 (C-12), 128.3, 136.2, 141.0 (C-3), 143.9 (C-13), 176.2 (C-28). Analysis calculated for C**_40_**H**_57_**N_3_O (***M*** **595.90**): C 80.62, H 9.64, N 7.05; found: C 80.60, H 9.63, N 7.04. APCI (*m*/*z*): 596.89 (M + H)^+^ 100%.

N-([3,2b]-Indolo-olean-12-en-28-oyl)-morpholine **24**. Yield 0.52 g (87%). *R_f_* 0.25; mp 190–191 °C. [α]*_D_*^20^ + 56 (*c* 0.1, CHCl_3_). ^1^H NMR (500 MHz, CDCl_3_): 0.84, 0.92, 0.99, 1.02, 1.11, 1.15, 1.31 (7 s, 21H, 7CH_3_), 1.31–2.20 (m, 19H, CH and CH_2_), 3.01–3.11 (m, 4H, 2CH_2_), 2.71–2.95 (m, 2H, H-1), 3.52–3.69 (m, 4H, 2CH_2_), 5.26 (s, 1H, H-12), 7.08–7.45 (m, 4H_arom_, 4CH), 8.09 (br. s, 1H, NH). ^13^C NMR (125.5 MHz, CDCl_3_): 15.8, 16.9, 17.6, 19.3, 21.3, 23.3, 23.4, 23.8, 28.3, 30.7, 31.0, 32.6, 34.0, 34.3, 35.6, 37.0, 37.4, 38.8, 39.6, 41.5, 42.3, 45.3, 45.9, 46.1, 46.4, 48.6, 55.4, 67.0, 67.0, 106.8, 111.0 (C-2), 118.0, 118.6, 120.8, 121.9 (C-12), 128.3, 136.2, 141.0 (C-3), 144.6 (C-13), 176.2 (C-28). Analysis calculated for C**_40_**H**_56_**N_2_O_2_ (*M* 596.88): C 80.50, H 9.46, N 4.69; found: C 80.49, H 9.45, N 4.68. APCI (*m*/*z*): 597.87 (M + H)^+^ 100%.

N-2-[3-(2E-pyridinyl)-prop-2-en-1-one]-3-oxoursan-12-en-28-oyl)-methylpiperazine **29**. Yield 0.53 g (85%). *R_f_* 0.32; mp 167–168°C. [α]*_D_*^20^ + 24 (*c* 0.1, CHCl_3_). ^1^H NMR (δ, ppm): 0.80, 0.90, 1.01, 1.11, 1.19, 1.35, 1.40 (7 s, 21H, 7CH_3_), 1.39–2.19 (m, 19H, CH and CH_2_), 2.30 (s, 3H, NCH_3_), 2.31–2.41 (m, 4H, 2CH_2_), 2.89–2.98 (m, 2H, H-1), 3.41–3.71 (m, 4H, 2CH_2_), 5.21 (s, 1H, H-12), 7.35 (m, 1H, Ar-CH), 7.41 (s, 1H, vinilic H), 7.73 (d, 1H, J 8 Hz), 8.52 (d, 1H, J 4.0 Hz, Ar-CH), 8.73 (s, 1H, Ar-CH). ^13^C NMR (δ, ppm): 15.5, 16.7, 17.5, 17.7, 18.5, 20.3, 21.3, 22.3, 22.7, 23.6, 24.5, 28.2, 29.7, 29.9, 30.6, 32.2, 34.2, 36.3, 38.7, 39.3, 39.6, 42.4, 44.2, 45.2, 45.3, 45.3, 46.0, 53.1, 55.1, 55.1, 123.4, 124.8 (C-12), 128.3, 131.8, 133.6, 135.9 (C-13), 137.1 (C-2), 149.1, 151.0, 175.1 (CON), 207.4 (C-3). Analysis calculated for C**_41_**H**_59_**N_3_O_2_ (***M*** **625.93**): C 78.67, H 9.50, N 6.71; found: C 78.54, H 9.36, N 6.69. APCI (*m*/*z*): 626.73 (M + H)^+^ 100%.

N-2-[4-(2E-pyridinyl)-prop-2-en-1-one]-3-oxoursan-12-en-28-oyl)-methylpiperazine **30**. Yield 0.53 g (85%). *R_f_* 0.30; mp 150–151 °C. [α]*_D_*^20^ + 8 (*c* 0.5, CHCl_3_). ^1^H NMR (δ, ppm): 0.80, 0.90, 1.01, 1.11, 1.19, 1.34, 1.41 (7 s, 21H, 7CH_3_), 1.39–2.19 (m, 19H, CH and CH_2_), 2.31 (s, 3H, NCH_3_), 2.31–2.42 (m, 4H, 2CH_2_), 2.90–2.98 (m, 2H, H-1), 3.41–3.71 (m, 4H, 2CH_2_), 5.23 (s, 1H, H-12), 6.85 (s, 1H, vinilic H), 7.02 (d, 2H, J 5.84 Hz, Ar-CH), 8.36 (d, 2H, J 5.68 Hz, Ar-CH). ^13^C NMR (δ, ppm): 15.5, 16.7, 17.5, 17.7, 18.5, 20.3, 21.3, 22.3, 22.7, 23.6, 24.5, 28.2, 29.7, 29.9, 30.6, 31.5, 32.2, 34.2, 36.3, 38.7, 39.3, 39.6, 42.4, 44.2, 45.2, 45.3, 45.3, 46.0, 53.1, 55.1, 55.1, 122.0, 122.0, 125.1 (C-12), 134.0 (C-2), 138.0 (C-13), 143.8, 149.4, 149.4, 175.8 (CON), 207.4 (C-3). Analysis calculated for C_41_H_59_N_3_O_2_ (*M* 625.93): C 78.67, H 9.50, N 6.71; found: C 78.54, H 9.36, N 6.69. APCI (*m*/*z*): 626.73 (M + H)^+^ 100%.

N-2-[3-(2E-furyl)-prop-2-en-1-one]-3-oxoursan-12-en-28-oyl)-methylpiperazine **31**. Yield 0.55 g (90%). *R_f_* 0.28; mp 147–148 °C. [α]*_D_*^20^ + 73 (*c* 0.5, CHCl_3_). ^1^H NMR (δ, ppm): 0.79, 0.87, 0.91, 1.09, 1.15, 1.28, 1.41 (7 s, 21H, 7CH_3_), 1.39–2.19 (m, 19H, CH and CH_2_), 2.31 (s, 3H, NCH_3_), 2.36–2.49 (m, 4H, 2CH_2_), 3.08–3.12 (m, 2H, H-1), 3.61–3.72 (m, 4H, 2CH_2_), 5.29 (s, 1H, H-12), 6.45–6.48 (m, 1H, furf-CH), 6.58 (d, 1H, J 3.4 Hz, furf-CH), 7.27–7.29 (m, 1H, furf-CH), 7.55 (s, 1H, vinilic H). ^13^C NMR (δ, ppm): 15.8, 16.6, 17.5, 20.4, 21.3, 22.5, 22.6, 23.6, 23.6, 28.2, 29.9, 30.5, 31.4, 32.1, 34.3, 34.4, 35.7, 38.7, 39.3, 39.5, 39.6, 42.3, 44.3, 44.8, 44.9, 45.3, 45.7, 52.8, 54.9, 54.9, 112.2, 115.5, 124.2, 125.1 (C-12), 130.9 (C-2), 138.1 (C-13), 144.4, 152.6, 175.3 (C-28), 207.2 (C-3). Analysis calculated for C**_40_**H**_58_**N_2_O_3_ (***M*** **614.92**): C 78.13, H 9.51, N 4.56; found: C 78.10, H 9.48, N 4.50. APCI (*m*/*z*): 615.85 (M + H)^+^ 100%.

N-([3,2b]-Indolo-ursan-12-en-28-oyl)-methylpiperazine **36**. Yield 0.53 g (88%). *R_f_* 0.30; mp 195–196 °C. [α]*_D_*^20^ + 36 (*c* 0.1, CHCl_3_). ^1^H NMR (500 MHz, CDCl_3_): 0.80, 0.91, 0.99, 1.10, 1.20, 1.32, 1.41 (7 s, 21H, 7CH_3_), 1.35–1.86 (m, 19H, CH and CH_2_), 2.15–2.38 (m, 4H, 2CH_2_), 2.35 (s, 3H, NCH_3_), 2.75–2.98 (m, 2H, H-1), 3.61–3.78 (m, 4H, 2CH_2_), 5.35 (s, 1H, H-12), 7.07–7.44 (m, 4H_arom_, 4CH), 7.85 (br. s, 1H, NH). ^13^C NMR (125.5 MHz, CDCl_3_): 15.7, 16.9, 17.5, 19.3, 21.3, 23.3, 23.4, 23.7, 23.8, 28.3, 30.6, 31.0, 32.6, 34.0, 34.3, 35.6, 37.0, 37.4, 38.1, 38.8, 39.6, 41.5, 42.3, 45.3, 45.9, 46.1, 46.4, 48.6, 53.3, 55.1, 55.1, 106.9, 110.3 (C-2), 117.9, 118.9, 120.9, 125.5 (C-12), 128.3, 135.6 (C-13), 136.1, 140.9 (C-3), 175.4 (C-28). Analysis calculated for C_41_H_59_N_3_O (*M* 609.47): C 80.74, H 9.75, N 6.89; found: C 80.73, H 9.74, N 6.85. APCI (*m*/*z*): 610.47 (M + H)^+^ 100%.

#### 3.1.4. Synthesis of Compounds **12** and **32**

Sodium borohydride (25 mg, 0.75 mmol) was added under stirring for 10 min to a solution of compound **5** (0.27 g, 0.5 mmol) or 25 (0.27 g, 0.5 mmol) in *i*-PrOH (15 mL) and kept for 2 h. The mixture was diluted with 10% HCl (30 mL); the residue was filtered, washed with water, dried, and recrystallized from EtOH.

3β-Hydroxy-2-[3-(2E-pyridinyl)-prop-2-en-1-one]-olean-12-en-28-oic acid **12**. Yield 0.44 g (81%). *R_f_* 0.30; mp 180–181 °C. [α]*_D_*^20^ + 35 (*c* 0.1, CHCl_3_). ^1^H NMR (δ, ppm): 0.61, 0.82, 0.89, 1.01, 1.09, 1.10, 1.18 (7 s, 21H, 7CH_3_), 1.15–2.61 (m, 21H, CH and CH_2_), 1.85–2.25 (m, 2H, H-1), 3.86 (s, 1H, H-3), 5.20 (s, 1H, H-12), 6.70 (s, 1H, H-1′), 7.27 (m, 1H, H_arom_), 7.52 (d, 1H, J 8 Hz, H_arom_), 8.44 (m, 2H, H_arom_). ^13^C NMR (δ, ppm): 15.5, 15.7, 16.6, 17.0, 18.4, 21.3, 23.2, 23.8, 24.1, 28.0, 28.6, 30.7, 32.6, 36.6, 38.9, 39.1, 39.7, 40.6, 41.6, 41.9, 44.5, 46.7, 47.7, 53.1, 55.5, 80.8 (C-3), 118.9, 123.3, 123.4 (C-12), 134.8, 137.5 (C-2), 143.2 (C-13), 144.0, 145.2, 148.4, 184.3 (C-28). Analysis calculated for C_36_H_51_NO_3_ (*M* 545.39): C 79.22, H 9.42, N 2.57; found: C 79.21, H 9.41, N 2.56. APCI (*m*/*z*): 546.39 (M + H)^+^ 100%.

3β-Hydroxy-2-[3-(2E-pyridinyl)-prop-2-en-1-one]-ursan-12-en-28-oic acid **32**. Yield 0.45 g (83%). *R_f_* 0.30; mp 180–181 °C. [α]*_D_*^20^ + 35 (*c* 0.1, CHCl_3_). ^1^H NMR (δ, ppm): 0.51, 0.61, 0.75, 0.82, 0.89, 0.96, 1.18, 1.41 (7 s, 21H, 7CH_3_), 1.39–1.95 (m, 21H, CH and CH_2_), 1.98–2.18 (m, 2H, H-1), 3.86 (s, 1H, H-3), 5.19 (s, 1H, H-12), 6.69 (s, 1H, H-1′), 7.27 (m, 1H, H_arom_), 7.52 (d, 1H, J 8 Hz, H_arom_), 8.44 (m, 2H, H_arom_). ^13^C NMR (δ, ppm): 15.6, 15.7, 16.7, 17.1, 18.5, 21.3, 23.3, 23.9, 24.2, 28.0, 28.7, 30.8, 32.7, 36.7, 38.9, 39.2, 39.8, 40.7, 41.6, 42.0, 44.5, 46.7, 47.7, 53.2, 55.5, 80.7 (C-3), 118.9, 123.3, 124.9 (C-12), 134.8, 137.5 (C-2), 138.6 (C-13), 144.1, 145.2, 148.3, 181.4 (C-28). Analysis calculated for C_36_H_51_NO_3_ (*M* 545.39): C 79.22, H 9.42, N 2.57; found: C 79.21, H 9.40, N 2.56. APCI (*m*/*z*): 546.39 (M + H)^+^ 100%.

#### 3.1.5. Synthesis of Compounds **19**, **20**, **33** and **34**

To a solution of 3-oximino-derivatives (1 mmol), obtained from **8** or **28**, in dry dioxane (15 mL) SOCl_2_ (0.4 mL) was added and the mixture was stirred for 30 min, then poured into H_2_O (50 mL); the precipitate was filtered, washed with water and dried. The residue was purified by column chromatography on Al_2_O_3_ eluting using petroleum ether—ethyl acetate (10:1 to 0:10) as eluent.

N-(3,4-Seco-2-cyano-olean-4(23),12(13)-diene-28-oyl)-methylpiperaine **19**. Yield 0.16 g (30%). *R_f_* 0.82; mp 200–201 °C. [α]*_D_*^20^ + 10 (*c* 0.1, CHCl_3_). ^1^H NMR (δ, ppm): 0.72, 0.81, 0.83, 0.84, 1.12, 1.71 (6 s, 18H, 6CH_3_), 1.12–2.41 (m, 23H, CH and CH_2_), 2.26 (s, 3H, NCH_3_), 2.52–2.48 (m, 4H, 2CH_2_), 3.52–3.70 (m, 4H, 2CH_2_), 4.61 and 4.87 (both d, ^2^J = 0.8 Hz, 2H, H-24), 5.25 (s, 1H, H-12). ^13^C NMR (δ, ppm): 11.5, 16.9, 19.1, 20.5, 22.6, 23.0, 23.6, 24.0, 24.2, 25.8, 26.4, 27.9, 29.8, 30.4, 31.5, 33.0, 34.0, 34.4, 38.0, 38.8, 39.5, 42.3, 43.6, 45.2, 45.9, 46.3, 47.3, 50.7, 55.1, 114.1, 120.3, 120.7 (C-12), 145.0 (C-13), 146.9, 174.9 (C-28). Analysis calculated for C_35_H_55_N_3_O (*M* 533.83): C 78.75, H 10.39, N 7.87; found: C 78.74, H 10.36, N 7.85. APCI (*m*/*z*): 534.43 (M + H)^+^ 100%.

N-(3-Oxo-3a-homo-3a-aza-olean-12-en-28-oyl)-methylpiperazine **20**. Yield 0.38 g (68%). *R_f_* 0.20; mp 156–157 °C. [α]*_D_*^20^ + 8 (*c* 0.1, CHCl_3_). ^1^H NMR (δ, ppm): 0.78, 0.82, 0.89, 0.99, 1.01, 1.15, 1.31 (7 s, 21H, 7CH_3_), 1.21–2.21 (m, 23H, CH and CH_2_), 2.31 (s, 3H, NCH_3_), 2.31–2.52 (m, 4H, 2CH_2_), 3.41–3.71 (m, 4H, 2CH_2_), 5.28 (s, 1H, H-12), 5.62 (br. s, 1H, NH). ^13^C NMR (δ, ppm): 13.9, 14.8, 19.2, 21.5, 23.5, 24.1, 25.5, 25.7, 27.7, 28.2, 29.3, 30.4, 30.9, 31.4, 31.8, 32.4, 34.7, 36.9, 37.0, 37.6, 37.8, 38.3, 38.8, 39.3, 39.8, 40.1, 41.3, 43.0, 43.3, 43.8, 43.8, 43.9, 44.1, 45.2, 51.3, 52.0, 52.3, 52.5, 53.0, 53.2, 53.7, 54.2, 119.3, 142.4, 172.6, 174.9 (C-28). Analysis calculated for C_35_H_57_N_3_O_2_ (*M* 551.73): C 76.18, H 10.41, N 7.61; found: C 76.16, H 10.40, N 7.60. APCI (*m*/*z*): 552.45 (M + H)^+^ 100%.

N-(3,4-Seco-2-cyano-ursan-4(23),12(13)-dien-28-oyl)-methylpiperazine **33**. Yield 0.17 g (32%). *R_f_* 0.82; mp 197–198 °C. [α]*_D_*^20^ + 17 (*c* 0.1, CHCl_3_). ^1^H NMR (δ, ppm): 0.72, 0.81, 0.88, 0.99, 1.12, 1.76 (6 s, 18H, 6CH_3_), 1.12–2.61 (m, 23H, CH and CH_2_), 2.21 (s, 3H, NCH_3_), 2.21–2.65 (m, 4H, 2CH_2_), 3.31–3.62 (m, 4H, 2CH_2_), 4.41 and 4.71 (both d, J 0.8 Hz, 2H, H-24), 5.32 (s, 1H, H-12). ^13^C NMR (δ, ppm): 13.5, 17.0, 19.1, 20.4, 23.0, 23.5, 23.6, 23.7, 24.0, 25.6, 27.2, 29.3, 30.7, 32.3, 33.0, 34.0, 34.3, 37.8, 39.1, 39.4, 42.0, 42.4, 45.7, 46.2, 46.4, 50.5, 52.4, 53.8, 54.4, 55.5, 114.2, 120.1, 123.0 (C-12), 139.0 (C-13), 146.6, 177.7 (C-28). Analysis calculated for C_35_H_55_N_3_O (*M* 533.80): C 78.75, H 10.39, N 7.87; found: C 78.74, H 10.36, N 7.85. APCI (*m*/*z*): 534.43 (M + H)^+^ 100%.

N-(3-Oxo-3a-homo-3a-aza-ursan-12-en-28-oyl)-methylpiperazine **34**. Yield 0.34 g (62%). *R_f_* 0.28; mp 144–145 °C. [α]*_D_*^20^ + 4 (*c* 0.1, CHCl_3_). ^1^H NMR (δ, ppm): 0.72, 0.81, 0.89, 0.99, 1.11, 1.39, 1.71 (7 s, 21H, 7CH_3_), 1.12–2.41 (m, 23H, CH and CH_2_), 2.21 (s, 3H, NCH_3_), 2.31–2.52 (m, 4H, 2CH_2_), 3.29–3.61 (m, 4H, 2CH_2_), 5.15 (s, 1H, H-12), 5.63 (br. s, 1H, NH). ^13^C NMR (δ, ppm): 17.5, 17.7, 18.9, 20.8, 21.2, 23.0, 23.3, 24.5, 27.8, 28.2, 30.4, 30.6, 32.7, 33.2, 33.6, 33.7, 34.1, 35.7, 37.6, 38.6, 39.4, 39.9, 40.3, 41.5, 42.5, 45.3, 46.0, 53.4, 54.1, 54.5, 55.0, 124.7 (C-12), 138.9 (C-13), 175.1, 177.6 (C-28). Analysis calculated for C_35_H_57_N_3_O_2_ (*M* 551.73): C 76.18, H 10.41, N 7.61; found: C 76.16, H 10.40, N 7.60. APCI (*m*/*z*): 552.45 (M + H)^+^ 100%.

#### 3.1.6. Synthesis of Compounds **21** and **35**

A mixture of compound **20** or **34** (0.5 mmol) and LiAlH_4_ (230 mg, 0.75 mmol) in dry THF (15 mL) was refluxed for 1 h and then poured into a 5% HCl solution (100 mL). The crude product was extracted with CHCl_3_ (40 mL), the organic layer was washed with H_2_O, dried under CaCl_2_, and evaporated in vacuo. The residue was purified by column chromatography on Al_2_O_3_ using chloroform and chloroform-ethanol (100:1) as eluent.

N-(3-Deoxy-3a-homo-3a-aza-olean-12-en-28-oyl)-methylpiperazine (**21**). Yield 0.17 g (64%), *R_f_* 0.28; mp 138–139 °C. [α]*_D_*^20^ + 48 (*c* 0.2, CHCl_3_).^1^H NMR (δ, ppm): 0.80, 0.82, 0.88, 1.02, 1.12, 1.35, 1.62 (7 s, 21H, 7CH_3_), 1.15–2.23 (m, 24H, CH, CH_2_), 2.21 (s, 3H, NCH_3_), 2.19–2.61 (m, 4H, 2CH_2_), 3.51–3.70 (m, 4H, 2CH_2_), 3.00–3.10 (m, 1H, H3a), 3.21–3.31 (m, 1H, H3b), 5.23 (s, 1H, H-12).^13^C NMR (δ, ppm): 14.5, 16.5, 16.6, 19.3, 22.0, 22.4, 22.8, 23.2, 25.8, 27.4, 28.0, 30.1, 31.0, 32.5, 33.6, 33.9, 35.8, 36.6, 37.3, 40.9, 41.2, 42.8, 45.9, 46.4, 47.3, 47.5, 49.3, 54.5, 55.3, 55.4, 55.8, 63.0 (C-3), 121.4 (C-12), 144.5 (C-13), 175.4 (C-28). Analysis calculated for C_35_H_59_N_3_O (*M* 537.86): C 78.16, H 11.06, N 7.81; found: C 78.15, H 11.05, N 7.80. APCI (*m*/*z*): 538.47 (M + H)^+^ 100%.

N-(3-Deoxy-3a-homo-3a-aza-ursan-12-en-28-oyl)-methylpiperazine (**35**). Yield 0.21 g (79%), *R_f_* 0.28; mp 140–141 °C. [α]*_D_*^20^ + 48 (*c* 0.1, CHCl_3_).^1^H NMR (δ, ppm): 0.72, 0.81, 0.86, 0.99, 1.12, 1.36, 1.55 (7 s, 21H, 7CH_3_), 1.12–2.12 (m, 24H, CH, CH_2_), 2.21 (s, 3H, NCH_3_), 2.19–2.61 (m, 4H, 2CH_2_), 3.51–3.70 (m, 4H, 2CH_2_), 3.00–3.10 (m, 1H, H3a), 3.21–3.31 (m, 1H, H3b), 5.19 (s, 1H, H-12). ^13^C NMR (δ, ppm): 16.7, 17.0, 17.2, 21.2, 21.8, 23.4, 23.7, 24.0, 26.3, 27.9, 30.7, 31.4, 32.7, 33.5, 33.8, 36.6, 37.3, 37.5, 38.8, 39.1, 38.4, 39.6, 40.7, 41.3, 42.2, 45.6, 47.3, 47.8, 52.5, 55.1, 56.7, 63.9 (C-3), 125.4 (C-12), 138.1 (C-13), 178.2 (C-28). Analysis calculated for C_35_H_59_N_3_O (*M* 537.86): C 78.16, H 11.06, N 7.81; found: C 78.17, H 11.03, N 7.79. APCI (*m*/*z*): 538.46 (M + H)^+^ 100%.

### 3.2. NCI-60 Cytotoxicity Drug Screen

The NCI-60 cell line panel is organized into nine subpanels with diverse histology representing leukemia, melanoma, non-small-cell lung, colon, kidney, ovarian, breast, prostate, and central nervous system cancers. Details of the NCI-60 cell line screening protocols and reporting procedures have been described previously [69,70,71,72]. Briefly, test compounds were assayed at a single-dose concentration (10 μM) in the full NCI-60 cancer cell line panel. Upon initial indication of activity in the single-dose experiment, compounds were subsequently tested at five doses starting at 100 μM and decreasing by logarithmic dilution to a final concentration of 0.01 μM. Cell viability after 48 h of incubation was visualized using sulforhodamine B. Through the use of a time zero cell control, the total cell growth can be determined for each cell line, thus allowing calculations of GI_50_, TGI, and LC_50_.

### 3.3. Cell Cycle Analysis

The cell cycle of HEK293, A549, MCF-7, and SH-SY5Y cells was measured by a flow cytometry assay. Briefly, after incubation with vehicle (0.1% DMSO, Sigma Aldrich, St. Louis, MO, USA) or compound 29 at its IC_50_ value for 72 h, cells were harvested and centrifuged (400× *g*, 5 min). The pellets were then gently resuspended in 1 mL of ice-cold 70% ethanol and incubated for 24 h at −20 °C. After permeabilization, the cells were washed twice with phosphate-buffered saline (PBS, Sigma Aldrich, St. Louis, MO, USA), resuspended in PBS, containing RNase A (0.5 mg/mL; Sigma Aldrich, St. Louis, MO, USA), and incubated for 5 min at room temperature. Then PI (propidium iodide, 50 µg/mL; Sigma Aldrich, St. Louis, MO, USA) was added and suspensions were incubated for another 30 min. The PI fluorescence of individual cells/nuclei was measured on Novocyte 2060 flow cytometer (Agilent Technologies, Inc., Santa Clara, CA, USA) in linear scale. Data analysis was performed using the cell cycle module of NovoExpress 1.3.0 software (Agilent Technologies, Inc., Santa Clara, CA, USA). Data are expressed as mean ± S.E.M from three experiments, performed in triplicate. Comparison of cell cycle phases was performed using Wilcoxon *t*-test (Statistica 12.5 (TIBCO Software Inc., Palo Alto, CA, USA).

### 3.4. Cell Apoptosis Assay

For the apoptotic stages detection HEK293 and MCF-7 cells were cultured in 12-well plates (Corning Inc., Gllendale, AZ, USA) (5 × 10^5^ cells/well) in DMEM, containing 10% FBS for 24 h. Cells were treated with compound 29 (at appropriate IC_50_ concentrations) for various time points. After incubation with the compound, 29 cells were harvested and stained with Metabolic Activity Dead Cell Apoptosis Kit with C12 Resazurin, Annexin V APC, and SYTOX Green (#V35114, Thermo Fisher Scientific, Waltham, MA USA) according to the manufacturer’s recommendations. The samples were analyzed by NovoCyte 2060 flow cytometer (Agilent Technologies, Inc., Santa Clara, CA, USA), using 488 nm excitation and collecting fluorescence emission with 530/30 bandpass filter for SYTOX Green live/dead cell staining; using 633 nm excitation and collecting fluorescence emission with 690/50 BP filter for Annexin V APC. Following the application of the standard fluorescence compensation technique, cell percentages of Annevin V/SYTOX dual parameter dot plot were used for statistical analysis (15,000 events were collected in each probe).

### 3.5. Mitochondrial Membrane Potential Assay

Alterations of mitochondrial membrane potential were measured by JC-1 reagent (#T3168, Thermo Fisher Scientific, Waltham, MA USA) according to the manufacturer’s recommendations. HEK293 and MCF-7 cells were cultured in 12-well plates (Corning Inc., Gllendale, AZ, USA) (5 × 10^5^ cells/well) in DMEM, containing 10% FBS for 24 h. Cells were treated with compound 29 (at appropriate IC_50_ concentrations) for various time points. Samples were analyzed by NovoCyte 2060 flow cytometer (Agilent Technologies, Inc., Santa Clara, CA, USA), using 488 nm excitation and collecting fluorescence emission with 530/30 bandpass filter for JC-1 monomers (green) and 590/30 bandpass filter for JC-1 aggregates (red). Following the application of the standard fluorescence compensation technique, the ratio of medians for JC-1 monomers (green) and JC-1 aggregates (red) fluorescence was used for statistical analysis (15,000 events were collected in each probe).

### 3.6. Caspase 8, 9 Activity Assay

For flow cytometry detection of activated caspase-8 and caspase-9 in apoptotic cells, HEK293 and MCF-7 were cultured in 12-well plates (Corning Inc., Gllendale, AZ, USA) (5 × 10^5^ cells/well) in DMEM, containing 10% FBS for 24 h. Cells were treated with compound 29 (at appropriate IC_50_ concentration) for various time points. After incubation with the compound, 29 cells were harvested and stained with Vybrant FAM Caspase-8 Assay Kit (# V35119, Thermo Fisher Scientific, Waltham, MA USA) and CaspGLOW Fluorescein Active Caspase-9 Staining Kit (#88-7006-42, Thermo Fisher Scientific, Waltham, MA USA) according to the manufacturer’s recommendations. The samples were analyzed by NovoCyte 2060 flow cytometer (Agilent Technologies, Inc., Santa Clara, CA, USA), using 488 nm excitation and collecting fluorescence emission; a 530/30 bandpass filter for caspase reagent, and a 690/50 BP filter for propidium iodide (PI) dead cell staining. Following the application of the standard fluorescence compensation technique, medians of fluorescence histogram into caspase^+^/PI^−^ cell populations were used for statistical analysis (15,000 events were collected in each probe gated as “live cells”).

### 3.7. Measurement of Intracellular Reactive Oxygen Species Level

HEK293 and MCF-7 were cultured for 24 h in 12-well plates (Corning Inc., Gllendale, AZ, USA) (5 × 10^5^ cells/well) in DMEM, containing 10% FBS, for flow cytometry detection of ROS generation. Cells were treated with compound 29 (at its IC_50_ value for certain cell lines) for various time points. After incubation with compound 29, culture media were replaced by loading media (in serum-free DMEM) with CM-H_2_DCFDA (5 µM; #C6827, Thermo Fisher Scientific, Waltham, MA USA). After the staining procedure (30 min. 37 °C, 5% CO_2_), loading media was replaced by DMEM, containing 10% FBS for CM-H_2_DCFDA intracellular esterases cleavage (20 min, 37 °C, 5% CO_2_). The samples were harvested and analyzed by NovoCyte 2060 flow cytometer (Agilent Technologies, Inc., Santa Clara, CA, USA), using 488 nm excitation and collecting fluorescence emission; a 530/30 bandpass filter for CM-H_2_DCFDA and a 690/50 BP filter for propidium iodide (PI) dead cell staining. Following the application of the standard fluorescence compensation technique, medians of fluorescence histogram into CM-H_2_DCFDA ^+^/PI^−^ cell populations were used for statistical analysis (15,000 events were collected in each probe gated as “live cells”).

### 3.8. Statistical Analysis

Flow cytometry data were statistically analyzed using the Wilcoxon *t*-test (Statistica 12.5 (TIBCO Software Inc., Palo Alto, CA, USA).). Data are expressed as means ± S.E.M. Normal distribution of data was evaluated by the Shapiro-Wilk’s test.

### 3.9. Network-like Similarity Graphs Analysis

Analysis was performed with DataWarrior [55], a freely available program that implements molecular descriptors calculation, network-like similarity graph construction, and data mining techniques. SkelSpheres and Flexophore fingerprints were computed for imported 2D structures of the target compounds. To search for discontinuous regions in the network containing cytotoxicity cliffs pairs encoding critical structure variations for cytotoxicity we used a Tanimoto automatically determined similarity threshold. Graph nodes were color-coded according to mean growth percentages obtained for the particular compound in the NCI-60 cytotoxicity screen.

## 4. Conclusions

Thus, a series of new oleanane and ursane triterpenic acids and their C28 amides with a modified A-ring was synthesized and screened for cytotoxic activity against the NCI-60 cancer cell line panel. The results of the assay showed that eleven triterpenoids possess cytotoxicity against cancer cells, and six of them were selected for complete dose–response studies. Additionally, we complemented the results obtained by applying the network-like similarity graphs approach to the mining of relevant structure–cytotoxicity relationships trends. We have found that C2-modified triterpenic acids and their C28 amides demonstrate discontinuous SAR with complex pharmacophore-defined dependencies. The SAR analysis revealed that modification of 3-oxo-triterpenic acid by Claisen-Schmidt reaction to introduce a C2-furfurylidene fragment has a positive effect on anticancer activity for both oleanane and ursane types, as well as the synthesis of *N*-methylpiperazinylamides of 3β-hydroxy-C2-[3-pyridinylidene]-triterpenic acids. Among the oleanane type triterpenoids, C2-[4-pyridinylidene]-oleanonic morpholinyl amide 13 exhibited sub-μM potencies against 15 different tumor cell lines and revealed particular selectivity for *non-small cell lung cancer* (HOP-92) with a GI_50_ value of 0.0347 μM. The superior results were observed for C2-[3-pyridinylidene]-ursonic *N*-methyl-piperazinyl amide 29, which exhibited a broad-spectrum inhibition activity with GI_50_ < 1 μM against 33 tumor cell lines and < 2 μM against all 60 cell lines. The data for cell cycle analysis indicates that compounds 13 and 29 could exhibit both cytostatic and cytotoxic activity, depending on the cell line evaluated. Our results suggest that the antiproliferative effect of compound 29 is mediated through ROS-triggered apoptosis which involves the mitochondrial membrane potential depolarization and caspase activation.

## Figures and Tables

**Table 1:** Anti-proliferative activity of compounds **7**, **9**, **10**, **12**, **13**, **17**, **23**, **27**, **29**, **31**, and **32**.

Compound	NCS Number ^1^	Range of Growth, % ^2^	One-Dose Mean Growth (%) ^3^	Five-Dose Mean GI_50_ (μM) ^4^	Most Sensitive Cancer Cell Line
**7**	804711	−95.08 to 21.69	−40.62	3.39	LOXIMVI, melanoma
**9**	797794	−32.37 to100.15	55.08	NT ^5^	HL-60(TB), leukemia
**10**	797795	−41.72 to 86.87	50.20	NT	HL-60(TB), leukemia
**12**	804688	−79.45 to 37.32	−10.67	8.13	HT29, Colon Cancer
**13**	806889	−84.00 to 48.59	4.46	1.55	LOX IMVI, Melanoma
**17**	806887	18.33 to 89.94	61.35	NT	NCI-H460, non-small cell lung cancer
**23**	806876	25.52 to 110.87	87.33	NT	SR, leukemia
**27**	804712	−99.47 to 31.21	−58.89	4.57	UO-31, renal cancer
**29**	801984	−97.65 to −9.56	−74.90	0.75	SK-MEL-5, melanoma
**31**	804510	−97.52 to 63.50	29.45	NT	UO-31, renal cancer
**32**	804692	−80.36 to 25.43	−12.29	15.14	COLO-205, colon cancer

^1^ National Service Centre number assigned by the Developmental Therapeutics Program, NCI to compounds tested in the NCI-60 assay; ^2^ Range of growth of cell lines; ^3^ Growth percent at 10 μM vs. negative control; ^4^ Average GI_50_ value of each compound across the 60 cell lines; ^5^ Compounds are not tested at five-dose experiments.

**Table 2:** Cytostatic (log GI_50_) ^a^ and cytotoxic (log LC_50_) ^b^ and log TGI_50_ ^c^ data for compounds **7**, **13**, **27** and **29**.

Compound	Log GI_50_			Log TGI_50_			Log LC_50_		
**7**	Median	∆	Range	Median	∆	Range	Median	∆	Range
**13**	−5.47	0.51	0.77	−4.67	0.74	1.41	−4.13	0.99	1.12
**27**	−5.81	1.65	2.3	−4.60	1.10	1.70	−4.13	1.10	1.23
**29**	−6.12	0.65	0.98	−5.67	0.60	1.04	−5.21	0.22	1.43

^a^ GI_50_: 50% Growth inhibition, concentration of drug resulting in a 50% reduction in net protein increase compared with control cells; ^b^ LC_50_: Lethal concentration, concentration of drug lethal to 50% of cells; ^c^ Total Growth Inhibition; ∆: the average of numbers obtained by subtracted each log_10_ from the average log_10_.

**Table 3:** In vitro anticancer activity of the most active compounds **13** and **29** against 60 human cancer cell lines in the second stage in single concentration 0.01–100 μM.

Subpanel/Cell Lines (μM)	Compound 13			Compound 29			Doxorubicine * NSC 123127	
	GI_50_	TGI	LC_50_	GI_50_	TGI	LC_50_	GI_50_	LC_50_
Leukemia								
CCRF-CEM	0.871	>100	>100	0.360	1.59	—	0.08	100.00
HL-60(TB)	1.16	3.96	>100	0.200	0.981	7.66	0.12	89.33
K-562	0.637	>100	>100	0.266		>100	0.19	100.00
MOLT-4	0.787	>100	>100	0.309	1.38	—	0.03	100.00
RPMI-8226	0.476	3.36	>100	0.171	0.541	—	0.08	100.00
SR	0.365	>100	>100	—		—	0.03	100.00
Non-Small Cell Lung Cancer								
A549/ATCC	1.22	>100	>100	1.06	2.32	5.06	0.06	100.00
EKVX	2.87	>100	>100	1.21	2.60	—	0.41	47.97
HOP-62	1.76	6.73	>100	1.41	2.80	5.54	0.07	67.61
HOP-92	0.0347	11.60	>100	0.177	1.33	3.87	0.10	42.27
NCI-H226	2.09	9.22	>100	1.63	3.62	—	0.05	6.40
NCI-H23	2.41	>100	>100	1.35	2.89	6.18	0.15	13.15
NCI-H322M	4.32	>100	>100	1.27	2.55	5.10	—	—
NCI-H460	1.32	4.17	>100	0.535	1.96	5.78	0.02	51.29
NCI-H522	1.30	6.72	>100	0.328	1.30	3.88	0.03	2.80
Colon Cancer								
COLO 205	1.38	—	>100	0.751	2.17	—	0.18	4.33
HCC-2998	1.66	7.82	>100	1.09	2.41	5.29	0.26	21.68
HCT-116	0.319	2.00	6.95	0.332	1.24	3.69	0.08	54.58
HCT-15	0.939	11.00	>100	0.654	2.02	4.99	6.46	100.00
HT29	0.912	54.5	>100	0.515	1.73	4.23	0.12	67.45
KM-12	2.70	>100	>100	0.651	2.07	5.23	0.27	92.68
SW-620	2.29	>100	>100	0.924	1.51	6.51	0.09	58.61
CNS Cancer								
SF-268	6.33	>100	>100	1.19	2.90	—	0.10	30.48
SF-295	2.03	34.8	>100	1.53	2.89	5.49	0.10	69.98
SF-539	1.73	3.70	7.94	1.28	2.58	5.19	0.12	27.23
SNB-19	6.93	27.7	85.3	0.956	2.23	5.05	0.04	49.77
SNB-75	3.21	>100	>100	1.00	2.24	4.98	0.07	3.30
U251	1.89	10.3	>100	0.547	1.87	4.53	0.04	30.62
Melanoma								
LOX IMVI	1.24	2.72	5.95	0.800	2.13	—	0.07	50.35
MALME-3M	1.57	4.59	38.4	1.08	2.45	5.58	0.12	3.97
M14	1.41	5.23	>100	0.432	1.73	4.73	0.18	4.05
MDA-MB-435	1.82	—	>100	0.844	2.13	4.81	0.25	9.57
SK-MEL-2	2.33	7.26	>100	1.21	2.56	5.41	0.17	1.06
SK-MEL-28	1.84	5.41	31.2	1.15	2.40	5.00	0.21	15.92
SK-MEL-5	1.48	3.01	6.15	0.760	2.05	4.64	0.08	0.49
UACC-257	3.68	>100	>100	1.24	2.59	5.41	0.14	8.15
UACC-62	1.36	40.9	>100	0.870	2.13	4.74	0.12	0.74
Ovarian Cancer								
IGROV1	3.09	>100	>100	1.52	3.31	7.21	0.17	100.00
OVCAR-3	2.06	>100	>100	0.651	1.95	100	0.39	84.33
OVCAR-4	2.36	>100	>100	0.777	2.17	5.30	0.37	74.30
OVCAR-5	2.27	9.45	49.5	1.52	2.84	5.33	0.41	100.00
OVCAR-8	3.52	>100	>100	1.03	2.50	—	0.10	43.25
NCI/ADR-RES	2.49	46.4	>100	0.911	2.53	—	7.16	100.00
SK-OV-3	3.58	>100	>100	1.44	2.76	5.29	0.22	100.00
Renal Cancer								
786–0	1.66	12.4	>100	0.670	2.00	4.58	0.13	51.64
A498	1.83	4.96	83.0	1.39	2.76	5.48	0.10	1.90
ACHN	2.20	21.7	>100	1.03	2.20	4.69	0.08	100.00
CAKI-1	1.07	>100	>100	1.22	2.47	5.00	0.95	100.00
RXF 393	1.22	3.03	7.53	1.00	2.22	4.91	0.10	4.69
SN12C	2.37	20.6	>100	0.804	2.16	5.05	0.07	72.44
TK-10	6.46	>100	>100	1.34	2.64	5.20	—	—
UO-31	0.893	14.2	45.9	0.497	1.87	4.47	0.49	26.18
Prostate Cancer								
PC-3	0.357	46.1	>100	0.275	1.33	3.82	0.32	87.10
DU-145	2.77	36.4	>100	0.989	2.22	4.95	0.11	100.00
Breast Cancer								
MCF7	0.509	15.3	>100	0.484	2.05	5	0.03	51.29
MDA-MB-231/ATCC	3.06	41.1	>100	0.943	2.25	5.16	0.51	34.75
HS 578T	2.56	>100	>100	1.22	5.85	>100	0.33	85.70
BT-549	1.39	10.0	>100	1.04	2.31	—	0.23	21.33
T -47D	0.781	>100	>100	0.511	2.16	6.74	0.06	85.70
MDA-MB-468	0.712	>100	>100	0.352	1.46	5.11	0.05	2.52

* Doxorubicine: the comparison drug.

**Table 4:** Apoptosis events in HEK293 and MCF-7 cells upon compound **29** treatment.

	Apoptosis, % of Cells	
	Early	Late
HEK293; 24 h		
control	0.45 ± 0.02	1.07 ± 0.04
**29**	4.96 ± 1.3 **	7.62 ± 1.04 **
HEK293; 48 h		
control	2.16 ± 0.98	1.84 ± 0.78
**29**	12.11 ± 1.7 **	65.39 ± 3.5 ***
MCF-7; 24 h		
control	4.88 ± 1.23	1.64 ± 0.095
**29**	5.38 ± 1.2	17.36 ± 3.5 **
MCF-7; 48 h		
control	2.12 ± 1.03	0.85 ± 0.01
**29**	10.23 ± 2.4 *	41.76 ± 3.6 ***

Note: Cells were treated with compound **29** for 24 and 48 h. Data are expressed as mean ± S.E.M from two experiments, performed in triplicate (*—*p* < 0.05, **—*p* < 0.01, ***—*p* < 0.001 compared with corresponding control (untreated) cells; Wilcoxon *t*-test).

**Table 5:** Possible mechanism of action for the lead compounds according to CellMiner ^1^.

Compound	Pearson’s Correlation ^2^	*p* Value	NSC #	Name	Target or Mechanism of Action	FDA Status
**7**	–	–	–	–	–	–
**12**	0.511	0.000174	730001	*N*-(2-Aminophenyl)-4-(3-(3,4-dihydro-1*H*-pyrido [3,4-b]indol-2(9*H*)-yl)prop-1-en-2-yl)benzamide	HDAC	–
**13**	0.694	0	734945	*N*-(4-Aminophenyl)-4-(3-(3,4-dihydroisoquinolin-2(1*H*)-yl)prop-1-en-2-yl)benzamide	HDAC	–
	0.594	0.000001	88536	Calusterone	Hormone	FDA approved
	0.554	0.000005	753686	Olaparib	PARP1	FDA approved
	0.563	0.000017	77213	Procarbazine	Alkylating at *N*^7^ position of guanine	FDA approved
	0.594	0.000021	172112	Spirohydantoin mustard	Alkylating at *N*^7^ position of guanine	–
	0.521	0.000023	73754	Fluorodopan	Alkylating at *N*^7^ position of guanine	–
	0.531	0.000025	755809	Vismodegib	SMO and tyrosine kinase	FDA approved
	0.518	0.000031	777193	LDK-378	ALK	FDA approved
	0.528	0.000067	23759	Testolactone	Hormone	FDA approved
**27**	0.514	0.000031	663249	Triapine	Ribonucleotide reductase	Clinical trial
	0.500	0.000054	681634	Camptothecin derivative	Topoisomerase 1	–
**29**	0.528	0.000021	735408	*N*-(2-Aminophenyl)-4-(3-(6-oxophenanthridin-5(6*H*)-yl)prop-1-en-2-yl)benzamide	HDAC	–
	0.564	0.000024	730001	*N*-(2-Aminophenyl)-4-(3-(3,4-dihydro-1*H*-pyrido [3,4-b]indol-2(9*H*)-yl)prop-1-en-2-yl)benzamide	HDAC	–
	0.518	0.000044	734945	*N*-(4-Aminophenyl)-4-(3-(3,4-dihydroisoquinolin-2(1*H*)-yl)prop-1-en-2-yl)benzamide	HDAC	–
**32**	0.588	0.000001	761191	AP-26113	ALK, EGFR	Clinical trial
	0.571	0.000002	764040	Alectinib	PIK3, ALK, mTOR	FDA approved
	0.567	0.000008	730003	*N*-(2-Aminophenyl)-4-(3-(4-(3-(trifluoromethyl)phenyl)piperazin-1-yl)prop-1-en-2-yl)benzamide	HDAC	–
	0.549	0.000008	776422	LDK-378	ALK	FDA approved
	0.507	0.000042	89201	Estramustine	Alkylating at *N*^7^ position of guanine, tubulin	FDA approved
	0.505	0.000053	777193	LDK-378	ALK	FDA approved
	0.524	0.000079	702294	Estramustine	Alkylating at *N*^7^ position of guanine, tubulin	FDA approved

^1^ The drug activity levels used were expressed as pGI_50_ and obtained from the Developmental Therapeutics Program (DTP) at http://dtp.cancer.gov/index.html. ^2^ Pearson’s correlations between the compound and NCI synthetic library, only correlations with *r* > 0.5 were considered.

**Table 6:** Predicted ADMET characteristics of compound **29**.

Category	Property	Predicted Value			Mean Value ^1^
		pkCSM [61]	SwissADME [63]	ADMETlab [64]	
Physicochemical	Water solubility (lg mol/L)	−5.402	−8.51	−6.08	−6.96
	LogP	8.08	6.53	8.08	7.56
Absorption	Intestinal absorption (human, %)	100	Low	++ (0.714)	Yes
	P-glycoprotein substrate	Yes	No	−−− (0.24)	No
	P-glycoprotein I inhibitor	Yes		+++ (0.908)	
Distribution	VDss (human, L/kg)	0.209		0.433	0.32
	BBB permeability (lg BB)	−0.106	No	+ (0.68)	
Metabolism	CYP2D6 substrate	No		− (0.39)	No
	CYP3A4 substrate	Yes		+ (0.591)	Yes
	CYP1A2 inhibitior	No	No	−−− (0.064)	No
	CYP2C19 inhibitior	No	No	− (0.408)	No
	CYP2C9 inhibitior	No	No	− (0.452)	No
	CYP2D6 inhibitior	No	No	− (0.374)	No
	CYP3A4 inhibitior	No	No	+ (0.664)	No
Excretion	Total clearance (mL/min/kg)	0.244		1.331	0.79
	Renal OCT2 substrate	No			
	T_1/2_ (h)			2.01	
Toxicity	AMES toxicity	No		−−− (0.262)	No
	hERG I inhibitor	No			
	hERG II inhibitor	Yes		+ (0.573)	Yes
	Oral rat acute LD_50_ (mg/kg)	164,3		191.2	177.75
	Hepatotoxicity	Yes		− (0.308)	
	Skin Sensitisation	No		− (0.416)	No

^1^ Mean of numerical values or consensus of categorical values, if possible.

## Data Availability

The data presented in this study are available on request from the corresponding authors.

## Supplementary Materials

The following are available online at https://www.mdpi.com/article/10.3390/ijms22189796/s1.
