# Supplementary material for: Novel A-Ring Chalcone Derivatives of Oleanolic and Ursolic Amides with Anti-Proliferative Effect Mediated through ROS-Triggered Apoptosis
Source: Int J Mol Sci. 2021 Sep 10;22(18):9796. doi: 10.3390/ijms22189796 (PMC8465963; doi:10.3390/ijms22189796)
Supplement: Supplementary file 1 [file ijms-22-09796-s001.zip › ijms-1373432-SI.pdf]

SUPPLEMENTARY MATERIAL

Novel A-ring chalcone derivatives of oleanolic and ursolic amides with anti-proliferative effect mediated through ROS-triggered apoptosis

Elmira Khusnutdinova[1], Anastasiya Petrova[1], Zulfia Zileeva[2], Ulyana Kuzmina[2], Liana Zainullina[2], Yulia Vakhitova[2], Denis Babkov[3], Oxana Kazakova[1]*

[1]*Ufa Institute of Chemistry UFRC RAS, 71, pr. Oktyabrya, Ufa, 450054 Russian Federation*

[2]*Institute of Biochemistry and Genetics UFRC RAS, 71, pr. Oktyabrya, Ufa, 450054 Russian Federation*

3Scientific Center for Innovative Drugs, Volgograd State Medical University, 39, Novorossiyskaya st., Volgograd 400087 Russian Federation

*Correspondence: obf@anrb.ru

Abstract: A series of A-ring modified oleanolic and ursolic acid derivatives including C28 amides (3-oxo-C2-nicotinoylidene/furfurylidene, 3β-hydroxy-C2-nicotinoylidene, 3β-nicotinoyloxy-, 2-cyano-3,4-seco-4(23)-ene, indolo-, lactame and azepane) were synthesized and screened for their cytotoxic activity against the NCI-60 cancer cell line panel. The results of the first assay of thirty-two tested compounds showed, that eleven derivatives exhibited cytotoxicity against cancer cells, and six of them were selected for complete dose-response studies. A systematic study of local SARs has been carried out by comparative analysis of potency distributions and similarity relationships among the synthesized compounds using network-like similarity graphs. Among the oleanane type triterpenoids C2-[4-pyridinylidene]-oleanonic C28-morpholinyl amide exhibited sub-micromolar potencies against 15 different tumor cell lines and revealed particular selectivity for Non-Small Cell Lung Cancer (HOP-92) with GI_{50} value of 0.0347 μM. On the other hand, superior results were observed for C2-[3-pyridinylidene]-ursonic *N*-methyl-piperazinyl amide **29**, which exhibited a broad-spectrum inhibition activity with GI_{50} < 1 μM against 33 tumor cell lines and < 2 μM against all 60 cell lines. This compound has been further evaluated for cell cycle analysis to decipher the mechanism of action. The data indicate that compound **29** could exhibit both cytostatic and cytotoxic activity, dependently on cell line evaluated. The cytostatic activity appears to be determined by induction of the cell cycle arrest at the S (MCF-7, SH-SY5Y cells) or G_0/G_1 phases (A549 cells), whereas cytotoxicity of the compound against normal cells is nonspecific and arises from apoptosis without significant alterations in cell cycle distribution (HEK293 cells). Our results suggest that anti-proliferative effect of compound **29** is mediated through ROS-triggered apoptosis that involves mitochondrial membrane potential depolarization and caspase activation.

Table of contents

Table S1. Percentage of growth inhibition of compounds **5-16** against 60 individual cell lines 3
Table S2. Percentage of growth inhibition of compounds **17-24** against 60 individual cell lines 5
Table S2. Percentage of growth inhibition of compounds **25-36** against 60 individual cell lines 7
Figure S1. Percentage of growth inhibition of compounds **7** against 60 individual cell lines 10
Figure S2. Cytostatic and cytotoxic effects of compound **7** at different doses 11
Figure S3. GI_{50}, TGI and LC_{50} mean graphs obtained for compound **7** 13
Figure S4. ^{1}H NMR spectrum of compound **7** 15
Figure S5. ^{13}C NMR spectrum of compound **7** 15
Figure S6. Percentage of growth inhibition of compounds **12** against 60 individual cell lines 16
Figure S7. Cytostatic and cytotoxic effects of compound **12** at different doses 17
Figure S8. GI_{50}, TGI and LC_{50} mean graphs obtained for compound **12** 19
Figure S9. ^{1}H NMR spectrum of compound **12** 21
Figure S10. ^{13}C NMR spectrum of compound **12** 21
Figure S11. Percentage of growth inhibition of compounds **13** against 60 individual cell lines 22
Figure S12. Cytostatic and cytotoxic effects of compound **13** at different doses 23
Figure S13. GI_{50}, TGI and LC_{50} mean graphs obtained for compound **13** 25
Figure S14. ^{1}H NMR spectrum of compound **13** 26
Figure S15. ^{13}C NMR spectrum of compound **13** 26
Figure S16. Percentage of growth inhibition of compounds **27** against 60 individual cell lines 27
Figure S17. Cytostatic and cytotoxic effects of compound **27** at different doses 28
Figure S18. GI_{50}, TGI and LC_{50} mean graphs obtained for compound **27** 30
Figure S19. ^{1}H NMR spectrum of compound **27** 32
Figure S20. ^{13}C NMR spectrum of compound **27** 32
Figure S21. Percentage of growth inhibition of compounds **29** against 60 individual cell lines 33
Figure S22. Cytostatic and cytotoxic effects of compound **29** at different doses 34
Figure S23. GI_{50}, TGI and LC_{50} mean graphs obtained for compound **29** 36
Figure S24. ^{1}H NMR spectrum of compound **29** 38
Figure S25. ^{13}C NMR spectrum of compound **29** 38
Figure S26. Percentage of growth inhibition of compounds **32** against 60 individual cell lines 39
Figure S27. Cytostatic and cytotoxic effects of compound **32** at different doses 40
Figure S28. GI_{50}, TGI and LC_{50} mean graphs obtained for compound **32** 42
Figure S29. ^{1}H NMR spectrum of compound **32** 44
Figure S30. ^{13}C NMR spectrum of compound **32** 44
Cytotoxicity assay 45
Table S3. In vitro cytotoxic activity of compound 29 in human HEK293, A549, MCF-7, SH-SY5Y cell lines 45
Table S4. Gene ontology (GO) term enrichment analysis for lead compounds across the NCI-60 panel cell lines based on CellMiner analysis 46

Table S1. Percentage of growth inhibition of compounds **5-16** against 60 individual cell lines

Panel/Cell Line	**Growth Percent** in 48 h*											
	**5**	**6**	**7**	**8**	**9**	**10**	**11**	**12**	**13**	**14**	**15**	**16**
**Leukemia**												
CCRF-CEM	81.14	55.44	**4.17**	86.21	**19.71**	33.30	85.39	**1.37**	**–6.27**	87.45	48.35	67.23
HL-60(TB)	99.28	64.19	**–16.40**	99.26	**–41.72**	**–32.37**	58.79	**–8.82**	**–37.64**	87.30	38.90	27.41
K-562	81.26	50.78	**10.48**	73.56	**19.02**	**10.88**	59.27	**9.27**	6.99	65.28	45.87	39.94
MOLT-4	93.95	65.99	**2.21**	91.05	**2.92**	**20.46**	67.38	**4.37**	**–16.90**	70.39	60.40	55.06
RPMI-8226	83.64	50.09	**0.32**	83.64	**7.22**	**19.06**	73.82	**–12.00**	**–33.46**	85.46	89.70	43.69
SR	—	—	**21.69**	67.34	**7.07**	**15.50**	51.80	37.32	**–31.32**	86.34	78.30	63.25
**Non-Small Cell Lung Cancer**												
A549/ATCC	75.59	43.10	**–4.53**	82.06	43.79	46.96	76.06	**5.82**	15.20	78.89	76.45	53.88
EKVX	100.95	70.55	**–19.62**	98.94	80.23	78.83	81.95	**0.84**	14.47	78.65	89.98	71.97
HOP-62	91.46	96.55	**–85.28**	89.67	83.10	85.23	90.89	**–2.51**	19.82	89.20	93.24	88.98
HOP-92	89.60	75.24	**–82.14**	99.60	41.02	40.71	50.50	**–21.77**	**–6.70**	95.64	66.78	63.06
NCI-H226	101.80	85.47	**–42.37**	99.80	68.69	79.67	73.08	**–23.01**	**–20.45**	87.35	86.80	71.87
NCI-H23	99.94	83.12	**–25.64**	99.98	72.36	72.79	92.18	**6.10**	19.48	85.67	56.60	74.93
NCI-H322M	90.88	83.12	**–48.84**	90.88	87.64	96.34	80.44	**–16.96**	40.91	67.20	100.87	91.37
NCI-H460	97.90	56.12	**–55.88**	103.2	33.15	**31.86**	94.19	**–16.72**	**–21.75**	60.25	98.00	61.66
NCI-H522	85.11	69.50	**–39.26**	99.00	55.88	49.44	88.72	**–8.98**	**–1.20**	85.30	67.89	66.99
**Colon Cancer**												
COLO 205	—	—	**–80.73**	105.63	41.91	42.94	85.01	**–73.04**	**–21.09**	95.35	87.08	72.06
HCC-2998	110.29	89.16	**–58.14**	110.29	43.78	66.64	81.96	**–37.55**	**–24.52**	87.45	67.50	78.52
HCT-116	82.96	52.66	**–71.83**	103.25	16.39	**20.00**	74.82	**–33.38**	**–32.58**	64.25	50.23	40.48
HCT-15	86.57	50.44	**–38.57**	99.05	36.31	41.43	83.33	**–5.12**	**7.19**	75.85	70.85	66.97
HT29	105.77	67.77	**–56.69**	98.23	5.24	33.07	78.30	**–79.45**	**5.31**	87.25	60.76	50.90
KM-12	90.95	43.99	**–65.88**	100.25	45.83	55.05	84.64	**–38.09**	**6.58**	90.56	75.25	62.32
SW-620	111.99	78.63	**–32.81**	112.05	33.79	44.55	86.56	**13.32**	10.40	98.67	80.57	71.52
**CNS Cancer**												
SF-268	89.70	72.44	**–27.22**	105.00	64.43	68.34	85.08	**22.44**	48.59	96.45	87.30	79.17
SF-295	94.35	69.00	**–56.16**	99.45	67.11	74.03	101.00	**–7.25**	**25.41**	87.65	78.46	76.05
SF-539	99.13	89.54	**–83.33**	87.30	69.29	80.02	83.90	**–26.80**	**–7.46**	100.85	92.87	92.53
SNB-19	101.39	75.54	**–9.61**	99.45	61.23	64.44	81.85	**16.56**	47.48	65.78	56.35	93.97
SNB-75	86.93	77.65	**–15.61**	105.90	85.26	82.11	74.56	**19.52**	42.99	98.78	100.15	99.09
U251	90.76	55.06	**–36.10**	109.26	44.77	44.75	76.35	**–8.69**	**18.38**	92.37	90.31	81.03
**Melanoma**												
LOX IMVI	105.97	75.94	**–95.08**	110.00	40.21	68.52	73.42	**–87.08**	–84.00	87.24	90.25	68.60
MALME-3M	102.16	101.89	**–80.74**	103.25	57.71	63.38	87.42	**–30.13**	–19.70	89.67	67.35	90.51
M14	98.39	83.28	**–31.49**	98.42	57.45	57.46	81.32	**3.32**	–8.66	80.43	79.45	87.84
MDA-MB-435	100.33	97.31	**–70.60**	85.23	48.15	59.08	83.04	**7.02**	6.81	89.34	80.36	82.68

SK-MEL-2	90.28	85.30	**–69.28**	95.30	78.15	81.50	100.70	**–29.87**	–6.74	98.25	68.10	99.08
SK-MEL-28	112.20	98.00	**–79.50**	113.15	74.70	77.95	100.20	**–35.31**	–14.41	100.25	96.30	88.32
SK-MEL-5	109.30	85.98	**–75.85**	99.15	54.27	49.77	95.12	**–77.09**	–63.45	67.45	86.44	74.42
UACC-257	81.41	87.20	**–23.74**	97.40	57.55	43.69	91.48	**–8.97**	14.82	78.80	90.84	89.87
UACC-62	93.31	86.99	**–89.71**	100.32	61.00	52.81	88.83	**–56.03**	2.88	86.25	80.60	75.67
**Ovarian Cancer**												
IGROV1	104.70	101.60	**–38.72**	99.25	74.38	85.65	78.76	**2.18**	33.08	87.24	98.25	106.77
OVCAR-3	95.49	78.13	**–69.95**	99.78	47.16	52.71	78.12	**–14.63**	16.05	67.57	46.50	70.53
OVCAR-4	94.22	76.62	**—**	93.00	57.40	58.06	76.38	**—**	14.20	89.67	78.70	63.73
OVCAR-5	112.79	97.25	**–64.17**	100.75	86.87	100.15	90.14	**–2.10**	30.26	98.10	100.05	94.69
OVCAR-8	87.80	76.93	**9.39**	79.00	55.28	54.01	91.69	**13.24**	33.97	87.45	90.60	88.55
NCI/ADR-RES	105.40	92.24	**–12.60**	110.05	56.33	59.09	81.70	**3.80**	26.18	78.25	69.10	90.03
SK-OV-3	84.15	93.98	**0.15**	87.25	77.58	83.80	97.46	**1.32**	28.89	100.35	98.40	96.56
**Renal Cancer**												
786-0	108.23	95.85	**–19.48**	99.28	67.16	73.66	63.47	**5.39**	26.16	95.67	90.35	84.67
A498	98.30	87.62	**–42.54**	87.25	—	—	106.44	**23.93**	–20.34	78.23	—	—
ACHN	101.73	84.87	**–71.79**	105.67	65.06	66.00	81.38	**2.77**	15.59	89.40	45.36	63.98
CAKI-1	90.76	79.20	**–34.98**	78.36	75.41	66.56	56.75	**5.69**	16.66	86.23	80.26	70.04
RXF 393	114.85	96.57	**–68.46**	97.95	**27.63**	64.93	83.94	**–37.38**	–36.76	97.30	95.36	97.17
SN12C	98.06	85.75	**–52.31**	87.05	49.92	57.83	80.68	**6.59**	18.04	89.90	80.65	84.29
TK-10	109.17	100.92	**–19.13**	85.20	77.11	89.45	90.95	**1.87**	35.94	96.32	90.16	85.13
UO-31	80.42	65.87	**–98.13**	86.40	37.28	38.59	54.75	**–57.64**	11.01	89.90	75.98	70.85
**Prostate Cancer**												
PC-3	81.88	50.31	**–15.99**	79.05	**19.49**	**19.07**	53.27	**4.87**	10.93	76.25	52.31	43.07
DU-145	100.68	83.89	**–44.97**	98.27	68.37	71.58	91.29	**5.35**	21.72	82.38	80.45	78.53
**Breast Cancer**												
MCF7	90.94	56.62	**–8.44**	99.25	**18.13**	**22.91**	81.81	**3.95**	8.43	80.27	78.25	51.86
MDA-MB-231/ATCC	110.58	96.38	**–77.92**	112.30	64.25	59.83	78.13	**–24.34**	33.93	98.60	100.65	94.63
HS 578T	98.42	81.39	**–5.24**	102.40	50.39	54.21	83.95	**3.00**	27.53	87.30	76.35	98.13
BT-549	90.21	79.63	**–27.20**	95.26	80.22	81.97	95.58	**0.61**	7.67	95.42	89.35	91.00
T-47D	—	—	**–0.55**	—	37.02	34.47	52.73	**15.13**	20.16	67.78	47.87	44.76
MDA-MB-468	115.28	80.09	**–3.74**	117.30	44.59	34.31	87.30	**4.01**	3.46	95.35	80.85	78.77

Table S2. Percentage of growth inhibition of compounds **17-24** against 60 individual cell lines

Panel/Cell Line	**Growth Percent** in 48 h*							
	**17**	**18**	**19**	**20**	**21**	**22**	**23**	**24**
**Leukemia**								
CCRF-CEM	67.87	68.28	77.35	92.53	95.57	70.96	104.04	89.30
HL-60(TB)	66.57	64.49	78.09	32.15	100.15	76.67	59.36	78.15
K-562	79.45	44.02	54.33	72.92	82.82	66.08	53.52	90.45
MOLT-4	59.73	64.08	67.17	60.71	84.85	63.04	67.94	78.46
RPMI-8226	72.25	39.79	53.68	87.70	81.29	71.56	94.50	87.50
SR	53.14	—	68.26	64.12	31.93	—	25.52	—
**Non-Small Cell Lung Cancer**								
A549/ATCC	79.10	78.68	71.54	93.11	96.42	69.45	83.54	**8.85**
EKVX	65.28	66.26	—	93.59	96.65	76.99	89.90	**15.84**
HOP-62	87.21	83.67	91.63	94.57	104.69	79.96	96.68	**–29.13**
HOP-92	67.88	64.40	53.84	54.63	71.76	70.68	72.24	**–3.06**
NCI-H226	72.77	91.58	76.78	100.94	100.06	86.92	101.46	**–24.20**
NCI-H23	92.94	73.44	93.75	89.77	98.37	93.28	98.91	**14.27**
NCI-H322M	94.69	71.92	95.83	89.72	96.48	88.60	92.12	**–16.38**
NCI-H460	84.45	51.59	64.06	102.41	106.35	75.84	77.58	**–31.13**
NCI-H522	89.40	59.37	83.96	103.21	95.92	78.15	99.15	**–4.55**
**Colon Cancer**								
COLO 205	94.71	—	76.89	94.20	101.54	—	90.71	**–80.36**
HCC-2998	92.01	77.95	94.15	99.11	102.36	99.44	90.86	**–8.19**
HCT-116	86.54	41.74	55.14	83.82	100.48	70.66	75.86	**–24.55**
HCT-15	81.91	48.98	—	92.88	92.57	81.54	83.25	**–2.04**
HT29	85.83	80.62	65.95	88.77	84.86	71.32	38.93	**–72.14**
KM-12	96.21	41.68	91.61	101.38	106.65	76.34	90.10	**–37.10**
SW-620	86.23	79.67	92.36	94.32	95.80	96.41	93.00	**14.91**
**CNS Cancer**								
SF-268	77.67	73.14	83.06	91.37	110.58	84.61	92.28	**21.76**
SF-295	90.62	56.68	91.69	101.92	102.67	92.25	104.82	**–27.29**
SF-539	83.58	84.10	86.27	84.78	93.21	76.69	92.09	**–42.08**
SNB-19	84.60	73.88	86.12	96.23	97.91	91.76	94.12	**3.05**
SNB-75	70.46	73.70	76.86	76.79	107.19	56.56	92.56	**–3.83**
U251	74.42	51.18	68.97	82.38	93.03	78.31	83.46	**–57.25**
**Melanoma**								
LOX IMVI	84.82	73.57	98.38	92.18	98.76	77.38	72.29	**–91.81**
MALME-3M	87.56	100.37	91.70	87.94	99.94	90.25	110.87	**–24.18**
M14	87.80	79.21	86.48	95.39	102.02	78.02	100.00	**1.69**
MDA-MB-435	86.45	84.72	87.44	87.50	101.44	84.57	90.08	**7.20**
SK-MEL-2	106.98	84.73	101.89	106.39	105.11	95.10	104.58	**–28.07**
SK-MEL-28	89.49	86.91	—	97.72	106.48	89.62	103.38	**–49.14**
SK-MEL-5	82.68	88.44	85.48	94.68	97.80	93.98	91.45	**–64.37**
UACC-257	86.57	78.17	87.75	96.98	101.18	86.10	106.40	**0.78**
UACC-62	77.53	67.10	93.82	89.99	94.55	95.87	94.78	**–70.29**
**Ovarian Cancer**								
IGROV1	92.59	91.24	85.08	92.70	106.21	84.41	70.58	**7.23**
OVCAR-3	89.70	64.39	84.47	90.85	98.37	78.33	96.18	**–39.02**
OVCAR-4	99.17	71.83	78.45	97.79	103.21	72.05	92.69	**—**
OVCAR-5	98.29	106.27	101.26	99.79	109.82	79.79	98.33	**–13.70**
OVCAR-8	82.79	65.59	74.92	99.54	97.48	79.59	98.84	**12.40**

NCI/ADR-RES	77.70	80.30	80.38	89.54	96.43	89.93	82.14	**1.11**
SK-OV-3	90.87	78.23	95.93	107.04	107.58	76.84	100.93	**5.90**
**Renal Cancer**								
786-0	80.25	82.23	85.32	78.90	96.14	74.36	80.01	**1.92**
A498	89.90	81.33	99.15	87.30	91.21	96.52	89.85	**19.00**
ACHN	87.84	81.28	88.97	98.99	106.74	86.87	94.71	**4.57**
CAKI-1	—	61.55	80.89	99.86	97.56	55.22	78.34	**7.71**
RXF 393	85.11	88.60	96.09	87.48	105.63	80.12	74.65	**–13.33**
SN12C	80.44	80.32	89.99	84.93	100.52	88.10	89.25	**3.00**
TK-10	96.88	89.10	94.58	105.73	108.37	92.38	100.72	**8.85**
UO-31	69.17	60.55	67.53	74.16	87.11	57.69	67.71	**–77.03**
**Prostate Cancer**								
PC-3	61.91	42.40	38.57	82.81	80.20	64.83	80.57	**17.45**
DU-145	95.66	70.06	96.84	98.78	114.40	84.59	90.05	**5.00**
**Breast Cancer**								
MCF7	73.50	56.30	47.65	95.30	95.64	69.92	88.32	**3.47**
MDA-MB-231/ATCC	81.31	87.63	90.78	98.88	101.42	79.01	105.66	**–17.58**
HS 578T	88.92	79.97	83.05	69.03	93.30	73.20	85.80	**4.34**
BT-549	94.07	79.31	82.85	83.68	95.75	96.96	99.81	**–7.55**
T-47D	58.79	—	63.76	73.55	92.11	—	75.95	**15.23**
MDA-MB-468	64.10	85.29	73.39	95.22	84.03	97.99	86.64	**5.39**

Table S3. Percentage of growth inhibition of compounds **25-36** against 60 individual cell lines

**Growth Percent** in 48 h*												
Panel/Cell Line	**25**	**26**	**27**	**28**	**29**	**30**	**31**	**32**	**33**	**34**	**35**	**36**
**Leukemia**												
CCRF-CEM	47.42	52.36	**–26.43**	79.54	**–51.66**	64.67	**16.50**	**–1.43**	78.65	89.13	—	81.83
HL-60(TB)	64.49	61.79	**–3.02**	66.57	**–54.38**	57.29	**–39.05**	**–16.46**	97.46	105.16	88.51	103.60
K-562	44.02	44.89	**7.11**	79.45	**–46.27**	40.20	**28.18**	**13.34**	89.80	85.13	98.76	80.66
MOLT-4	64.08	62.66	**–10.76**	59.73	**–51.92**	52.13	**22.55**	**2.42**	90.25	95.65	99.18	87.23
RPMI-8226	39.79	46.74	**–19.29**	72.25	**–59.28**	50.17	**19.52**	**–4.10**	97.55	67.56	105.94	71.50
SR	—	—	**31.21**	53.14	**—**	—	**31.96**	**25.42**	98.57	100.24	111.48	—
**Non-Small Cell Lung Cancer**												
A549/ATCC	36.68	33.79	**–52.50**	79.10	**–73.73**	63.88	**24.83**	**8.85**	105.61	98.70	104.09	78.75
EKVX	66.26	61.74	**–68.38**	65.28	**–81.47**	78.33	42.27	**15.84**	100.22	87.21	98.22	95.81
HOP-62	83.67	83.55	**–75.65**	87.21	**–95.90**	74.68	35.75	**–29.13**	105.25	87.63	106.12	96.63
HOP-92	64.40	76.60	**–87.70**	67.88	**–81.69**	42.79	56.91	**–3.06**	106.06	86.86	103.03	88.90
NCI-H226	91.58	77.28	**–52.26**	72.77	**–57.24**	85.54	37.41	**–24.20**	99.23	100.23	104.45	92.89
NCI-H23	73.44	72.46	**–81.14**	92.94	**–51.20**	93.19	32.53	**14.27**	102.20	108.65	99.96	101.80
NCI-H322M	71.92	74.10	**–73.42**	94.69	**–52.77**	78.20	51.55	**–16.38**	100.90	98.25	99.89	93.13
NCI-H460	51.59	45.68	**–55.64**	84.45	**–65.08**	68.30	**6.22**	**–31.13**	99.38	101.19	111.42	101.19
NCI-H522	59.37	56.95	**–82.75**	89.40	**–79.30**	69.99	**4.91**	**–4.55**	100.15	85.25	95.22	87.10
**Colon Cancer**												
COLO 205	—	—	**–60.86**	94.71	—	—	40.42	**–80.36**	—	—	112.37	—
HCC-2998	77.95	92.43	**–81.38**	92.01	**–92.35**	82.93	38.15	**–8.19**	111.24	98.50	110.45	108.48
HCT-116	41.74	40.36	**–72.97**	86.54	**–88.21**	55.45	**19.00**	**–24.55**	97.70	91.89	97.70	91.89
HCT-15	48.98	44.74	**–71.18**	81.91	**–79.85**	66.01	32.50	**–2.04**	100.56	83.20	101.16	87.47
HT29	80.62	78.88	**–67.91**	85.83	**–79.26**	46.89	**26.35**	**–72.14**	106.76	89.50	105.81	91.49
KM-12	41.68	42.52	**–73.47**	96.21	**–89.25**	67.48	35.73	**–37.10**	100.45	99.13	105.56	99.24
SW-620	79.67	73.66	**–44.87**	86.23	**–49.87**	83.09	39.90	**14.91**	99.67	85.36	102.20	107.07
**CNS Cancer**												
SF-268	73.14	70.65	**–58.68**	77.67	**–80.28**	82.40	50.26	**21.76**	105.62	100.36	107.23	98.93
SF-295	56.68	45.83	**–79.37**	90.62	**–84.56**	87.70	**29.83**	**–27.29**	99.48	89.85	105.54	99.15
SF-539	84.10	84.64	**–80.08**	83.58	**–79.85**	83.16	46.15	**–42.08**	78.60	100.23	102.56	100.72
SNB-19	73.88	64.55	**–52.62**	84.60	**–79.26**	78.19	47.74	**3.05**	76.23	98.30	94.42	96.78

SNB-75	73.70	54.63	–55.90	70.46	–89.25	76.28	62.26	–3.83	88.15	84.05	97.09	81.99
U251	51.18	51.38	–64.53	74.42	–49.87	68.81	17.41	–57.25	99.20	80.03	99.22	89.03
**Melanoma**												
LOX IMVI	73.57	65.46	–91.68	84.82	–97.06	84.26	–6.24	–91.81	87.36	78.50	99.66	99.53
MALME-3M	100.37	94.09	–85.97	87.56	–87.32	101.77	45.70	–24.18	100.15	113.30	106.26	125.60
M14	79.21	78.19	–43.60	87.80	–81.77	65.73	48.85	1.69	100.15	88.23	104.31	100.09
MDA-MB-435	84.72	87.58	–85.11	86.45	–65.11	81.90	42.63	7.20	80.23	98.85	102.23	102.77
SK-MEL-2	84.73	84.30	–64.03	106.98	–82.57	96.33	42.14	–28.07	78.30	100.78	109.34	96.79
SK-MEL-28	86.91	79.45	–79.25	89.49	–85.71	100.98	35.00	–49.14	121.34	87.88	105.34	108.86
SK-MEL-5	88.44	78.60	–91.68	82.68	–97.65	87.67	–46.79	–64.37	104.55	109.34	103.54	108.80
UACC-257	78.17	78.09	–39.11	86.57	–65.28	83.55	22.54	0.78	99.85	96.54	108.37	87.72
UACC-62	67.10	74.50	–94.42	77.53	–74.66	80.21	32.81	–70.29	95.35	100.60	93.11	97.67
**Ovarian Cancer**												
IGROV1	91.24	87.11	–59.55	92.59	–69.17	84.02	3.24	7.23	110.50	110.20	109.52	103.23
OVCAR-3	64.39	70.20	–74.54	89.70	–93.20	76.28	40.07	–39.02	97.85	99.25	110.89	98.33
OVCAR-4	71.83	60.84	—	99.17	–24.86	73.58	—	—	120.65	89.60	106.39	90.57
OVCAR-5	106.27	103.60	–48.80	98.29	–77.81	86.74	63.50	–13.70	99.45	107.85	100.44	106.81
OVCAR-8	65.59	62.28	–18.97	82.79	–76.16	76.67	25.41	12.40	112.30	87.56	101.47	89.66
NCI/ADR-RES	80.30	83.70	–58.89	77.70	–75.10	80.61	37.90	1.11	99.57	101.45	105.76	102.49
SK-OV-3	78.23	81.30	–17.34	90.87	–89.52	82.91	59.30	5.90	105.15	98.75	105.03	94.92
**Renal Cancer**												
786-0	82.23	85.32	–40.32	80.25	–85.02	81.38	40.60	1.92	89.30	92.65	96.34	101.36
A498	81.33	78.91	–56.23	89.90	–83.16	93.86	53.75	19.00	105.25	98.36	89.30	100.07
ACHN	81.28	74.33	–97.88	87.84	–94.41	91.02	20.79	4.57	111.25	96.86	104.31	95.76
CAKI-1	61.55	63.78	–74.62	—	–42.50	75.13	26.24	7.71	89.33	98.35	90.90	86.85
RXF 393	88.60	92.37	–84.03	85.11	–94.36	83.81	55.08	–13.33	105.30	89.25	114.31	92.23
SN12C	80.32	77.41	–56.00	80.44	–95.31	78.93	27.98	3.00	99.45	100.23	97.53	94.24
TK-10	89.10	91.60	–73.40	96.88	–97.40	86.08	43.67	8.85	110.56	86.87	104.46	100.02
UO-31	60.55	59.12	–99.47	69.17	–92.48	62.38	–97.52	–77.03	68.25	89.30	84.85	84.20
**Prostate Cancer**												
PC-3	42.40	47.47	–67.12	61.91	–79.62	33.85	29.87	17.45	56.42	78.34	99.49	75.26
DU-145	70.06	75.24	–73.15	95.66	–81.53	89.30	50.42	5.00	89.31	97.40	108.71	103.71
***Breast* Cancer**												
MCF7	56.30	48.93	–74.6	73.50	–73.82	58.54	23.91	3.47	67.42	67.67	91.44	87.69

			**7**									
MDA-MB-231/ATCC	87.63	96.29	**–81.50**	81.31	**–80.14**	81.36	**15.58**	**–17.58**	98.25	90.35	104.93	101.49
HS 578T	79.97	67.24	**–13.88**	88.92	**–30.69**	70.00	53.17	**4.34**	97.31	89.21	100.51	99.21
BT-549	79.31	80.55	**–62.50**	94.07	**–81.21**	89.29	50.80	**–7.55**	89.46	87.67	95.72	96.62
T-47D	—	—	**–14.27**	58.79	**—**	—	**20.28**	**15.23**	99.23	—	100.99	—
MDA-MB-468	85.29	68.79	**–38.00**	64.10	**–83.23**	41.07	**19.17**	**5.39**	111.13	100.25	107.06	95.80

The structures of leader compounds

Derivatives of oleanane type

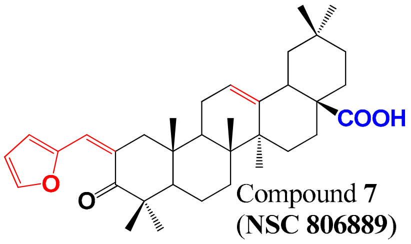

Compound **7** (**NSC 806889**)

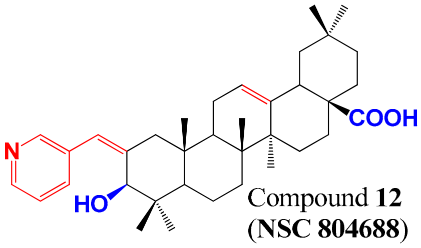

Compound **12** (**NSC 804688**)

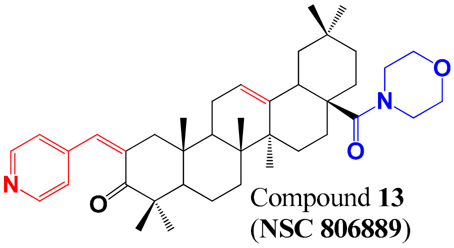

Compound **13** (**NSC 806889**)

Derivatives of ursane type

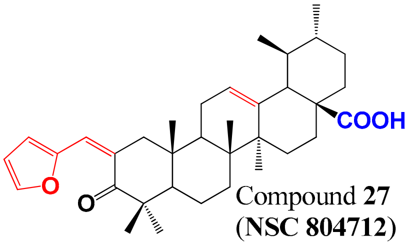

Compound **27** (**NSC 804712**)

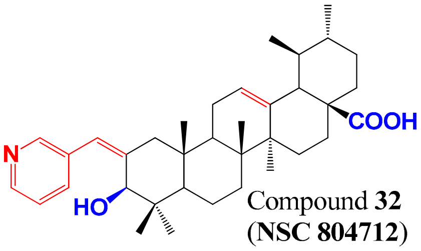

Compound **32** (**NSC 804712**)

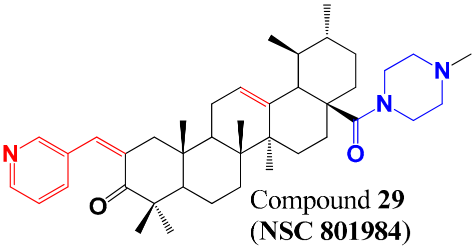

Compound **29** (**NSC 801984**)

Figure S1. Percentage of growth inhibition of compounds **7** against 60 individual cell lines

Developmental Therapeutics Program	NSC: D-806889 / 1	Conc: 1.00E-5 Molar	Test Date: Aug 27, 2018
One Dose Mean Graph	Experiment ID: 1808OS00		Report Date: Jan 01, 2019

Panel/Cell Line	Growth Percent
Leukemia	
CCRF-CEM	-6.27
HL-60(TB)	-37.64
K-562	6.99
MOLT-4	-16.90
RPMI-8226	-33.46
SR	-31.32
Non-Small Cell Lung Cancer	
A549/ATCC	15.20
EKVX	14.47
HOP-62	19.82
HOP-92	-6.70
NCI-H226	-20.45
NCI-H23	19.48
NCI-H322M	40.91
NCI-H460	-21.75
NCI-H522	-1.20
Colon Cancer	
COLO 205	-21.09
HCC-2998	-24.52
HCT-116	-32.58
HCT-15	7.19
HT29	5.31
KM12	6.58
SW-620	10.40
CNS Cancer	
SF-268	48.59
SF-295	25.41
SF-539	-7.46
SNB-19	47.48
SNB-75	42.99
U251	18.38
Melanoma	
LOX IMVI	-84.00
MALME-3M	-19.70
M14	-8.66
MDA-MB-435	6.81
SK-MEL-2	-6.74
SK-MEL-28	-14.41
SK-MEL-5	-63.45
UACC-257	14.82
UACC-62	2.88
Ovarian Cancer	
IGROV1	33.08
OVCAR-3	16.05
OVCAR-4	14.20
OVCAR-5	30.26
OVCAR-8	33.97
NCI/ADR-RES	26.18
SK-OV-3	28.89
Renal Cancer	
786-0	26.16
A498	-20.34
ACHN	15.59
CAKI-1	16.66
RXF 393	-36.76
SN12C	18.04
TK-10	35.94
UO-31	11.01
Prostate Cancer	
PC-3	10.93
DU-145	21.72
Breast Cancer	
MCF7	8.43
MDA-MB-231/ATCC	33.93
HS 578T	27.53
BT-549	-2.62
T-47D	20.16
MDA-MB-468	3.46
Mean	4.46
Delta	88.46
Range	132.59

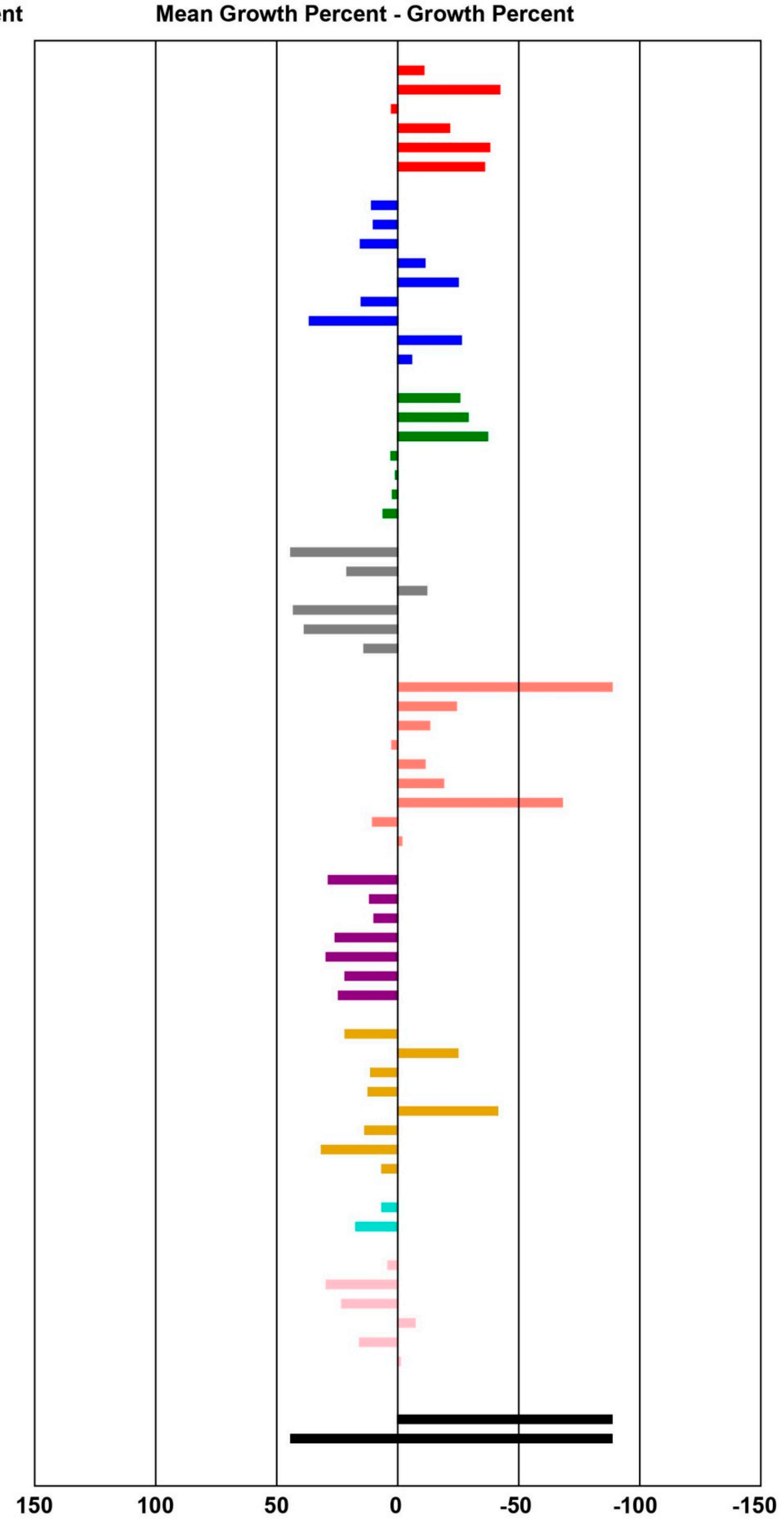

Figure S2. Cytostatic and cytotoxic effects of compound **7** (NSC 804711) on the NCI-60 panel of cancer cell lines at different doses, ranging from 10 nM to 100 μM. Results are displayed as dose-response curves (% growth versus sample concentration) for each cell line in the nine cancer subpanels.

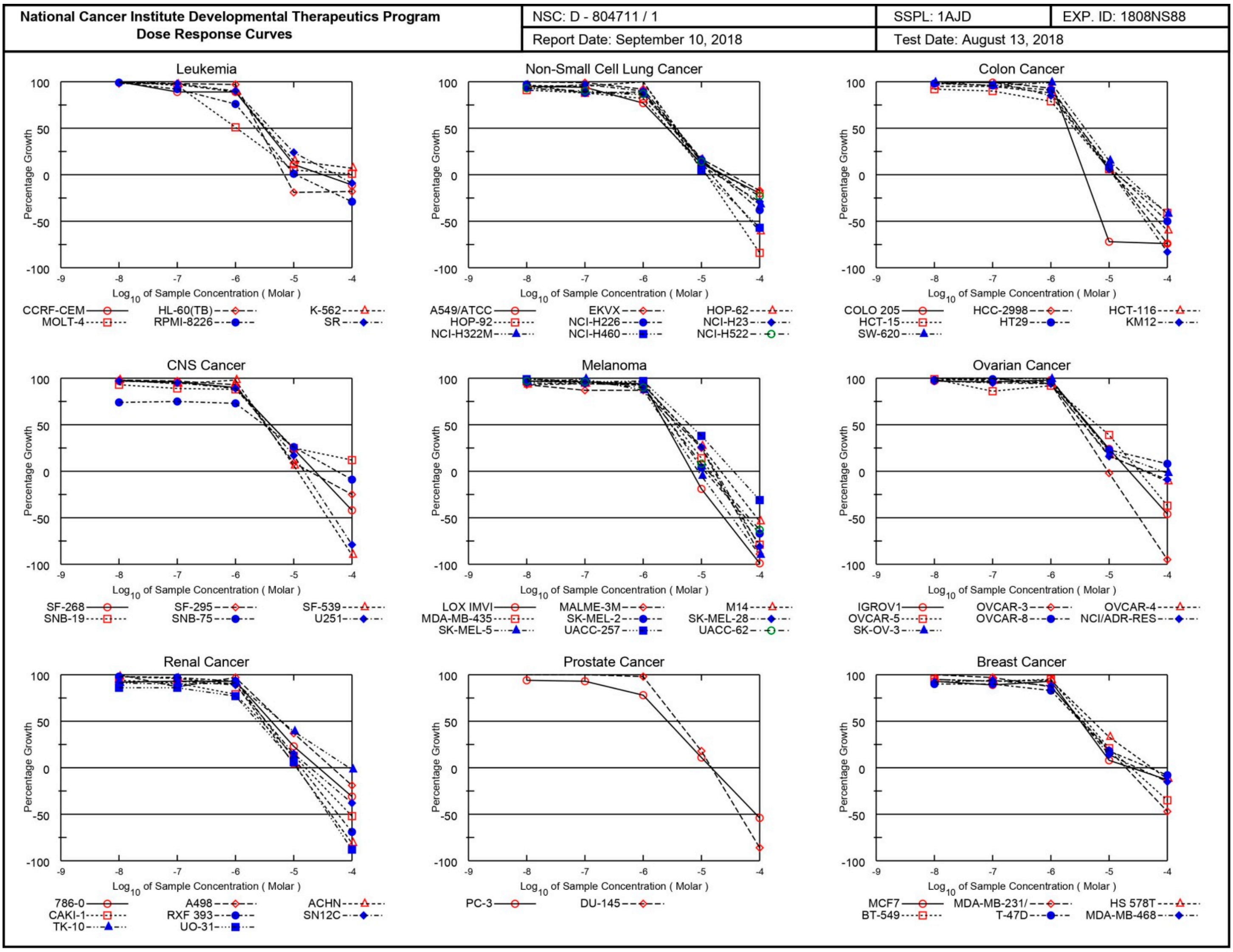

National Cancer Institute Developmental Therapeutics Program
In-Vitro Testing Results

NSC : D - 804711 / 1	Experiment ID : 1808NS88	Test Type : 08	Units : Molar
Report Date : September 10, 2018	Test Date : August 13, 2018	QNS :	MC :
COMI : OA-fur	Stain Reagent : SRB Dual-Pass Related	SSPL : 1AJD	

			Log10 Concentration												
	Time		Mean Optical Densities					Percent Growth							
Panel/Cell Line	Zero	Ctrl	-8.0	-7.0	-6.0	-5.0	-4.0	-8.0	-7.0	-6.0	-5.0	-4.0	GI50	TGI	LC50
**Leukemia**															
CCRF-CEM	0.602	3.095	3.186	2.830	2.819	0.879	0.535	104	89	89	11	-11	3.16E-6	3.16E-5	> 1.00E-4
HL-60(TB)	0.729	3.129	3.077	3.092	3.066	0.589	0.595	98	98	97	-19	-18	2.55E-6	6.84E-6	> 1.00E-4
K-562	0.207	2.267	2.371	2.196	2.043	0.513	0.344	105	97	89	15	7	3.36E-6	> 1.00E-4	> 1.00E-4
MOLT-4	0.625	2.904	2.898	2.820	1.782	0.744	0.654	100	96	51	5	1	1.04E-6	> 1.00E-4	> 1.00E-4
RPMI-8226	0.802	2.697	2.672	2.553	2.241	0.819	0.570	99	92	76	1	-29	2.22E-6	1.07E-5	> 1.00E-4
SR	0.196	0.597	0.601	0.590	0.555	0.293	0.178	101	98	90	24	-9	4.04E-6	5.24E-5	> 1.00E-4
**Non-Small Cell Lung Cancer**															
A549/ATCC	0.421	2.491	2.393	2.370	2.015	0.668	0.336	95	94	77	12	-20	2.60E-6	2.35E-5	> 1.00E-4
EKVX	0.918	2.548	2.572	2.532	2.423	1.188	0.762	101	99	92	17	-17	3.62E-6	3.11E-5	> 1.00E-4
HOP-62	0.475	1.690	1.647	1.630	1.692	0.630	0.186	96	95	100	13	-61	3.75E-6	1.49E-5	7.12E-5
HOP-92	0.810	1.505	1.441	1.422	1.378	0.867	0.127	91	88	82	8	-84	2.69E-6	1.22E-5	4.25E-5
NCI-H226	0.929	2.415	2.375	2.233	2.256	1.164	0.572	97	88	89	16	-38	3.42E-6	1.95E-5	> 1.00E-4
NCI-H23	0.661	2.219	2.107	2.166	2.059	0.810	0.467	93	97	90	10	-29	3.13E-6	1.76E-5	> 1.00E-4
NCI-H322M	0.478	1.552	1.479	1.435	1.405	0.655	0.326	93	89	86	16	-32	3.31E-6	2.19E-5	> 1.00E-4
NCI-H460	0.451	3.327	3.350	3.337	3.327	0.609	0.195	101	100	100	5	-57	3.38E-6	1.22E-5	7.76E-5
NCI-H522	0.696	2.253	2.141	2.099	2.051	0.917	0.539	93	90	87	14	-23	3.22E-6	2.43E-5	> 1.00E-4
**Colon Cancer**															
COLO 205	0.416	2.076	2.086	2.065	2.132	0.117	0.110	101	99	103	-72	-74	2.02E-6	3.89E-6	7.50E-6
HCC-2998	0.458	1.603	1.577	1.678	1.519	0.537	0.121	98	107	93	7	-74	3.15E-6	1.22E-5	5.08E-5
HCT-116	0.179	2.219	2.124	2.112	1.983	0.286	0.071	95	95	88	5	-60	2.90E-6	1.20E-5	6.96E-5
HCT-15	0.362	2.539	2.371	2.328	2.086	0.492	0.214	92	90	79	6	-41	2.50E-6	1.34E-5	> 1.00E-4
HT29	0.258	2.096	2.056	2.017	1.929	0.418	0.129	98	96	91	9	-50	3.14E-6	1.41E-5	> 1.00E-4
KM12	0.568	2.912	2.968	2.905	2.569	0.699	0.097	102	100	85	6	-83	2.77E-6	1.16E-5	4.24E-5
SW-620	0.213	1.903	1.878	1.832	1.887	0.463	0.124	99	96	99	15	-42	3.82E-6	1.82E-5	> 1.00E-4
**CNS Cancer**															
SF-268	0.499	1.840	1.801	1.790	1.709	0.818	0.289	97	96	90	24	-42	4.03E-6	2.30E-5	> 1.00E-4
SF-295	1.198	3.228	3.187	3.159	3.081	1.372	0.903	98	97	93	9	-25	3.22E-6	1.81E-5	> 1.00E-4
SF-539	0.917	2.502	2.463	2.413	2.473	1.016	0.091	98	94	98	6	-90	3.34E-6	1.16E-5	3.84E-5
SNB-19	0.562	2.294	2.165	2.104	2.085	0.999	0.769	93	89	88	25	12	4.02E-6	> 1.00E-4	> 1.00E-4
SNB-75	0.939	1.685	1.493	1.499	1.481	1.132	0.853	74	75	73	26	-9	3.04E-6	5.46E-5	> 1.00E-4
U251	0.370	2.038	1.994	1.956	1.852	0.649	0.080	97	95	89	17	-79	3.45E-6	1.50E-5	5.02E-5
**Melanoma**															
LOX IMVI	0.594	3.190	3.123	3.077	3.045	0.480	0.008	97	96	94	-19	-99	2.46E-6	6.77E-6	2.44E-5
MALME-3M	0.610	1.598	1.531	1.472	1.473	0.860	0.066	93	87	87	25	-89	3.99E-6	1.66E-5	4.55E-5
M14	0.462	2.262	2.139	2.145	2.138	0.952	0.212	93	94	93	27	-54	4.51E-6	2.16E-5	8.87E-5
MDA-MB-435	0.476	2.602	2.544	2.497	2.514	0.772	0.100	97	95	96	14	-79	3.63E-6	1.41E-5	4.87E-5
SK-MEL-2	1.109	2.420	2.406	2.386	2.312	1.161	0.362	99	97	92	4	-67	2.99E-6	1.14E-5	5.71E-5
SK-MEL-28	0.668	2.190	2.109	2.092	2.083	1.060	0.125	95	94	93	26	-81	4.35E-6	1.74E-5	5.10E-5
SK-MEL-5	0.751	2.793	2.707	2.779	2.524	0.711	0.074	96	99	87	-5	-90	2.51E-6	8.74E-6	3.36E-5
UACC-257	0.768	2.064	2.048	2.018	2.022	1.260	0.531	99	96	97	38	-31	6.24E-6	3.56E-5	> 1.00E-4
UACC-62	1.093	3.213	3.159	3.131	3.001	1.257	0.401	97	96	90	8	-63	3.06E-6	1.29E-5	6.50E-5
**Ovarian Cancer**															
IGROV1	0.374	1.845	1.802	1.780	1.813	0.734	0.201	97	96	98	24	-46	4.48E-6	2.22E-5	> 1.00E-4
OVCAR-3	0.486	1.714	1.765	1.709	1.646	0.478	0.024	104	100	94	-2	-95	2.90E-6	9.61E-6	3.29E-5
OVCAR-4	0.755	1.786	1.815	1.760	1.728	0.933	0.673	103	97	94	17	-11	3.76E-6	4.11E-5	> 1.00E-4
OVCAR-5	0.708	1.537	1.530	1.425	1.469	1.030	0.446	99	86	92	39	-37	6.15E-6	3.25E-5	> 1.00E-4
OVCAR-8	0.261	1.479	1.456	1.461	1.428	0.546	0.355	98	99	96	23	8	4.29E-6	> 1.00E-4	> 1.00E-4
NCI/ADR-RES	0.545	1.910	1.874	1.842	1.823	0.762	0.498	97	95	94	16	-9	3.64E-6	4.45E-5	> 1.00E-4
SK-OV-3	0.827	1.869	1.885	1.874	1.859	1.042	0.808	102	100	99	21	-2	4.22E-6	7.94E-5	> 1.00E-4
**Renal Cancer**															
786-0	0.493	2.131	1.996	2.014	2.025	0.866	0.340	92	93	93	23	-31	4.12E-6	2.65E-5	> 1.00E-4
A498	1.443	2.205	2.202	2.107	2.184	1.728	1.165	100	87	97	37	-19	6.14E-6	4.56E-5	> 1.00E-4
ACHN	0.599	2.408	2.364	2.344	2.219	0.667	0.114	98	96	90	4	-81	2.89E-6	1.11E-5	4.31E-5
CAKI-1	0.590	2.336	2.224	2.182	1.963	0.812	0.283	94	91	79	13	-52	2.72E-6	1.57E-5	9.30E-5
RXF 393	0.887	1.569	1.557	1.547	1.519	0.959	0.276	98	97	93	10	-69	3.30E-6	1.36E-5	5.77E-5
SN12C	0.542	2.505	2.344	2.364	2.291	0.836	0.337	92	93	89	15	-38	3.37E-6	1.92E-5	> 1.00E-4
TK-10	0.786	1.818	1.723	1.714	1.727	1.187	0.771	91	90	91	39	-2	6.11E-6	8.98E-5	> 1.00E-4
UO-31	0.773	2.118	1.926	1.924	1.805	0.856	0.092	86	86	77	6	-88	2.39E-6	1.16E-5	3.94E-5
**Prostate Cancer**															
PC-3	0.410	1.967	1.877	1.851	1.621	0.587	0.189	94	93	78	11	-54	2.62E-6	1.49E-5	8.68E-5
DU-145	0.325	1.525	1.587	1.578	1.503	0.547	0.047	105	104	98	18	-86	4.02E-6	1.51E-5	4.55E-5
**Breast Cancer**															
MCF7	0.456	2.628	2.477	2.387	2.467	0.626	0.396	93	89	93	8	-13	3.18E-6	2.35E-5	> 1.00E-4
MDA-MB-231/ATCC	0.621	1.575	1.580	1.547	1.449	0.803	0.331	100	97	87	19	-47	3.49E-6	1.95E-5	> 1.00E-4
HS 578T	0.841	1.820	1.768	1.752	1.759	1.161	0.741	95	93	94	33	-12	5.19E-6	5.41E-5	> 1.00E-4
BT-549	1.082	2.121	2.071	2.052	2.066	1.296	0.703	95	93	95	21	-35	4.01E-6	2.34E-5	> 1.00E-4
T-47D	0.582	1.424	1.341	1.341	1.284	0.731	0.535	90	90	83	18	-8	3.23E-6	4.84E-5	> 1.00E-4
MDA-MB-468	0.872	1.911	1.831	1.844	1.789	1.021	0.742	92	94	88	14	-15	3.29E-6	3.09E-5	> 1.00E-4

Figure S3. GI50 (50% Growth Inhibition), TGI (Total Growth Inhibition) and LC50 (50% Lethal Concentration) mean graphs obtained for compound **7** (NSC 804711) tested at five concentrations (0.01, 0.1, 1, 10, 100 μM) against the NCI-60 human cancer cell lines.

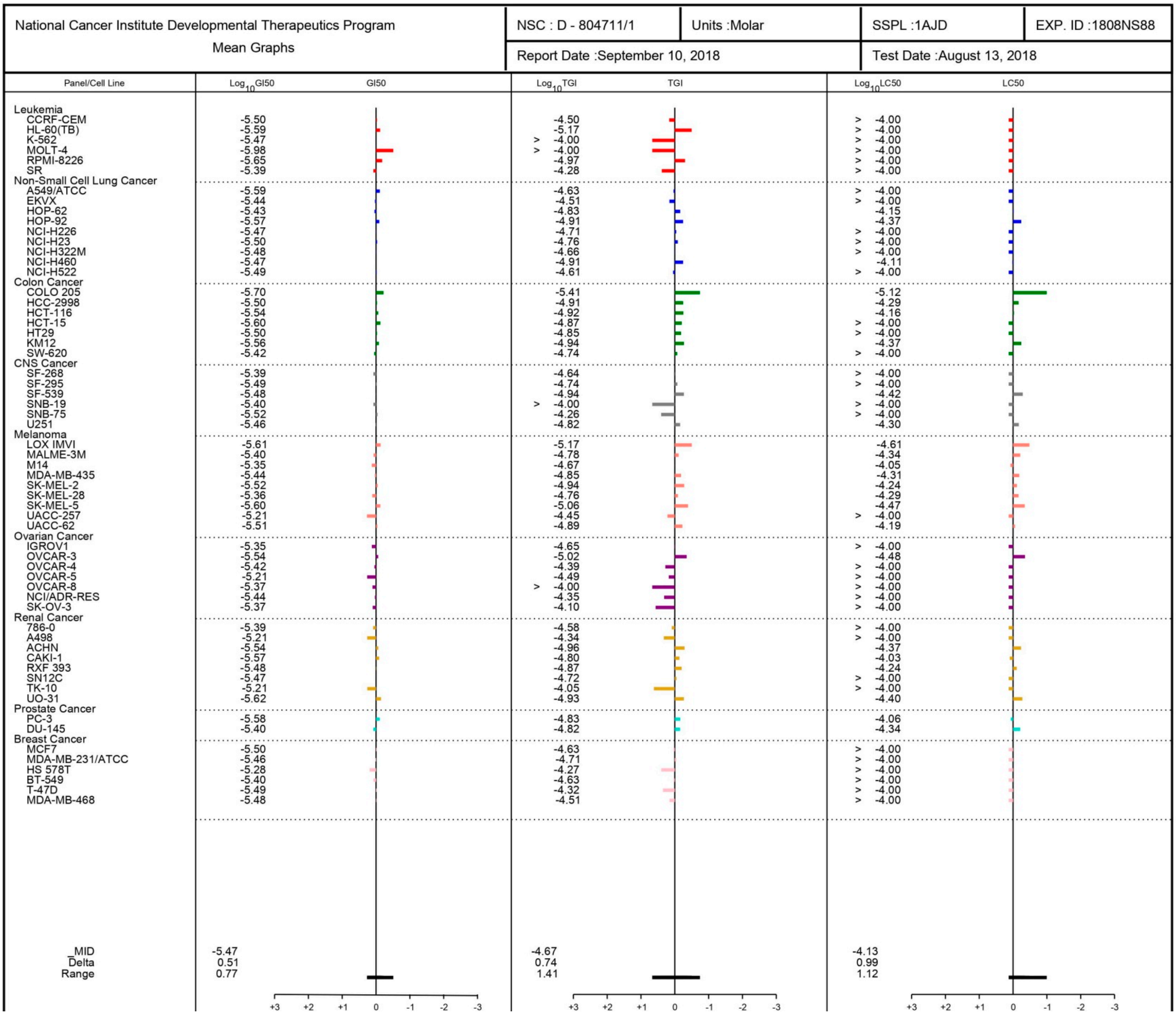

National Cancer Institute Developmental Therapeutics Program Dose Response Curves	NSC : D - 804711/1	SSPL:1AJD	EXP. ID:1808NS88
	Report Date:September 10, 2018	Test Date:August 13, 2018	

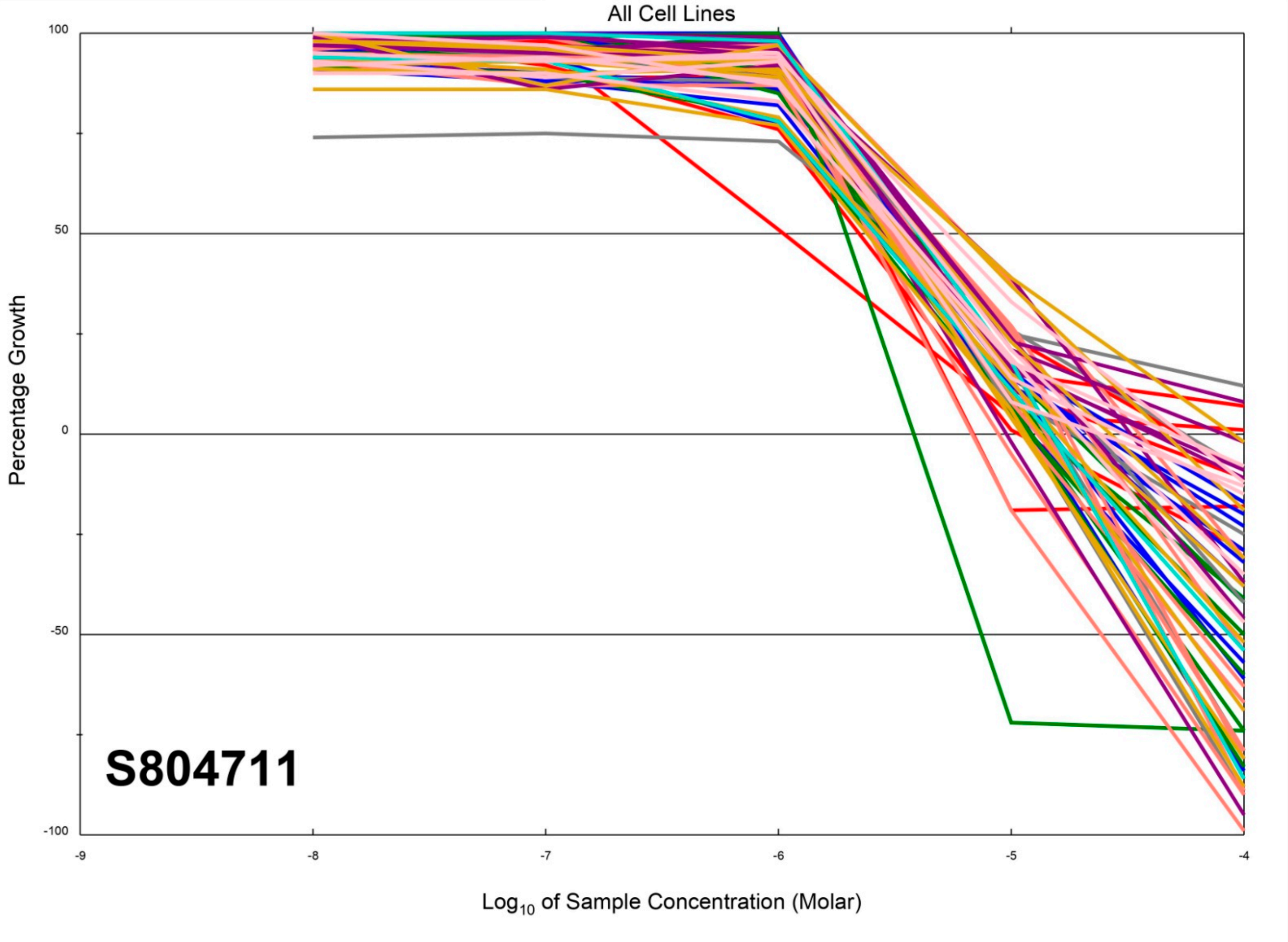

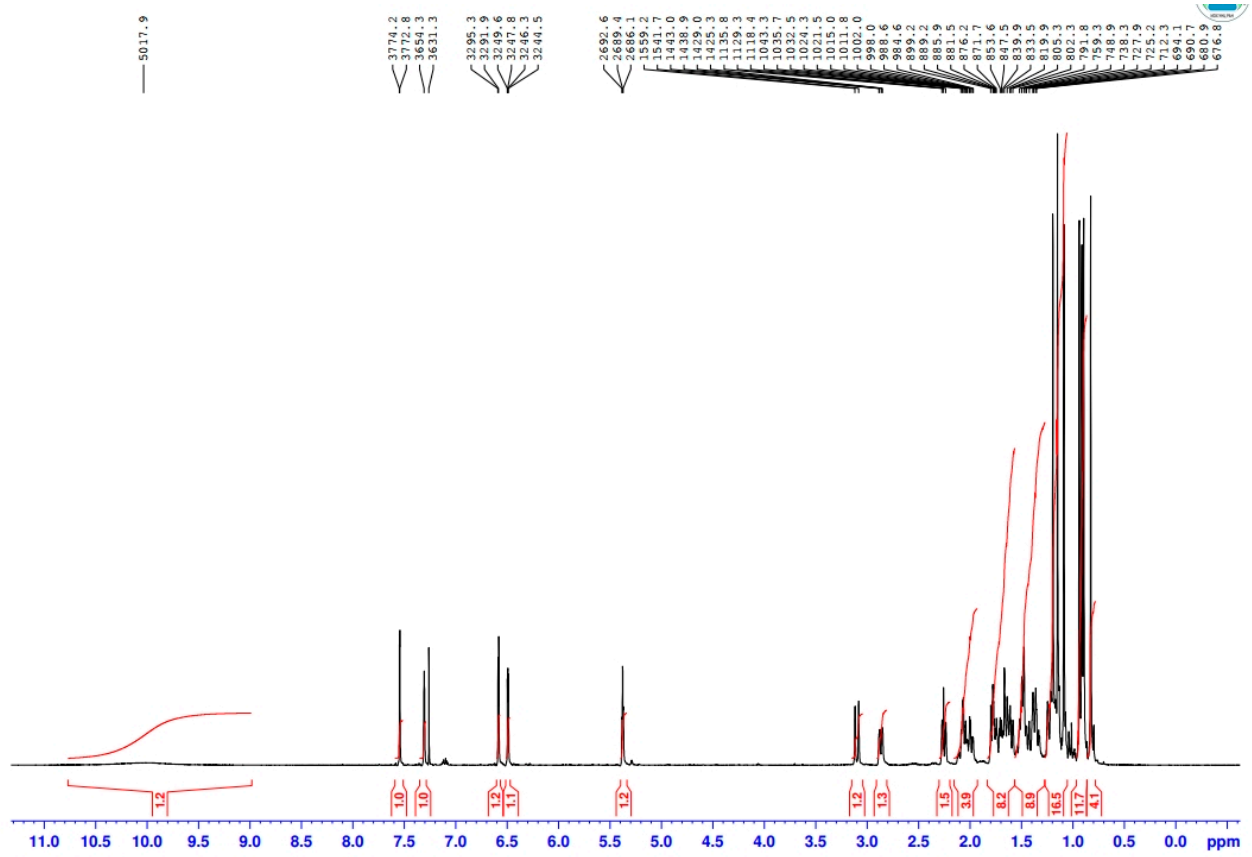

Figure S4. Spectrum NMR ^{1}H of compound **7**

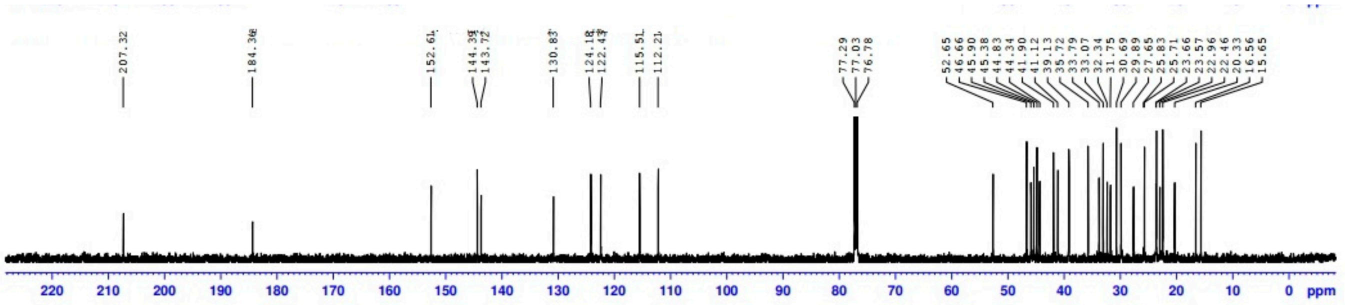

Figure S5. Spectrum NMR ^{13}C of compound **7**

Figure S6. Percentage of growth inhibition of compounds **12** against 60 individual cell lines

Developmental Therapeutics Program One Dose Mean Graph	NSC: D-804688 / 1	Conc: 1.00E-4 Molar	Test Date: Apr 30, 2018
	Experiment ID: 1804OS61		Report Date: Mar 28, 2020

Panel/Cell Line	Growth Percent
Leukemia	
CCRF-CEM	1.37
HL-60(TB)	-8.82
K-562	9.27
MOLT-4	4.37
RPMI-8226	-12.00
SR	37.32
Non-Small Cell Lung Cancer	
A549/ATCC	5.82
EKVX	0.84
HOP-62	-2.51
HOP-92	-21.77
NCI-H226	-23.01
NCI-H23	6.10
NCI-H322M	-16.96
NCI-H460	-16.72
NCI-H522	-8.98
Colon Cancer	
COLO 205	-73.04
HCC-2998	-37.55
HCT-116	-33.38
HCT-15	-5.12
HT29	-79.45
KM12	-38.09
SW-620	13.32
CNS Cancer	
SF-268	22.44
SF-295	-7.25
SF-539	-26.80
SNB-19	16.56
SNB-75	19.52
U251	-8.69
Melanoma	
LOX IMVI	-87.08
MALME-3M	-30.13
M14	3.32
MDA-MB-435	7.02
SK-MEL-2	-29.87
SK-MEL-28	-35.31
SK-MEL-5	-77.09
UACC-257	-8.97
UACC-62	-56.03
Ovarian Cancer	
IGROV1	2.18
OVCAR-3	-14.63
OVCAR-5	-2.10
OVCAR-8	13.24
NCI/ADR-RES	3.80
SK-OV-3	1.32
Renal Cancer	
786-0	5.39
A498	23.93
ACHN	2.77
CAKI-1	5.69
RXF 393	-37.38
SN12C	6.59
TK-10	1.87
UO-31	-57.64
Prostate Cancer	
PC-3	4.87
DU-145	5.35
Breast Cancer	
MCF7	3.95
MDA-MB-231/ATCC	-24.34
HS 578T	3.00
BT-549	0.61
T-47D	15.13
MDA-MB-468	4.01
Mean	-10.67
Delta	76.41
Range	124.40

Mean Growth Percent - Growth Percent

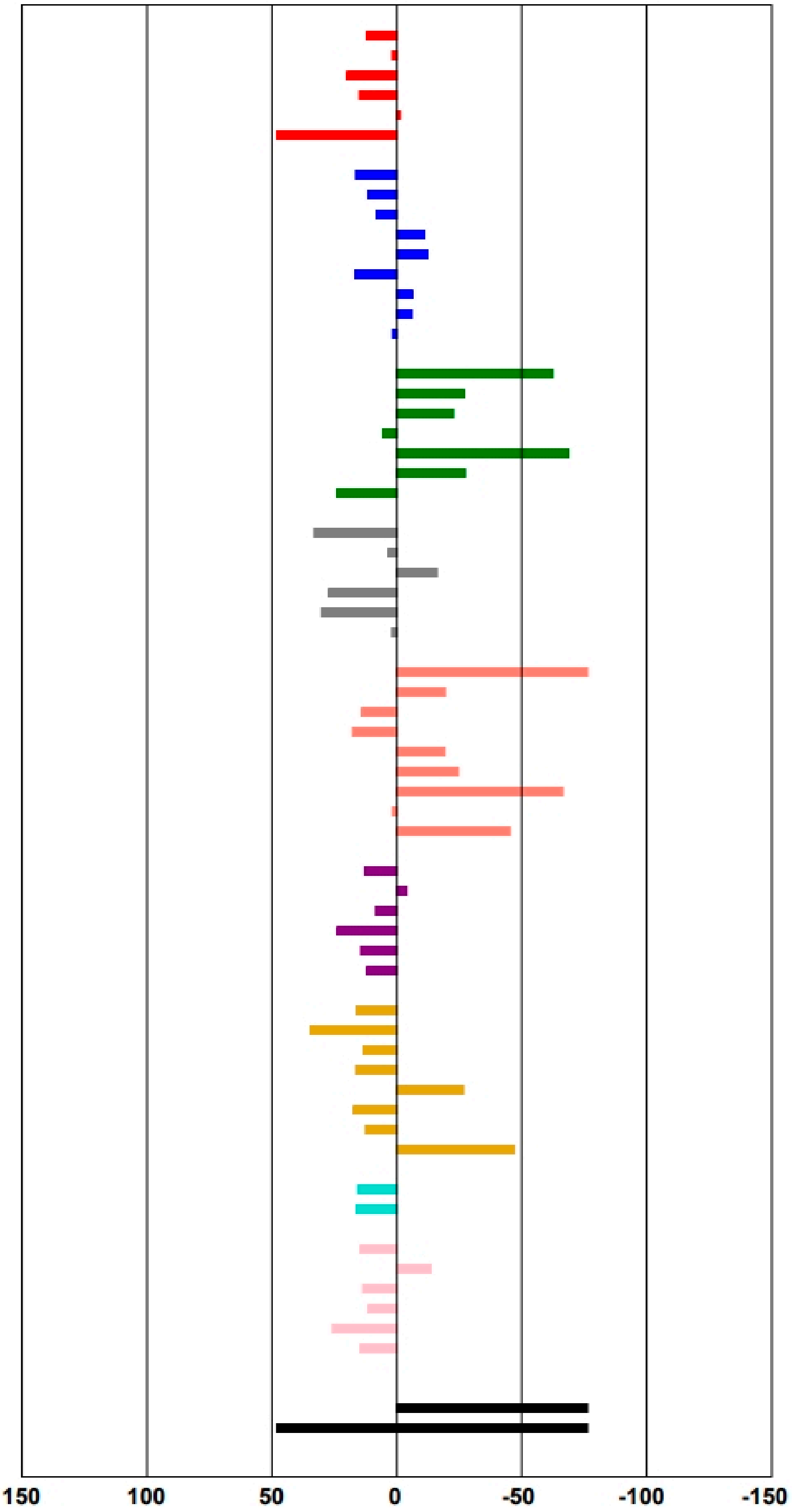

Figure S7. Cytostatic and cytotoxic effects of compound **12** (NSC 804688) on the NCI-60 panel of cancer cell lines at different doses, ranging from 10 nM to 100 μM. Results are displayed as dose-response curves (% growth versus sample concentration) for each cell line in the nine cancer subpanels.

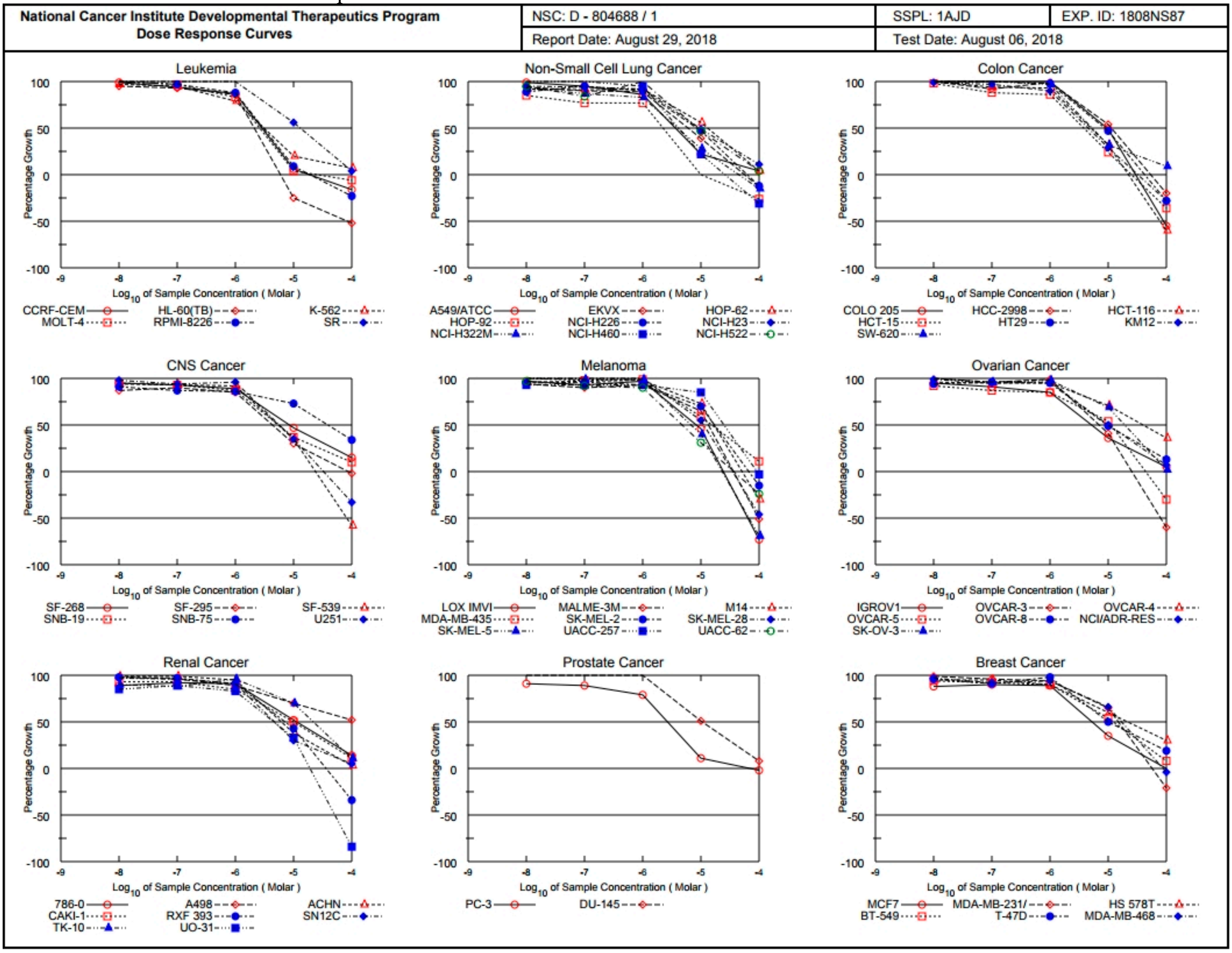

National Cancer Institute Developmental Therapeutics Program
In-Vitro Testing Results

NSC : D - 804688 / 1	Experiment ID : 1808NS87	Test Type : 08	Units : Molar
Report Date : August 29, 2018	Test Date : August 06, 2018	QNS :	MC :
COMI : OA-3NK-3OH	Stain Reagent : SRB Dual-Pass Related	SSPL : 1AJD	

			Log10 Concentration												
	Time		Mean Optical Densities					Percent Growth							
Panel/Cell Line	Zero	Ctrl	-8.0	-7.0	-6.0	-5.0	-4.0	-8.0	-7.0	-6.0	-5.0	-4.0	GI50	TGI	LC50
**Leukemia**															
CCRF-CEM	0.546	2.719	2.695	2.589	2.446	0.674	0.459	99	94	87	6	-16	2.88E-6	1.85E-5	> 1.00E-4
HL-60(TB)	0.909	3.191	3.075	3.031	2.879	0.680	0.435	95	93	86	-25	-52	2.12E-6	5.94E-6	8.33E-5
K-562	0.184	1.968	1.918	1.884	1.598	0.534	0.307	97	95	79	20	7	3.10E-6	> 1.00E-4	> 1.00E-4
MOLT-4	0.571	2.638	2.673	2.571	2.287	0.662	0.536	102	97	83	4	-6	2.63E-6	2.60E-5	> 1.00E-4
RPMI-8226	1.200	3.056	3.056	3.002	2.835	1.362	0.924	100	97	88	9	-23	3.02E-6	1.88E-5	> 1.00E-4
SR	0.279	1.386	1.386	1.412	1.418	0.901	0.326	100	102	103	56	4	1.32E-5	> 1.00E-4	> 1.00E-4
**Non-Small Cell Lung Cancer**															
A549/ATCC	0.550	2.852	2.831	2.741	2.528	1.058	0.638	99	95	86	22	4	3.65E-6	> 1.00E-4	> 1.00E-4
EKVX	0.967	2.584	2.491	2.415	2.414	1.596	0.844	94	90	89	39	-13	6.02E-6	5.66E-5	> 1.00E-4
HOP-62	0.589	1.940	1.836	1.813	1.834	1.344	0.648	92	91	92	56	4	1.30E-5	> 1.00E-4	> 1.00E-4
HOP-92	1.052	1.779	1.667	1.608	1.608	1.053	0.774	85	77	77	.	-26	2.22E-6	1.01E-5	> 1.00E-4
NCI-H226	0.715	2.095	1.942	2.020	1.957	1.367	0.630	89	95	90	47	-12	8.63E-6	6.30E-5	> 1.00E-4
NCI-H23	0.567	2.099	2.020	2.003	1.983	1.323	0.734	95	94	92	49	11	9.64E-6	> 1.00E-4	> 1.00E-4
NCI-H322M	0.770	2.270	2.166	2.075	2.020	1.189	0.658	93	87	83	28	-15	3.99E-6	4.53E-5	> 1.00E-4
NCI-H460	0.103	2.152	2.187	2.201	2.044	0.559	0.072	102	102	95	22	-31	4.14E-6	2.64E-5	> 1.00E-4
NCI-H522	0.984	2.678	2.601	2.403	2.693	1.788	1.055	95	84	101	47	4	8.97E-6	> 1.00E-4	> 1.00E-4
**Colon Cancer**															
COLO 205	0.485	2.248	2.323	2.274	2.328	1.354	0.217	104	101	105	49	-55	9.71E-6	2.96E-5	8.89E-5
HCC-2998	0.509	1.796	1.797	1.692	1.781	1.208	0.410	100	92	99	54	-20	1.14E-5	5.44E-5	> 1.00E-4
HCT-116	0.172	2.048	2.043	1.941	1.922	0.778	0.069	100	94	93	32	-60	5.12E-6	2.24E-5	7.81E-5
HCT-15	0.270	2.007	1.981	1.799	1.764	0.695	0.174	98	88	86	24	-36	3.85E-6	2.56E-5	> 1.00E-4
HT29	0.150	1.390	1.396	1.416	1.368	0.734	0.108	100	102	98	47	-28	8.78E-6	4.21E-5	> 1.00E-4
KM12	0.419	2.366	2.353	2.309	2.173	0.993	0.303	99	97	90	29	-28	4.58E-6	3.27E-5	> 1.00E-4
SW-620	0.173	2.298	2.318	2.303	2.229	0.863	0.363	101	100	97	32	9	5.34E-6	> 1.00E-4	> 1.00E-4
**CNS Cancer**															
SF-268	0.672	2.190	2.109	2.084	2.019	1.393	0.901	95	93	89	47	15	8.68E-6	> 1.00E-4	> 1.00E-4
SF-295	0.914	3.047	2.772	2.843	2.720	1.553	0.897	87	90	85	30	-2	4.30E-6	8.74E-5	> 1.00E-4
SF-539	0.940	2.498	2.409	2.384	2.378	1.493	0.395	94	93	92	35	-58	5.55E-6	2.40E-5	8.21E-5
SNB-19	0.727	2.573	2.472	2.447	2.326	1.418	0.918	95	93	87	37	10	5.55E-6	> 1.00E-4	> 1.00E-4
SNB-75	0.453	1.575	1.471	1.432	1.417	1.276	0.829	91	87	86	73	34	3.86E-5	> 1.00E-4	> 1.00E-4
U251	0.560	2.460	2.415	2.340	2.376	1.218	0.378	98	94	96	35	-33	5.59E-6	3.27E-5	> 1.00E-4
**Melanoma**															
LOX IMVI	0.303	2.306	2.249	2.170	2.245	1.221	0.081	97	93	97	46	-73	8.28E-6	2.42E-5	6.36E-5
MALME-3M	0.390	1.031	0.991	0.965	0.986	0.806	0.191	94	90	93	65	-51	1.34E-5	3.62E-5	9.77E-5
M14	0.418	2.120	2.056	2.076	2.079	1.659	0.294	96	97	98	73	-30	1.67E-5	5.13E-5	> 1.00E-4
MDA-MB-435	0.360	2.616	2.661	2.579	2.594	1.684	0.602	102	98	99	59	11	1.52E-5	> 1.00E-4	> 1.00E-4
SK-MEL-2	0.861	2.185	2.121	2.137	2.098	1.782	0.730	95	96	93	70	-15	1.70E-5	6.61E-5	> 1.00E-4
SK-MEL-28	0.798	2.228	2.349	2.319	2.278	1.579	0.433	108	106	103	55	-46	1.11E-5	3.50E-5	> 1.00E-4
SK-MEL-5	1.043	3.195	3.195	3.176	3.177	1.898	0.324	100	99	99	40	-69	6.71E-6	2.32E-5	6.69E-5
UACC-257	1.108	2.629	2.516	2.520	2.527	2.395	1.077	93	93	93	85	-3	2.49E-5	9.28E-5	> 1.00E-4
UACC-62	0.891	3.069	2.999	2.905	2.844	1.562	0.676	97	92	90	31	-24	4.72E-6	3.64E-5	> 1.00E-4
**Ovarian Cancer**															
IGROV1	0.550	2.355	2.264	2.201	2.091	1.192	0.634	95	91	85	36	5	5.13E-6	> 1.00E-4	> 1.00E-4
OVCAR-3	0.435	1.549	1.556	1.509	1.535	0.896	0.174	101	96	99	41	-60	7.08E-6	2.56E-5	7.95E-5
OVCAR-4	0.196	0.859	0.832	0.827	0.827	0.668	0.435	96	95	95	71	36	4.00E-5	> 1.00E-4	> 1.00E-4
OVCAR-5	0.553	1.357	1.289	1.250	1.233	0.987	0.388	92	87	85	54	-30	1.12E-5	4.40E-5	> 1.00E-4
OVCAR-8	0.559	2.626	2.498	2.534	2.528	1.570	0.830	94	96	95	49	13	9.47E-6	> 1.00E-4	> 1.00E-4
NCI/ADR-RES	0.631	2.201	2.193	2.117	2.131	1.410	0.742	99	95	96	50	7	9.82E-6	> 1.00E-4	> 1.00E-4
SK-OV-3	0.941	2.056	2.067	2.014	2.036	1.707	0.961	101	96	98	69	2	1.90E-5	> 1.00E-4	> 1.00E-4
**Renal Cancer**															
786-0	0.509	2.355	2.155	2.208	2.183	1.468	0.763	89	92	91	52	14	1.12E-5	> 1.00E-4	> 1.00E-4
A498	0.817	1.923	1.902	1.882	1.803	1.593	1.394	98	96	89	70	52	> 1.00E-4	> 1.00E-4	> 1.00E-4
ACHN	0.375	1.645	1.633	1.628	1.577	0.856	0.407	99	99	95	38	3	6.11E-6	> 1.00E-4	> 1.00E-4
CAKI-1	0.173	1.558	1.467	1.464	1.345	0.871	0.322	93	93	85	50	11	1.02E-5	> 1.00E-4	> 1.00E-4
RXF 393	1.038	1.993	1.972	1.966	1.910	1.452	0.689	98	97	91	43	-34	7.28E-6	3.66E-5	> 1.00E-4
SN12C	0.613	2.671	2.619	2.595	2.489	1.230	0.717	97	96	91	30	5	4.70E-6	> 1.00E-4	> 1.00E-4
TK-10	0.579	1.645	1.536	1.515	1.598	1.323	0.695	90	88	96	70	11	2.17E-5	> 1.00E-4	> 1.00E-4
UO-31	0.729	2.075	1.874	1.922	1.851	1.178	0.116	85	89	83	33	-84	4.64E-6	1.92E-5	5.12E-5
**Prostate Cancer**															
PC-3	0.514	2.071	1.935	1.899	1.745	0.682	0.505	91	89	79	11	-2	2.66E-6	7.25E-5	> 1.00E-4
DU-145	0.292	1.365	1.384	1.370	1.387	0.842	0.375	102	100	102	51	8	1.07E-5	> 1.00E-4	> 1.00E-4
**Breast Cancer**															
MCF7	0.304	1.964	1.764	1.797	1.780	0.883	0.312	88	90	89	35	.	5.25E-6	> 1.00E-4	> 1.00E-4
MDA-MB-231/ATCC	0.601	1.656	1.645	1.610	1.597	1.285	0.477	99	96	94	65	-21	1.49E-5	5.73E-5	> 1.00E-4
HS 578T	0.587	2.043	1.969	1.964	1.900	1.465	1.023	95	95	90	60	30	2.18E-5	> 1.00E-4	> 1.00E-4
BT-549	0.991	2.261	2.191	2.158	2.140	1.680	1.097	94	92	90	54	8	1.24E-5	> 1.00E-4	> 1.00E-4
T-47D	0.641	1.460	1.426	1.384	1.446	1.049	0.797	96	91	98	50	19	9.88E-6	> 1.00E-4	> 1.00E-4
MDA-MB-468	0.807	2.126	2.081	2.021	2.045	1.682	0.777	97	92	94	66	-4	1.71E-5	8.83E-5	> 1.00E-4

Figure S8. GI50 (50% Growth Inhibition), TGI (Total Growth Inhibition) and LC50 (50% Lethal Concentration) mean graphs obtained for compound **12** (NSC 804688) tested at five concentrations (0.01, 0.1, 1, 10, 100 μM) against the NCI-60 human cancer cell lines.

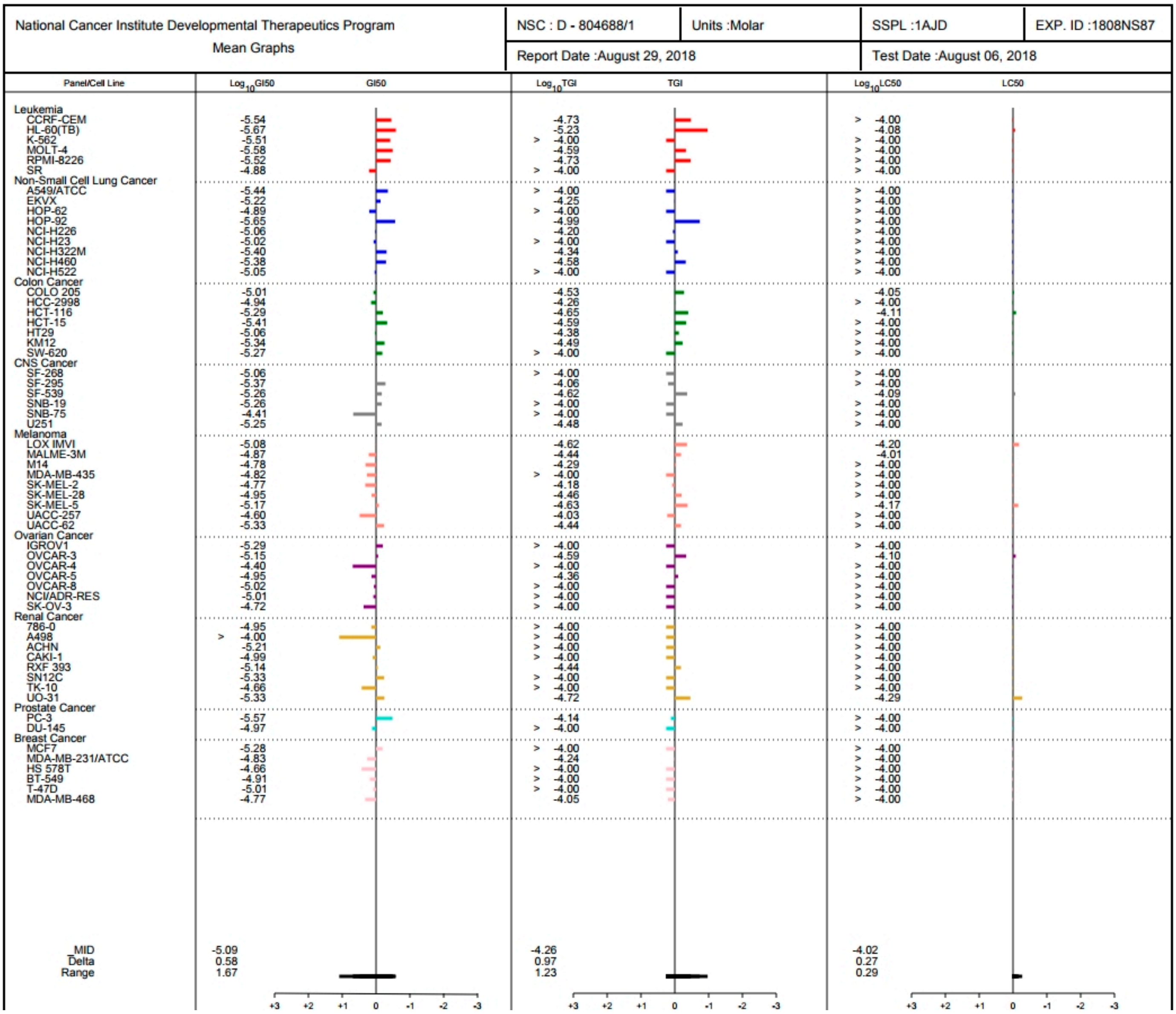

National Cancer Institute Developmental Therapeutics Program<br>Dose Response Curves	NSC : D - 804688/1	SSPL:1AJD	EXP. ID:1808NS87
	Report Date:August 29, 2018	Test Date:August 06, 2018	

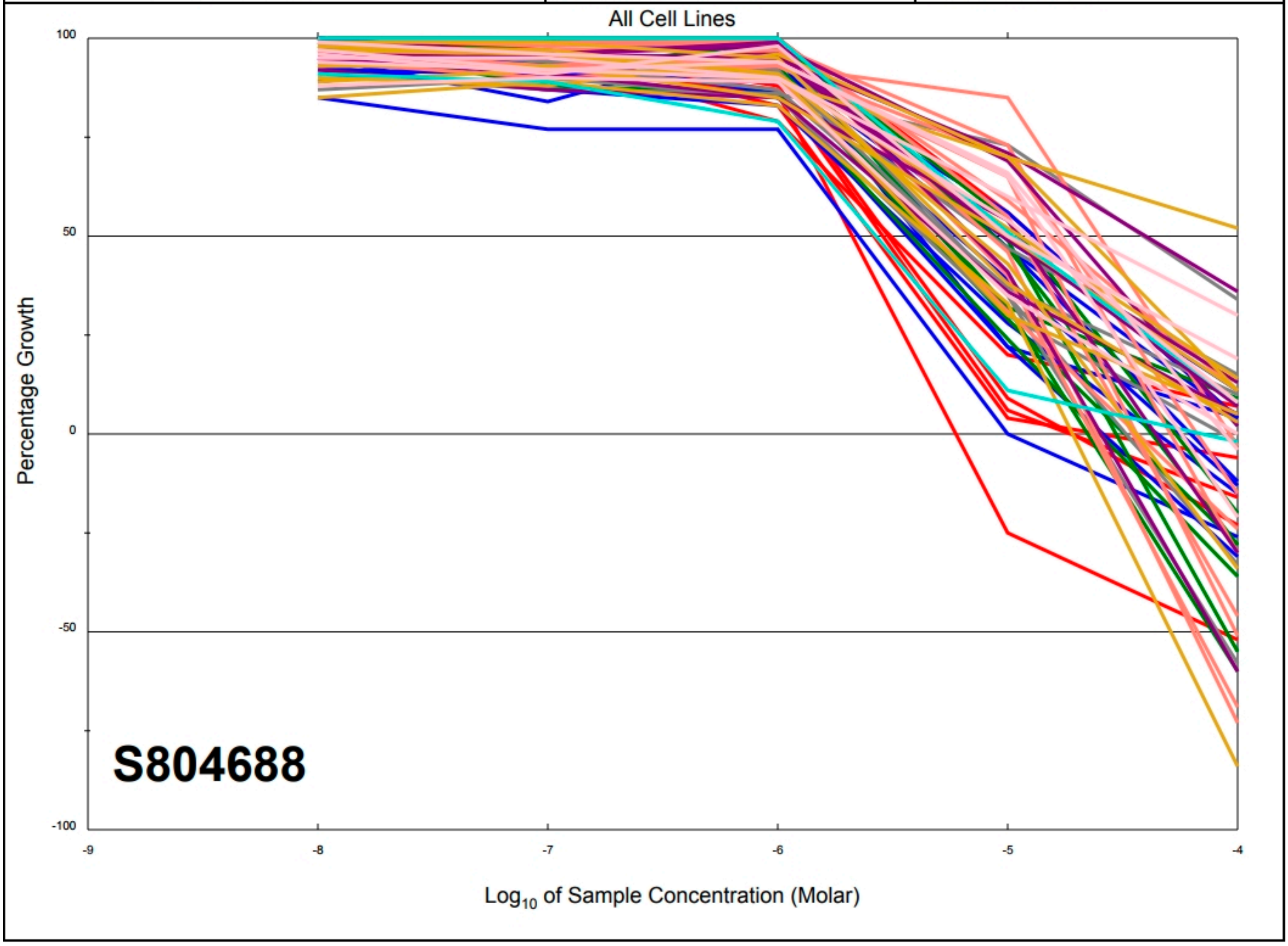

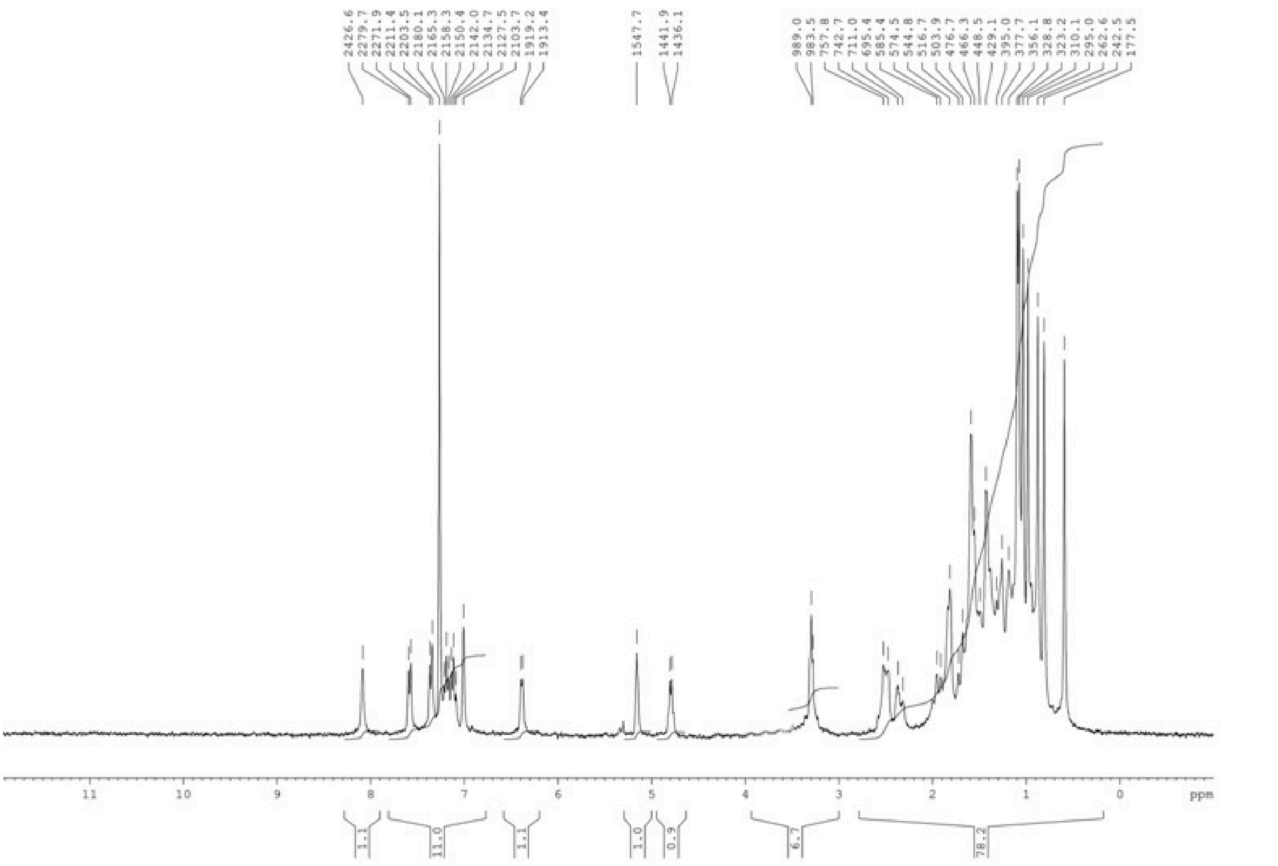

Figure S9. Spectrum NMR ^{1}H of compound **12**

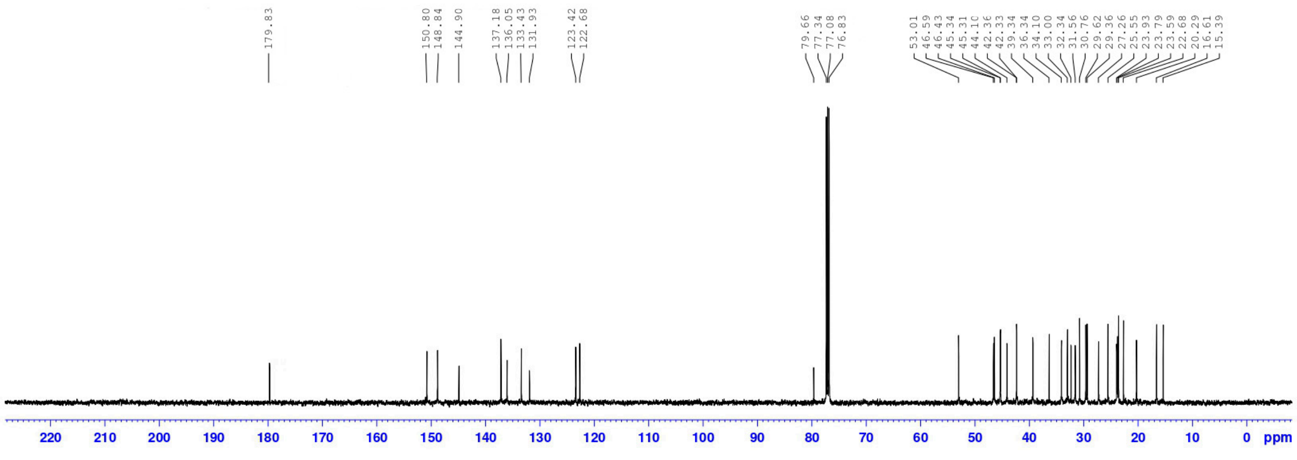

Figure S10. Spectrum NMR ^{13}C of compound **12**

Figure S11. Percentage of growth inhibition of compounds **13** against 60 individual cell lines

Developmental Therapeutics Program One Dose Mean Graph	NSC: D-806889 / 1	Conc: 1.00E-5 Molar	Test Date: Aug 27, 2018
	Experiment ID: 1808OS00		Report Date: Jan 01, 2019

Panel/Cell Line	Growth Percent
**Leukemia**	
CCRF-CEM	-6.27
HL-60(TB)	-37.64
K-562	6.99
MOLT-4	-16.90
RPMI-8226	-33.46
SR	-31.32
**Non-Small Cell Lung Cancer**	
A549/ATCC	15.20
EKVX	14.47
HOP-62	19.82
HOP-92	-6.70
NCI-H226	-20.45
NCI-H23	19.48
NCI-H322M	40.91
NCI-H460	-21.75
NCI-H522	-1.20
**Colon Cancer**	
COLO 205	-21.09
HCC-2998	-24.52
HCT-116	-32.58
HCT-15	7.19
HT29	5.31
KM12	6.58
SW-620	10.40
**CNS Cancer**	
SF-268	48.59
SF-295	25.41
SF-539	-7.46
SNB-19	47.48
SNB-75	42.99
U251	18.38
**Melanoma**	
LOX IMVI	-84.00
MALME-3M	-19.70
M14	-8.66
MDA-MB-435	6.81
SK-MEL-2	-6.74
SK-MEL-28	-14.41
SK-MEL-5	-63.45
UACC-257	14.82
UACC-62	2.88
**Ovarian Cancer**	
IGROV1	33.08
OVCAR-3	16.05
OVCAR-4	14.20
OVCAR-5	30.26
OVCAR-8	33.97
NCI/ADR-RES	26.18
SK-OV-3	28.89
**Renal Cancer**	
786-0	26.16
A498	-20.34
ACHN	15.59
CAKI-1	16.66
RXF 393	-36.76
SN12C	18.04
TK-10	35.94
UO-31	11.01
**Prostate Cancer**	
PC-3	10.93
DU-145	21.72
**Breast Cancer**	
MCF7	8.43
MDA-MB-231/ATCC	33.93
HS 578T	27.53
BT-549	-2.62
T-47D	20.16
MDA-MB-468	3.46
Mean	4.46
Delta	88.46
Range	132.59

Mean Growth Percent - Growth Percent

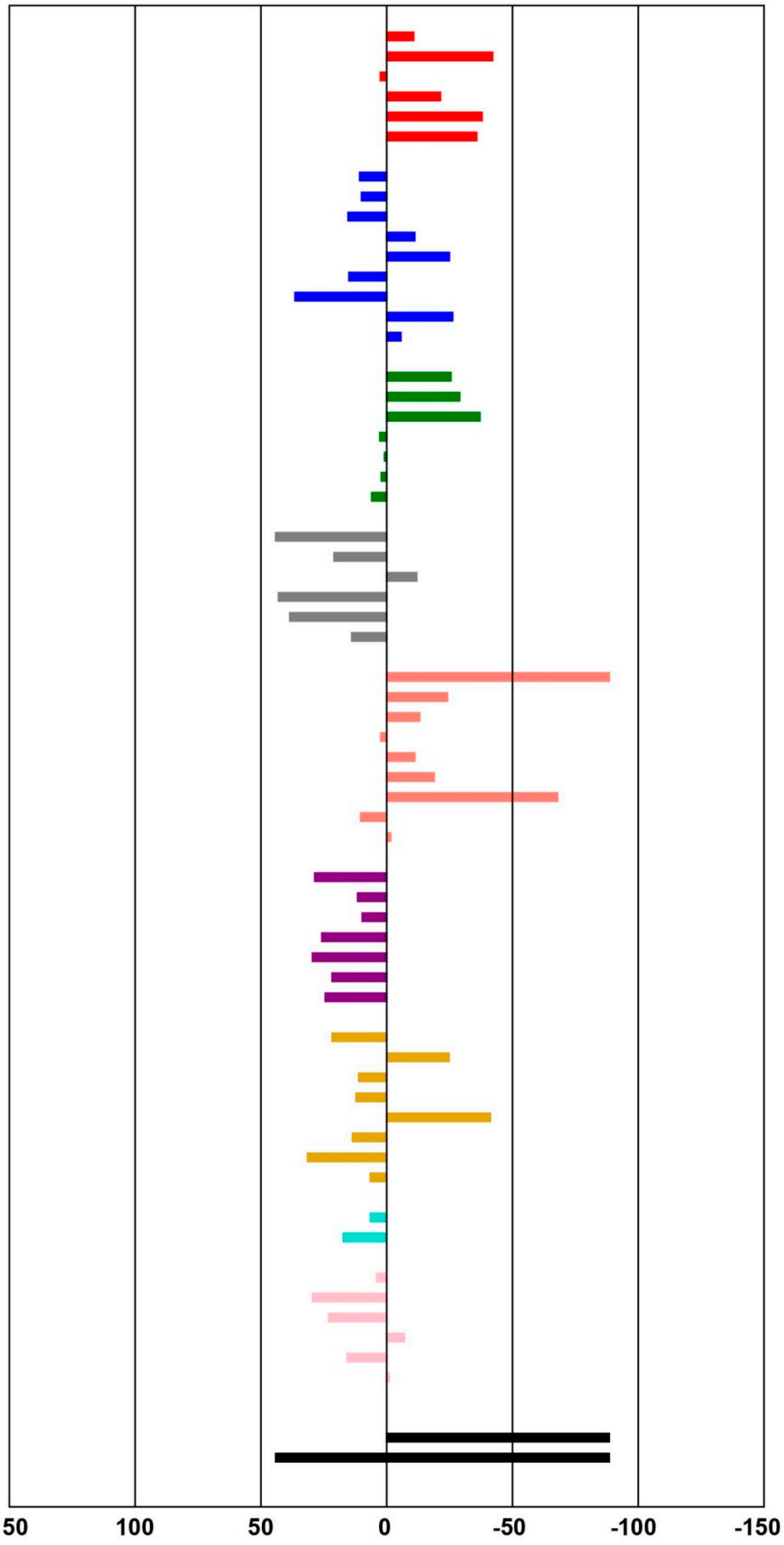

Figure S12. Cytostatic and cytotoxic effects of compound **13** (NSC 806889) on the NCI-60 panel of cancer cell lines at different doses, ranging from 10 nM to 100 μM. Results are displayed as dose-response curves (% growth versus sample concentration) for each cell line in the nine cancer subpanels.

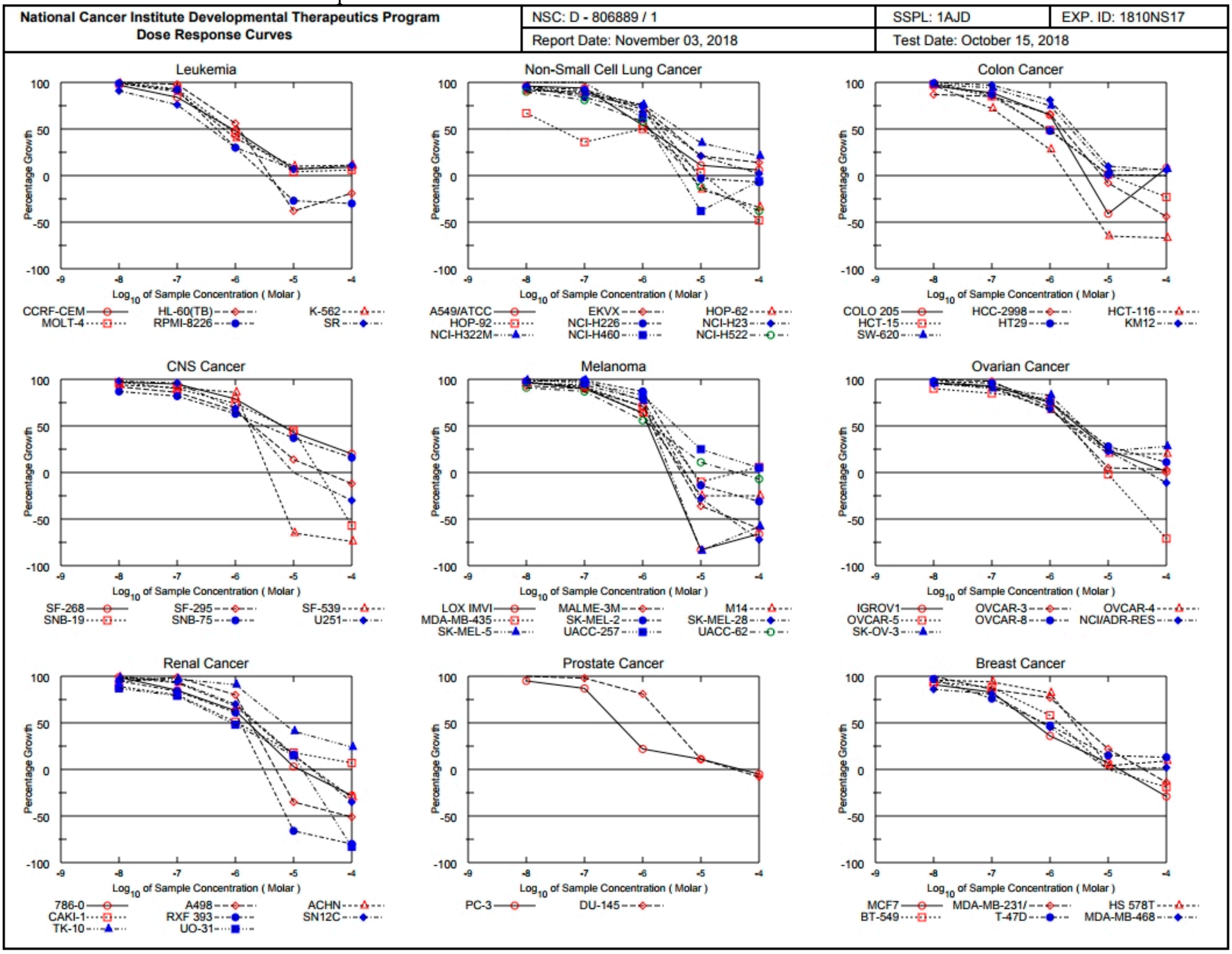

National Cancer Institute Developmental Therapeutics Program
In-Vitro Testing Results

NSC : D - 806889 / 1	Experiment ID : 1810NS17	Test Type : 08	Units : Molar
Report Date : November 03, 2018	Test Date : October 15, 2018	QNS :	MC :
COMI : 4-NK-OA-m	Stain Reagent : SRB Dual-Pass Related	SSPL : 1AJD	

			Log10 Concentration												
	Time		Mean Optical Densities					Percent Growth							
Panel/Cell Line	Zero	Ctrl	-8.0	-7.0	-6.0	-5.0	-4.0	-8.0	-7.0	-6.0	-5.0	-4.0	GI50	TGI	LC50
**Leukemia**															
CCRF-CEM	0.491	2.471	2.419	2.151	1.439	0.632	0.667	97	84	48	7	9	8.71E-7	> 1.00E-4	> 1.00E-4
HL-60(TB)	0.989	3.405	3.396	3.359	2.341	0.616	0.797	100	98	56	-38	-19	1.16E-6	3.96E-6	> 1.00E-4
K-562	0.225	2.748	2.733	2.506	1.239	0.469	0.512	99	90	40	10	11	6.37E-7	> 1.00E-4	> 1.00E-4
MOLT-4	0.659	3.224	3.223	3.040	1.814	0.762	0.807	100	93	45	4	6	7.87E-7	> 1.00E-4	> 1.00E-4
RPMI-8226	1.177	3.172	3.151	3.016	1.775	0.859	0.826	99	92	30	-27	-30	4.76E-7	3.36E-6	> 1.00E-4
SR	0.230	1.394	1.290	1.116	0.576	0.313	0.354	91	76	30	7	11	3.65E-7	> 1.00E-4	> 1.00E-4
**Non-Small Cell Lung Cancer**															
A549/ATCC	0.441	2.354	2.278	2.239	1.470	0.643	0.549	96	94	54	11	6	1.22E-6	> 1.00E-4	> 1.00E-4
EKVX	0.694	2.409	2.282	2.188	1.966	1.061	0.940	93	87	74	21	14	2.87E-6	> 1.00E-4	> 1.00E-4
HOP-62	0.503	1.834	1.718	1.696	1.450	0.429	0.331	91	90	71	-15	-34	1.76E-6	6.73E-6	> 1.00E-4
HOP-92	1.324	1.991	1.769	1.562	1.654	1.346	0.695	67	36	50	3	-48	3.47E-8	1.16E-5	> 1.00E-4
NCI-H226	1.012	2.019	1.967	1.937	1.765	0.985	0.944	95	92	75	-3	-7	2.09E-6	9.22E-6	> 1.00E-4
NCI-H23	0.529	1.901	1.830	1.688	1.457	0.823	0.561	95	84	68	21	2	2.41E-6	> 1.00E-4	> 1.00E-4
NCI-H322M	0.672	1.874	1.796	1.746	1.581	1.097	0.930	94	89	76	35	21	4.32E-6	> 1.00E-4	> 1.00E-4
NCI-H460	0.297	2.952	3.029	2.983	1.943	0.184	0.281	103	101	62	-38	-6	1.32E-6	4.17E-6	> 1.00E-4
NCI-H522	0.826	2.452	2.294	2.137	1.768	0.726	0.513	90	81	58	-12	-38	1.30E-6	6.72E-6	> 1.00E-4
**Colon Cancer**															
COLO 205	0.316	1.880	1.815	1.706	1.327	0.188	0.440	96	89	65	-41	8	1.38E-6	.	> 1.00E-4
HCC-2998	0.867	2.518	2.305	2.271	1.964	0.798	0.486	87	85	66	-8	-44	1.66E-6	7.82E-6	> 1.00E-4
HCT-116	0.205	2.425	2.355	1.811	0.826	0.073	0.067	97	72	28	-65	-67	3.19E-7	2.00E-6	6.95E-6
HCT-15	0.390	2.693	2.652	2.355	1.519	0.413	0.299	98	85	49	1	-23	9.39E-7	1.10E-5	> 1.00E-4
HT29	0.312	2.400	2.387	2.129	1.324	0.340	0.311	99	87	48	1	.	9.12E-7	5.45E-5	> 1.00E-4
KM12	0.591	3.020	3.009	2.959	2.548	0.830	0.747	100	97	81	10	6	2.70E-6	> 1.00E-4	> 1.00E-4
SW-620	0.271	2.051	2.008	1.953	1.608	0.366	0.402	98	94	75	5	7	2.29E-6	> 1.00E-4	> 1.00E-4
**CNS Cancer**															
SF-268	0.963	2.707	2.651	2.622	2.334	1.711	1.313	97	95	79	43	20	6.33E-6	> 1.00E-4	> 1.00E-4
SF-295	0.629	2.540	2.393	2.272	1.888	0.902	0.553	92	86	66	14	-12	2.03E-6	3.48E-5	> 1.00E-4
SF-539	0.833	2.615	2.514	2.453	2.361	0.291	0.219	94	91	86	-65	-74	1.73E-6	3.70E-6	7.94E-6
SNB-19	0.834	2.604	2.546	2.429	2.149	1.638	0.358	97	90	74	45	-57	6.93E-6	2.77E-5	8.53E-5
SNB-75	0.681	1.318	1.235	1.201	1.083	0.918	0.785	87	82	63	37	16	3.21E-6	> 1.00E-4	> 1.00E-4
U251	0.406	2.321	2.277	2.241	1.725	0.414	0.285	98	96	69	.	-30	1.89E-6	1.03E-5	> 1.00E-4
**Melanoma**															
LOX IMVI	0.403	2.601	2.529	2.387	1.807	0.068	0.137	97	90	64	-83	-66	1.24E-6	2.72E-6	5.95E-6
MALME-3M	0.589	1.394	1.341	1.318	1.160	0.376	0.237	93	90	71	-36	-60	1.57E-6	4.59E-6	3.84E-5
M14	0.397	1.981	1.917	1.861	1.396	0.299	0.297	96	92	63	-25	-25	1.41E-6	5.23E-6	> 1.00E-4
MDA-MB-435	0.380	1.806	1.759	1.699	1.392	0.344	0.463	97	92	71	-10	6	1.82E-6	.	> 1.00E-4
SK-MEL-2	1.335	3.119	3.088	3.097	2.889	1.149	0.926	98	99	87	-14	-31	2.33E-6	7.27E-6	> 1.00E-4
SK-MEL-28	0.721	1.940	1.887	1.866	1.675	0.516	0.201	96	94	78	-28	-72	1.84E-6	5.41E-6	3.12E-5
SK-MEL-5	0.954	3.395	3.377	3.378	2.841	0.153	0.398	99	99	77	-84	-58	1.48E-6	3.01E-6	6.15E-6
UACC-257	0.715	2.118	2.124	2.062	1.879	1.061	0.779	100	96	83	25	5	3.68E-6	> 1.00E-4	> 1.00E-4
UACC-62	0.917	2.881	2.709	2.620	2.017	1.132	0.854	91	87	56	11	-7	1.36E-6	4.09E-5	> 1.00E-4
**Ovarian Cancer**															
IGROV1	0.409	1.855	1.792	1.759	1.492	0.758	0.425	96	93	75	24	1	3.09E-6	> 1.00E-4	> 1.00E-4
OVCAR-3	0.486	1.843	1.839	1.823	1.442	0.560	0.531	100	98	70	5	3	2.06E-6	> 1.00E-4	> 1.00E-4
OVCAR-4	0.588	1.392	1.388	1.316	1.130	0.751	0.751	100	91	67	20	20	2.34E-6	> 1.00E-4	> 1.00E-4
OVCAR-5	0.578	1.590	1.487	1.435	1.375	0.567	0.167	90	85	79	-2	-71	2.27E-6	9.45E-6	4.95E-5
OVCAR-8	0.347	1.915	1.882	1.854	1.543	0.790	0.527	98	96	76	28	11	3.52E-6	> 1.00E-4	> 1.00E-4
NCI/ADR-RES	0.571	2.090	2.020	1.966	1.602	0.919	0.506	95	92	68	23	-11	2.49E-6	4.64E-5	> 1.00E-4
SK-OV-3	0.722	2.066	2.011	1.937	1.842	1.033	1.102	96	90	83	23	28	3.58E-6	> 1.00E-4	> 1.00E-4
**Renal Cancer**															
786-0	0.806	2.943	2.912	2.631	2.158	0.868	0.581	99	85	63	3	-28	1.66E-6	1.24E-5	> 1.00E-4
A498	1.765	2.751	2.689	2.736	2.558	1.143	0.860	94	99	80	-35	-51	1.83E-6	4.96E-6	8.30E-5
ACHN	0.398	1.852	1.839	1.756	1.390	0.618	0.280	99	93	68	15	-30	2.20E-6	2.17E-5	> 1.00E-4
CAKI-1	0.654	2.518	2.311	2.144	1.605	0.996	0.787	89	80	51	18	7	1.07E-6	> 1.00E-4	> 1.00E-4
RXF 393	1.136	1.994	1.950	1.859	1.658	0.391	0.224	95	84	61	-66	-80	1.22E-6	3.03E-6	7.53E-6
SN12C	0.645	2.469	2.469	2.357	1.929	0.936	0.420	100	94	70	16	-35	2.37E-6	2.06E-5	> 1.00E-4
TK-10	0.731	2.191	2.164	2.149	2.054	1.323	1.077	98	97	91	41	24	6.46E-6	> 1.00E-4	> 1.00E-4
UO-31	0.562	1.861	1.694	1.593	1.192	0.757	0.095	87	79	48	15	-83	8.93E-7	1.42E-5	4.59E-5
**Prostate Cancer**															
PC-3	0.607	2.689	2.588	2.424	1.073	0.827	0.575	95	87	22	11	-5	3.75E-7	4.61E-5	> 1.00E-4
DU-145	0.295	1.348	1.362	1.331	1.150	0.407	0.271	101	98	81	11	-8	2.77E-6	3.64E-5	> 1.00E-4
**Breast Cancer**															
MCF7	0.366	2.265	2.100	1.948	1.053	0.491	0.259	91	83	36	7	-29	5.09E-7	1.53E-5	> 1.00E-4
MDA-MB-231/ATCC	0.631	1.695	1.717	1.548	1.447	0.863	0.545	102	86	77	22	-14	3.06E-6	4.11E-5	> 1.00E-4
HS 578T	0.947	2.120	2.074	2.048	1.905	0.995	1.053	96	94	82	4	9	2.56E-6	> 1.00E-4	> 1.00E-4
BT-549	1.013	2.387	2.305	2.216	1.814	1.014	0.817	94	88	58	.	-19	1.39E-6	1.00E-5	> 1.00E-4
T-47D	0.707	1.726	1.699	1.476	1.185	0.863	0.842	97	76	47	15	13	7.81E-7	> 1.00E-4	> 1.00E-4
MDA-MB-468	0.927	1.853	1.723	1.678	1.340	0.929	0.944	86	81	45	.	2	7.12E-7	> 1.00E-4	> 1.00E-4

Figure S13. GI50 (50% Growth Inhibition), TGI (Total Growth Inhibition) and LC50 (50% Lethal Concentration) mean graphs obtained for compound **13** (NSC 806889) tested at five concentrations (0.01, 0.1, 1, 10, 100 μM) against the NCI-60 human cancer cell lines.

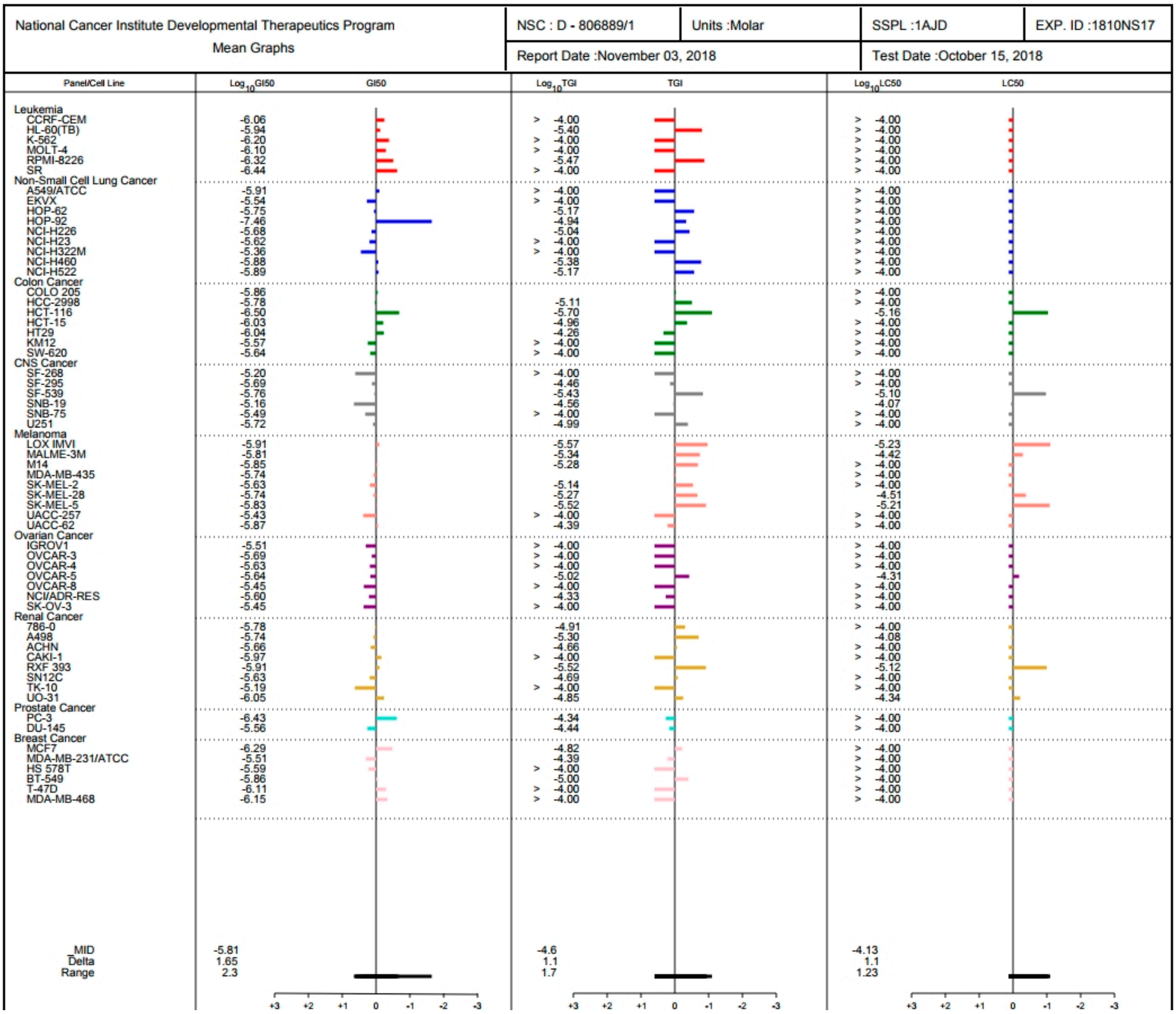

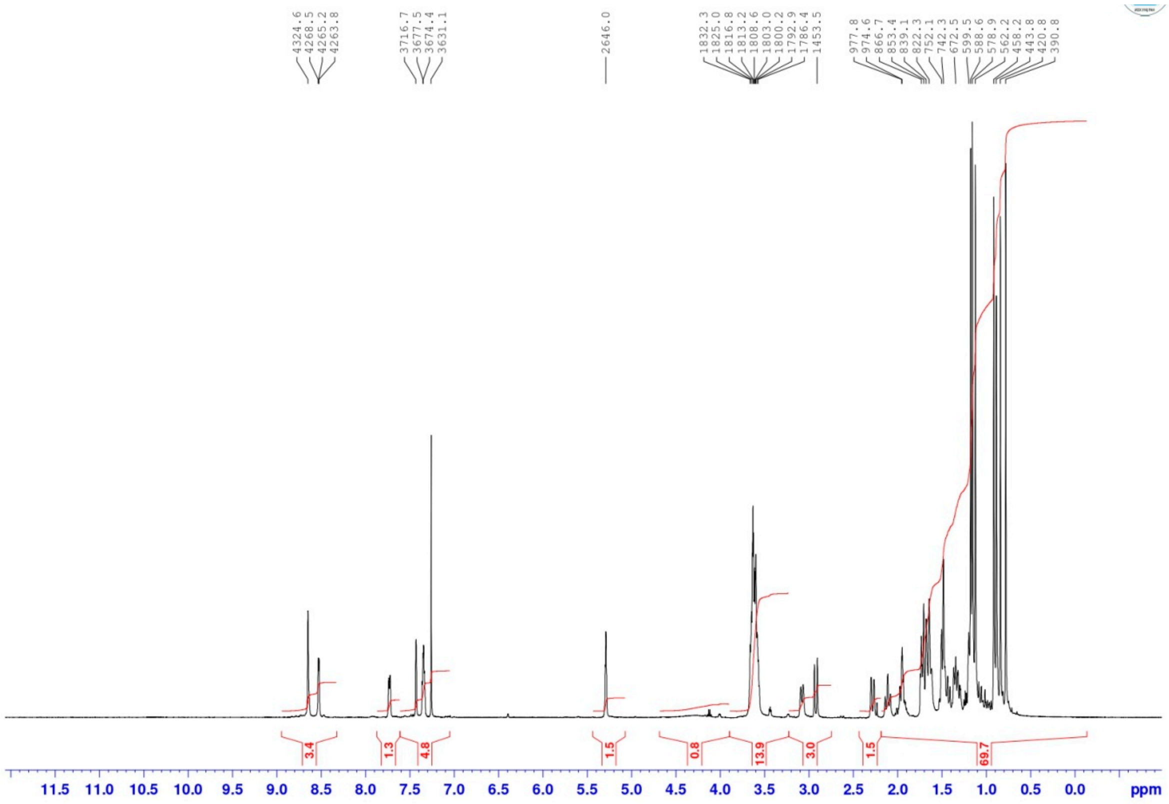

Figure S14. Spectrum NMR ^{1}H of compound **13**

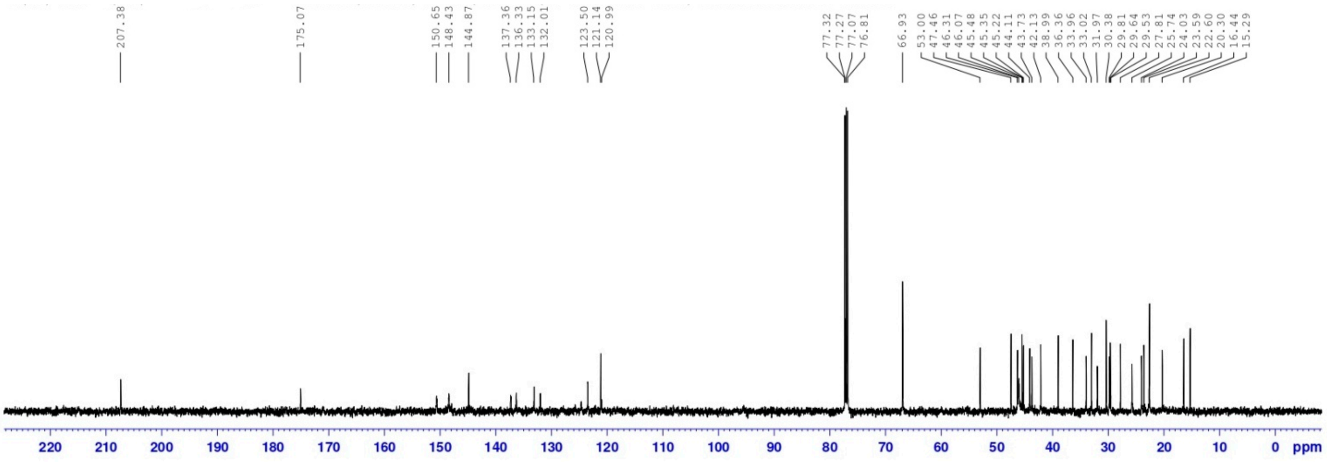

Figure S15. Spectrum NMR ^{13}C of compound **13**

Figure S16. Percentage of growth inhibition of compounds **27** against 60 individual cell lines

**Developmental Therapeutics Program**	**NSC:** D-804712 / 1	**Conc:** 1.00E-4 Molar	**Test Date:** Apr 30, 2018
**One Dose Mean Graph**	**Experiment ID:** 1804OS61		**Report Date:** Mar 24, 2020

Panel/Cell Line	Growth Percent
Leukemia	
CCRF-CEM	-26.43
HL-60(TB)	-3.02
K-562	7.11
MOLT-4	-10.76
RPMI-8226	-19.29
SR	31.21
Non-Small Cell Lung Cancer	
A549/ATCC	-52.50
EKVX	-68.38
HOP-62	-75.65
HOP-92	-87.70
NCI-H226	-52.26
NCI-H23	-81.14
NCI-H322M	-73.42
NCI-H460	-55.64
NCI-H522	-82.75
Colon Cancer	
COLO 205	-60.86
HCC-2998	-81.38
HCT-116	-72.97
HCT-15	-71.18
HT29	-67.91
KM12	-73.47
SW-620	-44.87
CNS Cancer	
SF-268	-58.68
SF-295	-79.37
SF-539	-80.08
SNB-19	-52.62
SNB-75	-55.90
U251	-64.53
Melanoma	
LOX IMVI	-91.68
MALME-3M	-85.97
M14	-43.60
MDA-MB-435	-85.11
SK-MEL-2	-64.03
SK-MEL-28	-79.25
SK-MEL-5	-91.68
UACC-257	-39.11
UACC-62	-94.42
Ovarian Cancer	
IGROV1	-59.55
OVCAR-3	-74.54
OVCAR-5	-48.80
OVCAR-8	-18.97
NCI/ADR-RES	-58.89
SK-OV-3	-17.34
Renal Cancer	
786-0	-40.32
A498	-56.23
ACHN	-97.88
CAKI-1	-74.62
RXF 393	-84.03
SN12C	-56.00
TK-10	-73.40
UO-31	-99.47
Prostate Cancer	
PC-3	-67.12
DU-145	-73.15
Breast Cancer	
MCF7	-74.67
MDA-MB-231/ATCC	-81.50
HS 578T	-13.88
BT-549	-62.50
T-47D	-14.27
MDA-MB-468	-38.00
Mean	-58.89
Delta	40.58
Range	130.68

Mean Growth Percent - Growth Percent

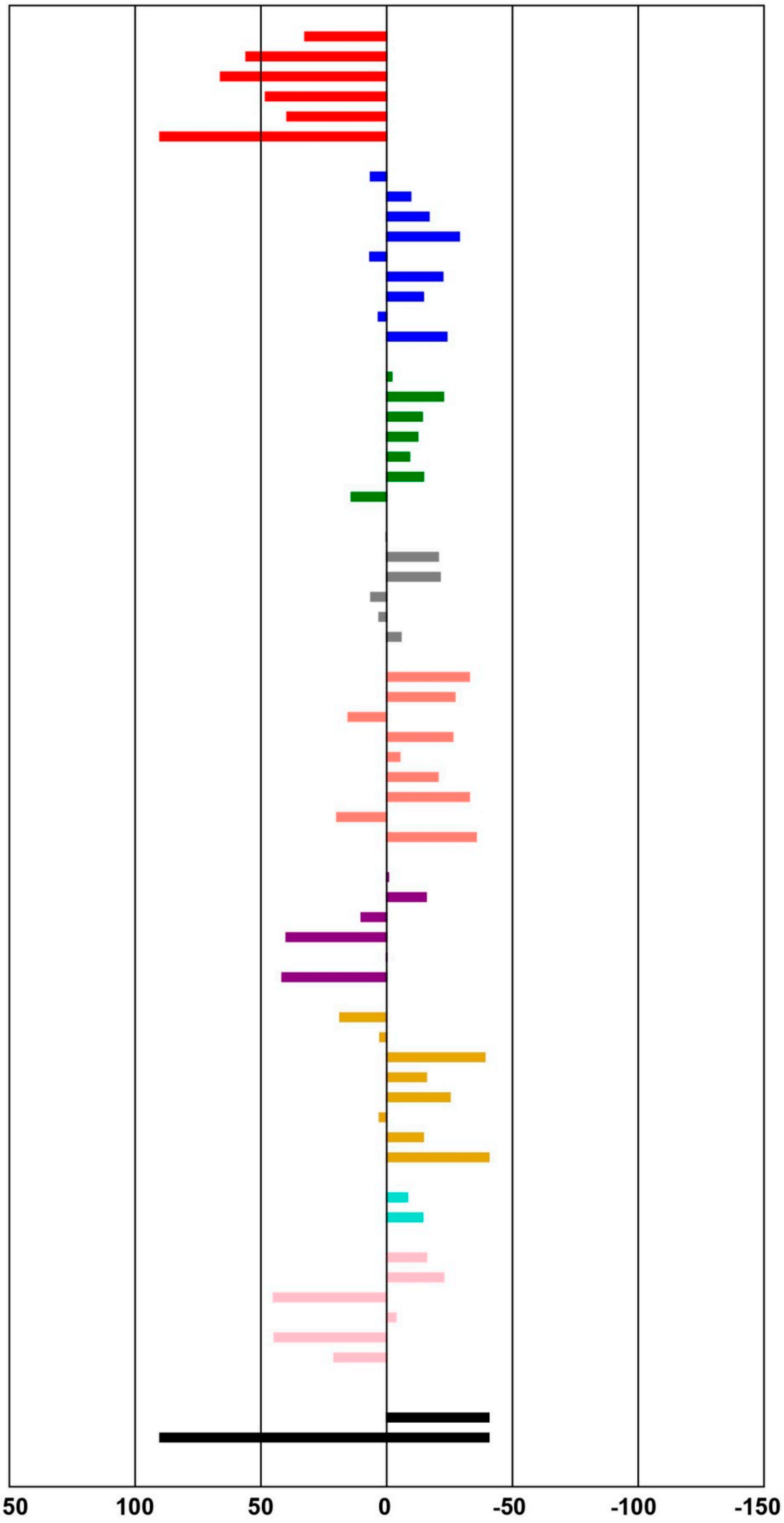

Figure S17. Cytostatic and cytotoxic effects of compound **27** (NSC 804712) on the NCI-60 panel of cancer cell lines at different doses, ranging from 10 nM to 100 μM. Results are displayed as dose-response curves (% growth versus sample concentration) for each cell line in the nine cancer subpanels.

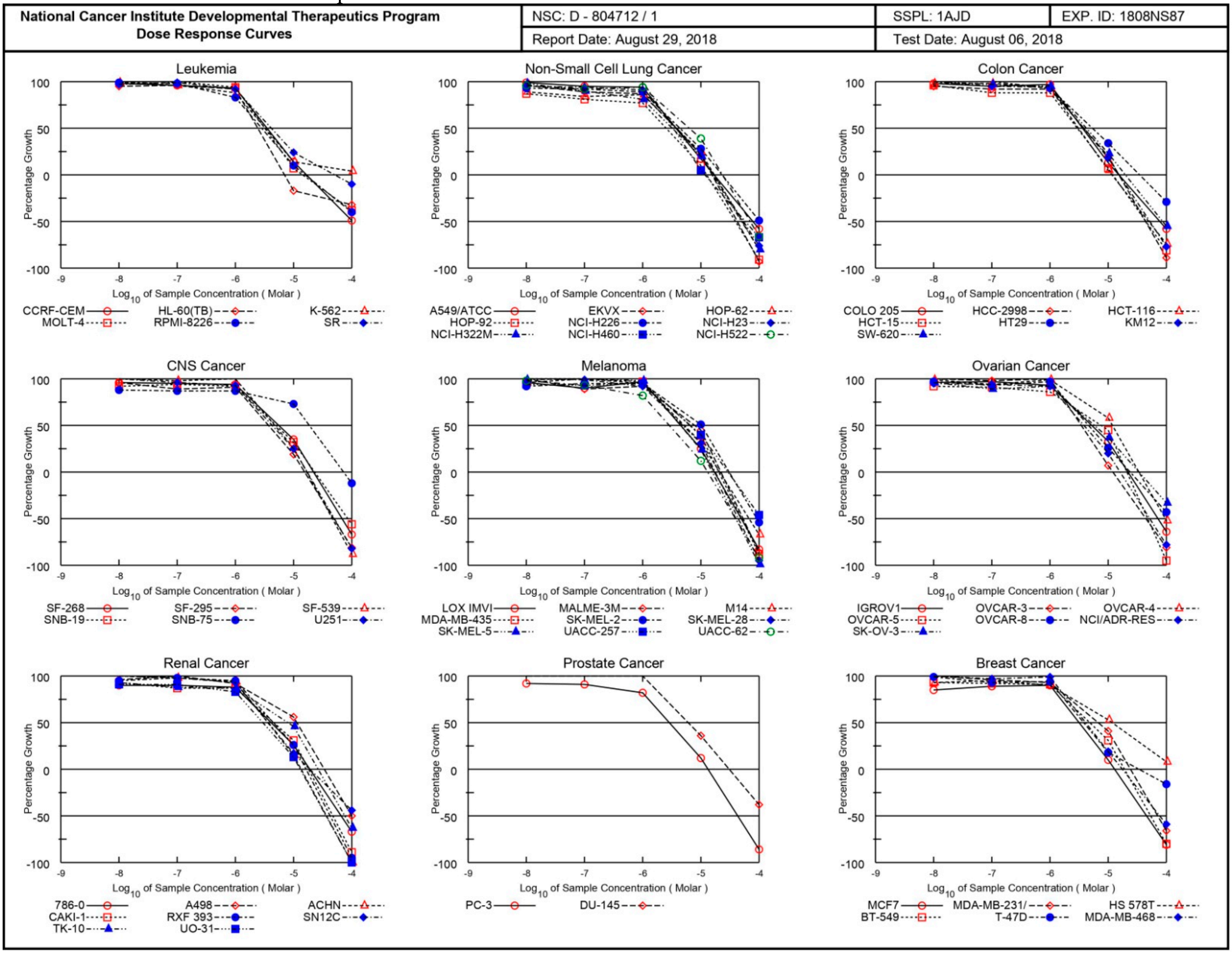

National Cancer Institute Developmental Therapeutics Program
In-Vitro Testing Results

NSC : D - 804712 / 1	Experiment ID : 1808NS87	Test Type : 08	Units : Molar
Report Date : August 29, 2018	Test Date : August 06, 2018	QNS :	MC :
COMI : UA-fur	Stain Reagent : SRB Dual-Pass Related	SSPL : 1AJD	

			Log10 Concentration												
	Time		Mean Optical Densities					Percent Growth							
Panel/Cell Line	Zero	Ctrl	-8.0	-7.0	-6.0	-5.0	-4.0	-8.0	-7.0	-6.0	-5.0	-4.0	GI50	TGI	LC50
Leukemia															
CCRF-CEM	0.546	2.752	2.711	2.664	2.604	0.858	0.280	98	96	93	14	-49	3.52E-6	1.68E-5	> 1.00E-4
HL-60(TB)	0.909	3.174	3.054	3.077	2.997	0.759	0.621	95	96	92	-17	-32	2.44E-6	7.04E-6	> 1.00E-4
K-562	0.184	1.981	1.958	1.911	1.773	0.427	0.255	99	96	88	14	4	3.26E-6	> 1.00E-4	> 1.00E-4
MOLT-4	0.571	2.684	2.718	2.680	2.548	0.709	0.352	102	100	94	7	-38	3.17E-6	1.40E-5	> 1.00E-4
RPMI-8226	1.200	3.076	3.067	3.036	2.759	1.383	0.725	99	98	83	10	-40	2.83E-6	1.57E-5	> 1.00E-4
SR	0.279	1.414	1.376	1.403	1.325	0.548	0.252	97	99	92	24	-10	4.12E-6	5.08E-5	> 1.00E-4
Non-Small Cell Lung Cancer															
A549/ATCC	0.550	2.884	2.855	2.759	2.745	0.938	0.234	99	95	94	17	-58	3.71E-6	1.68E-5	7.91E-5
EKVX	0.967	2.645	2.578	2.459	2.430	1.331	0.068	96	89	87	22	-93	3.69E-6	1.55E-5	4.22E-5
HOP-62	0.589	2.089	1.928	1.849	1.877	0.947	0.116	89	84	86	24	-80	3.78E-6	1.69E-5	5.12E-5
HOP-92	1.052	1.771	1.680	1.634	1.609	1.121	0.093	87	81	77	10	-91	2.54E-6	1.25E-5	3.90E-5
NCI-H226	0.715	2.188	2.083	2.069	2.020	1.132	0.364	93	92	89	28	-49	4.36E-6	2.32E-5	> 1.00E-4
NCI-H23	0.567	2.026	1.954	1.934	1.901	0.869	0.135	95	94	91	21	-76	3.85E-6	1.63E-5	5.36E-5
NCI-H322M	0.770	2.183	2.148	2.039	1.913	1.059	0.154	98	90	81	20	-80	3.24E-6	1.60E-5	5.02E-5
NCI-H460	0.103	2.159	2.208	2.167	2.159	0.213	0.035	102	100	100	5	-67	3.37E-6	1.19E-5	5.89E-5
NCI-H522	0.984	2.762	2.688	2.618	2.653	1.682	0.335	96	92	94	39	-66	6.35E-6	2.36E-5	7.05E-5
Colon Cancer															
COLO 205	0.485	2.350	2.320	2.256	2.288	0.723	0.204	98	95	97	13	-58	3.60E-6	1.51E-5	7.70E-5
HCC-2998	0.509	1.798	1.739	1.697	1.689	0.772	0.054	95	92	92	20	-89	3.84E-6	1.53E-5	4.37E-5
HCT-116	0.172	2.091	2.056	2.048	1.958	0.274	0.044	98	98	93	5	-74	3.10E-6	1.17E-5	4.94E-5
HCT-15	0.270	2.002	1.937	1.798	1.792	0.396	0.051	96	88	88	7	-81	2.95E-6	1.21E-5	4.43E-5
HT29	0.150	1.477	1.519	1.491	1.378	0.599	0.107	103	101	93	34	-29	5.30E-6	3.46E-5	> 1.00E-4
KM12	0.419	2.339	2.330	2.243	2.227	0.771	0.097	100	95	94	18	-77	3.82E-6	1.56E-5	5.22E-5
SW-620	0.173	2.397	2.394	2.322	2.288	0.676	0.078	100	97	95	23	-55	4.19E-6	1.95E-5	8.57E-5
CNS Cancer															
SF-268	0.672	2.126	2.064	2.051	2.035	1.185	0.220	96	95	94	35	-67	5.59E-6	2.21E-5	6.79E-5
SF-295	0.914	3.033	2.948	2.807	2.850	1.316	0.174	96	89	91	19	-81	3.72E-6	1.55E-5	4.89E-5
SF-539	0.940	2.397	2.432	2.374	2.390	1.313	0.112	102	98	100	26	-88	4.67E-6	1.68E-5	4.62E-5
SNB-19	0.727	2.519	2.384	2.406	2.383	1.305	0.320	92	94	92	32	-56	5.07E-6	2.32E-5	8.54E-5
SNB-75	0.453	1.559	1.422	1.414	1.410	1.260	0.398	88	87	87	73	-12	1.86E-5	7.18E-5	> 1.00E-4
U251	0.560	2.493	2.496	2.416	2.365	1.036	0.101	100	96	93	25	-82	4.27E-6	1.70E-5	5.01E-5
Melanoma															
LOX IMVI	0.303	2.262	2.178	2.063	2.213	0.792	0.051	96	90	97	25	-83	4.51E-6	1.70E-5	4.93E-5
MALME-3M	0.390	1.013	1.013	0.947	0.966	0.673	0.056	100	89	92	45	-86	8.01E-6	2.22E-5	5.34E-5
M14	0.418	2.094	2.031	2.102	2.018	0.963	0.137	96	100	95	32	-67	5.27E-6	2.12E-5	6.72E-5
MDA-MB-435	0.360	2.594	2.592	2.603	2.536	1.241	0.043	100	100	97	39	-88	6.58E-6	2.04E-5	5.03E-5
SK-MEL-2	0.861	2.284	2.164	2.216	2.212	1.580	0.395	92	95	95	51	-54	1.01E-5	3.04E-5	9.13E-5
SK-MEL-28	0.798	2.227	2.289	2.218	2.108	1.235	0.042	104	99	92	31	-95	4.81E-6	1.75E-5	4.40E-5
SK-MEL-5	1.043	3.222	3.206	3.217	3.183	1.561	0.008	99	100	98	24	-99	4.44E-6	1.56E-5	3.98E-5
UACC-257	1.108	2.641	2.542	2.534	2.565	1.715	0.600	94	93	95	40	-46	6.49E-6	2.91E-5	> 1.00E-4
UACC-62	0.891	2.961	2.930	2.812	2.592	1.135	0.072	98	93	82	12	-92	2.86E-6	1.30E-5	3.94E-5
Ovarian Cancer															
IGROV1	0.550	2.204	2.144	2.148	2.090	1.093	0.196	96	97	93	33	-64	5.19E-6	2.17E-5	7.10E-5
OVCAR-3	0.435	1.512	1.557	1.493	1.515	0.506	0.084	104	98	100	7	-81	3.44E-6	1.19E-5	4.45E-5
OVCAR-4	0.196	0.841	0.832	0.805	0.832	0.570	0.095	99	94	99	58	-52	1.18E-5	3.38E-5	9.68E-5
OVCAR-5	0.553	1.310	1.251	1.240	1.208	0.894	0.028	92	91	86	45	-95	7.59E-6	2.10E-5	4.78E-5
OVCAR-8	0.559	2.608	2.548	2.464	2.551	1.097	0.321	97	93	97	26	-43	4.63E-6	2.41E-5	> 1.00E-4
NCI/ADR-RES	0.631	2.216	2.145	2.119	2.084	0.954	0.136	95	94	92	20	-78	3.84E-6	1.61E-5	5.15E-5
SK-OV-3	0.941	2.171	2.118	2.036	2.067	1.400	0.631	96	89	92	37	-33	5.83E-6	3.39E-5	> 1.00E-4
Renal Cancer															
786-0	0.509	2.423	2.240	2.234	2.184	1.010	0.169	90	90	88	26	-67	4.09E-6	1.91E-5	6.60E-5
A498	0.817	1.902	1.854	1.912	1.811	1.430	0.406	96	101	92	56	-50	1.15E-5	3.38E-5	9.93E-5
ACHN	0.375	1.584	1.603	1.565	1.512	0.555	-0.002	102	98	94	15	-100	3.60E-6	1.35E-5	3.67E-5
CAKI-1	0.173	1.559	1.461	1.376	1.382	0.598	0.019	93	87	87	31	-89	4.55E-6	1.80E-5	4.72E-5
RXF 393	1.038	2.022	1.977	2.000	1.971	1.292	0.048	95	98	95	26	-95	4.46E-6	1.63E-5	4.22E-5
SN12C	0.613	2.584	2.606	2.549	2.456	0.948	0.341	101	98	93	17	-44	3.70E-6	1.89E-5	> 1.00E-4
TK-10	0.579	1.706	1.629	1.568	1.564	1.095	0.213	93	88	87	46	-63	7.90E-6	2.63E-5	7.55E-5
UO-31	0.729	1.937	1.825	1.827	1.726	0.887	0.002	91	91	83	13	-100	2.94E-6	1.31E-5	3.62E-5
Prostate Cancer															
PC-3	0.514	2.095	1.969	1.948	1.816	0.705	0.073	92	91	82	12	-86	2.89E-6	1.33E-5	4.30E-5
DU-145	0.292	1.335	1.410	1.375	1.410	0.671	0.182	107	104	107	36	-38	6.41E-6	3.09E-5	> 1.00E-4
Breast Cancer															
MCF7	0.304	1.951	1.705	1.771	1.783	0.473	0.058	85	89	90	10	-81	3.17E-6	1.30E-5	4.58E-5
MDA-MB-231/ATCC	0.601	1.579	1.608	1.539	1.512	1.002	0.202	103	96	93	41	-66	6.72E-6	2.41E-5	7.03E-5
HS 578T	0.587	2.026	1.923	1.947	1.886	1.349	0.706	93	95	90	53	8	1.16E-5	> 1.00E-4	> 1.00E-4
BT-549	0.991	2.223	2.141	2.123	2.107	1.369	0.198	93	92	91	31	-80	4.76E-6	1.89E-5	5.36E-5
T-47D	0.641	1.482	1.471	1.425	1.435	0.790	0.540	99	93	94	18	-16	3.79E-6	3.38E-5	> 1.00E-4
MDA-MB-468	0.807	2.110	2.106	2.066	2.097	1.050	0.335	100	97	99	19	-59	4.07E-6	1.74E-5	7.75E-5

Figure S18. GI50 (50% Growth Inhibition), TGI (Total Growth Inhibition) and LC50 (50% Lethal Concentration) mean graphs obtained for compound **27** (NSC 804712) tested at five concentrations (0.01, 0.1, 1, 10, 100 μM) against the NCI-60 human cancer cell lines.

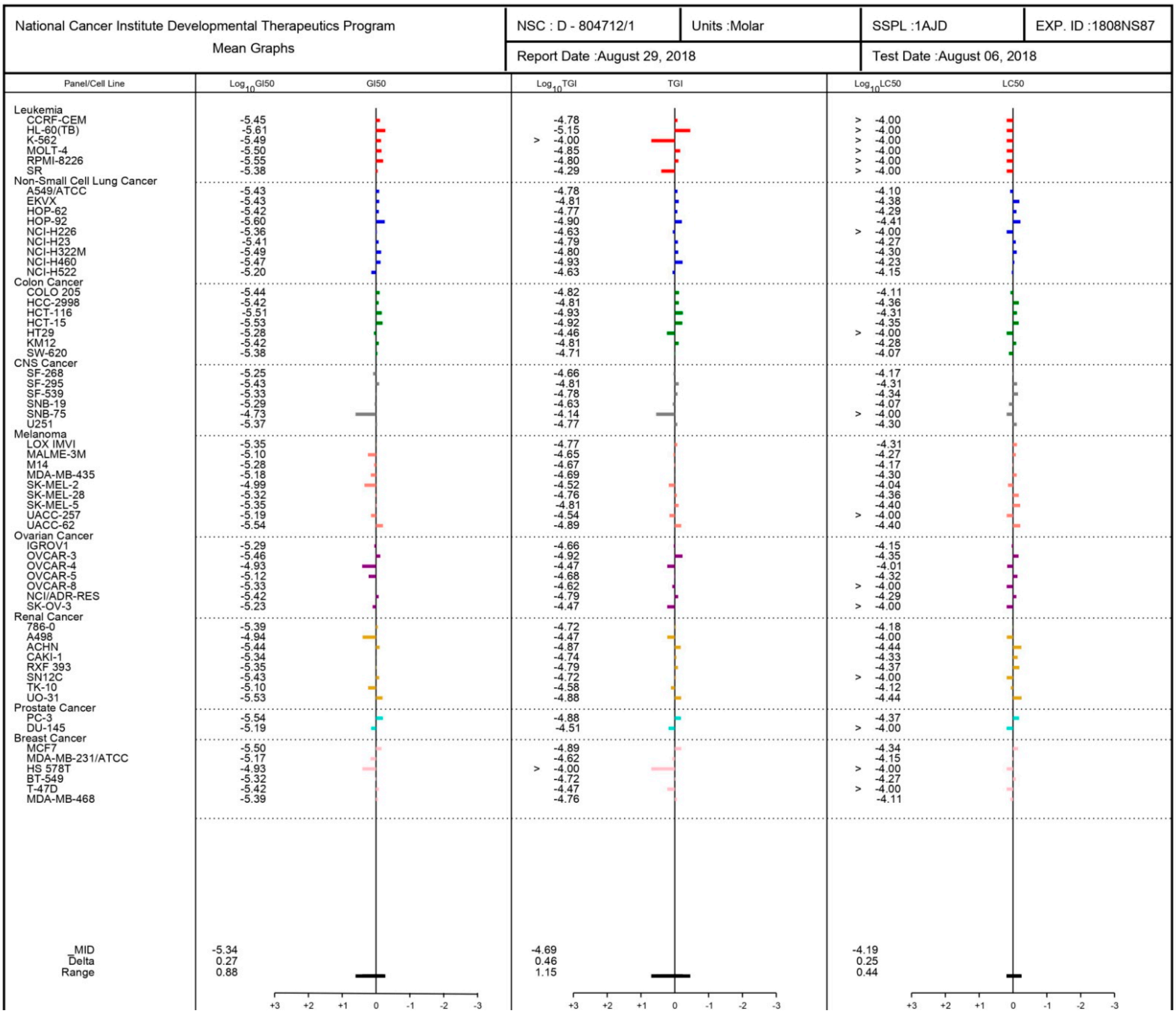

National Cancer Institute Developmental Therapeutics Program<br>Dose Response Curves	NSC : D - 804712/1	SSPL:1AJD	EXP. ID:1808NS87
	Report Date:August 29, 2018	Test Date:August 06, 2018	

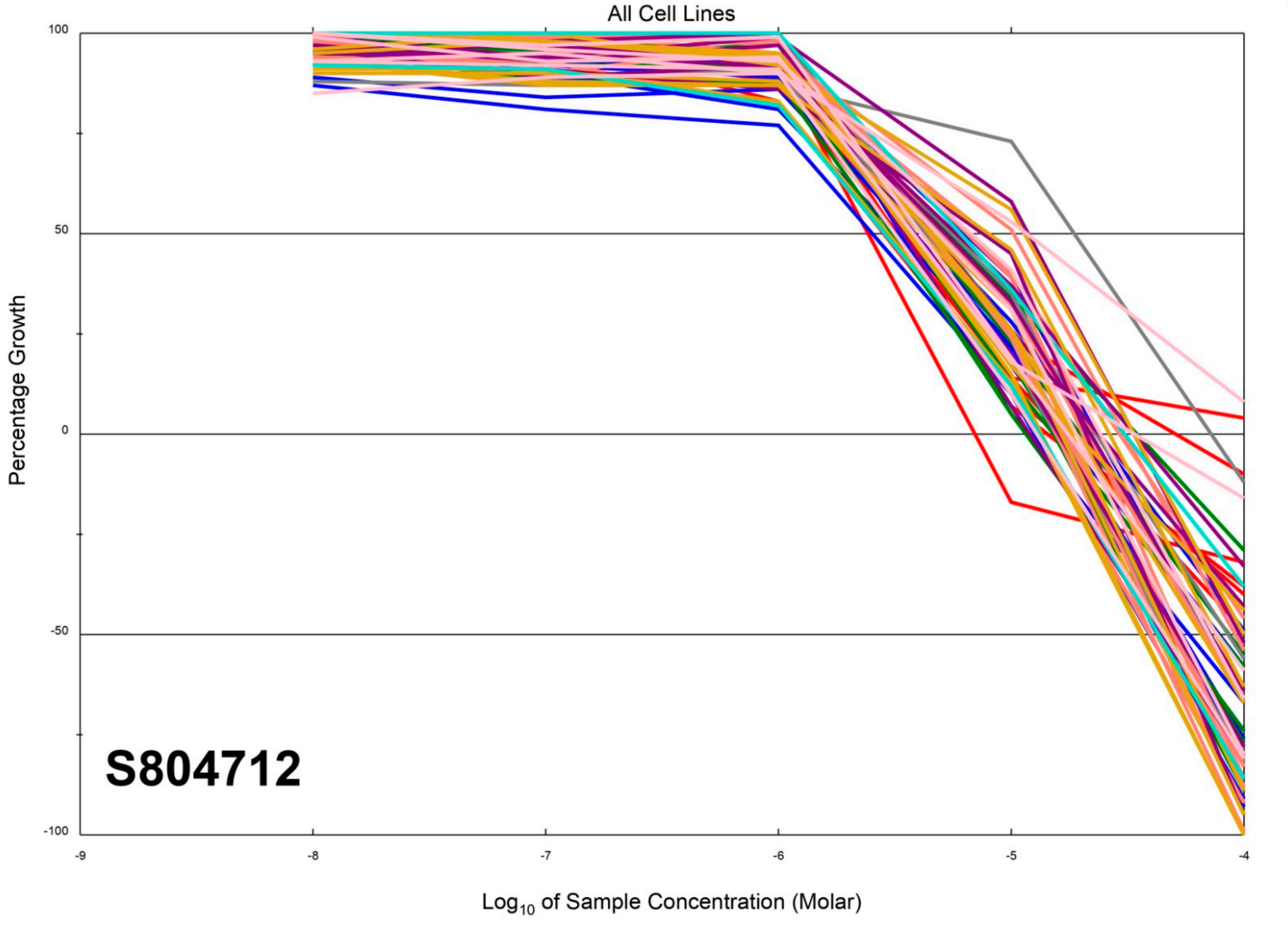

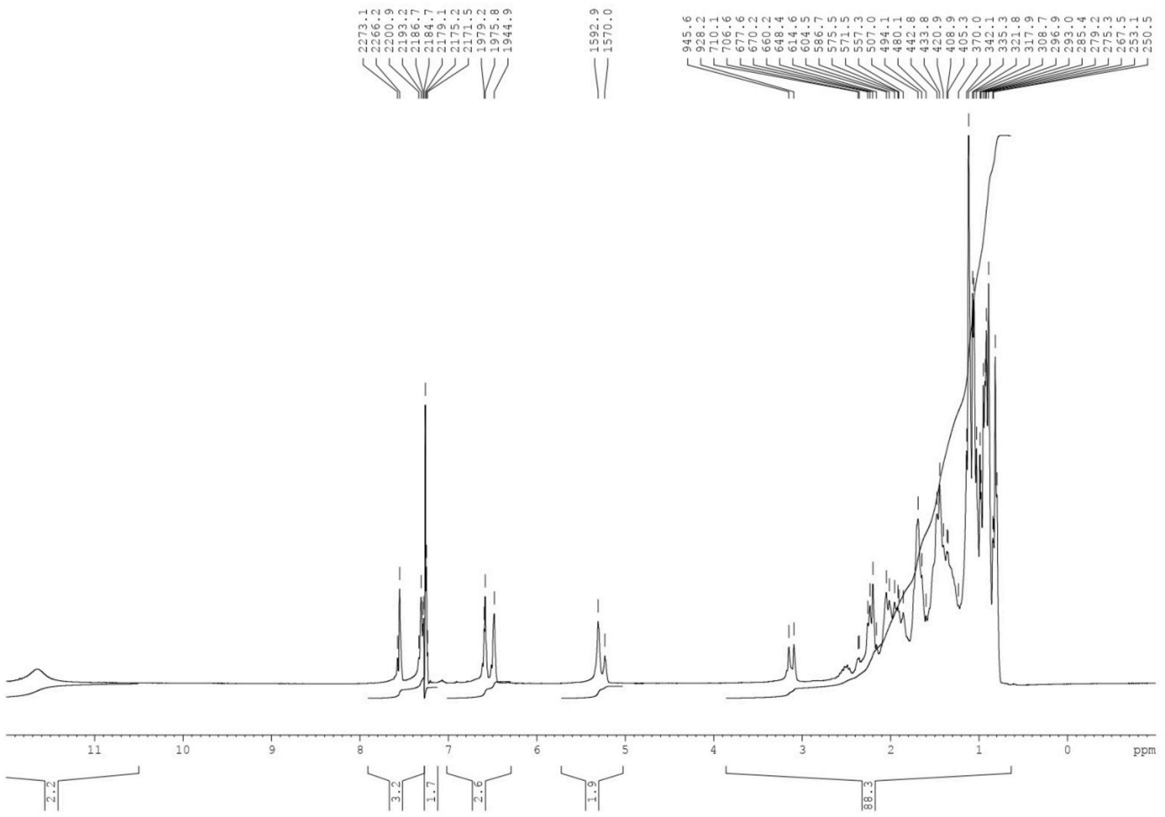

Figure S19. Spectrum NMR ^{1}H of compound **27**

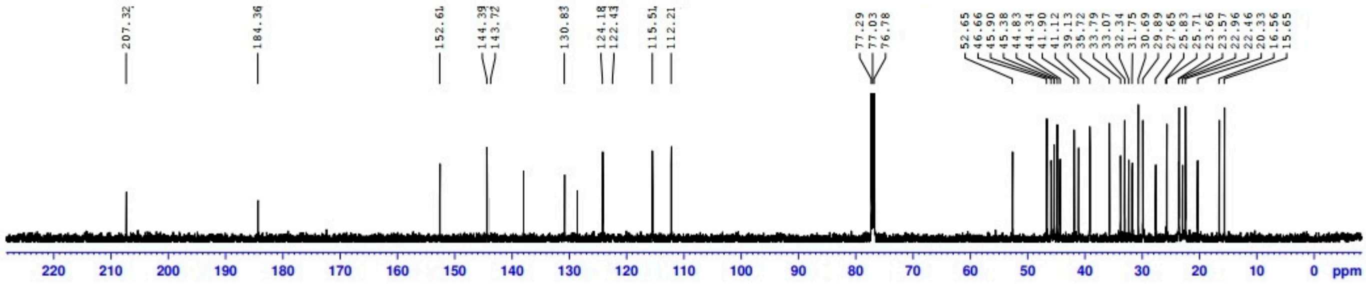

Figure S20. Spectrum NMR ^{13}C of compound **27**

Figure S21. Percentage of growth inhibition of compounds **29** against 60 individual cell lines

Developmental Therapeutics Program
One Dose Mean Graph

NSC: D-801984 / 1	Conc: 1.00E-5 Molar	Test Date: Nov 27, 2017
Experiment ID: 1711OS06		Report Date: Dec 22, 2017

Panel/Cell Line	Growth Percent
Leukemia	
CCRF-CEM	-51.66
HL-60(TB)	-54.38
K-562	-46.27
MOLT-4	-51.92
RPMI-8226	-59.28
Non-Small Cell Lung Cancer	
A549/ATCC	-73.73
EKVX	-81.47
HOP-62	-95.90
HOP-92	-81.69
NCI-H226	-57.24
NCI-H23	-51.20
NCI-H322M	-52.77
NCI-H460	-65.08
NCI-H522	-79.30
Colon Cancer	
HCC-2998	-92.35
HCT-116	-88.21
HCT-15	-79.85
HT29	-79.26
KM12	-89.25
SW-620	-49.87
CNS Cancer	
SF-268	-80.28
SF-295	-84.56
SF-539	-84.61
SNB-19	-95.91
SNB-75	-9.56
U251	-96.02
Melanoma	
LOX IMVI	-97.06
MALME-3M	-87.32
M14	-81.77
MDA-MB-435	-65.11
SK-MEL-2	-82.57
SK-MEL-28	-85.71
SK-MEL-5	-97.65
UACC-257	-65.28
UACC-62	-74.66
Ovarian Cancer	
IGROV1	-69.17
OVCAR-3	-93.20
OVCAR-4	-24.86
OVCAR-5	-77.81
OVCAR-8	-76.16
NCI/ADR-RES	-75.10
SK-OV-3	-89.52
Renal Cancer	
786-0	-85.02
A498	-83.16
ACHN	-94.41
CAKI-1	-42.50
RXF 393	-94.36
SN12C	-95.31
TK-10	-97.40
UO-31	-92.48
Prostate Cancer	
PC-3	-79.62
DU-145	-81.53
Breast Cancer	
MCF7	-73.82
MDA-MB-231/ATCC	-80.14
HS 578T	-30.69
BT-549	-81.21
MDA-MB-468	-83.23
Mean	-74.90
Delta	22.75
Range	88.09

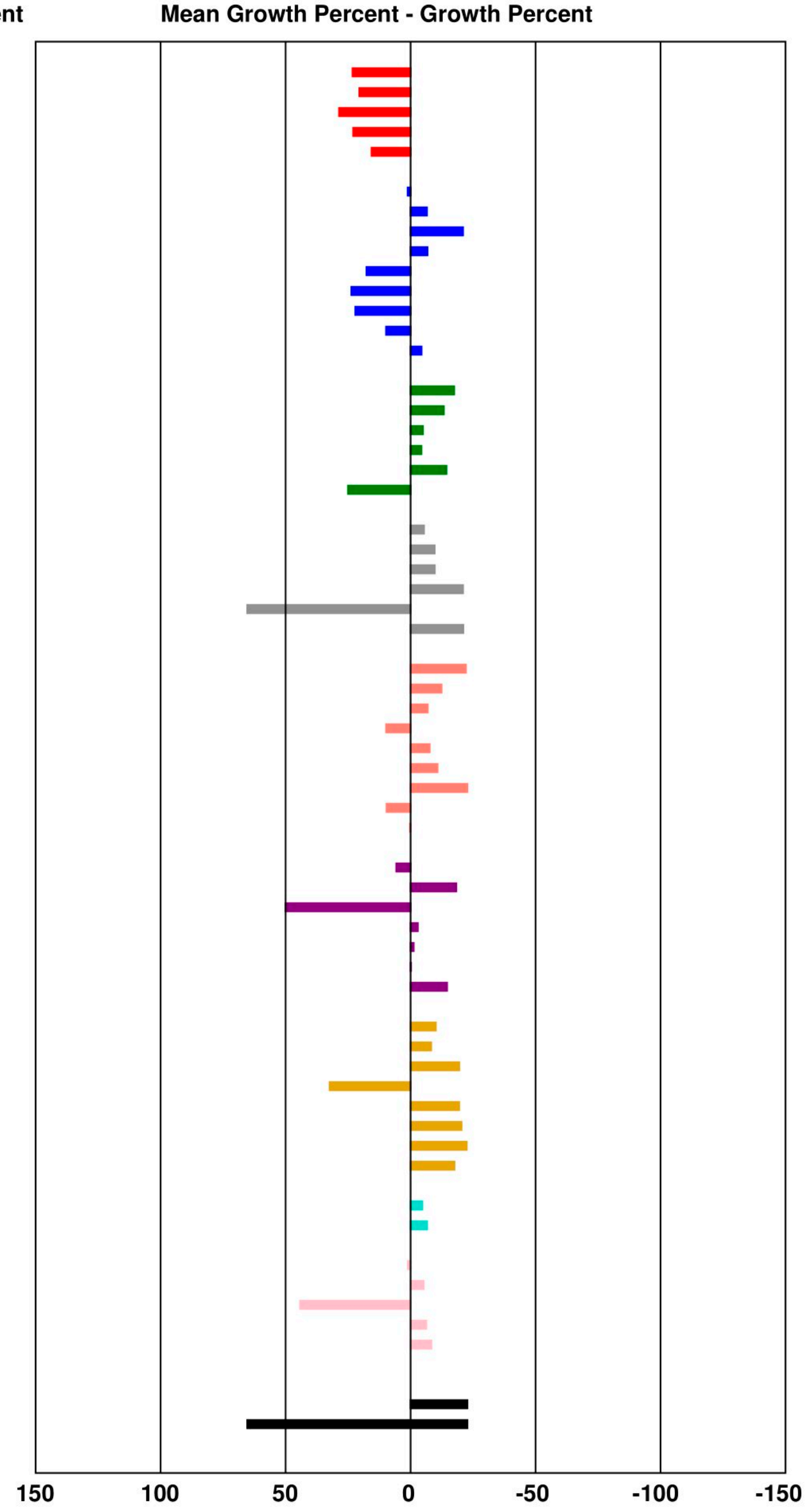

Figure S22. Cytostatic and cytotoxic effects of compound **29** (NSC 801984) on the NCI-60 panel of cancer cell lines at different doses, ranging from 10 nM to 100 μM. Results are displayed as dose-response curves (% growth versus sample concentration) for each cell line in the nine cancer subpanels.

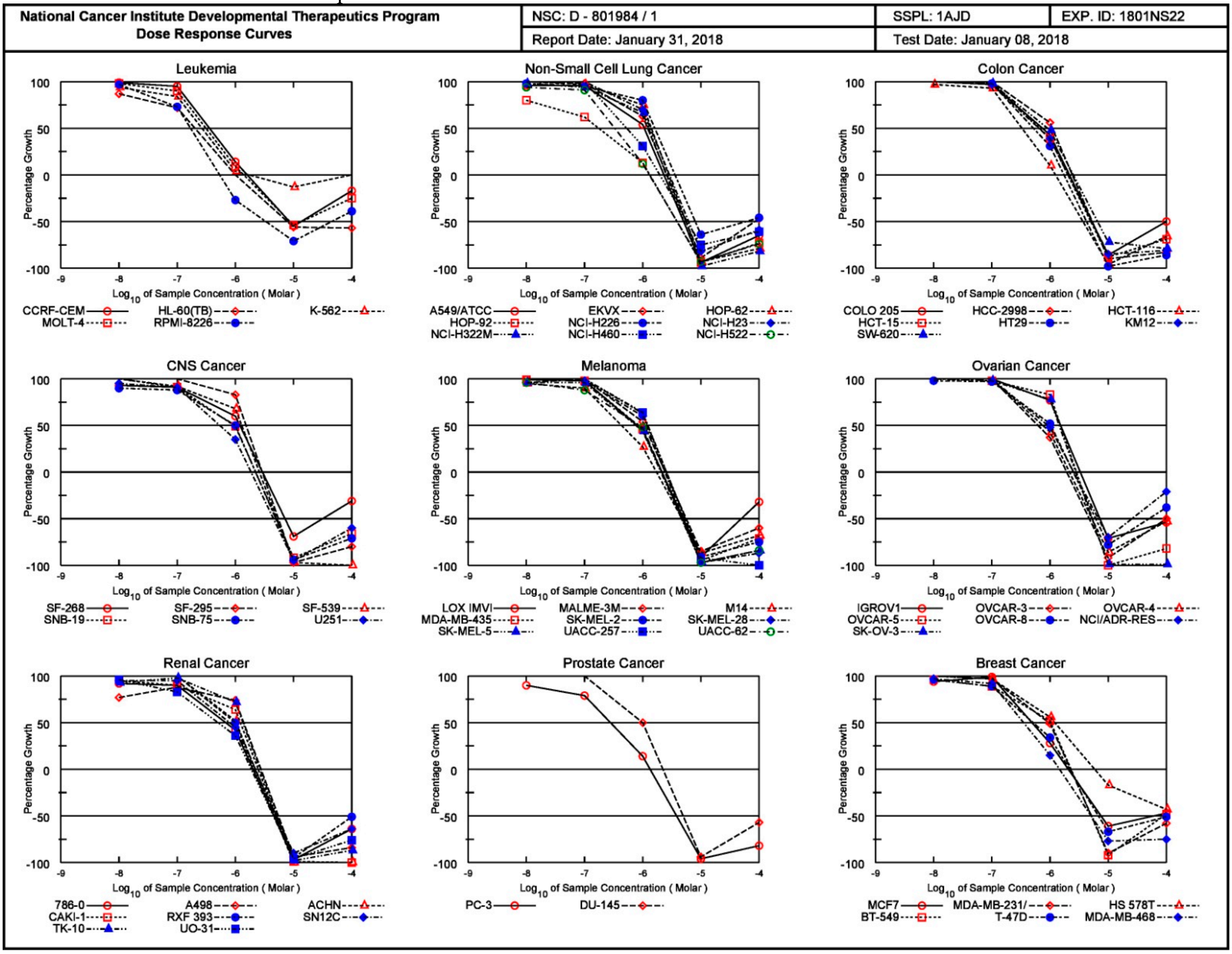

National Cancer Institute Developmental Therapeutics Program
In-Vitro Testing Results

NSC : D - 801984 / 1	Experiment ID : 1801NS22	Test Type : 08	Units : Molar
Report Date : January 31, 2018	Test Date : January 08, 2018	QNS :	MC :
COMI : PAV-74	Stain Reagent : SRB Dual-Pass Related	SSPL : 1AJD	

			Log10 Concentration												
	Time		Mean Optical Densities					Percent Growth							
Panel/Cell Line	Zero	Ctrl	-8.0	-7.0	-6.0	-5.0	-4.0	-8.0	-7.0	-6.0	-5.0	-4.0	GI50	TGI	LC50
**Leukemia**															
CCRF-CEM	0.545	2.910	2.892	2.798	0.875	0.243	0.453	99	95	14	-55	-17	3.60E-7	1.59E-6	.
HL-60(TB)	0.595	2.447	2.202	1.924	0.592	0.259	0.259	87	72	.	-56	-57	2.00E-7	9.81E-7	7.66E-6
K-562	0.147	1.784	1.680	1.518	0.218	0.128	0.155	94	84	4	-13	.	2.66E-7	.	> 1.00E-4
MOLT-4	0.552	2.524	2.493	2.320	0.725	0.252	0.414	98	90	9	-54	-25	3.09E-7	1.38E-6	.
RPMI-8226	0.764	2.310	2.263	1.895	0.561	0.223	0.469	97	73	-27	-71	-39	1.71E-7	5.41E-7	.
**Non-Small Cell Lung Cancer**															
A549/ATCC	0.463	2.228	2.153	2.165	1.410	0.030	0.162	96	96	54	-94	-65	1.06E-6	2.32E-6	5.06E-6
EKVX	0.635	2.014	2.036	1.984	1.501	0.072	0.337	102	98	63	-89	-47	1.21E-6	2.60E-6	.
HOP-62	0.756	2.154	2.174	2.108	1.805	0.053	0.161	101	97	75	-93	-79	1.41E-6	2.80E-6	5.54E-6
HOP-92	1.231	1.774	1.668	1.569	1.303	0.069	0.329	80	62	13	-94	-73	1.77E-7	1.33E-6	3.87E-6
NCI-H226	0.661	1.473	1.457	1.429	1.314	0.241	0.355	98	95	80	-64	-46	1.63E-6	3.62E-6	.
NCI-H23	0.453	1.900	1.968	1.994	1.462	0.083	0.185	105	106	70	-82	-59	1.35E-6	2.89E-6	6.18E-6
NCI-H322M	0.660	1.746	1.719	1.676	1.390	0.011	0.120	98	94	67	-98	-82	1.27E-6	2.55E-6	5.10E-6
NCI-H460	0.234	2.384	2.448	2.392	0.906	0.058	0.092	103	100	31	-75	-61	5.35E-7	1.96E-6	5.78E-6
NCI-H522	0.655	1.785	1.720	1.680	0.789	0.045	0.168	94	91	12	-93	-74	3.28E-7	1.30E-6	3.88E-6
**Colon Cancer**															
COLO 205	0.571	1.960	2.083	1.921	1.172	0.082	0.287	109	97	43	-86	-50	7.51E-7	2.17E-6	.
HCC-2998	0.911	3.069	3.072	3.115	2.112	0.088	0.157	100	102	56	-90	-83	1.09E-6	2.41E-6	5.29E-6
HCT-116	0.199	1.714	1.672	1.615	0.351	0.009	0.068	97	93	10	-96	-66	3.32E-7	1.24E-6	3.69E-6
HCT-15	0.240	1.805	1.809	1.798	0.847	0.028	0.074	100	100	39	-88	-69	6.54E-7	2.02E-6	4.99E-6
HT29	0.219	1.445	1.480	1.417	0.596	0.004	0.031	103	98	31	-98	-86	5.15E-7	1.73E-6	4.23E-6
KM12	0.431	2.355	2.622	2.293	1.187	0.065	0.082	114	97	39	-85	-81	6.51E-7	2.07E-6	5.23E-6
SW-620	0.238	1.597	1.767	1.568	0.894	0.066	0.050	113	98	48	-72	-79	9.24E-7	2.51E-6	6.51E-6
**CNS Cancer**															
SF-268	0.633	2.130	2.021	1.990	1.524	0.195	0.437	93	91	60	-69	-31	1.19E-6	2.90E-6	.
SF-295	0.773	2.646	2.652	2.657	2.328	0.025	0.153	100	101	83	-97	-80	1.53E-6	2.89E-6	5.49E-6
SF-539	0.985	2.521	2.528	2.402	2.028	0.030	-0.030	100	92	68	-97	-100	1.28E-6	2.58E-6	5.19E-6
SNB-19	0.453	1.866	1.870	1.744	1.148	0.037	0.154	100	91	49	-92	-66	9.56E-7	2.23E-6	5.05E-6
SNB-75	0.907	1.658	1.584	1.567	1.285	0.059	0.264	90	88	50	-94	-71	1.00E-6	2.24E-6	4.98E-6
U251	0.314	1.604	1.539	1.489	0.771	0.017	0.125	95	91	35	-95	-60	5.47E-7	1.87E-6	4.53E-6
**Melanoma**															
LOX IMVI	0.298	2.407	2.393	2.371	1.244	0.026	0.203	99	98	45	-91	-32	8.00E-7	2.13E-6	.
MALME-3M	0.503	1.059	1.039	1.094	0.806	0.073	0.200	96	106	54	-85	-60	1.08E-6	2.45E-6	5.58E-6
M14	0.381	1.545	1.482	1.425	0.698	0.049	0.121	95	90	27	-87	-68	4.32E-7	1.73E-6	4.73E-6
MDA-MB-435	0.483	2.374	2.357	2.337	1.356	0.025	0.140	99	98	46	-95	-71	8.44E-7	2.13E-6	4.81E-6
SK-MEL-2	1.024	1.940	2.011	2.000	1.601	0.091	0.253	108	107	63	-91	-75	1.21E-6	2.56E-6	5.41E-6
SK-MEL-28	0.601	1.698	1.770	1.679	1.256	0.016	0.076	107	98	60	-97	-87	1.15E-6	2.40E-6	5.00E-6
SK-MEL-5	0.876	3.133	3.043	3.038	1.865	0.028	0.137	96	96	44	-97	-84	7.60E-7	2.05E-6	4.64E-6
UACC-257	1.037	2.152	2.180	2.171	1.755	0.088	-0.043	103	102	64	-92	-100	1.24E-6	2.59E-6	5.41E-6
UACC-62	0.785	2.558	2.495	2.348	1.628	0.025	0.129	96	88	48	-97	-84	8.70E-7	2.13E-6	4.74E-6
**Ovarian Cancer**															
IGROV1	0.430	1.654	1.789	1.743	1.372	0.125	0.199	111	107	77	-71	-54	1.52E-6	3.31E-6	7.21E-6
OVCAR-3	0.302	1.303	1.427	1.353	0.677	0.024	0.151	112	105	37	-92	-50	6.51E-7	1.95E-6	1.00E-4
OVCAR-4	0.663	1.505	1.565	1.533	1.029	0.096	0.311	107	103	43	-86	-53	7.77E-7	2.17E-6	5.30E-6
OVCAR-5	0.661	1.389	1.392	1.377	1.266	.	0.122	100	98	83	-100	-82	1.52E-6	2.84E-6	5.33E-6
OVCAR-8	0.478	2.093	2.060	2.052	1.316	0.103	0.298	98	97	52	-78	-38	1.03E-6	2.50E-6	.
NCI/ADR-RES	0.422	1.651	1.713	1.712	1.008	0.125	0.335	105	105	48	-70	-21	9.11E-7	2.53E-6	.
SK-OV-3	0.834	1.798	1.832	1.774	1.583	0.011	0.008	104	98	78	-99	-99	1.44E-6	2.76E-6	5.29E-6
**Renal Cancer**															
786-0	0.549	2.178	2.047	2.018	1.226	0.017	0.197	92	90	42	-97	-64	6.70E-7	2.00E-6	4.58E-6
A498	1.455	2.188	2.023	2.102	1.998	0.090	0.230	77	88	74	-94	-84	1.39E-6	2.76E-6	5.48E-6
ACHN	0.374	1.503	1.578	1.518	0.964	-0.001	-0.003	107	101	52	-100	-100	1.03E-6	2.20E-6	4.69E-6
CAKI-1	0.552	1.784	1.727	1.664	1.344	0.004	-0.049	95	90	64	-99	-100	1.22E-6	2.47E-6	5.00E-6
RXF 393	1.092	1.742	1.858	1.788	1.418	0.057	0.540	118	107	50	-95	-51	1.00E-6	2.22E-6	4.91E-6
SN12C	0.466	1.786	1.714	1.724	1.064	0.046	0.166	95	95	45	-90	-64	8.04E-7	2.16E-6	5.05E-6
TK-10	0.700	1.470	1.413	1.451	1.251	0.013	0.089	93	98	72	-98	-87	1.34E-6	2.64E-6	5.20E-6
UO-31	0.583	1.947	1.886	1.713	1.070	0.023	0.143	96	83	36	-96	-76	4.97E-7	1.87E-6	4.47E-6
**Prostate Cancer**															
PC-3	0.539	1.942	1.803	1.642	0.729	0.024	0.099	90	79	14	-96	-82	2.75E-7	1.33E-6	3.82E-6
DU-145	0.422	1.782	1.866	1.872	1.098	0.026	0.182	106	107	50	-94	-57	9.89E-7	2.22E-6	4.95E-6
**Breast Cancer**															
MCF7	0.493	2.619	2.502	2.589	1.081	0.192	0.263	94	99	28	-61	-47	4.84E-7	2.05E-6	.
MDA-MB-231/ATCC	0.558	1.551	1.643	1.521	1.042	0.057	0.236	109	97	49	-90	-58	9.43E-7	2.25E-6	5.16E-6
HS 578T	1.124	2.039	2.137	2.012	1.640	0.932	0.646	111	97	56	-17	-43	1.22E-6	5.85E-6	> 1.00E-4
BT-549	1.065	2.190	2.141	2.065	1.655	0.091	0.558	96	89	52	-92	-48	1.04E-6	2.31E-6	.
T-47D	0.990	2.165	2.119	2.037	1.389	0.323	0.481	96	89	34	-67	-51	5.11E-7	2.16E-6	6.74E-6
MDA-MB-468	1.052	2.138	2.108	2.049	1.217	0.244	0.265	97	92	15	-77	-75	3.52E-7	1.46E-6	5.11E-6

Figure S23. GI50 (50% Growth Inhibition), TGI (Total Growth Inhibition) and LC50 (50% Lethal Concentration) mean graphs obtained for compound **29** (NSC 801984) tested at five concentrations (0.01, 0.1, 1, 10, 100 μM) against the NCI-60 human cancer cell lines.

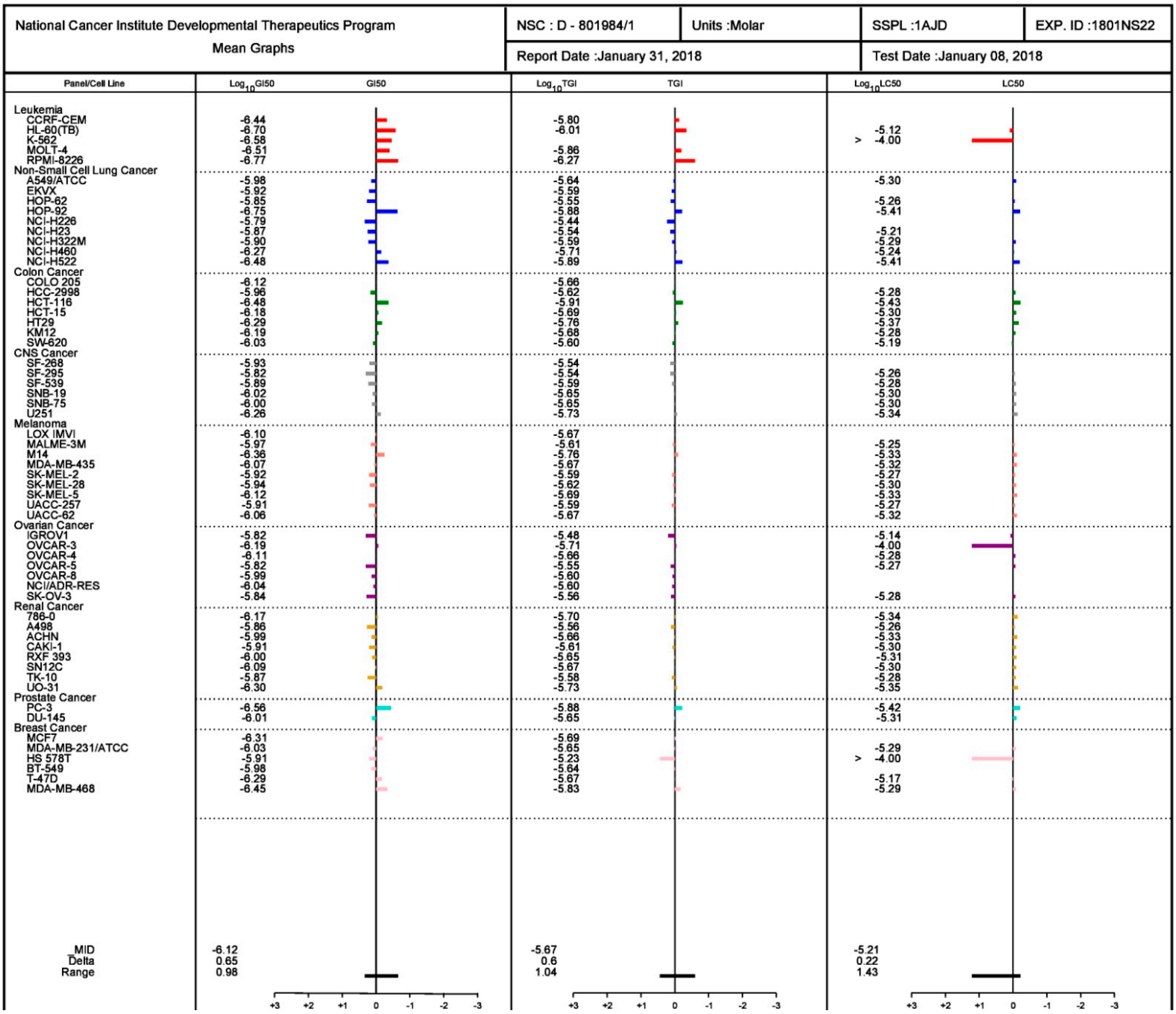

National Cancer Institute Developmental Therapeutics Program Dose Response Curves	NSC : D - 801984/1	SSPL:1AJD	EXP. ID:1801NS22
	Report Date:January 31, 2018	Test Date:January 08, 2018	

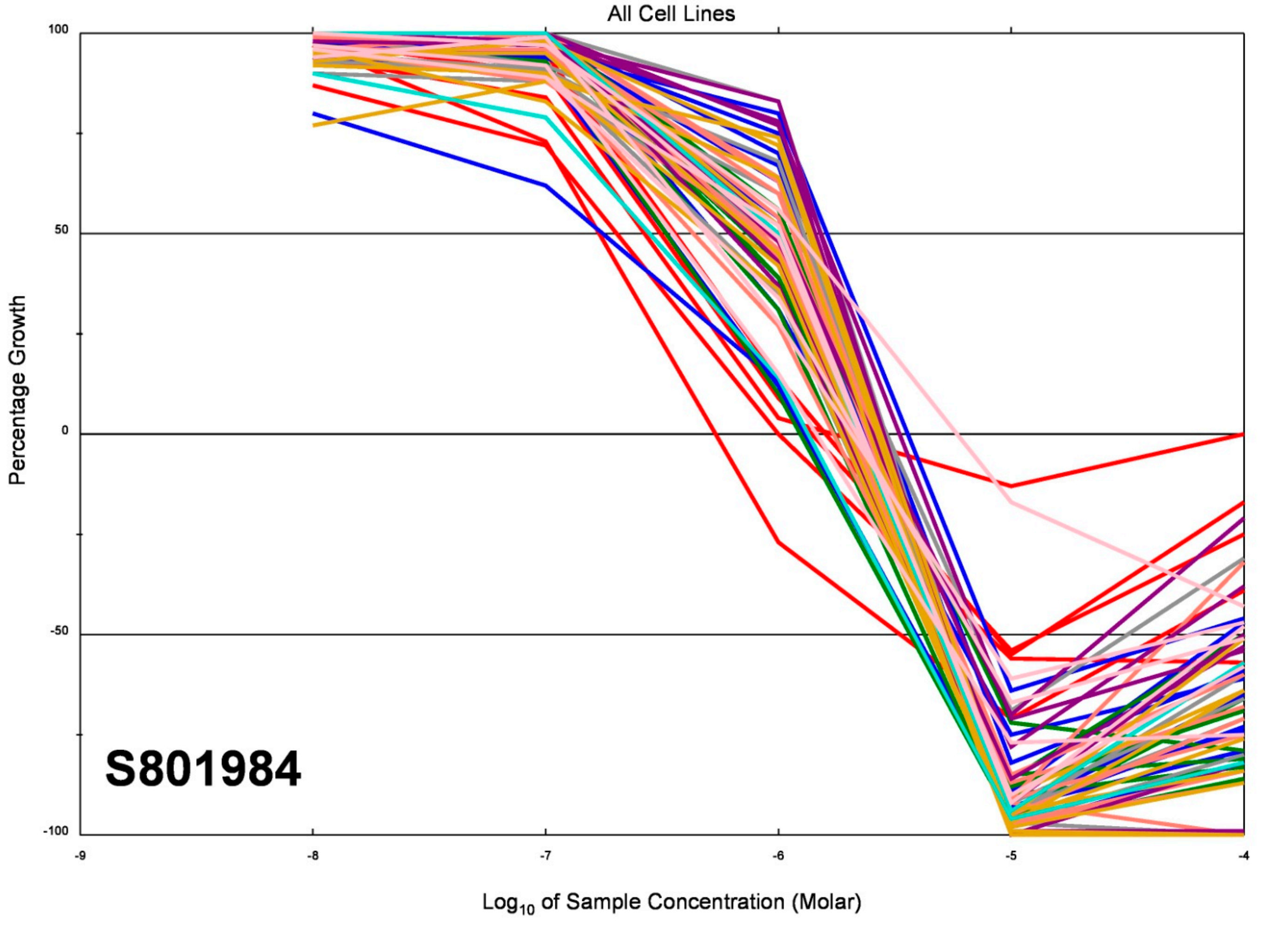

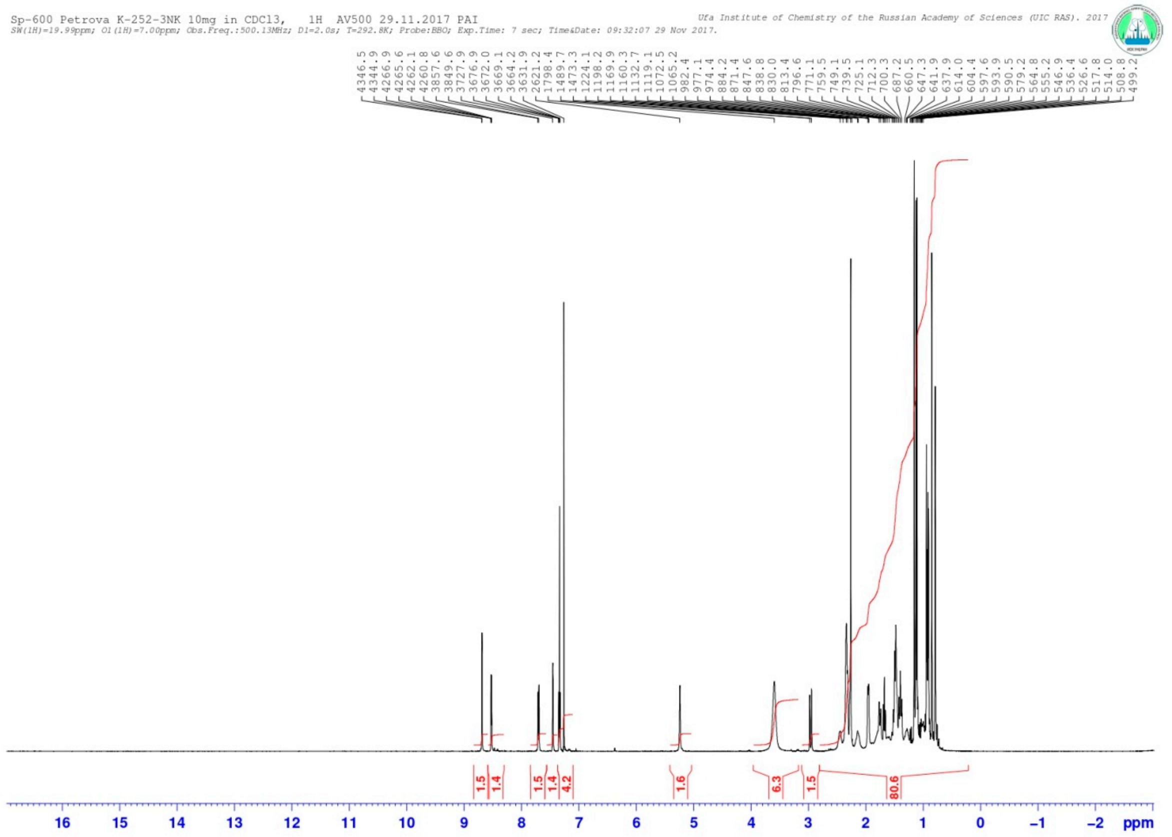

Figure S24. Spectrum NMR ^{1}H of compound **29**

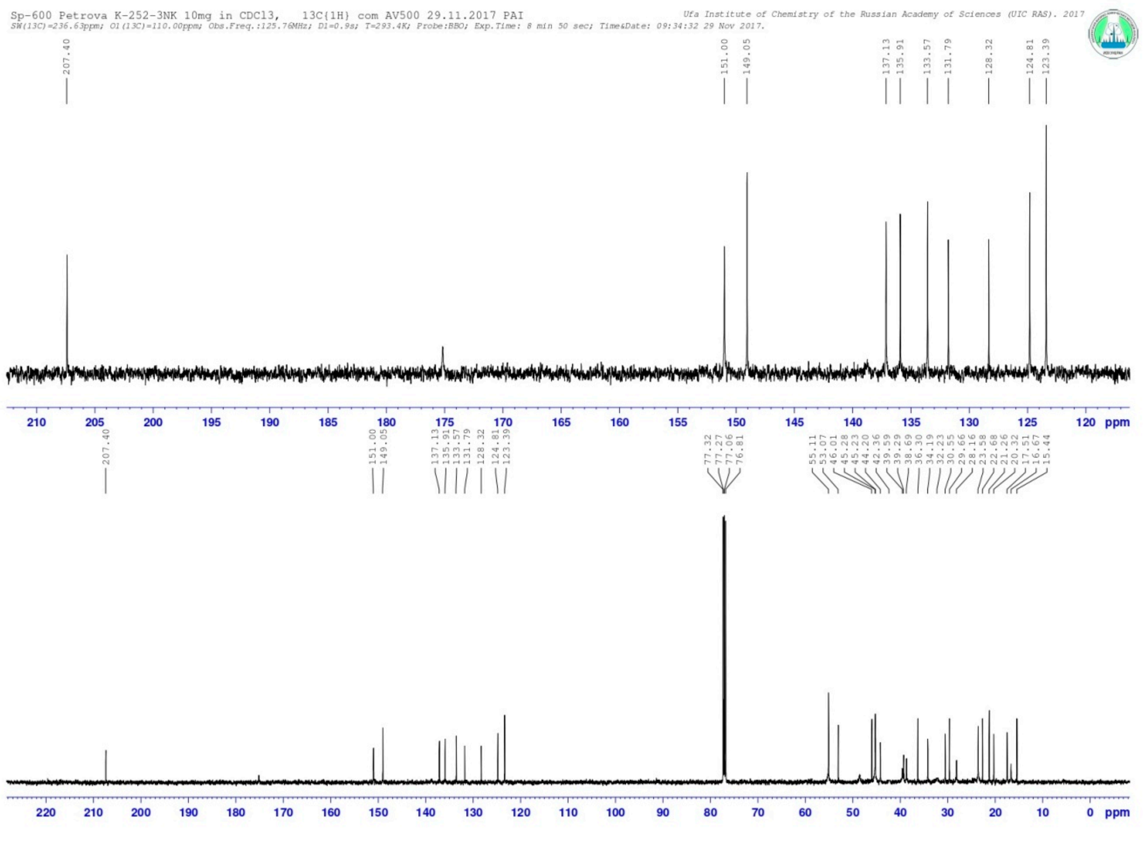

Figure S25. Spectrum NMR ^{13}C of compound **29**

Figure S26. Percentage of growth inhibition of compounds **32** against 60 individual cell lines

**Developmental Therapeutics Program One Dose Mean Graph**	**NSC:** D-804712 / 1	**Conc:** 1.00E-4 Molar	**Test Date:** Apr 30, 2018
	**Experiment ID:** 1804OS61		**Report Date:** Mar 24, 2020

Panel/Cell Line	Growth Percent
Leukemia	
CCRF-CEM	-26.43
HL-60(TB)	-3.02
K-562	7.11
MOLT-4	-10.76
RPMI-8226	-19.29
SR	31.21
Non-Small Cell Lung Cancer	
A549/ATCC	-52.50
EKVX	-68.38
HOP-62	-75.65
HOP-92	-87.70
NCI-H226	-52.26
NCI-H23	-81.14
NCI-H322M	-73.42
NCI-H460	-55.64
NCI-H522	-82.75
Colon Cancer	
COLO 205	-60.86
HCC-2998	-81.38
HCT-116	-72.97
HCT-15	-71.18
HT29	-67.91
KM12	-73.47
SW-620	-44.87
CNS Cancer	
SF-268	-58.68
SF-295	-79.37
SF-539	-80.08
SNB-19	-52.62
SNB-75	-55.90
U251	-64.53
Melanoma	
LOX IMVI	-91.68
MALME-3M	-85.97
M14	-43.60
MDA-MB-435	-85.11
SK-MEL-2	-64.03
SK-MEL-28	-79.25
SK-MEL-5	-91.68
UACC-257	-39.11
UACC-62	-94.42
Ovarian Cancer	
IGROV1	-59.55
OVCAR-3	-74.54
OVCAR-5	-48.80
OVCAR-8	-18.97
NCI/ADR-RES	-58.89
SK-OV-3	-17.34
Renal Cancer	
786-0	-40.32
A498	-56.23
ACHN	-97.88
CAKI-1	-74.62
RXF 393	-84.03
SN12C	-56.00
TK-10	-73.40
UO-31	-99.47
Prostate Cancer	
PC-3	-67.12
DU-145	-73.15
Breast Cancer	
MCF7	-74.67
MDA-MB-231/ATCC	-81.50
HS 578T	-13.88
BT-549	-62.50
T-47D	-14.27
MDA-MB-468	-38.00
Mean	-58.89
Delta	40.58
Range	130.68

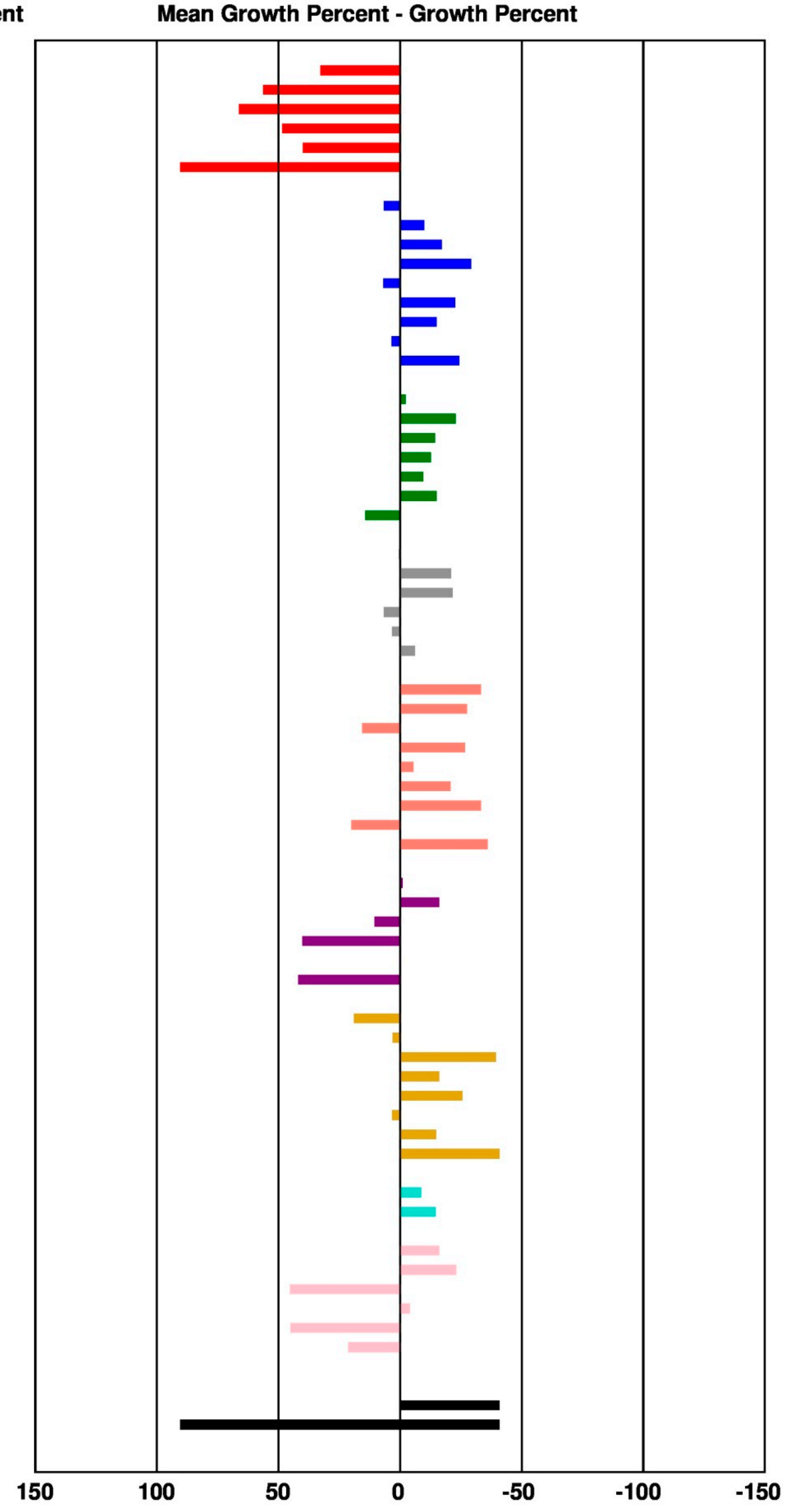

Figure S27. Cytostatic and cytotoxic effects of compound **32** (NSC 804692) on the NCI-60 panel of cancer cell lines at different doses, ranging from 10 nM to 100 μM. Results are displayed as dose-response curves (% growth versus sample concentration) for each cell line in the nine cancer subpanels.

National Cancer Institute Developmental Therapeutics Program Dose Response Curves	NSC: D - 804692 / 1	SSPL: 1AJD	EXP. ID: 1808NS87
	Report Date: August 29, 2018	Test Date: August 06, 2018	

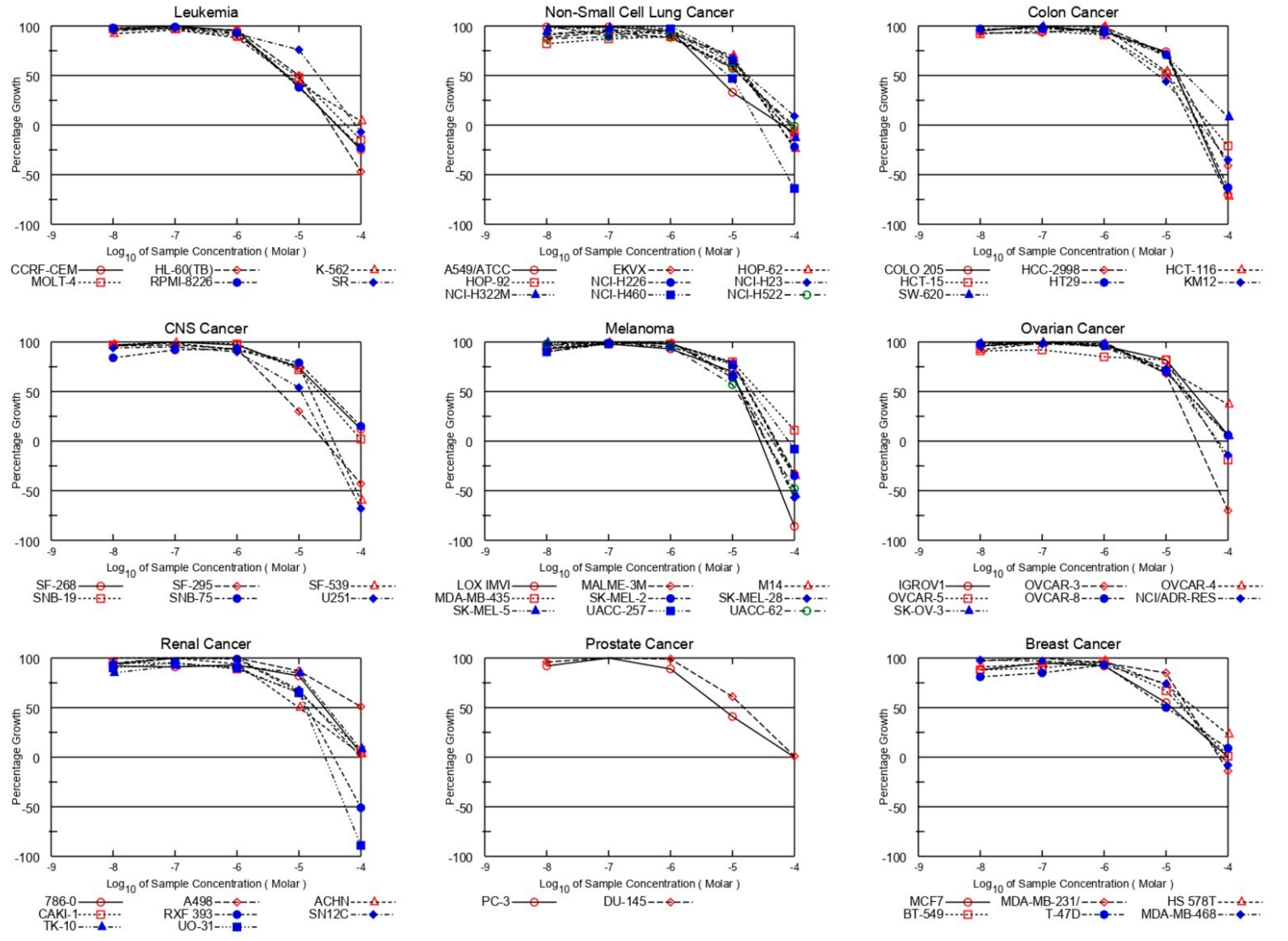

National Cancer Institute Developmental Therapeutics Program
In-Vitro Testing Results

NSC : D - 804692 / 1	Experiment ID : 1808NS87	Test Type : 08	Units : Molar
Report Date : August 29, 2018	Test Date : August 06, 2018	QNS :	MC :
COMI : UA-3NK-3OH	Stain Reagent : SRB Dual-Pass Related	SSPL : 1AJD	

			Log10 Concentration												
	Time		Mean Optical Densities					Percent Growth							
Panel/Cell Line	Zero	Ctrl	-8.0	-7.0	-6.0	-5.0	-4.0	-8.0	-7.0	-6.0	-5.0	-4.0	GI50	TGI	LC50
**Leukemia**															
CCRF-CEM	0.546	2.776	2.718	2.803	2.676	1.420	0.410	97	101	95	39	-25	6.43E-6	4.08E-5	> 1.00E-4
HL-60(TB)	0.909	3.237	3.120	3.191	3.154	2.065	0.482	95	98	96	50	-47	9.83E-6	3.26E-5	> 1.00E-4
K-562	0.184	1.996	1.854	1.918	1.781	0.950	0.255	92	96	88	42	4	6.79E-6	> 1.00E-4	> 1.00E-4
MOLT-4	0.571	2.650	2.589	2.582	2.436	1.559	0.488	97	97	90	48	-15	8.74E-6	5.82E-5	> 1.00E-4
RPMI-8226	1.200	3.082	3.052	3.065	2.942	1.916	0.919	98	99	93	38	-23	6.03E-6	4.15E-5	> 1.00E-4
SR	0.279	1.406	1.363	1.380	1.315	1.138	0.260	96	98	92	76	-7	2.07E-5	8.28E-5	> 1.00E-4
**Non-Small Cell Lung Cancer**															
A549/ATCC	0.550	2.803	2.789	2.784	2.699	1.283	0.504	99	99	95	33	-8	5.27E-6	6.24E-5	> 1.00E-4
EKVX	0.967	2.576	2.390	2.483	2.381	1.893	0.933	88	94	88	58	-4	1.33E-5	8.76E-5	> 1.00E-4
HOP-62	0.589	1.936	1.816	1.865	1.831	1.535	0.448	91	95	92	70	-24	1.64E-5	5.56E-5	> 1.00E-4
HOP-92	1.052	1.791	1.662	1.699	1.713	1.497	0.923	82	87	89	60	-12	1.38E-5	6.77E-5	> 1.00E-4
NCI-H226	0.715	2.089	2.097	1.956	2.028	1.626	0.561	101	90	96	66	-22	1.53E-5	5.68E-5	> 1.00E-4
NCI-H23	0.567	2.024	1.912	1.966	1.936	1.482	0.703	92	96	94	63	9	1.73E-5	> 1.00E-4	> 1.00E-4
NCI-H322M	0.770	2.187	2.145	2.160	2.193	1.714	0.673	97	98	100	67	-13	1.62E-5	6.93E-5	> 1.00E-4
NCI-H460	0.103	2.078	2.077	2.181	2.014	1.041	0.037	100	105	97	47	-64	8.88E-6	2.66E-5	7.48E-5
NCI-H522	0.984	2.859	2.614	2.653	2.674	2.047	0.970	87	89	90	57	-1	1.30E-5	9.43E-5	> 1.00E-4
**Colon Cancer**															
COLO 205	0.485	2.185	2.100	2.184	2.083	1.736	0.144	95	100	94	74	-70	1.46E-5	3.24E-5	7.22E-5
HCC-2998	0.509	1.836	1.740	1.741	1.828	1.465	0.301	93	93	99	72	-41	1.57E-5	4.35E-5	> 1.00E-4
HCT-116	0.172	1.792	1.816	1.929	1.772	1.039	0.049	101	108	99	54	-72	1.07E-5	2.68E-5	6.73E-5
HCT-15	0.270	2.040	1.892	1.945	1.883	1.194	0.214	92	95	91	52	-21	1.07E-5	5.20E-5	> 1.00E-4
HT29	0.150	1.428	1.387	1.385	1.378	1.058	0.056	97	97	96	71	-63	1.44E-5	3.39E-5	8.00E-5
KM12	0.419	2.341	2.264	2.316	2.188	1.271	0.272	96	99	92	44	-35	7.61E-6	3.61E-5	> 1.00E-4
SW-620	0.173	2.278	2.294	2.266	2.145	1.646	0.333	101	99	94	70	8	2.09E-5	> 1.00E-4	> 1.00E-4
**CNS Cancer**															
SF-268	0.672	2.172	2.108	2.188	2.120	1.779	0.854	96	101	97	74	12	2.43E-5	> 1.00E-4	> 1.00E-4
SF-295	0.914	2.929	2.848	2.864	2.787	1.522	0.520	96	97	93	30	-43	4.83E-6	2.58E-5	> 1.00E-4
SF-539	0.940	2.604	2.550	2.581	2.478	2.206	0.379	97	99	92	76	-60	1.56E-5	3.63E-5	8.49E-5
SNB-19	0.727	2.512	2.450	2.541	2.473	2.007	0.764	97	102	98	72	2	2.05E-5	> 1.00E-4	> 1.00E-4
SNB-75	0.453	1.609	1.419	1.517	1.524	1.371	0.626	84	92	93	79	15	2.86E-5	> 1.00E-4	> 1.00E-4
U251	0.560	2.405	2.295	2.317	2.230	1.564	0.178	94	95	90	54	-68	1.09E-5	2.78E-5	7.10E-5
**Melanoma**															
LOX IMVI	0.303	2.356	2.218	2.305	2.222	1.730	0.044	93	98	93	70	-86	1.34E-5	2.81E-5	5.89E-5
MALME-3M	0.390	1.073	1.005	1.072	1.061	0.931	0.264	90	100	98	79	-32	1.83E-5	5.12E-5	> 1.00E-4
M14	0.418	1.983	1.891	1.962	1.955	1.637	0.272	94	99	98	78	-35	1.77E-5	4.90E-5	> 1.00E-4
MDA-MB-435	0.360	2.676	2.591	2.671	2.642	2.224	0.622	96	100	98	80	11	2.76E-5	> 1.00E-4	> 1.00E-4
SK-MEL-2	0.861	2.104	2.064	2.092	2.103	1.659	0.560	97	99	100	64	-35	1.39E-5	4.44E-5	> 1.00E-4
SK-MEL-28	0.798	2.332	2.338	2.404	2.351	1.825	0.343	100	105	101	67	-57	1.37E-5	3.47E-5	8.77E-5
SK-MEL-5	1.043	3.201	3.171	3.207	3.214	2.479	0.482	99	100	101	67	-54	1.37E-5	3.57E-5	9.29E-5
UACC-257	1.108	2.591	2.449	2.560	2.521	2.257	1.023	90	98	95	77	-8	2.10E-5	8.12E-5	> 1.00E-4
UACC-62	0.891	3.051	3.004	3.049	2.944	2.121	0.467	98	100	95	57	-48	1.17E-5	3.50E-5	> 1.00E-4
**Ovarian Cancer**															
IGROV1	0.550	2.338	2.273	2.337	2.261	2.019	0.653	96	100	96	82	6	2.64E-5	> 1.00E-4	> 1.00E-4
OVCAR-3	0.435	1.476	1.463	1.519	1.536	1.144	0.131	99	104	106	68	-70	1.35E-5	3.11E-5	7.16E-5
OVCAR-4	0.196	0.888	0.831	0.884	0.875	0.700	0.451	92	99	98	73	37	4.30E-5	> 1.00E-4	> 1.00E-4
OVCAR-5	0.553	1.373	1.301	1.306	1.254	1.229	0.447	91	92	85	82	-19	2.09E-5	6.48E-5	> 1.00E-4
OVCAR-8	0.559	2.488	2.457	2.484	2.450	1.902	0.683	98	100	98	70	6	2.05E-5	> 1.00E-4	> 1.00E-4
NCI/ADR-RES	0.631	2.211	2.145	2.185	2.141	1.791	0.540	96	98	96	73	-14	1.85E-5	6.85E-5	> 1.00E-4
SK-OV-3	0.941	2.050	2.023	2.034	1.997	1.706	1.001	98	99	95	69	5	1.99E-5	> 1.00E-4	> 1.00E-4
**Renal Cancer**															
786-0	0.509	2.355	2.215	2.181	2.221	2.025	0.592	92	91	93	82	4	2.59E-5	> 1.00E-4	> 1.00E-4
A498	0.817	1.917	1.851	1.915	1.928	1.772	1.380	94	100	101	87	51	> 1.00E-4	> 1.00E-4	> 1.00E-4
ACHN	0.375	1.671	1.580	1.688	1.596	1.029	0.412	93	101	94	50	3	1.02E-5	> 1.00E-4	> 1.00E-4
CAKI-1	0.173	1.575	1.505	1.498	1.416	1.078	0.265	95	95	89	65	7	1.78E-5	> 1.00E-4	> 1.00E-4
RXF 393	1.038	1.908	1.931	1.937	1.898	1.612	0.508	103	103	99	66	-51	1.37E-5	3.66E-5	9.79E-5
SN12C	0.613	2.625	2.527	2.641	2.620	1.975	0.608	95	101	100	68	.	1.81E-5	9.73E-5	> 1.00E-4
TK-10	0.579	1.594	1.439	1.512	1.510	1.444	0.656	85	92	92	85	8	2.84E-5	> 1.00E-4	> 1.00E-4
UO-31	0.729	1.977	1.850	1.908	1.857	1.540	0.078	90	95	90	65	-89	1.25E-5	2.64E-5	5.56E-5
**Prostate Cancer**															
PC-3	0.514	2.170	2.040	2.166	1.980	1.185	0.520	92	100	89	41	.	6.35E-6	> 1.00E-4	> 1.00E-4
DU-145	0.292	1.411	1.367	1.502	1.398	0.977	0.309	96	108	99	61	1	1.54E-5	> 1.00E-4	> 1.00E-4
**Breast Cancer**															
MCF7	0.304	2.002	1.790	1.922	1.862	1.230	0.312	88	95	92	55	.	1.21E-5	> 1.00E-4	> 1.00E-4
MDA-MB-231/ATCC	0.601	1.524	1.500	1.544	1.479	1.383	0.517	97	102	95	85	-14	2.25E-5	7.21E-5	> 1.00E-4
HS 578T	0.587	1.991	1.870	1.911	1.953	1.613	0.909	91	94	97	73	23	2.88E-5	> 1.00E-4	> 1.00E-4
BT-549	0.991	2.203	2.057	2.080	2.149	1.809	0.999	88	90	96	67	1	1.83E-5	> 1.00E-4	> 1.00E-4
T-47D	0.641	1.434	1.282	1.311	1.382	1.036	0.713	81	85	93	50	9	9.88E-6	> 1.00E-4	> 1.00E-4
MDA-MB-468	0.807	2.052	2.032	2.012	1.970	1.733	0.743	98	97	93	74	-8	1.98E-5	8.01E-5	> 1.00E-4

Figure S28. GI50 (50% Growth Inhibition), TGI (Total Growth Inhibition) and LC50 (50% Lethal Concentration) mean graphs obtained for compound **32** (NSC 804692) tested at five concentrations (0.01, 0.1, 1, 10, 100 μM) against the NCI-60 human cancer cell lines.

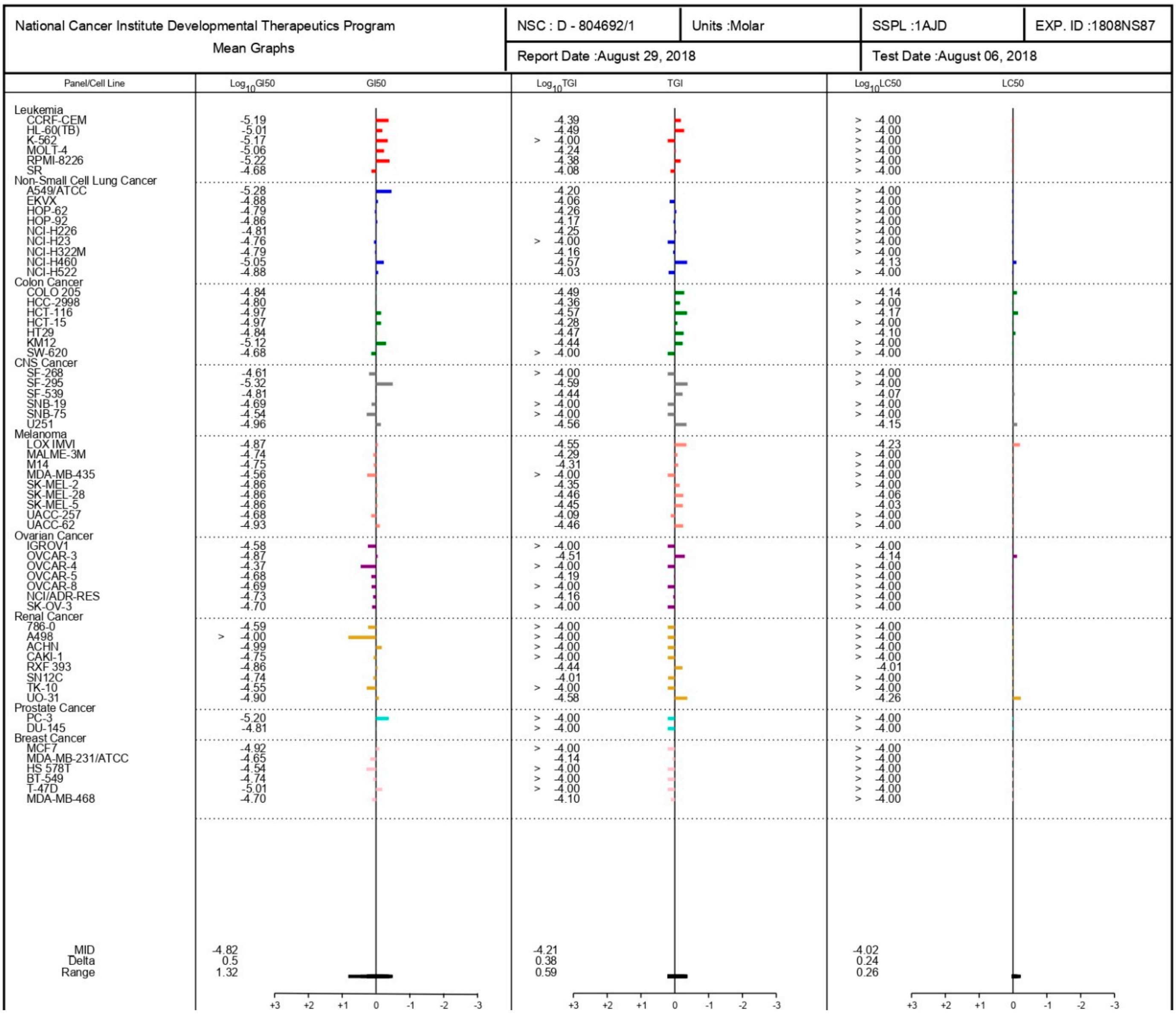

National Cancer Institute Developmental Therapeutics Program Dose Response Curves	NSC : D - 804692/1	SSPL:1AJD	EXP. ID:1808NS87
	Report Date:August 29, 2018	Test Date:August 06, 2018	

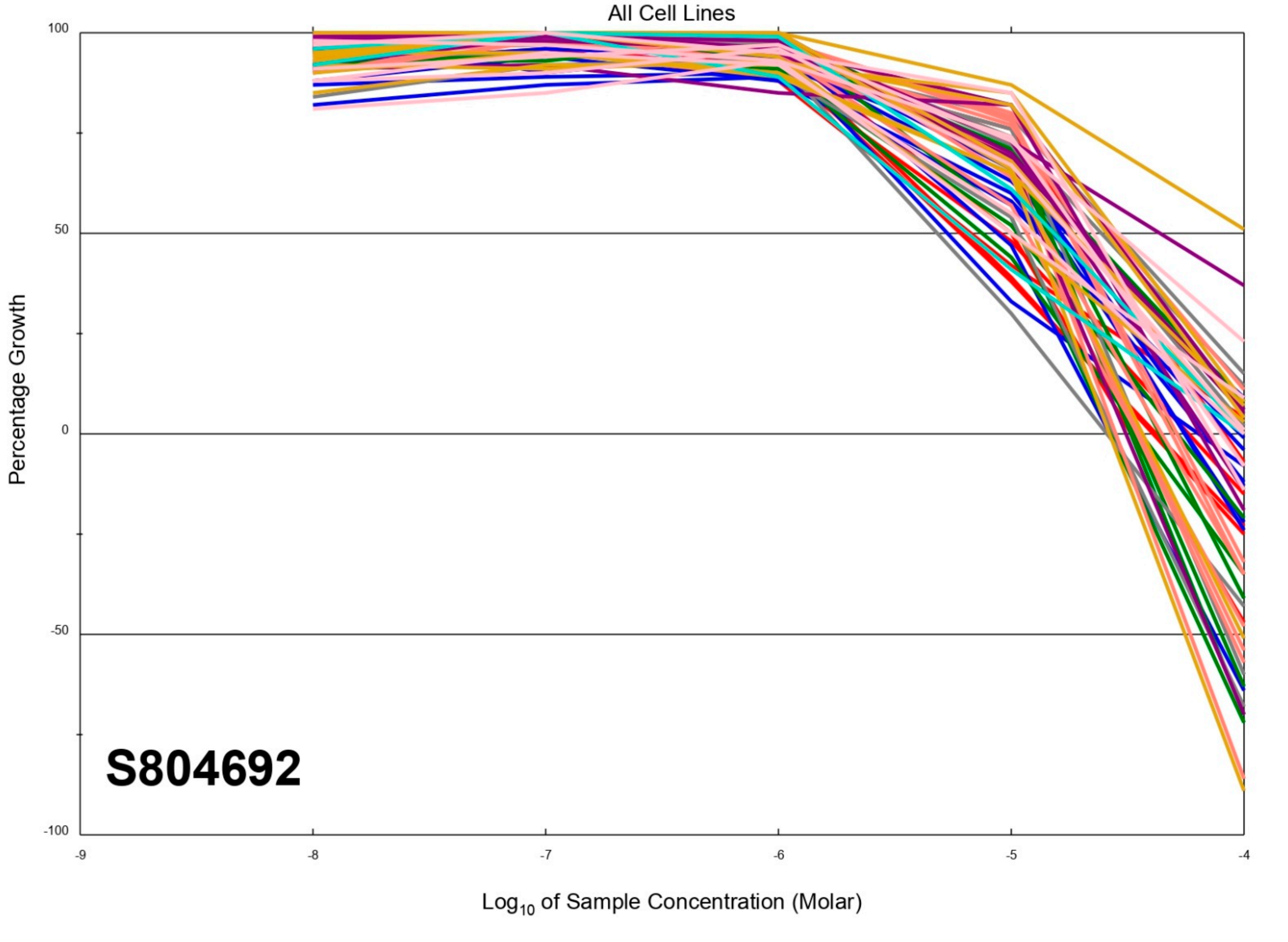

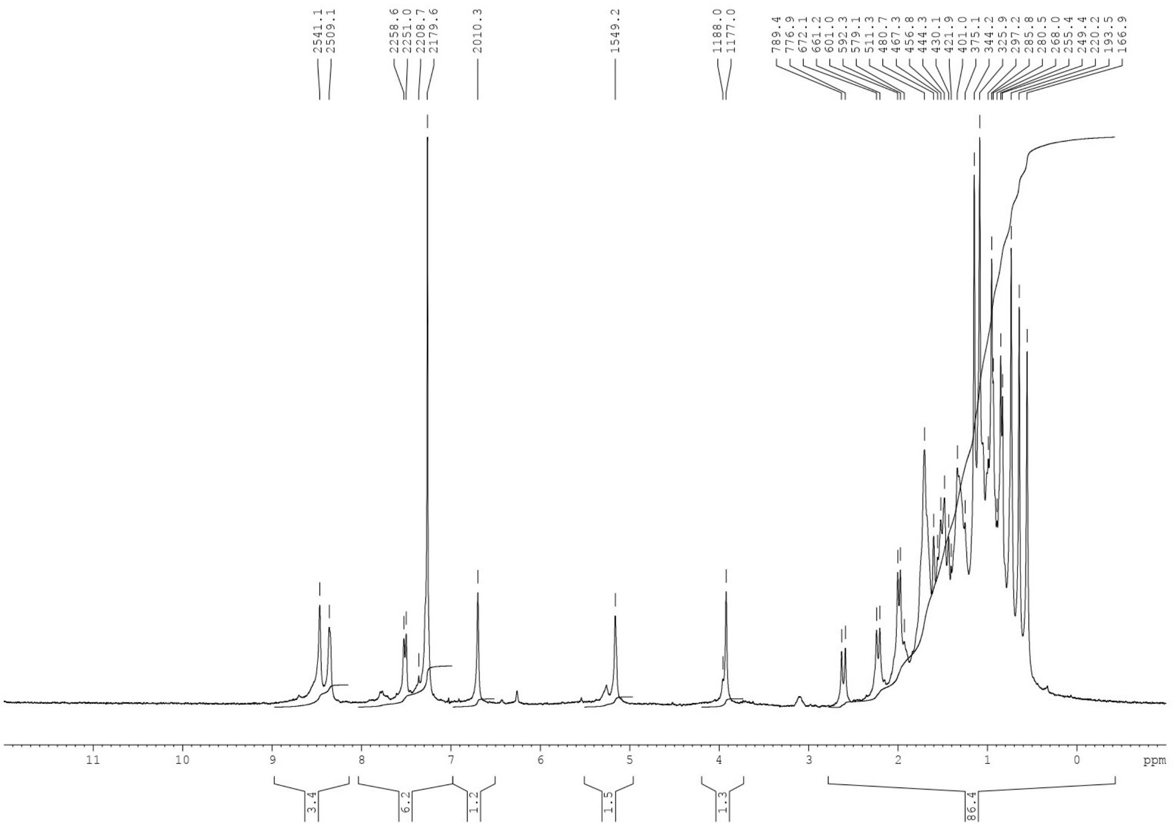

Figure S29. Spectrum NMR ^{1}H of compound **32**

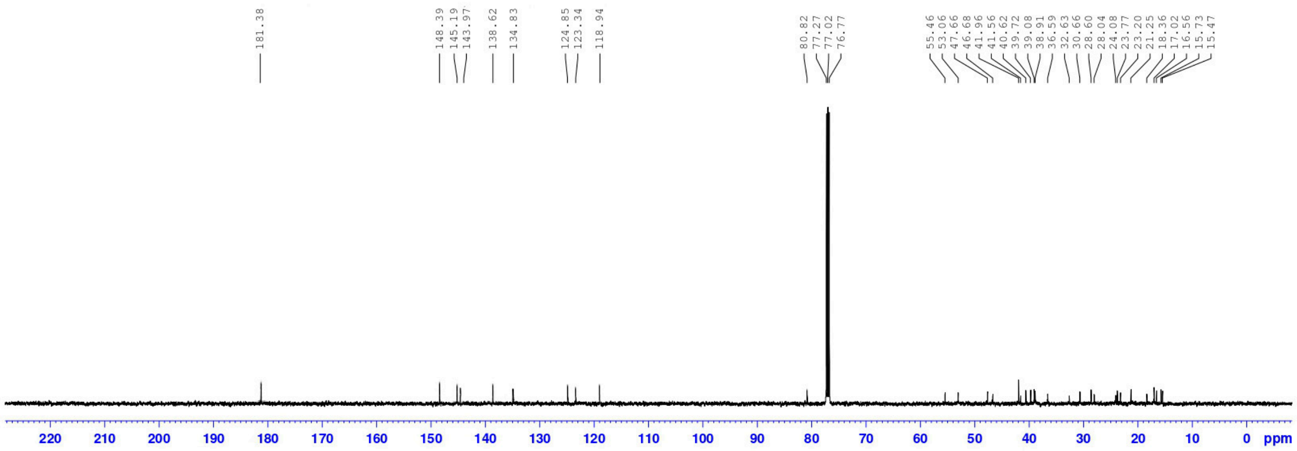

Figure S30. Spectrum NMR ^{13}C of compound **32**

Cytotoxicity assay

Culture conditions and treatments

HEK293 (human embryonic kidney 293 cells), A549 (human lung carcinoma), MCF7 (human breast adenocarcinoma), SH-SY5Y (human neuroblastoma) cell lines were purchased from the Russian Cell Culture Collection (Institute of Cytology Russian Academy of Science, Saint Petersburg, Russia). HEK293, A549, MCF7 and SH-SY5Y cells were maintained in Dulbecco's modified Eagle's medium (DMEM) (Invitrogen, USA) supplemented with 2 mM L-glutamine (Sigma-Aldrich, UK), 10% fetal bovine serum (FBS; Invitrogen, USA), 50 µg/mL gentamicin sulfate (Invitrogen, USA) at 37°C and 5% CO2. Compound **29** was dissolved in 100% DMSO (Sigma-Aldrich, UK) to 100 mM stock solutions and diluted in completed DMEM immediately before addition to the assay plates. DMSO was maintained at a final concentration of 0.1%.

Cell viability

Cells were cultured at appropriate density in 96-well plates (3×10^4 cells/well for HEK293; 1.2×10^4 cells/well for A549; 1.2×10^4 cells/well for MCF7, 3×10^4 cells/well for SH-SY5Y) and allowed to grow for 24 h. Thereafter, cells were treated with compound **29** at a final concentrations of 1, 10, 100 µM for 72 hours and cell viability was measured by conventional MTT assay following manufacturer's instruction (Thermo Fisher Scientific, USA) using «2300 EnSpire® Multimode Plate Reader» (Perkin Elmer, USA) at 590 nm. The concentration of the compound that inhibited 50% cell viability (IC_{50} value) was calculated using nonlinear regression analysis (GraphPad Prism v.5.02; GraphPad Software Inc., USA). The viability of control group (cells treated with 0.1% DMSO) was set at 100%, and viability of treated groups was determined through the comparison of its optical density with control. Data were expressed as mean ± S.E.M. calculated from two independent experiments, performed in triplicate.

Table S3. In vitro cytotoxic activity of compound **29** in human HEK293, A549, MCF-7, SH-SY5Y cell lines

Compound	IC_{50} (µM)[a]			
	HEK293	A549	MCF-7	SH-SY5Y
**29**	14.69 ± 1.20	14.73 ± 1.47	11.15 ± 2.53	14.63 ± 3.35

Note. [a] IC_{50} (*µ*M) values obtained from MTT assays. Cells were incubated with compounds for 72 hours. Values were the mean ± S.E.M. from two independent experiments, performed in triplicate.

Table S4. Gene ontology (GO) term enrichment analysis for lead compounds across the NCI-60 panel cell lines based on CellMiner analysis

Cmpd.	GO Term	*P* value	Adjusted *P* value[1]	Genes
**7**	T cell activation	2.8712E-18	6.6094E-15	CD86 WAS NLRC3 CD3G IKZF1 CD1D GIMAP5 ITGAL CD3E CD3D CD1A SPN RAG2 RAG1 BCL11B BCL11A THEMIS RHOH CD2 ZAP70 LCK CD8A TREML2 CD7 CD48 ADA
	Lymphocyte activation	4.9741E-17	5.7252E-14	CD86 WAS CXCR5 NLRC3 CD3G IKZF1 CD1D GIMAP5 ITGAL CD3E CD3D CD1A SPN RAG2 RAG1 BCL11B BCL11A THEMIS RHOH LAX1 TSHR IL4 CD2 ZAP70 LCK CD8A TREML2 CD7 CD48 ADA
	Leukocyte activation	2.5989E-14	1.9942E-11	CD86 WAS CXCR5 NLRC3 CD3G IKZF1 CD1D GIMAP5 ITGAL CD3E CD3D CD1A SPN RAG2 RAG1 BCL11B BCL11A THEMIS RHOH LAX1 TSHR IL4 CD2 ZAP70 LCK CD8A TREML2 CD7 CD48 ADA
	Lymphocyte differentiation	6.3691E-13	3.3232E-10	BCL11B BCL11A THEMIS RHOH IKZF1 CD1D GIMAP5 CD3E CD3D CD1A TSHR SPN IL4 ZAP70 LCK CD8A RAG2 ADA RAG1
	T cell differentiation	7.2181E-13	3.3232E-10	BCL11B BCL11A THEMIS RHOH IKZF1 CD1D GIMAP5 CD3E CD3D CD1A SPN ZAP70 LCK CD8A RAG2 RAG1
	Cell activation	2.0708E-12	7.9450E-10	CD86 WAS CXCR5 NLRC3 CD3G IKZF1 CD1D GIMAP5 ITGAL CD3E CD3D CD1A SPN RAG2 RAG1 BCL11B BCL11A THEMIS RHOH LAX1 TSHR IL4 CD2 ZAP70 LCK CD8A TREML2 CD7 CD48 ADA
	Immune system process	2.7378E-12	9.0034E-10	CD86 IGHV1OR15-1 LST1 CD3G IKZF1 GIMAP5 ITGAL CD3E CD3D SPN VPREB1 CYSLTR2 ARHGDIB CCR9 RAG2 RAG1 CD96 CR2 THEMIS RHOH CBFA2T3 LAX1 SIGIRR ZAP70 LCK CD8A PECAM1 HCLS1 CD48 NOTCH1 TCF7 WAS CXCR5 NLRC3 CD1E CD1D IL2RG CD1C CORO1A CD1B FYB CD1A PSMB10 TRPM2 SEMA4D BCL11B BCL11A TSHR SLC25A38 IL4 CD2 IGLL1 CD5 TREML2 CD7 UBE2N TNFSF8 PDCD1 ADA
	Leukocyte differentiation	1.5520E-11	4.4659E-9	BCL11B BCL11A THEMIS RHOH IKZF1 CD1D GIMAP5 CD3E CD3D CBFA2T3 CD1A TSHR SPN IL4 ZAP70 LCK CD8A RAG2 ADA RAG1
	Hemopoiesis	2.9515E-8	7.5493E-6	BCL11B BCL11A THEMIS RHOH IKZF1 CD1D GIMAP5 CD3E CD3D CBFA2T3 CD1A TSHR SPN SLC25A38 IL4 ZAP70 LCK CD8A HCLS1 RAG2 ADA RAG1
	Hemopoietic or lymphoid organ development	4.4421E-8	1.0226E-5	BCL11B BCL11A CXCR5 THEMIS RHOH IKZF1 CD1D GIMAP5 CD3E CD3D CBFA2T3 CD1A TSHR SPN SLC25A38 IL4 ZAP70 LCK CD8A HCLS1 RAG2 ADA RAG1
	T cell selection	5.5893E-8	1.1697E-5	SPN ZAP70 THEMIS CD1D CD3E CD3D CD1A
	Regulation of T cell receptor signaling pathway	1.2596E-7	2.3508E-5	ELF1 LCK TRAT1 PTPN22 UBASH3A ADA
	Immune system development	1.3276E-7	2.3508E-5	BCL11B BCL11A CXCR5 THEMIS RHOH IKZF1 CD1D GIMAP5 CD3E CD3D CBFA2T3 CD1A TSHR SPN SLC25A38 IL4 ZAP70 LCK CD8A HCLS1 RAG2 ADA RAG1
	Regulation of antigen receptor-mediated signaling pathway	3.9349E-7	6.0915E-5	ELF1 LCK TRAT1 PTPN22 UBASH3A ADA
	Immune response	3.9693E-7	6.0915E-5	CD86 NOTCH1 IGHV1OR15-1 LST1 TCF7 WAS CD1E ITGAL IL2RG CD1C CORO1A CD1B FYB CD1A PSMB10 SPN TRPM2 VPREB1 CYSLTR2 ARHGDIB CCR9 RAG2 RAG1 CD96 CR2 SEMA4D LAX1 SIGIRR IL4 ZAP70 IGLL1 CD8A CD7 TNFSF8 PDCD1 ADA
	Positive regulation of immune system process	1.1442E-6	1.6462E-4	CD86 CR2 TRAT1 THEMIS IKZF1 CD1D GIMAP5 CD3E CORO1A LAX1 CD1A SPN IL4 CD2 TRPM2 ZAP70 LCK CD5 UBE2N CD38 ADA
	Positive regulation of T cell activation	1.5855E-6	2.1470E-4	SPN CD86 IL4 ZAP70 LCK CD5 IKZF1 GIMAP5 CD3E CORO1A ADA
	Positive regulation of leukocyte activation	3.0181E-6	3.8598E-4	CD86 IKZF1 GIMAP5 CD3E CORO1A SPN IL4 CD2 ZAP70 LCK CD5 CD38 ADA
	Regulation of lymphocyte	3.9854E-6	4.8286E-4	CD86 LST1 IKZF1 GIMAP5 CD3E CORO1A LAX1 SPN IL4 CD2 ZAP70 LCK CD5 CD38 ADA

	activation			
	Regulation of T cell activation	4.8483E-6	5.3146E-4	CD86 IKZF1 GIMAP5 CD3E CORO1A LAX1 SPN IL4 CD2 ZAP70 LCK CD5 ADA
	Positive regulation of cell activation	4.8483E-6	5.3146E-4	CD86 IKZF1 GIMAP5 CD3E CORO1A SPN IL4 CD2 ZAP70 LCK CD5 CD38 ADA
	Positive regulation of lymphocyte activation	5.9111E-6	6.1851E-4	SPN CD86 IL4 ZAP70 LCK CD5 CD38 IKZF1 GIMAP5 CD3E CORO1A ADA
	Positive regulation of alpha-beta T cell activation	6.1925E-6	6.1979E-4	CD86 ZAP70 IKZF1 GIMAP5 CD3E ADA
	B cell activation	7.9193E-6	7.5960E-4	CD86 IL4 BCL11A CXCR5 IKZF1 RAG2 LAX1 ADA TSHR RAG1
	Negative T cell selection	9.6036E-6	8.8430E-4	SPN ZAP70 THEMIS CD3E
	Positive regulation of T cell receptor signaling pathway	1.2556E-5	1.0736E-3	LCK TRAT1 ADA
	Regulation of biological process	1.2592E-5	1.0736E-3	ATF1 CD86 ADAMDEC1 GABPB2 VIPR2 DGKA GFI1 LST1 YEATS4 IKZF1 GIMAP5 IKZF2 RXFP2 RXFP3 IKZF5 PAIP2B HMGN5 MYC GRAP2 RASSF5 MYB ARHGDIB TBC1D10C UBASH3A LGALS9 RAG2 ZNF202 TMPO RAG1 ZBTB39 ARL11 RPS9 USP7 KDM2B GTF3A THEMIS MED4 VRK3 LAX1 PIAS1 SIGIRR RFX7 KAT6B RAB37 CD8A BTF3P12 TRIM13 PSME2 ULK1 PADI4 ERG CFTR ABCG1 BLK ZNF154 KHDRBS1 NOTCH1 AKNA CTBP1 MAX PDE1B CRABP1 TCF7 AGAP2 SUPT4H1 PRDM12 CD1D IQGAP2 BHLHE23 RASGRP2 ARHGAP15 CORO1A CD1A CDKN2AIP CPLX4 MBTD1 TRPM2 SCAP VILL ZNF664 DRD4 SCAI BTF3 BCL11B BCL11A SETD1B EAF2 ZBTB12 IL4 SEPSECS PEX5L LHX3 UBE2N TNFSF8 RGL4 GRAP ZNF775 ADA ARHGAP9 ZNF253 ITK PIWIL3 CDCA7L LECT2 PTPN22 CD3G CTCF ITGAL CD3E PHF6 PIK3CG SPN SIX6 CASP12 ZMYM2 BCL7A NETO1 CASP10 CASP6 PSD4 TAGAP CD38 ZNF124 CERS3 MAP4K1 SREBF1 DUSP2 CR2 TGIF2 RHOH TERF2 CBFA2T3 SRP9 MED28 ARFGAP2 RBL2 ZAP70 ELF1 TFDP2 LCK PEX5 PECAM1 C14ORF39 HCLS1 DSG1 NAF1 CD48 RHOU MDFI HMX1 KATNB1 CXCR4 NLRC3 ZBTB44 IL2RG AIF1 FYB PSMB10 SLC7A3 CAMKK2 FGD3 UBE2NL SLIT1 PLAGL2 PTK2B SLC25A20 MBNL3 ZNF586 ZNF740 EIF4B CYTH1 ZNF101 GDF10 FOXB1 CENPV ZNF581 SKOR2 SEMA4D USP20 UTP3 TRAT1 IRX4 LBX1 TSHR CD2 MFAP4 CD6 CD5 ZNF614 AGGF1 TPT1
	T cell differentiation in the thymus	1.8679E-5	1.5357E-3	SPN ZAP70 CD3E RAG2 CD3D RAG1
	Regulation of leukocyte activation	1.9593E-5	1.5552E-3	CD86 LST1 IKZF1 GIMAP5 CD3E CORO1A LAX1 SPN IL4 CD2 ZAP70 LCK CD5 CD38 ADA
	Regulation of immune system process	2.1754E-5	1.6693E-3	CD86 LST1 PTPN22 IKZF1 CD1D GIMAP5 CD3E CORO1A CD1A SPN TRPM2 CD38 UBASH3A CR2 TRAT1 THEMIS LAX1 IL4 CD2 ZAP70 ELF1 LCK CD5 UBE2N ADA
	Regulation of cell activation	4.1917E-5	3.1097E-3	CD86 LST1 IKZF1 GIMAP5 CD3E CORO1A LAX1 SPN IL4 CD2 ZAP70 LCK CD5 CD38 ADA
	Positive regulation of alpha-beta T cell differentiation	4.4578E-5	3.1097E-3	CD86 ZAP70 IKZF1 GIMAP5 ADA
	Positive regulation of calcium-mediated signaling	4.4578E-5	3.1097E-3	ZAP70 CD8A TRAT1 CD3E ADA
	Biological regulation	5.3658E-5	3.5847E-3	ATF1 ADAMDEC1 GABPB2 VIPR2 GFI1 LST1 IKZF1 IKZF2 RXFP2 RXFP3 IKZF5 MYC RASSF5 MYB TBC1D10C UBASH3A LGALS9 TMPO ARL11 MED4 SIGIRR RFX7 TRIM13 PSME2 ULK1 PADI4 CFTR ZNF154 CTBP1 PDE1B EPB41 CRABP1 AGAP2 SUPT4H1 PRDM12 CD1D IQGAP2

				CD1A CDKN2AIP MBTD1 TRPM2 SCAP SCAI BTF3 BCL11B BCL11A SETD1B EAF2 PEX5L TNFSF8 RGL4 GRAP ADA ARHGAP9 ZNF253 ITK CD3G CTCF CD3E PHF6 CASP12 ZMYM2 CASP10 CCR9 CD38 ZNF124 MAP4K1 SREBF1 DUSP2 RHOH TERF2 CBFA2T3 SRP9 MED28 HCLS1 CD48 RHOU MDFI HMX1 WAS CXCR4 AIF1 SLC7A3 CAMKK2 FGD3 PLAGL2 MBNL3 ZNF586 ZNF101 GDF10 FOXB1 CENPV ZNF581 UTP3 LBX1 AGGF1 CD86 DGKA YEATS4 GIMAP5 PAIP2B HMGN5 GRAP2 ARHGDIB RAG2 ZNF202 RAG1 ZBTB39 RPS9 USP7 KDM2B GTF3A THEMIS VRK3 LAX1 PIAS1 KAT6B RAB37 CD8A BTF3P12 ERG ABCG1 BLK KHDRBS1 NOTCH1 AKNA MAX TCF7 BHLHE23 RASGRP2 ARHGAP15 CORO1A CPLX4 VILL ZNF664 DRD4 ZBTB12 IL4 SEPSECS LHX3 UBE2N ZNF775 PIWIL3 CDCA7L LECT2 SLC40A1 PTPN22 ITGAL PIK3CG SPN SIX6 BCL7A NETO1 CASP6 PSD4 TAGAP CERS3 CR2 TGIF2 ARFGAP2 RBL2 ZAP70 ELF1 TFDP2 LCK PEX5 PECAM1 C14ORF39 DSG1 NAF1 KATNB1 NLRC3 ZBTB44 IL2RG FYB PSMB10 UBE2NL SLIT1 PTK2B SLC25A20 ZNF740 EIF4B CYTH1 SKOR2 SEMA4D USP20 TRAT1 IRX4 TSHR SLC25A38 CD2 MFAP4 CD6 CD5 CD7 ZNF614 TPT1
	Thymic T cell selection	5.4503E-5	3.5847E-3	SPN ZAP70 CD3E CD3D
	B cell differentiation	6.4092E-5	4.0983E-3	IL4 BCL11A IKZF1 RAG2 ADA TSHR RAG1
	Regulation of alpha-beta T cell activation	6.9013E-5	4.2938E-3	CD86 ZAP70 IKZF1 GIMAP5 CD3E ADA
	Regulation of cellular process	9.0302E-5	5.3707E-3	ATF1 CD86 ADAMDEC1 GABPB2 VIPR2 DGKA GFI1 LST1 YEATS4 IKZF1 GIMAP5 IKZF2 RXFP2 RXFP3 IKZF5 PAIP2B HMGN5 MYC GRAP2 RASSF5 MYB ARHGDIB TBC1D10C UBASH3A LGALS9 ZNF202 TMPO RAG1 ZBTB39 ARL11 RPS9 USP7 KDM2B GTF3A MED4 VRK3 LAX1 PIAS1 SIGIRR RFX7 KAT6B RAB37 CD8A BTF3P12 TRIM13 PSME2 ULK1 PADI4 ERG ABCG1 BLK ZNF154 KHDRBS1 NOTCH1 AKNA CTBP1 MAX PDE1B CRABP1 TCF7 AGAP2 SUPT4H1 PRDM12 IQGAP2 BHLHE23 RASGRP2 ARHGAP15 CORO1A CDKN2AIP CPLX4 MBTD1 TRPM2 SCAP VILL ZNF664 DRD4 SCAI BTF3 BCL11B BCL11A SETD1B EAF2 ZBTB12 IL4 SEPSECS PEX5L LHX3 UBE2N TNFSF8 RGL4 GRAP ZNF775 ADA ARHGAP9 ZNF253 ITK PIWIL3 CDCA7L LECT2 PTPN22 CD3G CTCF ITGAL CD3E PHF6 PIK3CG SPN SIX6 CASP12 ZMYM2 BCL7A NETO1 CASP10 CASP6 PSD4 TAGAP CD38 ZNF124 CERS3 MAP4K1 SREBF1 DUSP2 TGIF2 RHOH TERF2 CBFA2T3 SRP9 MED28 ARFGAP2 RBL2 ZAP70 ELF1 TFDP2 LCK PECAM1 C14ORF39 HCLS1 CD48 RHOU MDFI HMX1 KATNB1 CXCR4 NLRC3 ZBTB44 IL2RG AIF1 FYB PSMB10 SLC7A3 CAMKK2 FGD3 SLIT1 PLAGL2 PTK2B SLC25A20 MBNL3 ZNF586 ZNF740 EIF4B CYTH1 ZNF101 GDF10 FOXB1 CENPV ZNF581 SKOR2 SEMA4D TRAT1 IRX4 LBX1 TSHR CD2 MFAP4 CD6 CD5 ZNF614 AGGF1 TPT1
	Regulation of gene expression	9.0989E-5	5.3707E-3	ATF1 CD86 GABPB2 GFI1 YEATS4 IKZF1 IKZF2 IKZF5 PAIP2B HMGN5 MYC MYB ZNF202 TMPO ZBTB39 USP7 KDM2B GTF3A MED4 PIAS1 SIGIRR RFX7 KAT6B BTF3P12 PADI4 ERG ABCG1 ZNF154 KHDRBS1 NOTCH1 AKNA CTBP1 MAX TCF7 SUPT4H1 PRDM12 BHLHE23 CDKN2AIP MBTD1 SCAP ZNF664 SCAI BTF3 BCL11B BCL11A SETD1B EAF2 ZBTB12 IL4 SEPSECS LHX3 UBE2N ZNF775 ZNF253 PIWIL3 CDCA7L CTCF PHF6 SPN SIX6 ZMYM2 BCL7A ZNF124 CERS3 SREBF1 TGIF2 RHOH TERF2 CBFA2T3 SRP9 MED28 RBL2 ELF1 TFDP2 C14ORF39 HCLS1 DSG1 MDFI HMX1 NLRC3 ZBTB44 CAMKK2 SLIT1 PLAGL2 PTK2B MBNL3 ZNF586 ZNF740 EIF4B ZNF101 FOXB1 ZNF581 SKOR2 UTP3 IRX4 LBX1 ZNF614
	Regulation of calcium-mediated signaling	9.9951E-5	5.7522E-3	ZAP70 CD8A TRAT1 CD3E ADA
	Positive regulation of antigen receptor-mediated signaling pathway	1.2125E-4	6.8078E-3	LCK TRAT1 ADA
	Regulation of signaling pathway	1.5436E-4	8.4603E-3	NOTCH1 LECT2 AGAP2 CXCR4 PTPN22 IQGAP2 CD3E RASGRP2 SLC7A3 FGD3 CASP10 PSD4 TBC1D10C SLIT1 PTK2B UBASH3A LGALS9 DRD4 SCAI CYTH1 MAP4K1 DUSP2 SKOR2 USP20 TRAT1 RHOH VRK3 LAX1 ARFGAP2 SIGIRR IL4 ZAP70 PEX5L ELF1 LCK CD8A TRIM13 UBE2N HCLS1 ULK1 ADA MDFI

	Regulation of alpha-beta T cell differentiation	1.5907E-4	8.5155E-3	CD86 ZAP70 IKZF1 GIMAP5 ADA
	Positive regulation of immune response	1.6964E-4	8.8753E-3	CD86 TRPM2 ZAP70 CR2 THEMIS UBE2N CD1D GIMAP5 CD3E LAX1 ADA CD1A
	Negative thymic T cell selection	2.3831E-4	1.2191E-2	SPN ZAP70 CD3E
	Regulation of signal transduction	2.7982E-4	1.4003E-2	AGAP2 CXCR4 IQGAP2 CD3E RASGRP2 CDKN2AIP SLC7A3 FGD3 CASP10 PSD4 TBC1D10C SLIT1 PTK2B LGALS9 DRD4 SCAI CYTH1 MAP4K1 DUSP2 TRAT1 RHOH VRK3 LAX1 ARFGAP2 SIGIRR IL4 ZAP70 PEX5L CD8A TRIM13 UBE2N HCLS1 ADA MDFI
	Regulation of cellular biosynthetic process	2.9488E-4	1.4443E-2	ATF1 CD86 GABPB2 GFI1 YEATS4 IKZF1 GIMAP5 IKZF2 RXFP2 IKZF5 PAIP2B HMGN5 MYC MYB ZNF202 TMPO ZBTB39 USP7 KDM2B GTF3A MED4 PIAS1 SIGIRR RFX7 KAT6B BTF3P12 PADI4 ERG ABCG1 ZNF154 KHDRBS1 NOTCH1 AKNA CTBP1 MAX TCF7 SUPT4H1 PRDM12 BHLHE23 MBTD1 SCAP ZNF664 DRD4 SCAI BTF3 BCL11B BCL11A SETD1B EAF2 ZBTB12 IL4 SEPSECS LHX3 UBE2N ZNF775 ZNF253 PIWIL3 CDCA7L CTCF CD3E PHF6 SPN SIX6 ZMYM2 BCL7A ZNF124 CERS3 SREBF1 TGIF2 RHOH TERF2 CBFA2T3 SRP9 MED28 RBL2 ELF1 TFDP2 C14ORF39 HCLS1 MDFI HMX1 NLRC3 ZBTB44 CAMKK2 PLAGL2 PTK2B ZNF586 ZNF740 EIF4B ZNF101 FOXB1 ZNF581 SKOR2 IRX4 LBX1 TSHR ZNF614
	Regulation of lymphocyte proliferation	3.1431E-4	1.4793E-2	SPN IL4 ZAP70 CD5 LST1 CD38 CD3E CORO1A ADA
	Regulation of signaling process	3.1489E-4	1.4793E-2	AGAP2 CXCR4 IQGAP2 CD3E RASGRP2 CDKN2AIP SLC7A3 FGD3 CASP10 PSD4 TBC1D10C SLIT1 PTK2B LGALS9 DRD4 SCAI CYTH1 MAP4K1 DUSP2 TRAT1 RHOH VRK3 LAX1 ARFGAP2 SIGIRR IL4 ZAP70 PEX5L CD8A TRIM13 UBE2N HCLS1 ADA MDFI
	Regulation of macromolecule biosynthetic process	3.2753E-4	1.5080E-2	ATF1 CD86 ZNF253 GABPB2 PIWIL3 CDCA7L GFI1 YEATS4 CTCF IKZF1 IKZF2 CD3E PHF6 IKZF5 SPN PAIP2B SIX6 HMGN5 ZMYM2 BCL7A MYC MYB ZNF202 ZNF124 TMPO CERS3 ZBTB39 SREBF1 USP7 TGIF2 KDM2B GTF3A RHOH MED4 TERF2 CBFA2T3 SRP9 MED28 PIAS1 SIGIRR RBL2 ELF1 TFDP2 RFX7 KAT6B BTF3P12 C14ORF39 HCLS1 PADI4 ERG MDFI ABCG1 HMX1 ZNF154 KHDRBS1 NOTCH1 AKNA CTBP1 MAX TCF7 SUPT4H1 NLRC3 PRDM12 ZBTB44 BHLHE23 CAMKK2 MBTD1 PLAGL2 SCAP PTK2B ZNF664 ZNF586 ZNF740 EIF4B SCAI ZNF101 BTF3 FOXB1 ZNF581 SKOR2 BCL11B BCL11A SETD1B IRX4 EAF2 ZBTB12 LBX1 IL4 SEPSECS LHX3 ZNF614 UBE2N ZNF775
	Regulation of mononuclear cell proliferation	3.4060E-4	1.5374E-2	SPN IL4 ZAP70 CD5 LST1 CD38 CD3E CORO1A ADA
	Regulation of leukocyte proliferation	3.6868E-4	1.6087E-2	SPN IL4 ZAP70 CD5 LST1 CD38 CD3E CORO1A ADA
	Regulation of transcription	3.7520E-4	1.6087E-2	ATF1 CD86 ZNF253 GABPB2 CDCA7L GFI1 YEATS4 CTCF IKZF1 IKZF2 PHF6 IKZF5 SPN SIX6 HMGN5 ZMYM2 BCL7A MYC MYB ZNF202 ZNF124 TMPO CERS3 ZBTB39 SREBF1 USP7 TGIF2 KDM2B GTF3A RHOH MED4 TERF2 CBFA2T3 MED28 PIAS1 SIGIRR RBL2 ELF1 TFDP2 RFX7 KAT6B BTF3P12 C14ORF39 HCLS1 PADI4 ERG MDFI ABCG1 HMX1 ZNF154 KHDRBS1 NOTCH1 AKNA CTBP1 MAX TCF7 SUPT4H1 NLRC3 PRDM12 ZBTB44 BHLHE23 CAMKK2 MBTD1 PLAGL2 SCAP ZNF664 ZNF586 ZNF740 SCAI ZNF101 BTF3 FOXB1 ZNF581 SKOR2 BCL11B BCL11A SETD1B IRX4 EAF2 ZBTB12 LBX1 IL4 LHX3 ZNF614 UBE2N ZNF775
	Chromatin modification	3.7737E-4	1.6087E-2	CENPV KDM2B UTP3 SETD1B SUPT4H1 EAF2 YEATS4 CTCF IKZF1 TERF2 MBTD1 RBL2 HMGN5 KAT6B UBE2N PADI4 RAG2 RAG1
	Regulation of biosynthetic process	3.9379E-4	1.6266E-2	ATF1 CD86 GABPB2 GFI1 YEATS4 IKZF1 GIMAP5 IKZF2 RXFP2 IKZF5 PAIP2B HMGN5 MYC MYB ZNF202 TMPO ZBTB39 USP7 KDM2B GTF3A MED4 PIAS1 SIGIRR RFX7 KAT6B BTF3P12 PADI4 ERG ABCG1 ZNF154 KHDRBS1 NOTCH1 AKNA CTBP1 MAX TCF7 SUPT4H1 PRDM12 BHLHE23 MBTD1 SCAP ZNF664 DRD4 SCAI BTF3 BCL11B BCL11A SETD1B EAF2 ZBTB12 IL4 SEPSECS LHX3 UBE2N ZNF775 ZNF253 PIWIL3 CDCA7L CTCF CD3E PHF6 SPN SIX6 ZMYM2 BCL7A ZNF124 CERS3 SREBF1 TGIF2 RHOH TERF2 CBFA2T3 SRP9 MED28 RBL2 ELF1 TFDP2 C14ORF39 HCLS1 MDFI HMX1 NLRC3 ZBTB44 CAMKK2

				PLAGL2 PTK2B ZNF586 ZNF740 EIF4B ZNF101 FOXB1 ZNF581 SKOR2 IRX4 LBX1 TSHR ZNF614
	Negative regulation of antigen receptor-mediated signaling pathway	4.0984E-4	1.6266E-2	ELF1 PTPN22 UBASH3A
	Negative regulation of T cell receptor signaling pathway	4.0984E-4	1.6266E-2	ELF1 PTPN22 UBASH3A
	Positive T cell selection	4.0984E-4	1.6266E-2	ZAP70 THEMIS CD3D
	Positive regulation of T cell differentiation	4.2222E-4	1.6474E-2	CD86 ZAP70 IKZF1 GIMAP5 ADA
	Regulation of cell communication	4.3377E-4	1.6642E-2	ATF1 NOTCH1 LECT2 AGAP2 CXCR4 PTPN22 IQGAP2 CD3E RASGRP2 CDKN2AIP CPLX4 SLC7A3 FGD3 NETO1 CASP10 PSD4 TBC1D10C SLIT1 CD38 PTK2B UBASH3A LGALS9 DRD4 SCAI CYTH1 MAP4K1 DUSP2 SKOR2 TRAT1 RHOH VRK3 LAX1 ARFGAP2 SIGIRR IL4 ZAP70 PEX5L ELF1 LCK CD8A TRIM13 UBE2N HCLS1 ADA MDFI
	Regulation of metabolic process	4.6717E-4	1.7630E-2	ATF1 CD86 GABPB2 DGKA GFI1 YEATS4 IKZF1 GIMAP5 IKZF2 RXFP2 IKZF5 PAIP2B HMGN5 MYC MYB TBC1D10C ZNF202 TMPO ZBTB39 USP7 KDM2B GTF3A MED4 VRK3 LAX1 PIAS1 SIGIRR RFX7 KAT6B BTF3P12 PSME2 ULK1 PADI4 ERG ABCG1 ZNF154 KHDRBS1 NOTCH1 AKNA CTBP1 MAX TCF7 AGAP2 SUPT4H1 PRDM12 BHLHE23 CDKN2AIP MBTD1 SCAP ZNF664 DRD4 SCAI BTF3 BCL11B BCL11A SETD1B EAF2 ZBTB12 IL4 SEPSECS LHX3 UBE2N ZNF775 ZNF253 PIWIL3 CDCA7L CTCF CD3E PHF6 SPN SIX6 ZMYM2 BCL7A ZNF124 CERS3 MAP4K1 SREBF1 DUSP2 TGIF2 RHOH TERF2 CBFA2T3 SRP9 MED28 ARFGAP2 RBL2 ELF1 TFDP2 C14ORF39 HCLS1 DSG1 MDFI HMX1 CXCR4 NLRC3 ZBTB44 PSMB10 CAMKK2 FGD3 UBE2NL SLIT1 PLAGL2 PTK2B SLC25A20 MBNL3 ZNF586 ZNF740 EIF4B ZNF101 FOXB1 ZNF581 SKOR2 UTP3 TRAT1 IRX4 LBX1 TSHR CD6 ZNF614
	Antigen processing and presentation, endogenous lipid antigen via MHC class Ib	5.4182E-4	1.9383E-2	CD1D CD1A
	Purine nucleoside monophosphate biosynthetic process	5.4276E-4	1.9383E-2	CECR1 AMPD3 PAICS ADA
	Purine ribonucleoside monophosphate biosynthetic process	5.4276E-4	1.9383E-2	CECR1 AMPD3 PAICS ADA
	Regulation of cellular metabolic process	5.4732E-4	1.9383E-2	ATF1 CD86 GABPB2 DGKA GFI1 YEATS4 IKZF1 GIMAP5 IKZF2 RXFP2 IKZF5 PAIP2B HMGN5 MYC MYB TBC1D10C ZNF202 TMPO ZBTB39 USP7 KDM2B GTF3A MED4 VRK3 LAX1 PIAS1 SIGIRR RFX7 KAT6B BTF3P12 PSME2 ULK1 PADI4 ERG ABCG1 ZNF154 KHDRBS1 NOTCH1 AKNA CTBP1 MAX TCF7 AGAP2 SUPT4H1 PRDM12 BHLHE23 MBTD1 SCAP ZNF664 DRD4 SCAI BTF3 BCL11B BCL11A SETD1B EAF2 ZBTB12 IL4 SEPSECS LHX3 UBE2N ZNF775 ZNF253 PIWIL3 CDCA7L CTCF CD3E PHF6 SPN SIX6 ZMYM2 BCL7A ZNF124 CERS3 MAP4K1 SREBF1 DUSP2 TGIF2 RHOH TERF2 CBFA2T3 SRP9 MED28 ARFGAP2 RBL2 ELF1 TFDP2 C14ORF39 HCLS1 MDFI HMX1 CXCR4 NLRC3 ZBTB44 PSMB10 CAMKK2 FGD3 PLAGL2 PTK2B SLC25A20 MBNL3 ZNF586 ZNF740 EIF4B ZNF101 FOXB1 ZNF581 SKOR2 TRAT1 IRX4 LBX1 TSHR CD6 ZNF614
	Regulation of nitrogen compound	5.8961E-4	2.0565E-2	ATF1 CD86 ZNF253 GABPB2 CDCA7L GFI1 YEATS4 CTCF IKZF1 GIMAP5 IKZF2 PHF6 RXFP2 IKZF5 SPN SIX6 HMGN5 ZMYM2 BCL7A MYC MYB TBC1D10C ZNF202 ZNF124 TMPO CERS3 ZBTB39 SREBF1

	metabolic process			USP7 TGIF2 KDM2B GTF3A RHOH MED4 TERF2 CBFA2T3 MED28 PIAS1 ARFGAP2 SIGIRR RBL2 ELF1 TFDP2 RFX7 KAT6B BTF3P12 C14ORF39 HCLS1 PADI4 ERG MDFI ABCG1 HMX1 ZNF154 KHDRBS1 NOTCH1 AKNA CTBP1 MAX TCF7 AGAP2 SUPT4H1 NLRC3 PRDM12 ZBTB44 BHLHE23 CAMKK2 FGD3 MBTD1 PLAGL2 SCAP PTK2B MBNL3 ZNF664 ZNF586 DRD4 ZNF740 SCAI ZNF101 BTF3 FOXB1 ZNF581 SKOR2 BCL11B BCL11A SETD1B IRX4 EAF2 ZBTB12 LBX1 TSHR IL4 LHX3 ZNF614 UBE2N ZNF775
	Regulation of nucleobase, nucleoside, nucleotide and nucleic acid metabolic process	6.8067E-4	2.3280E-2	ATF1 CD86 ZNF253 GABPB2 CDCA7L GFI1 YEATS4 CTCF IKZF1 IKZF2 PHF6 RXFP2 IKZF5 SPN SIX6 HMGN5 ZMYM2 BCL7A MYC MYB TBC1D10C ZNF202 ZNF124 TMPO CERS3 ZBTB39 SREBF1 USP7 TGIF2 KDM2B GTF3A RHOH MED4 TERF2 CBFA2T3 MED28 PIAS1 ARFGAP2 SIGIRR RBL2 ELF1 TFDP2 RFX7 KAT6B BTF3P12 C14ORF39 HCLS1 PADI4 ERG MDFI ABCG1 HMX1 ZNF154 KHDRBS1 NOTCH1 AKNA CTBP1 MAX TCF7 AGAP2 SUPT4H1 NLRC3 PRDM12 ZBTB44 BHLHE23 CAMKK2 FGD3 MBTD1 PLAGL2 SCAP PTK2B MBNL3 ZNF664 ZNF586 DRD4 ZNF740 SCAI ZNF101 BTF3 FOXB1 ZNF581 SKOR2 BCL11B BCL11A SETD1B IRX4 EAF2 ZBTB12 LBX1 TSHR IL4 LHX3 ZNF614 UBE2N ZNF775
	Positive regulation of lymphocyte differentiation	6.8935E-4	2.3280E-2	CD86 ZAP70 IKZF1 GIMAP5 ADA
	Positive regulation of lymphocyte proliferation	6.9779E-4	2.3280E-2	SPN IL4 ZAP70 CD38 CD3E CORO1A ADA
	Positive regulation of mononuclear cell proliferation	7.6665E-4	2.5212E-2	SPN IL4 ZAP70 CD38 CD3E CORO1A ADA
	Regulation of macromolecule metabolic process	7.8613E-4	2.5488E-2	ATF1 CD86 GABPB2 GFI1 YEATS4 IKZF1 IKZF2 IKZF5 PAIP2B HMGN5 MYC MYB ZNF202 TMPO ZBTB39 USP7 KDM2B GTF3A MED4 PIAS1 SIGIRR RFX7 KAT6B BTF3P12 PSME2 PADI4 ERG ABCG1 ZNF154 KHDRBS1 NOTCH1 AKNA CTBP1 MAX TCF7 SUPT4H1 PRDM12 BHLHE23 CDKN2AIP MBTD1 SCAP ZNF664 SCAI BTF3 BCL11B BCL11A SETD1B EAF2 ZBTB12 IL4 SEPSECS LHX3 UBE2N ZNF775 ZNF253 PIWIL3 CDCA7L CTCF CD3E PHF6 SPN SIX6 ZMYM2 BCL7A ZNF124 CERS3 MAP4K1 SREBF1 TGIF2 RHOH TERF2 CBFA2T3 SRP9 MED28 RBL2 ELF1 TFDP2 C14ORF39 HCLS1 DSG1 MDFI HMX1 NLRC3 ZBTB44 PSMB10 CAMKK2 UBE2NL SLIT1 PLAGL2 PTK2B MBNL3 ZNF586 ZNF740 EIF4B ZNF101 FOXB1 ZNF581 SKOR2 UTP3 TRAT1 IRX4 LBX1 CD6 ZNF614
	Regulation of T cell differentiation	8.2471E-4	2.6150E-2	CD86 CD2 ZAP70 IKZF1 GIMAP5 ADA
	Positive regulation of leukocyte proliferation	8.4077E-4	2.6150E-2	SPN IL4 ZAP70 CD38 CD3E CORO1A ADA
	Purine nucleoside monophosphate metabolic process	8.5196E-4	2.6150E-2	CECR1 AMPD3 PAICS ADA
	Purine ribonucleoside monophosphate metabolic process	8.5196E-4	2.6150E-2	CECR1 AMPD3 PAICS ADA
	Regulation of primary metabolic process	9.8357E-4	2.9792E-2	ATF1 CD86 GABPB2 GFI1 YEATS4 IKZF1 GIMAP5 IKZF2 RXFP2 IKZF5 PAIP2B HMGN5 MYC MYB TBC1D10C ZNF202 TMPO ZBTB39 USP7 KDM2B GTF3A MED4 PIAS1 SIGIRR RFX7 KAT6B BTF3P12 PSME2 PADI4 ERG ABCG1 ZNF154 KHDRBS1 NOTCH1 AKNA CTBP1 MAX TCF7 AGAP2 SUPT4H1 PRDM12 BHLHE23 MBTD1 SCAP ZNF664 DRD4 SCAI BTF3 BCL11B BCL11A SETD1B EAF2 ZBTB12 IL4 SEPSECS LHX3 UBE2N ZNF775 ZNF253 PIWIL3 CDCA7L CTCF CD3E PHF6 SPN SIX6 ZMYM2 BCL7A ZNF124 CERS3 MAP4K1 SREBF1 TGIF2 RHOH TERF2 CBFA2T3 SRP9 MED28 ARFGAP2 RBL2 ELF1 TFDP2 C14ORF39 HCLS1 MDFI HMX1 NLRC3 ZBTB44 PSMB10 CAMKK2 FGD3 UBE2NL PLAGL2

				PTK2B SLC25A20 MBNL3 ZNF586 ZNF740 EIF4B ZNF101 FOXB1 ZNF581 SKOR2 IRX4 LBX1 TSHR CD6 ZNF614
	Antigen receptor-mediated signaling pathway	1.2205E-3	3.6487E-2	ZAP70 THEMIS UBE2N CD3E LAX1
	Negative regulation of immune system process	1.2701E-3	3.7485E-2	SPN ELF1 LST1 PTPN22 UBASH3A GIMAP5 LAX1 ADA
	Regulation of immune response	1.3090E-3	3.8144E-2	CD86 CR2 THEMIS CD1D GIMAP5 CD3E LAX1 CD1A SPN IL4 TRPM2 ZAP70 UBE2N ADA
	V(D)J recombination	1.3338E-3	3.8379E-2	BCL11B RAG2 RAG1
	Negative regulation of thymocyte apoptosis	1.6003E-3	4.2837E-2	ADA RAG1
	Cellular response to sterol depletion	1.6003E-3	4.2837E-2	INSIG1 SCAP
	ER-nuclear sterol response pathway	1.6003E-3	4.2837E-2	INSIG1 SCAP
	Pre-B cell allelic exclusion	1.6003E-3	4.2837E-2	RAG2 RAG1
	Response to sterol depletion	1.6003E-3	4.2837E-2	INSIG1 SCAP
	Antigen processing and presentation of lipid antigen via MHC class Ib	1.6003E-3	4.2837E-2	CD1D CD1A
	Regulation of developmental process	1.6664E-3	4.3972E-2	ATF1 CD86 NOTCH1 GFI1 LST1 KATNB1 IKZF1 GIMAP5 BHLHE23 ARHGAP15 CORO1A FGD3 SPN TRPM2 CASP6 MYC SLIT1 PTK2B MBNL3 GDF10 TGIF2 SEMA4D LBX1 PIAS1 IL4 CD2 ZAP70 AGGF1 ULK1 RHOU ADA ABCG1
	Chromatin organization	1.6932E-3	4.3972E-2	CENPV KDM2B UTP3 SETD1B SUPT4H1 EAF2 NAP1L2 YEATS4 CTCF IKZF1 TERF2 MBTD1 RBL2 HMGN5 KAT6B UBE2N TSSK6 PADI4 RAG2 RAG1
	Immune response-activating signal transduction	1.7000E-3	4.3972E-2	CD86 ZAP70 THEMIS UBE2N CD3E LAX1
	Activation of immune response	1.7929E-3	4.5859E-2	CD86 TRPM2 ZAP70 CR2 THEMIS UBE2N CD3E LAX1
	Regulation of lymphocyte differentiation	1.8674E-3	4.7239E-2	CD86 CD2 ZAP70 IKZF1 GIMAP5 ADA
**12**	Regulation of immune system process	1.2657E-6	4.0122E-3	FCN2 CR2 SPI1 AMBPTNFSF14 PRKCBTESCZBTB16 CARD9 LST1 LMO2 SIRPB2 IKZF1 CORO1AINSF7 TRPM2 CD4 TBX21 TLR9 SPACA3 CD38 CD37 TCF3
	Cytosolic calcium ion homeostasis	2.6988E-6	4.2776E-3	JPH4 CD52 CCKBRHRH3 P2RY1 CD38 CXCR4 PTK2BKCNA5 GHRLADRA1ARXFP3
	Elevation of cytosolic calcium ion concentration	7.1470E-6	5.8581E-3	JPH4 CD52 CCKBR HRH3 P2RY1 CD38 CXCR4 PTK2B GHRL ADRA1A RXFP3
	Cellular calcium ion homeostasis	8.4571E-6	5.8581E-3	CD52 CXCR4 KCNA5 CACNA1FADRA1ARXFP3 JPH4 CCKBRHRH3 P2RY1 CD38 PTK2BGHRLDRD4
	Positive regulation of	9.2400E-6	5.8581E-3	FCN2 CR2 TNFSF14 PRKCB CARD9 SIRPB2 IKZF1 CORO1A F7 TRPM2 CD4 TBX21 TLR9 SPACA3 CD38 CD37 TCF3

	immune system process			
	Calcium ion homeostasis	1.2950E-5	6.8417E-3	CD52 CXCR4 KCNA5 CACNA1FADRA1ARXFP3 JPH4 CCKBRHRH3 P2RY1 CD38 PTK2BGHRLDRD4
	Cellular metal ion homeostasis	1.6369E-5	7.4128E-3	CD52 CXCR4 KCNA5 CACNA1FADRA1ARXFP3 JPH4 CCKBRHRH3 P2RY1 CD38 PTK2BGHRLDRD4
	Metal ion homeostasis	2.8622E-5	1.1342E-2	CD52 CXCR4 KCNA5 CACNA1FADRA1ARXFP3 JPH4 CCKBRHRH3 P2RY1 CD38 PTK2BGHRLDRD4
	Defense response	3.4928E-5	1.2172E-2	FCN2 CD84 STAB2 WAS CXCR4 KLK3 LSP1 MEFV ITGAL CORO1A AIF1 INS NEUROD2 TRPM2 PENK DRD4 CCL25 CR2 CARD9 NFATC3 LILRB2 OPRM1 CCKBR P2RX1 TLR9 SPACA3 CD48
	Di-, tri-valent inorganic cation homeostasis	4.1890E-5	1.2172E-2	CD52 CXCR4 KCNA5 CACNA1FADRA1ARXFP3 JPH4 CCKBRHRH3 P2RY1 CD38 PTK2BGHRLDRD4 CUTC
	Regulation of postsynaptic membrane potential	4.2236E-5	1.2172E-2	CHRNA4 GHRL OPRM1 GRIN2C DRD4
	Cellular ion homeostasis	6.2215E-5	1.6435E-2	CD52 CHRNA4 CXCR4 KCNA5 OPRM1 GRIN2CCACNA1FADRA1ARXFP3 JPH4 P2RX3 CCKBRHRH3 P2RX1 P2RY1 CD38 PTK2BGHRLDRD4
	Ion homeostasis	7.1082E-5	1.6681E-2	CD52 CHRNA4 CXCR4 KCNA5 OPRM1 GRIN2CCACNA1FADRA1ARXFP3 JPH4 P2RX3 CCKBRHRH3 P2RX1 P2RY1 CD38 PTK2BGHRLDRD4 CUTC
	Cellular chemical homeostasis	7.7015E-5	1.6681E-2	CD52 CHRNA4 CXCR4 KCNA5 OPRM1 GRIN2CCACNA1FADRA1ARXFP3 JPH4 P2RX3 CCKBRHRH3 P2RX1 P2RY1 CD38 PTK2BGHRLDRD4
	Cellular di-, tri-valent inorganic cation homeostasis	7.8932E-5	1.6681E-2	CD52 CXCR4 KCNA5 CACNA1FADRA1ARXFP3 JPH4 CCKBRHRH3 P2RY1 CD38 PTK2BGHRLDRD4
	Behavioral defense response	9.8494E-5	1.9068E-2	NEUROD2 CCKBR PENK DRD4
	Response to hypoxia	1.0266E-4	1.9068E-2	ANGPT4 HLFP2RX3 RBP3 CHRNA4 GPR182 CD38 CXCR4 PTK2BKCNA5 LCT
	Homeostatic process	1.0827E-4	1.9068E-2	HLFCHRNA4 CXCR4 KCNA5 PLGGLRXADRB1 CACNA1FCORO1AADRA1ARXFP3 INSJPH4 HRH3 P2RY1 CD38 PTK2BDRD4 CD52 TNFSF14 OPRM1 GRIN2CTERF2 P2RX3 CCKBRP2RX1 TLR9 GHRLCUTCABCG1
	Chemical homeostasis	1.3690E-4	2.2840E-2	CD52 HLFCHRNA4 CXCR4 KCNA5 OPRM1 GRIN2CCACNA1FADRA1ARXFP3 INSJPH4 P2RX3 CCKBRHRH3 P2RX1 P2RY1 CD38 PTK2BGHRLDRD4 CUTCABCG1
	Insoluble fraction	1.5132E-4	2.2988E-2	CHRNA4 RGS16 LST1 MLC1 GPTLIN7APLGADRB1 RASGRP2 SLC6A2 RASGRP4 GYS2 HRH3 CD38 NCKAP1LCD52 SYT2 AMBPSLC12A1 LILRB2 OPRM1 CYP4F8 F7 CYP2C8 CYP26C1 P2RX3 CCKBRP2RX1 CEACAM4 ULK1 LCT
	Cellular homeostasis	1.6408E-4	2.2988E-2	CD52 CHRNA4 CXCR4 KCNA5 PLGGLRXOPRM1 GRIN2CCACNA1FADRA1ARXFP3 JPH4 P2RX3 CCKBRHRH3 P2RX1 P2RY1 CD38 PTK2BGHRLDRD4
	Immune system process	1.6711E-4	2.2988E-2	FCN2 IGSF6 HLFCHRNA4 LST1 WASNLRC3 IKZF1 ITGALIL2RGCORO1ARASGRP4 INSTRPM2 CCL25 CR2 TNFSF14 PRKCBZBTB16 CARD9 LMO2 LILRB2 OPRM1 CBFA2T3 CD4 IGLL1 TREML2 PECAM1 TLR9 SPACA3 TNFSF8 CD48 PDCD1 TCF3
	Response to oxygen levels	1.7278E-4	2.2988E-2	ANGPT4 HLFP2RX3 RBP3 CHRNA4 GPR182 CD38 CXCR4 PTK2BKCNA5 LCT
	Membrane fraction	1.7656E-4	2.2988E-2	CHRNA4 RGS16 LST1 MLC1 GPTLIN7APLGADRB1 RASGRP2 SLC6A2 RASGRP4 HRH3 CD38 NCKAP1LCD52 SYT2 AMBPSLC12A1 LILRB2 OPRM1 CYP4F8 F7 CYP2C8 CYP26C1 P2RX3 CCKBRP2RX1 CEACAM4 ULK1 LCT
	Lymphocyte activation	1.8129E-4	2.2988E-2	CD4 TNFSF14 PRKCB CHRNA4 TREML2 WAS NLRC3 CD48 IKZF1 TCF3 ITGAL INS
	Cell activation	1.9178E-4	2.3382E-2	TNFSF14 PRKCB CHRNA4 KRT2 WAS NLRC3 IKZF1 ITGAL INS CD4 P2RX1 TREML2 SPACA3 CD48 TCF3
	Fear response	2.2415E-4	2.6316E-2	NEUROD2 PENK ADRB1 DRD4
	Cellular cation homeostasis	2.4830E-4	2.8112E-2	CD52 CXCR4 KCNA5 CACNA1FADRA1ARXFP3 JPH4 CCKBRHRH3 P2RY1 CD38 PTK2BGHRLDRD4

	Multicellular organismal response to stress	2.7763E-4	2.9768E-2	NEUROD2 P2RX3 PENK ADRB1 DRD4
	Cation homeostasis	2.8171E-4	2.9768E-2	CD52 CXCR4 KCNA5 CACNA1FADRA1ARXFP3 JPH4 CCKBRHRH3 P2RY1 CD38 PTK2BGHRLDRD4 CUTC
	Positive regulation of leukocyte activation	3.0070E-4	3.0749E-2	CD4 TNFSF14 TBX21 SPACA3 CD38 SIRPB2 IKZF1 TCF3 CORO1A
	T cell activation	3.6358E-4	3.6017E-2	CD4 TNFSF14 TREML2 WAS NLRC3 CD48 IKZF1 ITGAL INS
	Guanyl-nucleotide exchange factor activity	3.8604E-4	3.7084E-2	PSD DOCK11 ARHGEF15 DOCK8 TAGAP CHAT RGL4 RASGRP2 RASGRP4 ARHGEF6
	Leukocyte activation	4.0924E-4	3.7247E-2	TNFSF14 PRKCB CHRNA4 WAS NLRC3 IKZF1 ITGAL INS CD4 TREML2 SPACA3 CD48 TCF3
	Positive regulation of cell activation	4.1124E-4	3.7247E-2	CD4 TNFSF14 TBX21 SPACA3 CD38 SIRPB2 IKZF1 TCF3 CORO1A
**13**	Calcitonin binding	1.1242E-4	6.8949E-3	CALCR
	Regulation of N-terminal protein palmitoylation	1.1242E-4	6.8949E-3	HHATL
	Negative regulation of N-terminal protein palmitoylation	1.1242E-4	6.8949E-3	HHATL
	Calcitonin receptor activity	3.3723E-4	1.5513E-2	CALCR
	Negative regulation of lipoprotein metabolic process	5.6202E-4	2.0682E-2	HHATL
	Regulation of lipoprotein metabolic process	7.8678E-4	2.4128E-2	HHATL
	Osteoclast differentiation	1.0115E-3	2.6589E-2	CALCR
	Microtubule-based flagellum part	1.3486E-3	2.7571E-2	CALCR
	Flagellum part	1.3486E-3	2.7571E-2	CALCR
	Negative regulation of ossification	1.9102E-3	3.5148E-2	CALCR
	Microtubule-based flagellum	2.6962E-3	4.1340E-2	CALCR
	Regulation of mrna stability	2.8085E-3	4.1340E-2	CALCR
	Regulation of RNA stability	2.9208E-3	4.1340E-2	CALCR
	Peptide hormone binding	3.2575E-3	4.2813E-2	CALCR
	Flagellum	4.0430E-3	4.3388E-2	CALCR
	Myeloid leukocyte differentiation	4.2674E-3	4.3388E-2	CALCR
	Activation of adenylate	4.6039E-3	4.3388E-2	CALCR

	cyclase activity by G-protein signaling pathway			
	Regulation of adenylate cyclase activity involved in G-protein signaling pathway	4.6039E-3	4.3388E-2	CALCR
	Positive regulation of adenylate cyclase activity by G-protein signaling pathway	4.6039E-3	4.3388E-2	CALCR
	Acrosomal vesicle	4.7160E-3	4.3388E-2	CALCR
	Hormone binding	5.8373E-3	4.9561E-2	CALCR
	Activation of adenylate cyclase activity	6.2856E-3	4.9561E-2	CALCR
	Positive regulation of adenylate cyclase activity	6.3976E-3	4.9561E-2	CALCR
	Positive regulation of cyclase activity	6.5097E-3	4.9561E-2	CALCR
	Positive regulation of lyase activity	6.7338E-3	4.9561E-2	CALCR
**27**	–	–	–	–
**29**	Intracellular membrane-bounded organelle	6.9338E-14	9.9785E-11	RPL3 SPI1 TFRC EHMT2 THYN1 HNRNPR IKZF5 UXT SNRPD1 MYC CHEK2 MYB ZNF845 GLUL EIF2A RPS12 SMARCC1 ENDOG ATP6V1G2 ESCO1 ZNF160 OXSM METTL3 SYTL1 LARS2 ISL2 EWSR1 PDE12 CLNS1A BTK PADI4 SF1 ZNF397 GCDH ABCB7 EPB41 MRPL16 MTMR8 AAAS ZBTB3 MRPL11 MBTD1 DHX30 AFMID PCBP2 HESX1 ST8SIA4 SCAP ZNF700 PATZ1 SIAH2 SRPK1 MED13L RPS25 POLA1 INTS5 INTS4 PSMG2 ADCK3 HNRNPA1L2 ZNF136 TOP2B GMEB1 ZNF493 SUV39H1 CASP14 MRPL42 ZNF808 SCML2 ZNF41 DLAT OFD1 NDC1 ACAD8 COX8A MRPS25 STRBP TET1 TMC8 MRPS21 CBFA2T3 GNL3 DDB1 RPUSD4 TAF6L BDH1 PDIK1L ZADH2 TLR9 TUT1 PTMA SLC29A2 MYCBP RPL11 ATP5A1 AIF1 EXOSC7 HIRA ZNF589 METTL7A RPL17 LAPTM5 GTF2H4 DEF6 SPCS2 ABCB10 LYRM4 LYRM7 NDUFAB1 PKN1 NCAPD3 CD86 KDM1A ZBTB26 UBP1 TSEN2 RPL10A BZRAP1 MACROD1 KPNA5 ZNF445 HMGN2 HADH MEN1 ZBTB39 RPS9 KDM2B HIST1H2AK LMO2 GTF3A VRK1 RPSA SDHD EMSY MAPKAPK3 POLG2 GEMIN8 SRSF9 FKBP5 ZNF551 KHDRBS1 MAX NDUFB11 ARHGAP19 CORO1A ATXN3 H1FX NDUFV2 MDN1 TRAPPC2 DNAJC19 PDHA1 NEK4 ANKRD23 SELP PAN2 GNL3L TAF4B STT3B RSL24D1 CDCA7L HP1BP3 ATP2A3 LRMP LYL1 ZNRD1 TGIF2 GLRX5 DDX51 LSS PTP4A2 HSH2D SLC25A19 ZNF639 PEX6 PCCB PECAM1 SLC25A6 ATF6B HDAC1 ZBTB48 VPRBP PDHB RETN RNF5 HDAC6 ZBTB40 RBBP4 RBBP8 RBBP7 VHL CLASP2 GALNT6 NDUFA5 ZNF184 PNRC2 SLC25A38 SNRNP40 EIF3L ZNF614 PDCD2 MMACHC
	Membrane-bounded organelle	7.6817E-14	9.9785E-11	RPL3 SPI1 TFRC EHMT2 THYN1 HNRNPR IKZF5 UXT SNRPD1 MYC CHEK2 MYB ZNF845 GLUL EIF2A RPS12 SMARCC1 ENDOG ATP6V1G2 ESCO1 ZNF160 OXSM METTL3 SYTL1 LARS2 ISL2 EWSR1 PDE12 CLNS1A BTK PADI4 SF1 ZNF397 GCDH ABCB7 EPB41 MRPL16 MTMR8 AAAS ZBTB3 MRPL11 MBTD1 DHX30 AFMID PCBP2 HESX1 ST8SIA4 SCAP ZNF700 PATZ1 SIAH2 SRPK1 MED13L RPS25 POLA1 INTS5 INTS4 PSMG2 ADCK3 HNRNPA1L2 ZNF136 TOP2B GMEB1 ZNF493 SUV39H1 CASP14 MRPL42 ZNF808 SCML2 ZNF41 DLAT OFD1 NDC1 ACAD8 COX8A MRPS25 STRBP TET1 TMC8 MRPS21 CBFA2T3 GNL3 DDB1 RPUSD4 TAF6L BDH1 PDIK1L ZADH2 TLR9 TUT1 PTMA SLC29A2 MYCBP RPL11 ATP5A1 AIF1 EXOSC7 HIRA ZNF589 METTL7A RPL17

				LAPTM5 GTF2H4 DEF6 SPCS2 ABCB10 LYRM4 LYRM7 NDUFAB1 PKN1 NCAPD3 CD86 KDM1A ZBTB26 UBP1 TSEN2 RPL10A BZRAP1 MACROD1 KPNA5 ZNF445 HMGN2 HADH MEN1 ZBTB39 RPS9 KDM2B HIST1H2AK LMO2 GTF3A VRK1 RPSA SDHD EMSY MAPKAPK3 POLG2 GEMIN8 SRSF9 FKBP5 ZNF551 KHDRBS1 MAX NDUFB11 ARHGAP19 CORO1A ATXN3 H1FX NDUFV2 MDN1 TRAPPC2 DNAJC19 PDHA1 NEK4 ANKRD23 SELP PAN2 GNL3L TAF4B STT3B RSL24D1 CDCA7L HP1BP3 ATP2A3 LRMP LYL1 ZNRD1 TGIF2 GLRX5 DDX51 LSS PTP4A2 HSH2D SLC25A19 ZNF639 PEX6 PCCB PECAM1 SLC25A6 ATF6B HDAC1 ZBTB48 VPRBP PDHB RETN RNF5 HDAC6 ZBTB40 RBBP4 RBBP8 RBBP7 VHL CLASP2 GALNT6 NDUFA5 ZNF184 PNRC2 SLC25A38 SNRNP40 EIF3L ZNF614 PDCD2 MMACHC
	Intracellular	1.9578E-13	1.3950E-10	RPL3 SPI1 TFRC EHMT2 THYN1 HNRNPR NUDT3 IKZF5 UXT FRAT1 SNRPD1 MYC CHEK2 MYB ZNF845 ZNF720 RPS10 GLUL EIF2A RPS12 SMARCC1 ENDOG ATP6V1G2 ESCO1 ZNF160 OXSM RPL22 METTL3 SYTL1 PHKA2 RNF123 LARS2 ISL2 EWSR1 PDE12 CLNS1A BTK C3ORF20 PADI4 RPL29 GTSF1 SF1 ZNF397 GCDH ABCB7 EPB41 PDE1B MRPL16 MTMR8 VPS26B AAAS IQGAP2 ZBTB3 MRPL11 MBTD1 DHX30 AFMID PCBP2 HESX1 ST8SIA4 SCAP ZNF700 CEP192 PATZ1 SIAH2 SRPK1 MED13L RPS25 POLA1 INTS5 INTS4 PSMG2 ADCK3 HNRNPA1L2 ZNF136 ARHGAP9 TOP2B GMEB1 ZNF493 SUV39H1 CASP14 MRPL42 ZNF808 SCML2 ZNF41 DLAT OFD1 NDC1 ACAD8 COX8A MRPS25 STRBP TET1 TMC8 MRPS21 CBFA2T3 GNL3 DDB1 RPUSD4 TAF6L KIF9 BDH1 PDIK1L ZADH2 TLR9 TUT1 PTMA SLC29A2 MYCBP RPL11 ATP5A1 ASIP AIF1 CAMKK2 EXOSC7 HIRA RNF138 RPL14 ZNF589 METTL7A RPL17 LAPTM5 GTF2H4 DEF6 SPCS2 ABCB10 LYRM4 LYRM7 NDUFAB1 PKN1 NCAPD3 CD86 KDM1A ZBTB26 UBP1 TSEN2 RPL10A BZRAP1 MACROD1 KPNA5 ZNF445 HMGN2 HADH MEN1 ZBTB39 RPS9 KDM2B HIST1H2AK LMO2 GTF3A VRK1 RPSA SDHD EMSY MAPKAPK3 POLG2 CARD17 GEMIN8 SRSF9 FKBP5 ZNF551 KHDRBS1 MAX NDUFB11 ARHGAP19 RASGRP2 CORO1A DPP3 ATXN3 H1FX NDUFV2 MDN1 TRAPPC2 DNAJC19 PDHA1 NEK4 ANKRD23 SELP PAN2 RPS10P5 GNL3L TAF4B STT3B RSL24D1 CDCA7L AMD1 NGLY1 HP1BP3 ATP2A3 LRMP LSP1 LYL1 ZNRD1 TAGAP PGPEP1 ADSL TGIF2 ACTR6 GLRX5 SH2D3C ABHD14A DDX51 LSS PTP4A2 HSH2D SLC25A19 ZNF639 PEX6 PCCB PECAM1 SLC25A6 EEF1A1P5 ATF6B HDAC1 ZBTB48 VPRBP TULP2 PDHB RETN RNF5 HDAC6 ZBTB40 RBBP4 RBBP8 RBBP7 VHL DCAF11 EIF4B CLASP2 GALNT6 NDUFA5 ZNF184 USP20 RPL23A ACVR2B PNRC2 SLC25A38 RPIA SNRNP40 EIF3L IMPDH2 ZNF614 PDCD2 ZNF852 MMACHC
	Intracellular part	2.1478E-13	1.3950E-10	RPL3 SPI1 TFRC EHMT2 THYN1 HNRNPR NUDT3 IKZF5 UXT FRAT1 SNRPD1 MYC CHEK2 MYB ZNF845 RPS10 GLUL EIF2A RPS12 SMARCC1 ENDOG ATP6V1G2 ESCO1 ZNF160 OXSM RPL22 METTL3 SYTL1 PHKA2 RNF123 LARS2 ISL2 EWSR1 PDE12 CLNS1A BTK C3ORF20 PADI4 RPL29 GTSF1 SF1 ZNF397 GCDH ABCB7 EPB41 PDE1B MRPL16 MTMR8 VPS26B AAAS IQGAP2 ZBTB3 MRPL11 MBTD1 DHX30 AFMID PCBP2 HESX1 ST8SIA4 SCAP ZNF700 CEP192 PATZ1 SIAH2 SRPK1 MED13L RPS25 POLA1 INTS5 INTS4 PSMG2 ADCK3 HNRNPA1L2 ZNF136 TOP2B GMEB1 ZNF493 SUV39H1 CASP14 MRPL42 ZNF808 SCML2 ZNF41 DLAT OFD1 NDC1 ACAD8 COX8A MRPS25 STRBP TET1 TMC8 MRPS21 CBFA2T3 GNL3 DDB1 RPUSD4 TAF6L KIF9 BDH1 PDIK1L ZADH2 TLR9 TUT1 PTMA SLC29A2 MYCBP RPL11 ATP5A1 ASIP AIF1 CAMKK2 EXOSC7 HIRA RPL14 ZNF589 METTL7A RPL17 LAPTM5 GTF2H4 DEF6 SPCS2 ABCB10 LYRM4 LYRM7 NDUFAB1 PKN1 NCAPD3 CD86 KDM1A ZBTB26 UBP1 TSEN2 RPL10A BZRAP1 MACROD1 KPNA5 ZNF445 HMGN2 HADH MEN1 ZBTB39 RPS9 KDM2B HIST1H2AK LMO2 GTF3A VRK1 RPSA SDHD EMSY MAPKAPK3 POLG2 CARD17 GEMIN8 SRSF9 FKBP5 ZNF551 KHDRBS1 MAX NDUFB11 ARHGAP19 RASGRP2 CORO1A DPP3 ATXN3 H1FX NDUFV2 MDN1 TRAPPC2 DNAJC19 PDHA1 NEK4 ANKRD23 SELP PAN2 RPS10P5 GNL3L TAF4B STT3B RSL24D1 CDCA7L AMD1 NGLY1 HP1BP3 ATP2A3 LRMP LSP1 LYL1 ZNRD1 PGPEP1 ADSL TGIF2 ACTR6 GLRX5 SH2D3C ABHD14A DDX51 LSS PTP4A2 HSH2D SLC25A19 ZNF639 PEX6 PCCB PECAM1 SLC25A6 EEF1A1P5 ATF6B HDAC1 ZBTB48 VPRBP TULP2 PDHB RETN RNF5 HDAC6 ZBTB40 RBBP4 RBBP8 RBBP7 VHL DCAF11 EIF4B CLASP2 GALNT6 NDUFA5 ZNF184 USP20 RPL23A ACVR2B PNRC2 SLC25A38 RPIA SNRNP40 EIF3L IMPDH2 ZNF614 PDCD2 MMACHC
	Intracellular organelle	1.3820E-12	7.1811E-10	RPL3 SPI1 TFRC EHMT2 THYN1 HNRNPR IKZF5 UXT SNRPD1 MYC CHEK2 MYB ZNF845 RPS10 GLUL EIF2A RPS12 SMARCC1 ENDOG ATP6V1G2 ESCO1 ZNF160 OXSM RPL22 METTL3 SYTL1 LARS2 ISL2

				EWSR1 PDE12 CLNS1A BTK PADI4 RPL29 SF1 ZNF397 GCDH ABCB7 EPB41 MRPL16 MTMR8 AAAS IQGAP2 ZBTB3 MRPL11 MBTD1 DHX30 AFMID PCBP2 HESX1 ST8SIA4 SCAP ZNF700 CEP192 PATZ1 SIAH2 SRPK1 MED13L RPS25 POLA1 INTS5 INTS4 PSMG2 ADCK3 HNRNPA1L2 ZNF136 TOP2B GMEB1 ZNF493 SUV39H1 CASP14 MRPL42 ZNF808 SCML2 ZNF41 DLAT OFD1 NDC1 ACAD8 COX8A MRPS25 STRBP TET1 TMC8 MRPS21 CBFA2T3 GNL3 DDB1 RPUSD4 TAF6L KIF9 BDH1 PDIK1L ZADH2 TLR9 TUT1 PTMA SLC29A2 MYCBP RPL11 ATP5A1 ASIP AIF1 EXOSC7 HIRA RPL14 ZNF589 METTL7A RPL17 LAPTM5 GTF2H4 DEF6 SPCS2 ABCB10 LYRM4 LYRM7 NDUFAB1 PKN1 NCAPD3 CD86 KDM1A ZBTB26 UBP1 TSEN2 RPL10A BZRAP1 MACROD1 KPNA5 ZNF445 HMGN2 HADH MEN1 ZBTB39 RPS9 KDM2B HIST1H2AK LMO2 GTF3A VRK1 RPSA SDHD EMSY MAPKAPK3 POLG2 GEMIN8 SRSF9 FKBP5 ZNF551 KHDRBS1 MAX NDUFB11 ARHGAP19 CORO1A ATXN3 H1FX NDUFV2 MDN1 TRAPPC2 DNAJC19 PDHA1 NEK4 ANKRD23 SELP PAN2 RPS10P5 GNL3L TAF4B STT3B RSL24D1 CDCA7L HP1BP3 ATP2A3 LRMP LSP1 LYL1 ZNRD1 TGIF2 ACTR6 GLRX5 DDX51 LSS PTP4A2 HSH2D SLC25A19 ZNF639 PEX6 PCCB PECAM1 SLC25A6 ATF6B HDAC1 ZBTB48 VPRBP PDHB RETN RNF5 HDAC6 ZBTB40 RBBP4 RBBP8 RBBP7 VHL CLASP2 GALNT6 NDUFA5 ZNF184 RPL23A PNRC2 SLC25A38 SNRNP40 EIF3L ZNF614 PDCD2 MMACHC
	Organelle	1.6726E-12	7.2425E-10	RPL3 SPI1 TFRC EHMT2 THYN1 HNRNPR IKZF5 UXT SNRPD1 MYC CHEK2 MYB ZNF845 RPS10 GLUL EIF2A RPS12 SMARCC1 ENDOG ATP6V1G2 ESCO1 ZNF160 OXSM RPL22 METTL3 SYTL1 LARS2 ISL2 EWSR1 PDE12 CLNS1A BTK PADI4 RPL29 SF1 ZNF397 GCDH ABCB7 EPB41 MRPL16 MTMR8 AAAS IQGAP2 ZBTB3 MRPL11 MBTD1 DHX30 AFMID PCBP2 HESX1 ST8SIA4 SCAP ZNF700 CEP192 PATZ1 SIAH2 SRPK1 MED13L RPS25 POLA1 INTS5 INTS4 PSMG2 ADCK3 HNRNPA1L2 ZNF136 TOP2B GMEB1 ZNF493 SUV39H1 CASP14 MRPL42 ZNF808 SCML2 ZNF41 DLAT OFD1 NDC1 ACAD8 COX8A MRPS25 STRBP TET1 TMC8 MRPS21 CBFA2T3 GNL3 DDB1 RPUSD4 TAF6L KIF9 BDH1 PDIK1L ZADH2 TLR9 TUT1 PTMA SLC29A2 MYCBP RPL11 ATP5A1 ASIP AIF1 EXOSC7 HIRA RPL14 ZNF589 METTL7A RPL17 LAPTM5 GTF2H4 DEF6 SPCS2 ABCB10 LYRM4 LYRM7 NDUFAB1 PKN1 NCAPD3 CD86 KDM1A ZBTB26 UBP1 TSEN2 RPL10A BZRAP1 MACROD1 KPNA5 ZNF445 HMGN2 HADH MEN1 ZBTB39 RPS9 KDM2B HIST1H2AK LMO2 GTF3A VRK1 RPSA SDHD EMSY MAPKAPK3 POLG2 GEMIN8 SRSF9 FKBP5 ZNF551 KHDRBS1 MAX NDUFB11 ARHGAP19 CORO1A ATXN3 H1FX NDUFV2 MDN1 TRAPPC2 DNAJC19 PDHA1 NEK4 ANKRD23 SELP PAN2 RPS10P5 GNL3L TAF4B STT3B RSL24D1 CDCA7L HP1BP3 ATP2A3 LRMP LSP1 LYL1 ZNRD1 TGIF2 ACTR6 GLRX5 DDX51 LSS PTP4A2 HSH2D SLC25A19 ZNF639 PEX6 PCCB PECAM1 SLC25A6 ATF6B HDAC1 ZBTB48 VPRBP PDHB RETN RNF5 HDAC6 ZBTB40 RBBP4 RBBP8 RBBP7 VHL CLASP2 GALNT6 NDUFA5 ZNF184 RPL23A PNRC2 SLC25A38 SNRNP40 EIF3L ZNF614 PDCD2 MMACHC
	Ribosome	1.1682E-11	4.2321E-9	RPS9 RPL3 MRPS25 RPL22 RPL11 MRPL16 MRPS21 RPSA RPL23A RPL10A MRPL11 MRPL42 RPS25 RPS10P5 RPL14 RPL29 RPL17 RSL24D1 RPS10 SF1 RPS12
	Ribosomal subunit	1.3032E-11	4.2321E-9	RPS9 RPL3 MRPS25 RPL22 RPL11 MRPL16 MRPS21 RPSA RPL23A MRPL11 MRPL42 RPS25 RPL14 RPL29 RPL17 RPS10 RPS12
	Structural constituent of ribosome	1.2370E-10	3.5707E-8	RPS9 RPL3 MRPS25 RPL22 RPL11 MRPL16 MRPS21 RPSA RPL23A RPL10A MRPL11 MRPL42 RPS25 RPL14 RPL29 RPL17 RSL24D1 RPS12
	Cellular metabolic process	3.1440E-10	8.1682E-8	RPL3 KDM1A ARAF HNRNPR TSEN2 RPL10A NUDT3 UXT SNRPD1 CHEK2 PIM2 HADH RPS10 GLUL MEN1 EIF2A RPS12 RPS9 ENDOG ESCO1 OXSM RPL22 METTL3 RNASE6 GTF3A VRK1 RPSA SDHD PHKA2 EMSY LARS2 MAPKAPK3 POLG2 CLNS1A MSH5 BTK PADI4 TTLL12 RPL29 GEMIN8 SF1 SRSF9 FKBP5 BLK KHDRBS1 MAX PDE1B GIPR NDUFB11 MRPL16 MRPL11 ATXN3 NEU3 AFMID PCBP2 ST8SIA4 NDUFV2 TRAPPC2 DNAJC19 PDHA1 NEK4 SIAH2 ANKRD23 PAICS SRPK1 PAN2 RPS25 POLA1 INTS5 INTS4 TAF4B STT3B HNRNPA1L2 RSL24D1 TOP2B SUV39H1 AMD1 NGLY1 ATP2A3 MRPL42 ZNF808 ZNRD1 DLAT ACAD8 COX8A ADSL MRPS25 MRPS21 DDX51 DDB1 RPUSD4 TAF6L PTP4A2 IFNG PDIK1L TLR9 PTMA SLC29A2 ATF6B HDAC1 RPL11 ATP5A1 ASIP PDHB RNF5 HDAC6 CAMKK2 EXOSC7 RBBP4 RPL14 RBBP8 RBBP7 VHL DCAF11 RPL17 EIF4B GALNT6 NDUFA5 USP20 RPL23A PUS7L GTF2H4 ACVR2B ADAT2 PNRC2 SLC25A38 RPIA SNRNP40 SPCS2 EIF3L IMPDH2 NDUFAB1 PKN1
	Nucleus	5.2749E-10	1.2458E-7	RPL3 SPI1 KDM1A EHMT2 ZBTB26 THYN1 UBP1 HNRNPR TSEN2 IKZF5 UXT SNRPD1 MYC CHEK2 MYB ZNF845 KPNA5 ZNF445 HMGN2 MEN1 EIF2A ZBTB39 RPS9 SMARCC1 ESCO1 KDM2B ZNF160 HIST1H2AK

				METTL3 LMO2 GTF3A VRK1 RPSA EMSY ISL2 MAPKAPK3 EWSR1 CLNS1A BTK PADI4 GEMIN8 SF1 SRSF9 FKBP5 ZNF397 ZNF551 KHDRBS1 MAX EPB41 MTMR8 ARHGAP19 AAAS ZBTB3 MBTD1 ATXN3 AFMID PCBP2 HESX1 ZNF700 H1FX PATZ1 MDN1 NEK4 SIAH2 ANKRD23 SRPK1 MED13L PAN2 RPS25 POLA1 GNL3L INTS5 INTS4 TAF4B PSMG2 HNRNPA1L2 ZNF136 RSL24D1 TOP2B GMEB1 ZNF493 CDCA7L SUV39H1 HP1BP3 ATP2A3 CASP14 ZNF808 LYL1 SCML2 ZNRD1 ZNF41 OFD1 NDC1 TGIF2 STRBP TET1 DDX51 CBFA2T3 GNL3 DDB1 TAF6L HSH2D ZNF639 PDIK1L TUT1 PTMA SLC29A2 MYCBP ATF6B HDAC1 RPL11 ZBTB48 VPRBP RETN AIF1 HDAC6 ZBTB40 EXOSC7 HIRA RBBP4 RBBP8 ZNF589 RBBP7 VHL RPL17 ZNF184 GTF2H4 DEF6 PNRC2 SNRNP40 LYRM4 EIF3L ZNF614 PDCD2 PKN1 NCAPD3
	Translation	6.2526E-10	1.3013E-7	RPS9 RPL3 MRPS25 RPL22 RPL11 MRPL16 MRPS21 RPSA RPL23A RPL10A MRPL11 LARS2 MRPL42 RPS25 POLG2 EIF3L RPL14 RPL29 RPL17 RSL24D1 RPS10 EIF4B RPS12
	Cellular macromolecule metabolic process	6.5114E-10	1.3013E-7	RPL3 KDM1A ARAF HNRNPR TSEN2 RPL10A UXT SNRPD1 CHEK2 PIM2 RPS10 MEN1 EIF2A RPS12 RPS9 ENDOG ESCO1 RPL22 METTL3 RNASE6 GTF3A VRK1 RPSA PHKA2 EMSY LARS2 MAPKAPK3 POLG2 CLNS1A MSH5 BTK PADI4 TTLL12 RPL29 GEMIN8 SF1 SRSF9 FKBP5 BLK KHDRBS1 MAX MRPL16 MRPL11 ATXN3 PCBP2 ST8SIA4 TRAPPC2 DNAJC19 NEK4 SIAH2 SRPK1 PAN2 RPS25 POLA1 INTS5 INTS4 TAF4B STT3B HNRNPA1L2 RSL24D1 TOP2B SUV39H1 NGLY1 MRPL42 ZNF808 MRPS25 MRPS21 DDX51 DDB1 RPUSD4 TAF6L PTP4A2 IFNG PDIK1L TLR9 PTMA ATF6B HDAC1 RPL11 RNF5 HDAC6 CAMKK2 EXOSC7 RBBP4 RPL14 RBBP8 RBBP7 VHL DCAF11 RPL17 EIF4B GALNT6 USP20 RPL23A PUS7L GTF2H4 ACVR2B ADAT2 PNRC2 SNRNP40 SPCS2 EIF3L PKN1
	Gene expression	9.1628E-10	1.7003E-7	RPL3 SUV39H1 HNRNPR TSEN2 RPL10A MRPL42 ZNF808 SNRPD1 RPS10 RPS12 RPS9 MRPS25 RPL22 GTF3A MRPS21 RPSA DDX51 LARS2 TAF6L POLG2 CLNS1A RPL29 GEMIN8 PTMA SF1 SRSF9 KHDRBS1 ATF6B MAX RPL11 MRPL16 MRPL11 EXOSC7 PCBP2 RPL14 RPL17 EIF4B TRAPPC2 RPL23A PUS7L GTF2H4 ADAT2 SRPK1 RPS25 SNRNP40 SPCS2 EIF3L INTS5 INTS4 TAF4B HNRNPA1L2 RSL24D1
	Metabolic process	2.5585E-9	4.4313E-7	RPL3 TFRC KDM1A ARAF HNRNPR TSEN2 RPL10A NUDT3 UXT SNRPD1 CHEK2 PIM2 HADH RPS10 GLUL MEN1 EIF2A RPS12 RPS9 ENDOG ESCO1 KDM2B OXSM RPL22 METTL3 RNASE6 GTF3A VRK1 RPSA SDHD PHKA2 EMSY LARS2 MAPKAPK3 POLG2 CLNS1A MSH5 BTK CARD17 PADI4 TTLL12 RPL29 GEMIN8 SF1 SRSF9 FKBP5 BLK KHDRBS1 GCDH MAX PDE1B GIPR NDUFB11 MRPL16 PLD4 MRPL11 DPP3 ATXN3 NEU3 AFMID PCBP2 ST8SIA4 SCAP NDUFV2 TRAPPC2 DNAJC19 PDHA1 NEK4 SIAH2 ANKRD23 PAICS SRPK1 PAN2 RPS25 POLA1 INTS5 INTS4 TAF4B STT3B HNRNPA1L2 RSL24D1 TOP2B SUV39H1 AMD1 NGLY1 ATP2A3 CASP14 MRPL42 ZNF808 ZNRD1 DLAT PGPEP1 ACAD8 COX8A ADSL MRPS25 TET1 MRPS21 DDX51 LSS DDB1 RPUSD4 TAF6L PTP4A2 BDH1 IFNG PDIK1L ZADH2 TLR9 LCT PTMA SLC29A2 ATF6B HDAC1 RPL11 ATP5A1 ASIP PDHB RNF5 HDAC6 CAMKK2 EXOSC7 RBBP4 RPL14 RBBP8 METTL7A RBBP7 VHL DCAF11 RPL17 EIF4B GALNT6 NDUFA5 USP20 RPL23A PUS7L GTF2H4 ACVR2B ADAT2 PNRC2 SLC25A38 RPIA SNRNP40 SPCS2 EIF3L IMPDH2 NDUFAB1 PKN1
	Intracellular organelle lumen	4.1914E-9	6.8057E-7	TOP2B RPL3 HNRNPR TSEN2 MRPL42 ZNRD1 SNRPD1 MYC CHEK2 MYB DLAT HADH MEN1 RPS12 RPS9 SMARCC1 KDM2B MRPS25 METTL3 MRPS21 VRK1 DDX51 CBFA2T3 GNL3 DDB1 LARS2 TAF6L BDH1 POLG2 PCCB TUT1 GEMIN8 SLC29A2 GCDH MAX HDAC1 RPL11 ATP5A1 MRPL16 PDHB HDAC6 MRPL11 EXOSC7 ATXN3 DHX30 RBBP4 RBBP7 RPL17 PDHA1 GTF2H4 MED13L RPS25 POLA1 GNL3L EIF3L NDUFAB1 INTS5 INTS4 TAF4B RSL24D1
	Ribonucleoprotein complex	5.7443E-9	8.4169E-7	RPL3 RPL11 MRPL16 HNRNPR RPL10A MRPL11 MRPL42 SNRPD1 PCBP2 RPL14 RPL17 RPS10 EIF2A RPS12 RPS9 MRPS25 RPL22 MRPS21 RPSA RPL23A PNRC2 RPS25 RPS10P5 SNRNP40 RPL29 GEMIN8 HNRNPA1L2 RSL24D1 SF1
	Nucleic acid binding	5.8316E-9	8.4169E-7	TOP2B GMEB1 RPL3 SPI1 ZNF493 KDM1A ZBTB26 HP1BP3 UBP1 HNRNPR TSEN2 RPL10A IKZF5 ZNF808 LYL1 SCML2 ZNRD1 SNRPD1 MYC MYB ZNF41 ZNF845 ZNF445 HMGN2 ZNF720 MEN1 EIF2A ZC3H10 ZBTB39 RPS9 SMARCC1 ENDOG TGIF2 ESCO1 KDM2B ZNF160 STRBP HIST1H2AK RPL22 METTL3 LMO2 RNASE6 GTF3A TET1 DDX51 CBFA2T3 DDB1 RPUSD4 TAF6L ISL2 POLG2 EWSR1 ZNF639 MSH5 TLR9 TUT1 RPL29 SF1 SRSF9 ZNF397 ZNF551 KHDRBS1 EEF1A1P5 ATF6B MAX HDAC1 RPL11 ZBTB48 MRPL16 ZBTB3 ZBTB40 EXOSC7 DHX30 HIRA PCBP2 HESX1 RPL14 ZNF700 H1FX ZNF589 PATZ1 EIF4B

				ZNF184 RPL23A PUS7L GTF2H4 PAN2 RPS25 POLA1 ZNF614 TAF4B PDCD2 ZNF852 HNRNPA1L2 ZNF136
	Translational elongation	6.8301E-9	9.3392E-7	RPS9 RPL3 RPL22 RPL11 RPSA RPL23A RPL10A RPS25 RPL14 RPL29 RPL17 RPS10 RPS12
	Organelle lumen	9.2074E-9	1.1960E-6	TOP2B RPL3 HNRNPR TSEN2 MRPL42 ZNRD1 SNRPD1 MYC CHEK2 MYB DLAT HADH MEN1 RPS12 RPS9 SMARCC1 KDM2B MRPS25 METTL3 MRPS21 VRK1 DDX51 CBFA2T3 GNL3 DDB1 LARS2 TAF6L BDH1 POLG2 PCCB TUT1 GEMIN8 SLC29A2 GCDH MAX HDAC1 RPL11 ATP5A1 MRPL16 PDHB HDAC6 MRPL11 EXOSC7 ATXN3 DHX30 RBBP4 RBBP7 RPL17 PDHA1 GTF2H4 MED13L RPS25 POLA1 GNL3L EIF3L NDUFAB1 INTS5 INTS4 TAF4B RSL24D1
	Primary metabolic process	1.7713E-8	2.1914E-6	RPL3 TFRC KDM1A ARAF HNRNPR TSEN2 RPL10A NUDT3 UXT SNRPD1 CHEK2 PIM2 HADH RPS10 GLUL MEN1 EIF2A RPS12 RPS9 ENDOG ESCO1 OXSM RPL22 METTL3 RNASE6 GTF3A VRK1 RPSA PHKA2 EMSY LARS2 MAPKAPK3 POLG2 CLNS1A MSH5 BTK CARD17 PADI4 TTLL12 RPL29 GEMIN8 SF1 SRSF9 FKBP5 BLK KHDRBS1 MAX PDE1B MRPL16 PLD4 MRPL11 DPP3 ATXN3 NEU3 AFMID PCBP2 ST8SIA4 SCAP TRAPPC2 DNAJC19 PDHA1 NEK4 SIAH2 ANKRD23 PAICS SRPK1 PAN2 RPS25 POLA1 INTS5 INTS4 TAF4B STT3B HNRNPA1L2 RSL24D1 TOP2B SUV39H1 AMD1 ATP2A3 CASP14 MRPL42 ZNF808 ZNRD1 DLAT PGPEP1 ACAD8 ADSL MRPS25 MRPS21 DDX51 LSS DDB1 RPUSD4 TAF6L PTP4A2 IFNG PDIK1L TLR9 LCT PTMA SLC29A2 ATF6B HDAC1 RPL11 ATP5A1 PDHB RNF5 HDAC6 CAMKK2 EXOSC7 RBBP4 RPL14 RBBP8 RBBP7 VHL DCAF11 RPL17 EIF4B GALNT6 USP20 RPL23A PUS7L GTF2H4 ACVR2B ADAT2 PNRC2 RPIA SNRNP40 SPCS2 EIF3L IMPDH2 NDUFAB1 PKN1
	Membrane-enclosed lumen	2.0092E-8	2.3727E-6	TOP2B RPL3 HNRNPR TSEN2 MRPL42 ZNRD1 SNRPD1 MYC CHEK2 MYB DLAT HADH MEN1 RPS12 RPS9 SMARCC1 KDM2B MRPS25 METTL3 MRPS21 VRK1 DDX51 CBFA2T3 GNL3 DDB1 LARS2 TAF6L BDH1 POLG2 PCCB TUT1 GEMIN8 SLC29A2 GCDH MAX HDAC1 RPL11 ATP5A1 MRPL16 PDHB HDAC6 MRPL11 EXOSC7 ATXN3 DHX30 RBBP4 RBBP7 RPL17 PDHA1 GTF2H4 MED13L RPS25 POLA1 GNL3L EIF3L NDUFAB1 INTS5 INTS4 TAF4B RSL24D1
	Mitochondrion	2.7877E-8	3.1489E-6	TFRC RPL10A MRPL42 BZRAP1 MACROD1 DLAT HADH GLUL RPS12 ACAD8 COX8A ENDOG MRPS25 OXSM GLRX5 MRPS21 SDHD RPUSD4 LARS2 BDH1 POLG2 SLC25A19 PDE12 ZADH2 PCCB SLC25A6 GCDH MYCBP ABCB7 NDUFB11 ATP5A1 MRPL16 PDHB RNF5 MRPL11 DHX30 VHL NDUFV2 DNAJC19 PDHA1 NDUFA5 SLC25A38 PAN2 ABCB10 LYRM4 LYRM7 NDUFAB1 ADCK3 MMACHC
	Macromolecule metabolic process	3.2573E-8	3.3614E-6	RPL3 TFRC KDM1A ARAF HNRNPR TSEN2 RPL10A UXT SNRPD1 CHEK2 PIM2 RPS10 MEN1 EIF2A RPS12 RPS9 ENDOG ESCO1 RPL22 METTL3 RNASE6 GTF3A VRK1 RPSA PHKA2 EMSY LARS2 MAPKAPK3 POLG2 CLNS1A MSH5 BTK CARD17 PADI4 TTLL12 RPL29 GEMIN8 SF1 SRSF9 FKBP5 BLK KHDRBS1 MAX MRPL16 MRPL11 DPP3 ATXN3 PCBP2 ST8SIA4 TRAPPC2 DNAJC19 NEK4 SIAH2 SRPK1 PAN2 RPS25 POLA1 INTS5 INTS4 TAF4B STT3B HNRNPA1L2 RSL24D1 TOP2B SUV39H1 NGLY1 CASP14 MRPL42 ZNF808 PGPEP1 MRPS25 MRPS21 DDX51 DDB1 RPUSD4 TAF6L PTP4A2 IFNG PDIK1L TLR9 PTMA ATF6B HDAC1 RPL11 RNF5 HDAC6 CAMKK2 EXOSC7 RBBP4 RPL14 RBBP8 RBBP7 VHL DCAF11 RPL17 EIF4B GALNT6 USP20 RPL23A PUS7L GTF2H4 ACVR2B ADAT2 PNRC2 SNRNP40 SPCS2 EIF3L PKN1
	Mitochondrial matrix	3.3640E-8	3.3614E-6	GCDH PDHA1 MRPS25 ATP5A1 MRPL16 MRPS21 PDHB MRPL11 LARS2 MRPL42 DHX30 BDH1 POLG2 PCCB NDUFAB1 DLAT HADH RPS12
	Mitochondrial lumen	3.3640E-8	3.3614E-6	GCDH PDHA1 MRPS25 ATP5A1 MRPL16 MRPS21 PDHB MRPL11 LARS2 MRPL42 DHX30 BDH1 POLG2 PCCB NDUFAB1 DLAT HADH RPS12
	Cytosolic ribosome	4.3148E-8	4.1518E-6	RPS25 RPS9 RPL3 RPL22 RPL11 RPL14 RPSA RPL23A RPL29 RPS10 RPS12
	Nucleobase, nucleoside, nucleotide and nucleic acid metabolic process	8.7971E-8	8.1625E-6	TOP2B SUV39H1 ATP2A3 HNRNPR TSEN2 RPL10A NUDT3 ZNF808 ZNRD1 SNRPD1 MEN1 ADSL ENDOG ESCO1 METTL3 RNASE6 GTF3A DDX51 EMSY DDB1 RPUSD4 LARS2 TAF6L POLG2 CLNS1A MSH5 GEMIN8 PTMA SF1 SLC29A2 SRSF9 KHDRBS1 ATF6B MAX PDE1B RPL11 ATP5A1 EXOSC7 ATXN3 RBBP4 PCBP2 RPL14 RBBP8 RBBP7 TRAPPC2 PUS7L GTF2H4 PAICS ADAT2 PNRC2 SRPK1 PAN2 RPIA POLA1 SNRNP40 IMPDH2 INTS5 INTS4 TAF4B HNRNPA1L2
	Cellular nitrogen compound metabolic process	1.4145E-7	1.2672E-5	TOP2B SUV39H1 AMD1 ATP2A3 HNRNPR TSEN2 RPL10A NUDT3 ZNF808 ZNRD1 SNRPD1 GLUL MEN1 ACAD8 ADSL ENDOG ESCO1 METTL3 RNASE6 GTF3A DDX51 EMSY DDB1 RPUSD4 LARS2 TAF6L POLG2 CLNS1A MSH5 PADI4 GEMIN8 PTMA SF1 SLC29A2 SRSF9 KHDRBS1 ATF6B MAX PDE1B RPL11 ATP5A1 EXOSC7 ATXN3 RBBP4 AFMID PCBP2 RPL14 RBBP8 RBBP7 TRAPPC2 PUS7L GTF2H4 PAICS ADAT2 PNRC2 SRPK1 SLC25A38 PAN2 RPIA POLA1 SNRNP40 IMPDH2

				INTS5 INTS4 TAF4B HNRNPA1L2
	Mitochondrial part	4.1449E-7	3.5895E-5	GCDH ABCB7 NDUFB11 ATP5A1 MRPL16 PDHB MRPL11 MRPL42 DHX30 DLAT HADH NDUFV2 RPS12 COX8A DNAJC19 PDHA1 NDUFA5 MRPS25 MRPS21 SDHD SLC25A38 LARS2 ABCB10 BDH1 POLG2 SLC25A19 PCCB NDUFAB1 SLC25A6
	RNA binding	6.3067E-7	5.2854E-5	KHDRBS1 RPL3 SPI1 RPL11 MRPL16 HNRNPR RPL10A EXOSC7 DHX30 SNRPD1 PCBP2 RPL14 EIF4B EIF2A RPS9 KDM2B STRBP RPL22 METTL3 GTF3A RPL23A DDX51 PUS7L RPUSD4 RPS25 EWSR1 TLR9 TUT1 RPL29 HNRNPA1L2 SF1 SRSF9
	Nucleic acid metabolic process	7.3799E-7	5.9915E-5	TOP2B SUV39H1 HNRNPR TSEN2 RPL10A ZNF808 SNRPD1 MEN1 ENDOG ESCO1 METTL3 RNASE6 GTF3A DDX51 EMSY DDB1 RPUSD4 LARS2 TAF6L POLG2 CLNS1A MSH5 GEMIN8 PTMA SF1 SRSF9 KHDRBS1 ATF6B MAX RPL11 EXOSC7 ATXN3 RBBP4 PCBP2 RPL14 RBBP8 RBBP7 TRAPPC2 PUS7L GTF2H4 ADAT2 PNRC2 SRPK1 PAN2 POLA1 SNRNP40 INTS5 INTS4 TAF4B HNRNPA1L2
	Large ribosomal subunit	9.3871E-7	7.3902E-5	RPL3 RPL22 RPL11 MRPL16 RPL14 RPL23A RPL29 RPL17 MRPL11
	Nitrogen compound metabolic process	1.0534E-6	8.0488E-5	TOP2B SUV39H1 AMD1 ATP2A3 HNRNPR TSEN2 RPL10A NUDT3 ZNF808 ZNRD1 SNRPD1 GLUL MEN1 ACAD8 ADSL ENDOG ESCO1 METTL3 RNASE6 GTF3A DDX51 EMSY DDB1 RPUSD4 LARS2 TAF6L POLG2 CLNS1A MSH5 PADI4 GEMIN8 PTMA SF1 SLC29A2 SRSF9 KHDRBS1 ATF6B MAX PDE1B RPL11 ATP5A1 EXOSC7 ATXN3 RBBP4 AFMID PCBP2 RPL14 RBBP8 RBBP7 TRAPPC2 PUS7L GTF2H4 PAICS ADAT2 PNRC2 SRPK1 SLC25A38 PAN2 RPIA POLA1 SNRNP40 IMPDH2 INTS5 INTS4 TAF4B HNRNPA1L2
	Intracellular organelle part	2.8866E-6	2.1427E-4	RPL3 HNRNPR TSEN2 UXT SNRPD1 MYC CHEK2 MYB KPNA5 HMGN2 HADH RPS10 MEN1 RPS12 RPS9 SMARCC1 ATP6V1G2 KDM2B HIST1H2AK RPL22 METTL3 VRK1 RPSA SDHD LARS2 POLG2 RPL29 GEMIN8 SF1 GCDH ABCB7 MAX EPB41 NDUFB11 MRPL16 MTMR8 AAAS CORO1A MRPL11 ATXN3 DHX30 ST8SIA4 SCAP CEP192 H1FX NDUFV2 DNAJC19 PDHA1 MED13L SELP RPS25 POLA1 GNL3L INTS5 INTS4 TAF4B STT3B HNRNPA1L2 RSL24D1 TOP2B SUV39H1 HP1BP3 ATP2A3 LRMP MRPL42 ZNRD1 DLAT OFD1 NDC1 COX8A MRPS25 TET1 TMC8 MRPS21 DDX51 CBFA2T3 LSS GNL3 DDB1 TAF6L KIF9 BDH1 SLC25A19 PEX6 PCCB PECAM1 TLR9 TUT1 SLC29A2 SLC25A6 ATF6B HDAC1 RPL11 ATP5A1 ASIP PDHB HDAC6 EXOSC7 HIRA RBBP4 RPL14 RBBP7 RPL17 CLASP2 GALNT6 NDUFA5 LAPTM5 RPL23A GTF2H4 SLC25A38 SNRNP40 SPCS2 ABCB10 EIF3L NDUFAB1 NCAPD3
	Organelle part	3.3223E-6	2.3976E-4	RPL3 HNRNPR TSEN2 UXT SNRPD1 MYC CHEK2 MYB KPNA5 HMGN2 HADH RPS10 MEN1 RPS12 RPS9 SMARCC1 ATP6V1G2 KDM2B HIST1H2AK RPL22 METTL3 VRK1 RPSA SDHD LARS2 POLG2 RPL29 GEMIN8 SF1 GCDH ABCB7 MAX EPB41 NDUFB11 MRPL16 MTMR8 AAAS CORO1A MRPL11 ATXN3 DHX30 ST8SIA4 SCAP CEP192 H1FX NDUFV2 DNAJC19 PDHA1 ANKRD23 MED13L SELP RPS25 POLA1 GNL3L INTS5 INTS4 TAF4B STT3B HNRNPA1L2 RSL24D1 TOP2B SUV39H1 HP1BP3 ATP2A3 LRMP MRPL42 ZNRD1 DLAT OFD1 NDC1 COX8A MRPS25 TET1 TMC8 MRPS21 DDX51 CBFA2T3 LSS GNL3 DDB1 TAF6L KIF9 BDH1 SLC25A19 PEX6 PCCB PECAM1 TLR9 TUT1 SLC29A2 SLC25A6 ATF6B HDAC1 RPL11 ATP5A1 ASIP PDHB HDAC6 EXOSC7 HIRA RBBP4 RPL14 RBBP7 RPL17 CLASP2 GALNT6 NDUFA5 LAPTM5 RPL23A GTF2H4 SLC25A38 SNRNP40 SPCS2 ABCB10 EIF3L NDUFAB1 NCAPD3
	Cellular macromolecule biosynthetic process	3.5690E-6	2.5060E-4	RPL3 ATF6B MAX RPL11 MRPL16 RPL10A MRPL11 MRPL42 ZNF808 RBBP4 RPL14 ST8SIA4 RBBP7 RPL17 RPS10 EIF4B RPS12 TRAPPC2 GALNT6 RPS9 MRPS25 RPL22 GTF3A MRPS21 RPSA RPL23A GTF2H4 LARS2 TAF6L RPS25 POLA1 POLG2 EIF3L TAF4B STT3B RPL29 RSL24D1 PTMA
	Cytosolic part	4.2853E-6	2.9298E-4	UXT RPS25 RPS9 RPL3 RPL22 RPL11 RPL14 RPSA RPL23A RPL29 RPS10 RPS12
	Ribonucleoprotein complex biogenesis	4.8179E-6	3.2095E-4	SUV39H1 RPL11 DDX51 EXOSC7 RPS25 GNL3L SNRPD1 CLNS1A RPL14 GEMIN8 RSL24D1 SF1 SRSF9 EIF2A
	Small ribosomal subunit	5.0074E-6	3.2257E-4	MRPL42 RPS25 RPS9 MRPS25 MRPS21 RPSA RPS10 RPS12
	Nuclear part	5.0906E-6	3.2257E-4	TOP2B RPL3 SUV39H1 ATP2A3 HNRNPR TSEN2 ZNRD1 SNRPD1 MYC CHEK2 MYB KPNA5 MEN1 NDC1 RPS9 SMARCC1 KDM2B METTL3 TET1 VRK1 DDX51 CBFA2T3 GNL3 DDB1 TAF6L TUT1 GEMIN8 SF1 SLC29A2 MAX HDAC1 RPL11 MTMR8 AAAS HDAC6 EXOSC7 ATXN3

				HIRA RBBP4 RBBP7 RPL17 GTF2H4 MED13L RPS25 POLA1 SNRNP40 GNL3L EIF3L INTS5 INTS4 TAF4B NCAPD3 HNRNPA1L2 RSL24D1
	Macromolecule biosynthetic process	5.6990E-6	3.5252E-4	RPL3 ATF6B MAX RPL11 MRPL16 RPL10A MRPL11 MRPL42 ZNF808 RBBP4 RPL14 ST8SIA4 RBBP7 RPL17 RPS10 EIF4B RPS12 TRAPPC2 GALNT6 RPS9 MRPS25 RPL22 GTF3A MRPS21 RPSA RPL23A GTF2H4 LARS2 TAF6L RPS25 POLA1 POLG2 EIF3L TAF4B STT3B RPL29 RSL24D1 PTMA
	Macromolecular complex	7.1416E-6	4.3148E-4	TOP2B RPL3 SUV39H1 HP1BP3 HNRNPR TSEN2 RPL10A ITGAL UXT MRPL42 SNRPD1 KPNA5 DLAT HADH RPS10 MEN1 EIF2A RPS12 NDC1 RPS9 SMARCC1 ATP6V1G2 MRPS25 HIST1H2AK RPL22 LMO2 MRPS21 RPSA SDHD PHKA2 DDB1 TAF6L KIF9 CD48 RPL29 GEMIN8 SF1 SLC25A6 MAX HDAC1 EPB41 NDUFB11 RPL11 ATP5A1 MRPL16 VPS26B PDHB AAAS CORO1A HDAC6 MRPL11 EXOSC7 RBBP4 PCBP2 RPL14 SCAP H1FX RBBP7 VHL DCAF11 NDUFV2 RPL17 EIF4B CLASP2 NDUFA5 RPL23A GTF2H4 PNRC2 MED13L RPS25 POLA1 RPS10P5 SNRNP40 SPCS2 EIF3L NDUFAB1 INTS5 INTS4 TAF4B NCAPD3 STT3B HNRNPA1L2 RSL24D1
	Regulation of transcription	8.2169E-6	4.8517E-4	CD86 GMEB1 SPI1 ZNF493 KDM1A CDCA7L SUV39H1 ZBTB26 UBP1 IKZF5 ZNF808 SCML2 BCL7A MYC MYB ZNF41 ZNF845 ZNF445 HMGN2 ZNF720 MEN1 ACAD8 ZBTB39 SMARCC1 TGIF2 KDM2B ZNF160 GTF3A CBFA2T3 EMSY TAF6L ISL2 IFNG EWSR1 ZNF639 TLR9 PADI4 SF1 ZNF397 ZNF551 KHDRBS1 MYCBP ATF6B MAX HDAC1 ZBTB48 HDAC6 ZBTB3 ZBTB40 CAMKK2 MBTD1 ATXN3 HIRA RBBP4 HESX1 RBBP8 SCAP ZNF700 ZNF589 RBBP7 VHL PATZ1 TRAPPC2 ZNF184 GTF2H4 ACVR2B PNRC2 MED13L ZNF614 TAF4B PKN1 ZNF136
	Cellular protein metabolic process	9.4588E-6	5.4609E-4	RPL3 KDM1A ARAF RPL10A UXT MRPL42 CHEK2 PIM2 RPS10 EIF2A RPS12 RPS9 MRPS25 RPL22 MRPS21 VRK1 RPSA PHKA2 DDB1 LARS2 TAF6L PTP4A2 MAPKAPK3 POLG2 IFNG PDIK1L BTK TLR9 PADI4 TTLL12 RPL29 FKBP5 BLK HDAC1 RPL11 MRPL16 RNF5 HDAC6 MRPL11 CAMKK2 PCBP2 RPL14 ST8SIA4 VHL DCAF11 RPL17 EIF4B DNAJC19 GALNT6 NEK4 USP20 SIAH2 RPL23A ACVR2B SRPK1 PAN2 RPS25 SPCS2 EIF3L PKN1 STT3B RSL24D1
	Cellular biosynthetic process	1.9401E-5	1.0957E-3	RPL3 AMD1 ATP2A3 RPL10A MRPL42 ZNF808 DLAT RPS10 GLUL RPS12 RPS9 ADSL MRPS25 OXSM RPL22 GTF3A MRPS21 RPSA LARS2 TAF6L POLG2 PADI4 RPL29 PTMA ATF6B MAX RPL11 ATP5A1 ASIP MRPL16 MRPL11 RBBP4 RPL14 ST8SIA4 RBBP7 RPL17 EIF4B TRAPPC2 GALNT6 RPL23A GTF2H4 PAICS SLC25A38 RPS25 POLA1 EIF3L IMPDH2 NDUFAB1 TAF4B STT3B RSL24D1
	Chromatin organization	2.0525E-5	1.1345E-3	SMARCC1 KDM1A KDM2B HDAC1 EHMT2 SUV39H1 HIST1H2AK HP1BP3 EMSY HDAC6 MBTD1 TAF6L HIRA RBBP4 H1FX PADI4 PKN1 RBBP7 HMGN2 MEN1
	Cytosolic large ribosomal subunit	2.5929E-5	1.4034E-3	RPL3 RPL22 RPL11 RPL14 RPL23A RPL29
	Regulation of cellular biosynthetic process	2.8743E-5	1.5177E-3	CD86 GMEB1 SPI1 ZNF493 KDM1A CDCA7L SUV39H1 ZBTB26 UBP1 IKZF5 ZNF808 SCML2 BCL7A MYC MYB ZNF41 ZNF845 DLAT ZNF445 HMGN2 ZNF720 MEN1 EIF2A ACAD8 ZBTB39 SMARCC1 TGIF2 KDM2B ZNF160 GTF3A CBFA2T3 EMSY TAF6L ISL2 IFNG EWSR1 ZNF639 TLR9 PADI4 SF1 ZNF397 ZNF551 KHDRBS1 MYCBP ATF6B MAX HDAC1 GIPR ZBTB48 PDHB HDAC6 ZBTB3 ZBTB40 CAMKK2 MBTD1 ATXN3 HIRA RBBP4 HESX1 RBBP8 SCAP ZNF700 ZNF589 RBBP7 VHL PATZ1 EIF4B TRAPPC2 PDHA1 ZNF184 GTF2H4 ACVR2B PNRC2 MED13L ZNF614 TAF4B PKN1 ZNF136
	RNA metabolic process	3.0672E-5	1.5177E-3	KHDRBS1 MAX SUV39H1 RPL11 HNRNPR TSEN2 RPL10A EXOSC7 SNRPD1 PCBP2 RPL14 METTL3 RNASE6 GTF3A DDX51 PUS7L GTF2H4 ADAT2 PNRC2 SRPK1 RPUSD4 PAN2 LARS2 TAF6L SNRNP40 POLG2 CLNS1A INTS5 INTS4 TAF4B GEMIN8 HNRNPA1L2 SF1 SRSF9
	Non-membrane-bounded organelle	3.1226E-5	1.5177E-3	TOP2B RPL3 SUV39H1 HP1BP3 LSP1 TSEN2 RPL10A UXT MRPL42 ZNRD1 MYC HMGN2 HADH RPS10 MEN1 RPS12 OFD1 RPS9 SMARCC1 KDM2B ACTR6 MRPS25 STRBP HIST1H2AK RPL22 MRPS21 VRK1 RPSA DDX51 CBFA2T3 GNL3 KIF9 POLG2 CLNS1A TUT1 RPL29 SF1 SLC29A2 HDAC1 EPB41 RPL11 ASIP MRPL16 IQGAP2 CORO1A AIF1 HDAC6 MRPL11 EXOSC7 DHX30 HIRA RBBP4 RPL14 CEP192 H1FX RPL17 CLASP2 ANKRD23 RPL23A RPS25 POLA1 RPS10P5 GNL3L EIF3L NCAPD3 RSL24D1
	Intracellular non-membrane-bounded organelle	3.1226E-5	1.5177E-3	TOP2B RPL3 SUV39H1 HP1BP3 LSP1 TSEN2 RPL10A UXT MRPL42 ZNRD1 MYC HMGN2 HADH RPS10 MEN1 RPS12 OFD1 RPS9 SMARCC1 KDM2B ACTR6 MRPS25 STRBP HIST1H2AK RPL22 MRPS21 VRK1 RPSA DDX51 CBFA2T3 GNL3 KIF9 POLG2 CLNS1A TUT1 RPL29 SF1 SLC29A2 HDAC1 EPB41 RPL11 ASIP MRPL16 IQGAP2 CORO1A AIF1 HDAC6 MRPL11 EXOSC7 DHX30 HIRA RBBP4 RPL14 CEP192 H1FX RPL17

				CLASP2 ANKRD23 RPL23A RPS25 POLA1 RPS10P5 GNL3L EIF3L NCAPD3 RSL24D1
	Chromatin remodeling complex	3.1275E-5	1.5177E-3	TAF6L TOP2B SMARCC1 RBBP4 HDAC1 SUV39H1 RBBP7 HDAC6
	Regulation of gene expression	3.1546E-5	1.5177E-3	CD86 GMEB1 SPI1 ZNF493 KDM1A CDCA7L SUV39H1 ZBTB26 UBP1 IKZF5 ZNF808 SCML2 BCL7A MYC MYB ZNF41 ZNF845 ZNF445 HMGN2 ZNF720 MEN1 EIF2A ACAD8 ZBTB39 SMARCC1 TGIF2 KDM2B ZNF160 GTF3A CBFA2T3 EMSY TAF6L ISL2 IFNG EWSR1 ZNF639 PEX6 TLR9 PADI4 SF1 ZNF397 ZNF551 KHDRBS1 MYCBP ATF6B MAX HDAC1 ZBTB48 HDAC6 ZBTB3 ZBTB40 CAMKK2 MBTD1 ATXN3 HIRA RBBP4 HESX1 RBBP8 SCAP ZNF700 ZNF589 RBBP7 VHL PATZ1 EIF4B TRAPPC2 ZNF184 GTF2H4 ACVR2B PNRC2 SRPK1 MED13L ZNF614 TAF4B PKN1 ZNF136
	Nuclear lumen	3.4689E-5	1.6386E-3	TOP2B RPL3 MAX HDAC1 RPL11 HNRNPR TSEN2 HDAC6 EXOSC7 ATXN3 ZNRD1 RBBP4 SNRPD1 MYC CHEK2 MYB RBBP7 RPL17 MEN1 RPS9 SMARCC1 KDM2B METTL3 VRK1 DDX51 GTF2H4 CBFA2T3 GNL3 MED13L DDB1 TAF6L RPS25 POLA1 GNL3L EIF3L INTS5 TUT1 INTS4 TAF4B GEMIN8 RSL24D1 SLC29A2
	Regulation of biosynthetic process	3.8014E-5	1.7636E-3	CD86 GMEB1 SPI1 ZNF493 KDM1A CDCA7L SUV39H1 ZBTB26 UBP1 IKZF5 ZNF808 SCML2 BCL7A MYC MYB ZNF41 ZNF845 DLAT ZNF445 HMGN2 ZNF720 MEN1 EIF2A ACAD8 ZBTB39 SMARCC1 TGIF2 KDM2B ZNF160 GTF3A CBFA2T3 EMSY TAF6L ISL2 IFNG EWSR1 ZNF639 TLR9 PADI4 SF1 ZNF397 ZNF551 KHDRBS1 MYCBP ATF6B MAX HDAC1 GIPR ZBTB48 PDHB HDAC6 ZBTB3 ZBTB40 CAMKK2 MBTD1 ATXN3 HIRA RBBP4 HESX1 RBBP8 SCAP ZNF700 ZNF589 RBBP7 VHL PATZ1 EIF4B TRAPPC2 PDHA1 ZNF184 GTF2H4 ACVR2B PNRC2 MED13L ZNF614 TAF4B PKN1 ZNF136
	Mitochondrial inner membrane	4.2421E-5	1.9335E-3	COX8A DNAJC19 GCDH NDUFA5 ABCB7 NDUFB11 ATP5A1 SDHD SLC25A38 ABCB10 BDH1 SLC25A19 NDUFAB1 HADH NDUFV2 SLC25A6
	Positive regulation of receptor biosynthetic process	4.4597E-5	1.9976E-3	HDAC1 SCAP HDAC6
	Biosynthetic process	5.1368E-5	2.2619E-3	RPL3 AMD1 ATP2A3 RPL10A MRPL42 ZNF808 DLAT RPS10 GLUL RPS12 RPS9 ADSL MRPS25 OXSM RPL22 GTF3A MRPS21 RPSA LSS LARS2 TAF6L POLG2 PADI4 RPL29 PTMA ATF6B MAX RPL11 ATP5A1 ASIP MRPL16 MRPL11 RBBP4 RPL14 ST8SIA4 RBBP7 RPL17 EIF4B TRAPPC2 GALNT6 RPL23A GTF2H4 PAICS SLC25A38 RPS25 POLA1 EIF3L IMPDH2 NDUFAB1 TAF4B STT3B RSL24D1
	Cytoplasmic part	5.2314E-5	2.2652E-3	RPL3 TFRC RPL10A UXT BZRAP1 SNRPD1 MACROD1 HADH RPS10 GLUL MEN1 EIF2A RPS12 RPS9 ENDOG ATP6V1G2 OXSM RPL22 SYTL1 RPSA SDHD PHKA2 LARS2 MAPKAPK3 POLG2 PDE12 CLNS1A BTK RPL29 SF1 GCDH ABCB7 EPB41 PDE1B NDUFB11 MRPL16 VPS26B RASGRP2 CORO1A MRPL11 DHX30 AFMID ST8SIA4 SCAP CEP192 NDUFV2 TRAPPC2 DNAJC19 PDHA1 ANKRD23 SELP PAN2 RPS25 RPS10P5 TAF4B ADCK3 STT3B RSL24D1 TOP2B AMD1 ATP2A3 LRMP MRPL42 DLAT PGPEP1 OFD1 ACAD8 COX8A ADSL MRPS25 GLRX5 TMC8 MRPS21 CBFA2T3 LSS RPUSD4 PTP4A2 BDH1 SLC25A19 ZADH2 PEX6 PCCB PECAM1 TLR9 SLC25A6 MYCBP ATF6B HDAC1 RPL11 ATP5A1 ASIP PDHB RNF5 HDAC6 RPL14 METTL7A VHL RPL17 EIF4B CLASP2 GALNT6 NDUFA5 USP20 LAPTM5 RPL23A PNRC2 SLC25A38 RPIA SPCS2 ABCB10 LYRM4 EIF3L IMPDH2 LYRM7 NDUFAB1 PKN1 MMACHC
	Regulation of macromolecule biosynthetic process	5.6538E-5	2.4080E-3	CD86 GMEB1 SPI1 ZNF493 KDM1A CDCA7L SUV39H1 ZBTB26 UBP1 IKZF5 ZNF808 SCML2 BCL7A MYC MYB ZNF41 ZNF845 ZNF445 HMGN2 ZNF720 MEN1 EIF2A ACAD8 ZBTB39 SMARCC1 TGIF2 KDM2B ZNF160 GTF3A CBFA2T3 EMSY TAF6L ISL2 IFNG EWSR1 ZNF639 TLR9 PADI4 SF1 ZNF397 ZNF551 KHDRBS1 MYCBP ATF6B MAX HDAC1 ZBTB48 HDAC6 ZBTB3 ZBTB40 CAMKK2 MBTD1 ATXN3 HIRA RBBP4 HESX1 RBBP8 SCAP ZNF700 ZNF589 RBBP7 VHL PATZ1 EIF4B TRAPPC2 ZNF184 GTF2H4 ACVR2B PNRC2 MED13L ZNF614 TAF4B PKN1 ZNF136
	Chromatin modification	7.0440E-5	2.9516E-3	SMARCC1 KDM1A KDM2B HDAC1 EHMT2 SUV39H1 EMSY HDAC6 MBTD1 TAF6L HIRA RBBP4 PADI4 PKN1 RBBP7 MEN1
	Rrna binding	7.3986E-5	3.0511E-3	RPS9 KDM2B RPL11 MRPL16 RPL23A
	Nucleolus	8.9875E-5	3.6484E-3	RPS9 RPL3 KDM2B RPL11 VRK1 DDX51 TSEN2 CBFA2T3 GNL3 EXOSC7 RPS25 POLA1 GNL3L ZNRD1 EIF3L MYC TUT1 RPL17 RSL24D1 SLC29A2

	Cellular process	9.2073E-5	3.6801E-3	CD86 RPL3 CLPB TFRC KDM1A EHMT2 ARAF HNRNPR TSEN2 RPL10A NUDT3 UXT SNRPD1 MYC CHEK2 PIM2 KPNA5 HMGN2 HADH RPS10 GLUL MEN1 EIF2A RPS12 RPS9 SMARCC1 ENDOG ESCO1 KDM2B CD300A OXSM HIST1H2AK RPL22 METTL3 SYTL1 RNASE6 GTF3A VRK1 RPSA SDHD PHKA2 EMSY LARS2 ISL2 MAPKAPK3 POLG2 CLNS1A MSH5 BTK PADI4 TTLL12 RPL29 GEMIN8 SF1 SRSF9 FKBP5 BLK KHDRBS1 ABCB7 MAX EPB41 PDE1B GIPR NDUFB11 MRPL16 VPS26B AAAS LY9 CORO1A MRPL11 MBTD1 ATXN3 NEU3 AFMID PCBP2 ST8SIA4 SCAP CEP192 H1FX PATZ1 NDUFV2 TRAPPC2 DNAJC19 PDHA1 NEK4 SIAH2 ANKRD23 LY86 PAICS SRPK1 SELP PAN2 RPS25 POLA1 GNL3L INTS5 INTS4 TAF4B PSMG2 ADCK3 STT3B HNRNPA1L2 RSL24D1 TOP2B SUV39H1 AMD1 NGLY1 HP1BP3 ATP2A3 LRMP LSP1 ITGAL CASP14 MRPL42 ZNF808 ZNRD1 DLAT OFD1 NDC1 ACAD8 COX8A ADSL TGIF2 MRPS25 STRBP GLRX5 SH2D3C MRPS21 DDX51 CBFA2T3 DDB1 RPUSD4 TAF6L PTP4A2 KIF9 HSH2D IFNG SLC25A19 PDIK1L PEX6 PECAM1 TLR9 CD48 PTMA SLC29A2 SLC25A6 ATF6B HDAC1 RPL11 ATP5A1 ASIP PDHB RETN AIF1 RNF5 HDAC6 CAMKK2 EXOSC7 HIRA RBBP4 RPL14 RBBP8 RBBP7 VHL DCAF11 RPL17 EIF4B CLASP2 GALNT6 NDUFA5 USP20 RPL23A PUS7L GTF2H4 ACVR2B ADAT2 PNRC2 SLC25A38 RPIA SNRNP40 SPCS2 ABCB10 EIF3L IMPDH2 NDUFAB1 PDCD2 PKN1 NCAPD3
	Cytoplasm	1.1665E-4	4.5918E-3	RPL3 TFRC HNRNPR RPL10A NUDT3 UXT BZRAP1 FRAT1 SNRPD1 MACROD1 KPNA5 HMGN2 HADH RPS10 GLUL MEN1 EIF2A RPS12 RPS9 ENDOG ATP6V1G2 OXSM RPL22 SYTL1 VRK1 RPSA SDHD PHKA2 RNF123 LARS2 MAPKAPK3 POLG2 EWSR1 PDE12 CLNS1A BTK CARD17 C3ORF20 PADI4 RPL29 GEMIN8 GTSF1 SF1 FKBP5 ZNF397 GCDH ABCB7 MAX EPB41 PDE1B NDUFB11 MRPL16 MTMR8 VPS26B RASGRP2 CORO1A MRPL11 DPP3 ATXN3 DHX30 AFMID PCBP2 ST8SIA4 SCAP CEP192 NDUFV2 TRAPPC2 DNAJC19 PDHA1 SIAH2 ANKRD23 SRPK1 SELP PAN2 RPS25 POLA1 RPS10P5 TAF4B ADCK3 STT3B HNRNPA1L2 RSL24D1 TOP2B GMEB1 CDCA7L AMD1 NGLY1 ATP2A3 LRMP CASP14 MRPL42 DLAT PGPEP1 OFD1 ACAD8 COX8A ADSL ACTR6 MRPS25 STRBP GLRX5 SH2D3C TMC8 MRPS21 ABHD14A CBFA2T3 LSS DDB1 RPUSD4 PTP4A2 HSH2D BDH1 SLC25A19 ZADH2 PEX6 PCCB PECAM1 TLR9 SLC25A6 EEF1A1P5 MYCBP ATF6B HDAC1 RPL11 ATP5A1 ASIP VPRBP TULP2 PDHB AIF1 RNF5 HDAC6 CAMKK2 RPL14 METTL7A VHL RPL17 EIF4B CLASP2 GALNT6 NDUFA5 USP20 LAPTM5 RPL23A ACVR2B DEF6 PNRC2 SLC25A38 RPIA SPCS2 ABCB10 LYRM4 EIF3L IMPDH2 LYRM7 NDUFAB1 PDCD2 PKN1 MMACHC
	Organelle inner membrane	1.2633E-4	4.8987E-3	COX8A DNAJC19 GCDH NDUFA5 ABCB7 NDUFB11 ATP5A1 SDHD SLC25A38 ABCB10 BDH1 SLC25A19 NDUFAB1 HADH NDUFV2 SLC25A6
	Chromatin binding	1.4907E-4	5.6656E-3	POLA1 TOP2B HIRA SMARCC1 KDM1A SUV39H1 HESX1 PKN1 PATZ1 SF1 MEN1
	Protein metabolic process	1.5047E-4	5.6656E-3	RPL3 TFRC KDM1A ARAF RPL10A UXT CASP14 MRPL42 CHEK2 PIM2 PGPEP1 RPS10 EIF2A RPS12 RPS9 MRPS25 RPL22 MRPS21 VRK1 RPSA PHKA2 DDB1 LARS2 TAF6L PTP4A2 MAPKAPK3 POLG2 IFNG PDIK1L BTK TLR9 CARD17 PADI4 TTLL12 RPL29 FKBP5 BLK HDAC1 RPL11 MRPL16 RNF5 HDAC6 MRPL11 CAMKK2 DPP3 PCBP2 RPL14 ST8SIA4 VHL DCAF11 RPL17 EIF4B DNAJC19 GALNT6 NEK4 USP20 SIAH2 RPL23A ACVR2B SRPK1 PAN2 RPS25 SPCS2 EIF3L PKN1 STT3B RSL24D1
	Organellar ribosome	1.5527E-4	5.6816E-3	MRPL42 MRPS25 MRPL16 MRPS21 MRPL11 RPS12
	Mitochondrial ribosome	1.5527E-4	5.6816E-3	MRPL42 MRPS25 MRPL16 MRPS21 MRPL11 RPS12
	DNA binding	1.8590E-4	6.7081E-3	TOP2B GMEB1 SPI1 ZNF493 KDM1A ZBTB26 HP1BP3 UBP1 IKZF5 ZNF808 LYL1 SCML2 MYC MYB ZNF41 ZNF845 ZNF445 HMGN2 MEN1 ZBTB39 SMARCC1 TGIF2 ESCO1 KDM2B ZNF160 STRBP HIST1H2AK LMO2 GTF3A TET1 CBFA2T3 DDB1 TAF6L ISL2 POLG2 ZNF639 MSH5 SF1 ZNF397 ZNF551 KHDRBS1 ATF6B MAX HDAC1 ZBTB48 ZBTB3 ZBTB40 HIRA PCBP2 HESX1 ZNF700 H1FX ZNF589 PATZ1 ZNF184 GTF2H4 POLA1 ZNF614 TAF4B PDCD2 ZNF136
	Chromosome organization	1.9804E-4	7.0480E-3	SMARCC1 KDM1AKDM2BHDAC1 EHMT2 SUV39H1 HIST1H2AKHP1BP3 EMSYHDAC6 MBTD1 TAF6LHIRARBBP4 H1FXPADI4 PKN1 NCAPD3 RBBP7 HMGN2 MEN1
	RNA processing	2.2034E-4	7.7357E-3	KHDRBS1 SUV39H1 RPL11 HNRNPRDDX51 TSEN2 RPL10APUS7LADAT2 SRPK1 EXOSC7 SNRNP40 SNRPD1 CLNS1APCBP2 RPL14 INTS5 INTS4 GEMIN8 HNRNPA1L2 SF1 SRSF9
	Regulation of nucleobase,	2.5450E-4	8.8157E-3	CD86 GMEB1 SPI1 ZNF493 KDM1ACDCA7LSUV39H1 ZBTB26 UBP1 IKZF5 ZNF808 SCML2 BCL7AMYCMYBZNF41 ZNF845 ZNF445 HMGN2

	nucleoside, nucleotide and nucleic acid metabolic process			ZNF720 MEN1 ACAD8 ZBTB39 SMARCC1 TGIF2 KDM2BZNF160 GTF3ACBFA2T3 EMSYTAF6LISL2 IFNGEWSR1 ZNF639 TLR9 PADI4 SF1 ZNF397 ZNF551 KHDRBS1 MYCBPATF6BMAXHDAC1 GIPRZBTB48 HDAC6 ZBTB3 ZBTB40 CAMKK2 MBTD1 ATXN3 HIRARBBP4 HESX1 RBBP8 SCAPZNF700 ZNF589 RBBP7 VHLPATZ1 TRAPPC2 ZNF184 GTF2H4 ACVR2BPNRC2 SRPK1 MED13LZNF614 TAF4BPKN1 ZNF136
	Organelle envelope	2.9305E-4	9.9139E-3	NDC1 COX8ADNAJC19 GCDHNDUFA5 ABCB7 NDUFB11 ATP5A1 MTMR8 ATP2A3 TET1 SDHDAAASSLC25A38 POLA1 ABCB10 BDH1 SLC25A19 NDUFAB1 KPNA5 HADHNDUFV2 SLC29A2 SLC25A6
	Ribonucleoprotein complex assembly	2.9383E-4	9.9139E-3	RPS25 SNRPD1 CLNS1A GEMIN8 SF1 SRSF9 EIF2A
	Histone deacetylase complex	3.0567E-4	1.0052E-2	TAF6L RBBP4 HDAC1 RBBP7 HDAC6
	Cytosolic small ribosomal subunit	3.0567E-4	1.0052E-2	RPS25 RPS9 RPSA RPS10 RPS12
	Regulation of nitrogen compound metabolic process	3.3127E-4	1.0758E-2	CD86 GMEB1 SPI1 ZNF493 KDM1ACDCA7LSUV39H1 ZBTB26 UBP1 IKZF5 ZNF808 SCML2 BCL7AMYCMYBZNF41 ZNF845 ZNF445 HMGN2 ZNF720 MEN1 ACAD8 ZBTB39 SMARCC1 TGIF2 KDM2BZNF160 GTF3ACBFA2T3 EMSYTAF6LISL2 IFNGEWSR1 ZNF639 TLR9 PADI4 SF1 ZNF397 ZNF551 KHDRBS1 MYCBPATF6BMAXHDAC1 GIPRZBTB48 HDAC6 ZBTB3 ZBTB40 CAMKK2 MBTD1 ATXN3 HIRARBBP4 HESX1 RBBP8 SCAPZNF700 ZNF589 RBBP7 VHLPATZ1 TRAPPC2 ZNF184 GTF2H4 ACVR2BPNRC2 SRPK1 MED13LZNF614 TAF4BPKN1 ZNF136
	Ribosome biogenesis	3.4989E-4	1.1223E-2	EXOSC7 RPS25 GNL3L SUV39H1 RPL11 RPL14 DDX51 RSL24D1 EIF2A
	Envelope	3.8869E-4	1.2315E-2	NDC1 COX8ADNAJC19 GCDHNDUFA5 ABCB7 NDUFB11 ATP5A1 MTMR8 ATP2A3 TET1 SDHDAAASSLC25A38 POLA1 ABCB10 BDH1 SLC25A19 NDUFAB1 KPNA5 HADHNDUFV2 SLC29A2 SLC25A6
	Cell part	4.7907E-4	1.4951E-2	RPL3 SPI1 TFRCEHMT2 THYN1 HNRNPRNUDT3 IKZF5 UXTFRAT1 SNRPD1 MYCCHEK2 MYBZNF845 ZNF720 RPS10 GLULEIF2ARPS12 SMARCC1 ENDOGATP6V1G2 ESCO1 ZNF160 OXSMRPL22 METTL3 SYTL1 PHKA2 RNF123 LARS2 ISL2 EWSR1 PDE12 CLNS1ABTKC3ORF20 PADI4 RPL29 LETMD1 GTSF1 SF1 ZNF397 MTMR1 GCDHABCB7 EPB41 PDE1BGIPRMRPL16 MTMR8 VPS26BAAASIQGAP2 LY9 ZBTB3 MRPL11 MBTD1 NEU3 DHX30 AFMIDPCBP2 HESX1 ST8SIA4 SCAPNCKAP1LZNF700 CEP192 PATZ1 SIAH2 SRPK1 MED13LRPS25 POLA1 GPR182 INTS5 INTS4 PSMG2 ADCK3 HNRNPA1L2 ZNF136 PRRT4 ARHGAP9 TOP2BGMEB1 ZNF493 SUV39H1 MS4A4ACASP14 MRPL42 ZNF808 SCML2 ZNF41 CD37 DLATOFD1 NDC1 ACAD8 COX8AMRPS25 STRBPTET1 TMC8 MRPS21 CBFA2T3 LIME1 GNL3 DDB1 RPUSD4 TAF6LKIF9 BDH1 PDIK1LZADH2 TLR9 TUT1 CD48 PTMASLC29A2 MYCBPRPL11 ATP5A1 ASIPAIF1 CAMKK2 TMEM69 EXOSC7 HIRARNF138 RPL14 ZNF589 METTL7ARPL17 LAIR1 LAPTM5 GTF2H4 DEF6 SPCS2 ABCB10 LYRM4 LYRM7 NDUFAB1 PKN1 NCAPD3 CD86 KDM1AZBTB26 UBP1 TSEN2 RPL10ABZRAP1 FAM162AMACROD1 KPNA5 ZNF445 HMGN2 HADHMEN1 ZBTB39 RPS9 SLC15A2 KDM2BCD300AHIST1H2AKLMO2 GTF3AVRK1 RPSASDHDEMSYTMEM135 MAPKAPK3 POLG2 CARD17 GEMIN8 SRSF9 FKBP5 ZNF551 KHDRBS1 MAXNDUFB11 ARHGAP19 PLD4 RASGRP2 CORO1ADPP3 ATXN3 H1FXNDUFV2 CLCA4 MDN1 TRAPPC2 DNAJC19 PDHA1 NEK4 ANKRD23 LY86 SELPPAN2 RPS10P5 GNL3LTAF4BSTT3BRSL24D1 CDCA7LAMD1 NGLY1 HP1BP3 ATP2A3 LRMPLSP1 ITGALLYL1 ZNRD1 TAGAPPGPEP1 ADSLTGIF2 ACTR6 GLRX5 SH2D3CABHD14ADDX51 LSSPTP4A2 HSH2DSLC25A19 ZNF639 PEX6 PCCBPECAM1 LCTSLC25A6 EEF1A1P5 ATF6BHDAC1 RGS16 ZBTB48 VPRBPTULP2 PDHBRETNRNF5 HDAC6 ZBTB40 P2RY8 RBBP4 RBBP8 RBBP7 VHLDCAF11 EIF4BCLASP2 GALNT6 NDUFA5 ZNF184 USP20 RPL23AACVR2BPNRC2 SLC25A38 RPIASNRNP40 EIF3LIMPDH2 ZNF614 PDCD2 ZNF852 MMACHC
	Cell	4.8339E-4	1.4951E-2	RPL3 SPI1 TFRCEHMT2 THYN1 HNRNPRNUDT3 IKZF5 UXTFRAT1 SNRPD1 MYCCHEK2 MYBZNF845 ZNF720 RPS10 GLULEIF2ARPS12 SMARCC1 ENDOGATP6V1G2 ESCO1 ZNF160 OXSMRPL22 METTL3 SYTL1 PHKA2 RNF123 LARS2 ISL2 EWSR1 PDE12 CLNS1ABTKC3ORF20 PADI4 RPL29 LETMD1 GTSF1 SF1 ZNF397 MTMR1 GCDHABCB7 EPB41 PDE1BGIPRMRPL16 MTMR8 VPS26BAAASIQGAP2 LY9 ZBTB3 MRPL11 MBTD1 NEU3 DHX30 AFMIDPCBP2 HESX1 ST8SIA4 SCAPNCKAP1LZNF700 CEP192 PATZ1 SIAH2 SRPK1 MED13LRPS25 POLA1 GPR182 INTS5 INTS4 PSMG2 ADCK3 HNRNPA1L2 ZNF136

				PRRT4 ARHGAP9 TOP2BGMEB1 ZNF493 SUV39H1 MS4A4ACASP14 MRPL42 ZNF808 SCML2 ZNF41 CD37 DLATOFD1 NDC1 ACAD8 COX8AMRPS25 STRBPTET1 TMC8 MRPS21 CBFA2T3 LIME1 GNL3 DDB1 RPUSD4 TAF6LKIF9 BDH1 PDIK1LZADH2 TLR9 TUT1 CD48 PTMASLC29A2 MYCBPRPL11 ATP5A1 ASIPAIF1 CAMKK2 TMEM69 EXOSC7 HIRARNF138 RPL14 ZNF589 METTL7ARPL17 LAIR1 LAPTM5 GTF2H4 DEF6 SPCS2 ABCB10 LYRM4 LYRM7 NDUFAB1 PKN1 NCAPD3 CD86 KDM1AZBTB26 UBP1 TSEN2 RPL10ABZRAP1 FAM162AMACROD1 KPNA5 ZNF445 HMGN2 HADHMEN1 ZBTB39 RPS9 SLC15A2 KDM2BCD300AHIST1H2AKLMO2 GTF3AVRK1 RPSASDHDEMSYTMEM135 MAPKAPK3 POLG2 CARD17 GEMIN8 SRSF9 FKBP5 ZNF551 KHDRBS1 MAXNDUFB11 ARHGAP19 PLD4 RASGRP2 CORO1ADPP3 ATXN3 H1FXNDUFV2 CLCA4 MDN1 TRAPPC2 DNAJC19 PDHA1 NEK4 ANKRD23 LY86 SELPPAN2 RPS10P5 GNL3LTAF4BSTT3BRSL24D1 CDCA7LAMD1 NGLY1 HP1BP3 ATP2A3 LRMPLSP1 ITGALLYL1 ZNRD1 TAGAPPGPEP1 ADSLTGIF2 ACTR6 GLRX5 SH2D3CABHD14ADDX51 LSSPTP4A2 HSH2DSLC25A19 ZNF639 PEX6 PCCBPECAM1 LCTSLC25A6 EEF1A1P5 ATF6BHDAC1 RGS16 ZBTB48 VPRBPTULP2 PDHBRETNRNF5 HDAC6 ZBTB40 P2RY8 RBBP4 RBBP8 RBBP7 VHLDCAF11 EIF4BCLASP2 GALNT6 NDUFA5 ZNF184 USP20 RPL23AACVR2BPNRC2 SLC25A38 RPIASNRNP40 EIF3LIMPDH2 ZNF614 PDCD2 ZNF852 MMACHC
	Regulation of receptor biosynthetic process	5.0298E-4	1.5374E-2	HDAC1 SCAP HDAC6
	Cellular component biogenesis	5.4107E-4	1.6345E-2	MAXSUV39H1 RPL11 ASIPHP1BP3 CORO1AAIF1 HDAC6 EXOSC7 SNRPD1 RPL14 CEP192 H1FXVHLMDN1 EIF2AOFD1 NDC1 ADSLHIST1H2AKDDX51 GTF2H4 TAF6LRPS25 GNL3LCLNS1ATAF4BPSMG2 GEMIN8 RSL24D1 SF1 SRSF9
	Histone deacetylase binding	5.6775E-4	1.6954E-2	TOP2B RBBP4 HDAC1 PKN1 HDAC6
	Cytosol	6.1207E-4	1.7941E-2	TOP2BRPL3 HDAC1 PDE1BRPL11 AMD1 VPS26BRPL10ARASGRP2 HDAC6 UXTSNRPD1 AFMIDRPL14 CEP192 VHLPGPEP1 RPL17 RPS10 GLULEIF4BMEN1 EIF2ARPS12 OFD1 RPS9 ADSLRPL22 RPSARPL23ARPIARPS25 MAPKAPK3 CLNS1AIMPDH2 PEX6 BTKRPL29
	Binding	6.1460E-4	1.7941E-2	RPL3 SPI1 CLPBEHMT2 ARAFHNRNPRNUDT3 IKZF5 UXTPPP2R1BSNRPD1 MYCCHEK2 MYBZNF845 ZNF720 RPS10 GLULEIF2ARPS12 SMARCC1 ENDOGATP6V1G2 ESCO1 ZNF160 RPL22 METTL3 SYTL1 RNASE6 PHKA2 RNF123 LARS2 ISL2 EWSR1 CLNS1ABTKPADI4 RPL29 LETMD1 GTSF1 SF1 ZNF397 GCDHABCB7 EPB41 PDE1BGIPRMRPL16 MTMR8 IQGAP2 ZBTB3 MBTD1 DHX30 PCBP2 HESX1 SCAPZNF700 PATZ1 SIAH2 PAICSSRPK1 RPS25 POLA1 GPR182 INTS5 INTS4 PSMG2 ADCK3 HNRNPA1L2 ZNF136 ARHGAP9 TOP2BGMEB1 ZNF493 SUV39H1 ZNF808 SCML2 ZNF41 DLATFGFBP3 OFD1 NDC1 ACAD8 ZC3H10 MRPS25 STRBPTET1 CBFA2T3 GNL3 DDB1 RPUSD4 TAF6LKIF9 BDH1 PDIK1LZADH2 TLR9 TUT1 CD48 RNF165 MYCBPRPL11 ATP5A1 ASIPAIF1 CAMKK2 EXOSC7 HIRARNF138 RPL14 ZNF589 RPL17 LAIR1 PUS7LGTF2H4 DEF6 ADAT2 ABCB10 NDUFAB1 PKN1 NCAPD3 CD86 KDM1AZBTB26 UBP1 LZICTSEN2 RPL10ABZRAP1 PIM2 KPNA5 ZNF445 HMGN2 HADHMEN1 ZBTB39 RPS9 SLC15A2 KDM2BHIST1H2AKLMO2 GTF3AVRK1 RPSASDHDATAD3AMAPKAPK3 POLG2 MSH5 CARD17 GEMIN8 SRSF9 FKBP5 ZNF551 BLKKHDRBS1 MAXPLD4 RASGRP2 CORO1ADPP3 ATXN3 H1FXNDUFV2 MDN1 TRAPPC2 DNAJC19 PDHA1 NEK4 LY86 CNOT10 SELPPAN2 GNL3LTAF4BSTT3BDOCK8 NGLY1 HP1BP3 ATP2A3 LSP1 ITGALLYL1 ZNRD1 TGIF2 ACTR6 GLRX5 SH2D3CMSL2 DDX51 PTP4A2 HSH2DIFNGSLC25A19 ZNF639 PEX6 PCCBPECAM1 LCTSLC25A6 EEF1A1P5 ATF6BHDAC1 RGS16 ZBTB48 VPRBPRETNRNF5 HDAC6 ZBTB40 RBBP4 RBBP8 RBBP7 VHLDCAF11 EIF4BCLASP2 GALNT6 ZNF184 USP20 RPL23AACVR2BPNRC2 SLC25A38 SNRNP40 EIF3LIMPDH2 ZNF614 PDCD2 ZNF852 MMACHC
	Ncrna metabolic process	6.2309E-4	1.7987E-2	EXOSC7 LARS2 POLG2 SUV39H1 RPL11 RPL14 INTS5 INTS4 DDX51 TSEN2 PUS7L ADAT2
	Nurd complex	6.8310E-4	1.9502E-2	RBBP4 HDAC1 RBBP7
	Pyruvate dehydrogenase complex	8.2002E-4	2.2425E-2	PDHB DLAT

	Pyruvate dehydrogenase activity	8.2002E-4	2.2425E-2	PDHA1 PDHB
	Pyruvate dehydrogenase (acetyl-transferring) activity	8.2002E-4	2.2425E-2	PDHA1 PDHB
	Misfolded or incompletely synthesized protein catabolic process	8.2002E-4	2.2425E-2	RNF5 HDAC6
	Regulation of acetyl-coa biosynthetic process from pyruvate	8.9961E-4	2.4346E-2	PDHA1 PDHB DLAT
	Regulation of cofactor metabolic process	1.4521E-3	3.8496E-2	PDHA1 PDHB DLAT
	Regulation of coenzyme metabolic process	1.4521E-3	3.8496E-2	PDHA1 PDHB DLAT
	Ncrna processing	1.5139E-3	3.9729E-2	EXOSC7 SUV39H1 RPL11 RPL14 INTS5 INTS4 DDX51 TSEN2 PUS7L ADAT2
	Mitochondrial membrane	1.5595E-3	4.0516E-2	COX8ADNAJC19 GCDHNDUFA5 ABCB7 NDUFB11 ATP5A1 SDHDSLC25A38 ABCB10 BDH1 SLC25A19 NDUFAB1 HADHNDUFV2 SLC25A6
	Mitochondrial membrane part	1.5889E-3	4.0871E-2	ABCB10 NDUFA5 NDUFB11 ATP5A1 NDUFAB1 SDHD NDUFV2 SLC25A6
	Cellular response to stress	1.6874E-3	4.2978E-2	CLPBESCO1 MAXSH2D3CGTF2H4 RNF5 EMSYHDAC6 DDB1 ATXN3 POLA1 POLG2 IFNGCHEK2 MSH5 RBBP8 SCAPPKN1 RBBP7 MEN1 EIF2A
	Eukaryotic cell surface binding	1.7929E-3	4.5223E-2	SELP ATP5A1 CD48
	Catalytic activity	1.9709E-3	4.9234E-2	OTUD5 CLPBTFRCKDM1AEHMT2 ARAFTSEN2 NUDT3 CHEK2 PIM2 HADHGLULENDOGATP6V1G2 ESCO1 KDM2BOXSMMETTL3 RNASE6 VRK1 SDHDHDHD2 PHKA2 ATAD3ARNF123 LARS2 MAPKAPK3 POLG2 PDE12 BTKCARD17 PADI4 TTLL12 FKBP5 BLKMTMR1 GCDHABCB7 PDE1BMTMR8 PLD4 DPP3 ATXN3 NEU3 DHX30 AFMIDST8SIA4 NDUFV2 MDN1 PDHA1 NEK4 SIAH2 PAICSSRPK1 PAN2 POLA1 ADCK3 STT3BTOP2BSUV39H1 AMD1 NGLY1 ATP2A3 CASP14 ZNRD1 DLATPGPEP1 ACAD8 COX8AADSLGLRX5 TET1 ABHD14ADDX51 LSSRPUSD4 PTP4A2 KIF9 BDH1 PDIK1LZADH2 PEX6 PCCBTUT1 LCTEEF1A1P5 HDAC1 ATP5A1 PDHBRNF5 HDAC6 CAMKK2 EXOSC7 RNF138 METTL7ACLASP2 GALNT6 NDUFA5 USP20 PUS7LACVR2BADAT2 RPIASPCS2 ABCB10 IMPDH2 NDUFAB1 PKN1
**32**	Plasma membrane	9.5852E-10	1.6270E-6	ALKRYR2 KIR3DS1 AQP9 PRF1 TNFLTB4RHTR6 OR10W1 TNFRSF8 ERC2 CCR5 KIRREL2 KCND3 SYN1 RAB33ATMPRSS11DNCR1 HCKELMO1 CD226 TLR10 CD300CCSF1RNOD1 KIR2DL1 NOD2 KIR2DL2 KIR2DL3 KIR2DL4 CALHM1 PDE6CICOSIL12RB1 KIR2DL5AKIR2DS1 KIR2DS2 KIR2DS3 KIR2DS4 KIR2DS5 SLC4A10 OSMNKG7 GZMBKIR3DL1 KIR3DL2 KIR3DL3 SNX20 IL2RAIL2RBSLC26A4 PIK3AP1
	Signal transducer activity	2.7701E-9	1.6270E-6	ALKCSF1RRYR2 KIR3DS1 SH2D2ARORCRORAKIR2DL1 KIR2DL2 KIR2DL3 KIR2DL4 LTB4RHTR6 OR10W1 TNFRSF8 RGS22 CCR5 IL12RB1 JAK3 KIR2DL5AKIR2DS1 KIR2DS2 KIR2DS3 KIR2DS4 KIR2DS5 WNT9BKIR3DL1 KIR3DL2 KIR3DL3 NCR1 IL2RAIL2RBCD226 TLR10 CD300CPIK3AP1 CLEC4F
	Molecular transducer activity	2.7701E-9	1.6270E-6	ALKCSF1RRYR2 KIR3DS1 SH2D2ARORCRORAKIR2DL1 KIR2DL2 KIR2DL3 KIR2DL4 LTB4RHTR6 OR10W1 TNFRSF8 RGS22 CCR5 IL12RB1 JAK3 KIR2DL5AKIR2DS1 KIR2DS2 KIR2DS3 KIR2DS4 KIR2DS5 WNT9BKIR3DL1 KIR3DL2 KIR3DL3 NCR1 IL2RAIL2RBCD226 TLR10 CD300CPIK3AP1 CLEC4F
	Receptor activity	5.9428E-9	2.6178E-6	ALKCSF1RRYR2 KIR3DS1 RORCRORAKIR2DL1 KIR2DL2 KIR2DL3 KIR2DL4 LTB4RHTR6 OR10W1 TNFRSF8 CCR5 IL12RB1 KIR2DL5AKIR2DS1 KIR2DS2 KIR2DS3 KIR2DS4 KIR2DS5 KIR3DL1

				KIR3DL2 KIR3DL3 NCR1 IL2RAIL2RBCD226 TLR10 CD300CCLEC4F
	Immune response	8.4818E-9	2.9890E-6	IL10 SERPINB4 KIR3DS1 KIR2DS1 KIR2DS2 AQP9 KIR2DS5 OSMKIR3DL1 KIR2DL1 NOD2 TNFKIR2DL3 LTB4RIL2RAIL9 TLR10 ICOSCCR5
	Integral to plasma membrane	1.0850E-8	3.1864E-6	ALKCSF1RKIR3DS1 AQP9 KIR2DL1 TNFKIR2DL3 KIR2DL4 LTB4RHTR6 ICOSCCR5 IL12RB1 KCND3 KIR2DS3 KIR2DS4 KIR2DS5 OSMNKG7 KIR3DL1 KIR3DL2 TMPRSS11DNCR1 IL2RBCD226 CD300C
	Intrinsic to plasma membrane	1.5593E-8	3.9250E-6	ALKCSF1RKIR3DS1 AQP9 KIR2DL1 TNFKIR2DL3 KIR2DL4 LTB4RHTR6 ICOSCCR5 IL12RB1 KCND3 KIR2DS3 KIR2DS4 KIR2DS5 OSMNKG7 KIR3DL1 KIR3DL2 TMPRSS11DNCR1 IL2RBCD226 CD300C
	Plasma membrane part	2.6971E-8	5.9404E-6	ALKCSF1RKIR3DS1 AQP9 NOD1 KIR2DL1 TNFKIR2DL3 KIR2DL4 LTB4RHTR6 ICOSERC2 CCR5 IL12RB1 KCND3 KIR2DS3 KIR2DS4 KIR2DS5 SLC4A10 OSMNKG7 GZMBKIR3DL1 KIR3DL2 SYN1 TMPRSS11DNCR1 HCKIL2RAIL2RBCD226 CD300CSLC26A4
	Immune system process	7.6842E-8	1.5044E-5	IL10 SERPINB4 KIR3DS1 KIR2DS1 KIR2DS2 AQP9 ZBTB32 KIR2DS5 OSMKIR3DL1 KIR2DL1 NOD2 TNFKIR2DL3 LTB4RNCR1 THPOIL2RAIL9 TLR10 ICOSCCR5
	Cellular defense response	1.0071E-7	1.7746E-5	NCR1 KIR2DS3 PRF1 CD300C KIR3DL2 CCR5 KIR2DL4
	Defense response	2.8818E-7	4.6162E-5	IL10 KIR2DS3 PRF1 NOD1 NOD2 KIR3DL2 TNFKIR2DL4 LTB4RNCR1 HCKIL2RAIL9 TLR10 IL17FCD300CCCR5
	Cytokine binding	3.4838E-7	5.1154E-5	CSF1R IL2RA IL2RB CHRD TNFRSF8 IL17F CCR5 IL12RB1
	Regulation of cytokine production	4.8957E-6	6.6355E-4	IL10 IL26 IL9 ZBTB32 TNFRSF8 NOD1 IL17F NOD2 TNF
	Regulation of interleukin-6 production	6.1206E-6	7.7032E-4	IL10 NOD1 IL17F NOD2 TNF
	Membrane	1.0046E-5	1.1801E-3	ALKRYR2 KIR3DS1 AQP9 RASL11APRF1 ABCA13 TNFLTB4RHTR6 OR10W1 TNFRSF8 ERC2 CCR5 JAK3 HAVCR2 KIRREL2 KCND3 SEZ6LAP3B2 SYTL3 SYN1 RAB33ATMPRSS11DNCR1 HCKSLC25A18 ELMO1 CD226 TLR10 CD300CCLEC4FCSF1RNOD1 KIR2DL1 NOD2 KIR2DL2 JAKMIP1 KIR2DL3 KIR2DL4 STX11 CALHM1 PDE6CICOSIL12RB1 SLC15A4 SEC31BKIR2DL5AKIR2DS1 KIR2DS2 KIR2DS3 KIAA1024LKIR2DS4 KIR2DS5 SLC4A10 OSMNKG7 GZMBBTBD11 KIR3DL1 KIR3DL2 KIR3DL3 TM6SF1 SNX20 IL2RAIL2RBSLC26A4 PIK3AP1
	Positive regulation of stress-activated protein kinase signaling cascade	2.2576E-5	2.4862E-3	IL26 NOD1 NOD2 TNF
	Positive regulation of cytokine secretion	2.6213E-5	2.7169E-3	IL10 IL26 NOD2 TNF
	Interleukin-2 receptor activity	3.8604E-5	3.5800E-3	IL2RA IL2RB
	Inhibitory MHC class I receptor activity	3.8604E-5	3.5800E-3	KIR2DS5 KIR3DL1
	Positive regulation of stress-activated MAPK cascade	4.9963E-5	4.2912E-3	IL26 NOD1 NOD2
	Positive regulation of ERK1 and ERK2 cascade	5.1143E-5	4.2912E-3	IL26 NOD1 NOD2 TNF
	Regulation of tumor necrosis factor production	5.7685E-5	4.6200E-3	IL10 TNFRSF8 NOD1 NOD2
	Regulation of defense	6.7490E-5	5.1703E-3	IL10 NCR1 IL2RA OSM CD226 NOD2 TNF

	response			
	Regulation of cytokine secretion	7.2568E-5	5.3277E-3	IL10 IL26 NOD2 TNF
	Regulation of response to stimulus	1.0532E-4	7.0982E-3	IL10 NCR1 SOCS1 IL26 IL2RA OSM CD226 NOD1 KIR2DL1 NOD2 ICOS TNF
	Signaling	1.0679E-4	7.0982E-3	ALKCSF1RIL26 SH2D2AHTRA3 RASL11ACHRDRORANOD1 KIR2DL1 NOD2 TNFKIR2DL4 LTB4RHTR6 PSTPIP1 SOCS1 TNFRSF8 PDE6CCCR5 IL12RB1 JAK3 IL10 WNT9BOSMCIDEBSYN1 RAB33ANCR1 ACAP1 IL2RAIL2RBELMO1 CD226 TLR10 FGF11
	Interleukin-2 binding	1.1534E-4	7.0982E-3	IL2RA IL2RB
	Regulation of cytokine biosynthetic process	1.1953E-4	7.0982E-3	IL10 IL9 TNFRSF8 IL17F TNF
	Regulation of ERK1 and ERK2 cascade	1.2180E-4	7.0982E-3	IL26 NOD1 NOD2 TNF
	Regulation of stress-activated MAPK cascade	1.2488E-4	7.0982E-3	IL26 NOD1 NOD2
	MHC class I receptor activity	1.2488E-4	7.0982E-3	KIR3DS1 KIR2DS5 KIR3DL1
	Inflammatory response	1.5896E-4	8.0917E-3	IL10 IL2RA IL9 TLR10 NOD1 IL17F CCR5 TNF LTB4R
	Cytokine production	1.6094E-4	8.0917E-3	CD226 NOD1 IL17F NOD2
	Positive regulation of transcription factor activity	1.6104E-4	8.0917E-3	IL10 TNFRSF8 NOD1 NOD2 TNF
	Positive regulation of transcription regulator activity	1.6104E-4	8.0917E-3	IL10 TNFRSF8 NOD1 NOD2 TNF
	Membrane part	1.6533E-4	8.0917E-3	ALKRYR2 KIR3DS1 AQP9 PRF1 ABCA13 TNFLTB4RHTR6 OR10W1 TNFRSF8 ERC2 CCR5 HAVCR2 KIRREL2 KCND3 SEZ6LAP3B2 SYN1 TMPRSS11DNCR1 HCKSLC25A18 CD226 TLR10 CD300CCLEC4FCSF1RNOD1 KIR2DL1 KIR2DL2 KIR2DL3 KIR2DL4 CALHM1 ICOSIL12RB1 SLC15A4 SEC31BKIR2DL5AKIR2DS1 KIR2DS2 KIR2DS3 KIAA1024LKIR2DS4 KIR2DS5 SLC4A10 OSMNKG7 GZMBBTBD11 KIR3DL1 KIR3DL2 KIR3DL3 TM6SF1 IL2RAIL2RBSLC26A4
	Positive regulation of MAPKKK cascade	1.7052E-4	8.1005E-3	IL26 OSM NOD1 NOD2 TNF
	Regulation of response to stress	1.7470E-4	8.1005E-3	IL10 NCR1 IL26 IL2RA OSM CD226 NOD1 NOD2 TNF
	Signal transduction	1.8871E-4	8.5257E-3	CSF1RSH2D2AHTRA3 RASL11ARORANOD1 TNFKIR2DL4 LTB4RHTR6 PSTPIP1 SOCS1 TNFRSF8 PDE6CIL12RB1 JAK3 IL10 OSMRAB33ANCR1 IL2RBELMO1 CD226 TLR10 FGF11
	Positive regulation of protein secretion	2.0844E-4	9.1609E-3	IL10 IL26 NOD2 TNF
	Cytolysis	2.1316E-4	9.1609E-3	PRF1 GZMB GZMH
	External side of plasma membrane	2.1854E-4	9.1683E-3	IL2RA IL2RB ICOS CCR5 IL12RB1 TNF
	Receptor biosynthetic process	2.2974E-4	9.1999E-3	IL10 TNF
	Negative	2.2974E-4	9.1999E-3	IL10 TNF

	regulation of cytokine secretion involved in immune response			
	Cytokine activity	2.3640E-4	9.2565E-3	IL10 THPO IL26 IL9 OSM IL17F TNF
	Regulation of lymphocyte mediated immunity	2.6529E-4	1.0162E-2	IL10 NCR1 CD226 NOD2
	Positive regulation of JNK cascade	2.8993E-4	1.0238E-2	NOD1 NOD2 TNF
	Regulation of B cell mediated immunity	2.8993E-4	1.0238E-2	IL10 CD226 NOD2
	Regulation of immunoglobuli n mediated immune response	2.8993E-4	1.0238E-2	IL10 CD226 NOD2
	Positive regulation of DNA binding	2.9052E-4	1.0238E-2	IL10 TNFRSF8 NOD1 NOD2 TNF
	Positive regulation of interleukin-6 production	3.3419E-4	1.1546E-2	NOD1 NOD2 TNF
	Regulation of cytokine secretion involved in immune response	3.8133E-4	1.2752E-2	IL10 TNF
	Positive regulation of NF-kappab transcription factor activity	3.8358E-4	1.2752E-2	TNFRSF8 NOD1 NOD2 TNF
	Integral to membrane	3.9457E-4	1.2875E-2	ALKRYR2 KIR3DS1 AQP9 PRF1 ABCA13 TNFLTB4RHTR6 OR10W1 TNFRSF8 CCR5 HAVCR2 KIRREL2 KCND3 SEZ6LTMPRSS11DNCR1 SLC25A18 CD226 TLR10 CD300CCLEC4FCSF1RKIR2DL1 KIR2DL2 KIR2DL3 KIR2DL4 CALHM1 ICOSIL12RB1 SLC15A4 KIR2DL5AKIR2DS1 KIR2DS2 KIR2DS3 KIAA1024LKIR2DS4 KIR2DS5 SLC4A10 OSMNKG7 BTBD11 KIR3DL1 KIR3DL2 KIR3DL3 TM6SF1 IL2RAIL2RBSLC26A4
	Regulation of leukocyte mediated immunity	5.0218E-4	1.6007E-2	IL10 NCR1 CD226 NOD2
	Regulation of immune effector process	5.0875E-4	1.6007E-2	IL10 NCR1 CD226 NOD2 TNF
	Negative regulation of T cell proliferation	5.5406E-4	1.7013E-2	IL10 IL2RA ICOS
	Inflammatory response to antigenic stimulus	5.6966E-4	1.7013E-2	IL2RA TNF
	Negative regulation of interleukin-12 production	5.6966E-4	1.7013E-2	IL10 NOD2
	Positive regulation of	6.0351E-4	1.7634E-2	IL10 TNFRSF8 NOD1 NOD2 TNF

	binding			
	Response to bacterium	6.1589E-4	1.7634E-2	IL10 HCK SOCS1 THPO NOD1 NOD2 TNF
	Defense response to Gram-positive bacterium	6.2049E-4	1.7634E-2	HCK NOD1 NOD2
	Signaling process	6.5034E-4	1.7905E-2	CSF1RSH2D2AHTRA3 RASL11ARORANOD1 TNFKIR2DL4 LTB4RHTR6 PSTPIP1 SOCS1 TNFRSF8 PDE6CIL12RB1 JAK3 IL10 OSMSYN1 RAB33ANCR1 IL2RBELMO1 CD226 TLR10 FGF11
	Signal transmission	6.5034E-4	1.7905E-2	CSF1RSH2D2AHTRA3 RASL11ARORANOD1 TNFKIR2DL4 LTB4RHTR6 PSTPIP1 SOCS1 TNFRSF8 PDE6CIL12RB1 JAK3 IL10 OSMSYN1 RAB33ANCR1 IL2RBELMO1 CD226 TLR10 FGF11
	Intrinsic to membrane	6.6490E-4	1.8024E-2	ALKRYR2 KIR3DS1 AQP9 PRF1 ABCA13 TNFLTB4RHTR6 OR10W1 TNFRSF8 CCR5 HAVCR2 KIRREL2 KCND3 SEZ6LTMPRSS11DNCR1 SLC25A18 CD226 TLR10 CD300CCLEC4FCSF1RKIR2DL1 KIR2DL2 KIR2DL3 KIR2DL4 CALHM1 ICOSIL12RB1 SLC15A4 KIR2DL5AKIR2DS1 KIR2DS2 KIR2DS3 KIAA1024LKIR2DS4 KIR2DS5 SLC4A10 OSMNKG7 BTBD11 KIR3DL1 KIR3DL2 KIR3DL3 TM6SF1 IL2RAIL2RBSLC26A4
	Regulation of immune response	6.8163E-4	1.8198E-2	IL10 NCR1 CD226 KIR2DL1 NOD2 ICOS TNF
	Regulation of protein secretion	7.2591E-4	1.9090E-2	IL10 IL26 NOD2 TNF
	Transmembrane receptor activity	7.9247E-4	2.0283E-2	ALKCSF1RKIR3DS1 KIR2DS1 KIR2DS2 KIR2DS5 KIR3DL1 KIR2DL4 LTB4RHTR6 OR10W1 IL2RAIL2RBTNFRSF8 TLR10 CD300CCCR5 IL12RB1
	CARD domain binding	7.9427E-4	2.0283E-2	NOD1 NOD2
	Defense response to bacterium	8.9814E-4	2.2602E-2	IL10 HCK NOD1 NOD2 TNF
	Negative regulation of mononuclear cell proliferation	9.3640E-4	2.2602E-2	IL10 IL2RA ICOS
	Negative regulation of leukocyte proliferation	9.3640E-4	2.2602E-2	IL10 IL2RA ICOS
	Negative regulation of lymphocyte proliferation	9.3640E-4	2.2602E-2	IL10 IL2RA ICOS
	Positive regulation of B cell mediated immunity	1.0547E-3	2.4779E-2	CD226 NOD2
	Positive regulation of immunoglobulin mediated immune response	1.0547E-3	2.4779E-2	CD226 NOD2
	Response to stress	1.2649E-3	2.9326E-2	IL10 RYR2 KIR2DS3 AQP9 PRF1 OSMCIDEBNOD1 NOD2 KIR3DL2 TNFKIR2DL4 LTB4RNCR1 HCKIL2RAIL9 ALBTLR10 IL17FCD300CCCR5
	Cell surface	1.3159E-3	3.0113E-2	IL2RA IL2RB CD226 NOD2 ICOS CCR5 IL12RB1 TNF
	Peptidoglycan binding	1.3505E-3	3.0205E-2	NOD1 NOD2
	Regulation of immune system process	1.3543E-3	3.0205E-2	IL10 NCR1 IL2RA RORA CD226 KIR2DL1 NOD2 ICOS TNF
	Regulation of transcription factor activity	1.6281E-3	3.5416E-2	IL10 TNFRSF8 NOD1 NOD2 TNF
	Regulation of transcription	1.6281E-3	3.5416E-2	IL10 TNFRSF8 NOD1 NOD2 TNF

	regulator activity			
	Negative regulation of cytokine secretion	1.6813E-3	3.5692E-2	IL10 TNF
	Negative regulation of interleukin-6 production	1.6813E-3	3.5692E-2	IL10 TNF
	Regulation of stress-activated protein kinase signaling cascade	1.8081E-3	3.7481E-2	IL26 NOD1 NOD2 TNF
	Positive regulation of defense response	1.8081E-3	3.7481E-2	OSM CD226 NOD2 TNF
	Peptidyl-tyrosine phosphorylatio n	1.8361E-3	3.7619E-2	OSM IL12RB1 JAK3
	Response to stimulus	1.9436E-3	3.9121E-2	RYR2 KIR3DS1 AQP9 PRF1 NOD1 KIR2DL1 NOD2 TNFKIR2DL3 KIR2DL4 LTB4RSOCS1 THPOOR10W1 PDE6CICOSCCR5 IL10 SERPINB4 KIR2DS1 KIR2DS2 KIR2DS3 KIR2DS5 WNT9BOSMCIDEBKIR3DL1 KIR3DL2 NCR1 HCKIL2RAIL9 ALBTLR10 IL17FCD300C
	Positive regulation of protein transport	1.9734E-3	3.9121E-2	IL10 IL26 NOD2 TNF
	Regulation of production of molecular mediator of immune response	1.9760E-3	3.9121E-2	IL10 NOD2 TNF
	Regulation of tumor necrosis factor biosynthetic process	2.0465E-3	4.0067E-2	IL10 TNFRSF8
	Peptidyl-tyrosine modification	2.1224E-3	4.1096E-2	OSM IL12RB1 JAK3
	Signaling pathway	2.2724E-3	4.3110E-2	ALKIL10 CSF1RRASL11ACHRDWNT9BOSMCIDEBNOD1 KIR2DL1 NOD2 TNFLTB4RRAB33AHTR6 ACAP1 SOCS1 IL2RAIL2RBELMO1 TLR10 CCR5 IL12RB1 JAK3
	Negative regulation of T cell activation	2.2754E-3	4.3110E-2	IL10 IL2RA ICOS
	Protein binding	2.3458E-3	4.3972E-2	ALKRYR2 IL26 RASL11APRF1 RORATNFHTR6 PSTPIP1 TNFRSF8 NEFMERC2 CCR5 JAK3 SERPINB3 IL10 SERPINB4 BATF3 MYBPC2 KCND3 ZBTB32 AP3B2 SYTL3 SYN1 RAB33AHCKACAP1 ELMO1 CD226 TLR10 ATF3 CSF1RZBTB46 SH2D2AHTRA3 CHRDNOD1 KIR2DL1 NOD2 JAKMIP1 KIR2DL3 KIR2DL4 C1ORF94 STX11 MTMR7 CALHM1 SOCS1 THPOICOSIL12RB1 TRIM43 SH2D1BTDRD9 OSMCIDEBGZMBBTBD11 SNX20 IL2RAIL9 ALBIL2RBIL17FPIK3AP1 FGF11 MYO1G
	Regulation of inflammatory response	2.4318E-3	4.4892E-2	IL10 IL2RA OSM TNF
	Detection of bacterium	2.4459E-3	4.4892E-2	NOD1 NOD2
	Negative regulation of immune system process	2.5315E-3	4.5985E-2	IL10 IL2RA NOD2 ICOS
	Negative regulation of cytokine	2.6014E-3	4.6772E-2	IL10 NOD2 TNF

	production			

[1] P value was calculated using the hypergeometric test and corrected for multiple hypothesis testing ($P < 0.05$) using the Benjamini-Hochberg false discovery rate (FDR) adjustment.
